# Analysis of Cyclohexa-1,4-dienes with an Asymmetrically Substituted, 3° Carbon Atom as Chiral Dihydrogen Surrogates in Reagent-Controlled Transfer Hydrogenation of Alkenes

**DOI:** 10.1021/acs.joc.5c02009

**Published:** 2025-10-02

**Authors:** Paul E. Rucker, Martin Oestreich

**Affiliations:** Institut für Chemie, 26524 Technische Universität Berlin, Strasse des 17. Juni 115, 10623 Berlin, Germany

## Abstract

Building on a reagent-controlled, trityl-cation-initiated enantioselective transfer hydrogenation of alkenes recently developed by us, a broad set of chiral cyclohexa-1,4-diene-based dihydrogen surrogates was synthesized and applied to the reduction of 1,1-disubstituted alkenes. The expanded structural diversity allowed for a more refined investigation of the structure-selectivity relationship. The experimental results are in agreement with earlier computational predictions and highlight the role of steric factors proximal (gray circle) and distal (black circle) to the stereocenter in governing the efficiency of transfer hydrogenation.

## Graphical abstract

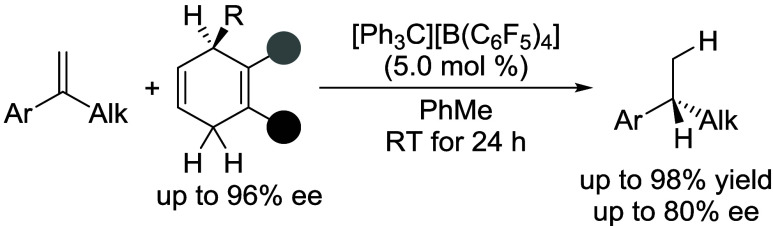

To this day, carbocations remain difficult to capture. Being one of the four key reactive intermediates in organic chemistry, these cations are central to countless transformations and continue to be the subject of a wide variety of research.^1^ Due to their fleeting nature along with their strong desire to even react with weak nucleophiles, discrimination between the two enantiotopic faces of prochiral planar carbenium ions is especially challenging.^2^ Early successes in this field were achieved by Olah and Bach with α-chiral carbenium ions relying on substrate control.^3–5^ Particularly noteworthy are advances by List and co-workers who made use of chiral counteranions under catalyst control,^6,7^ and this laboratory was even able to conduct enantioselective Friedel–Crafts reactions using a nonclassical carbonium ion.^8^ In turn, reagent-controlled approaches have been largely unsuccessful. Initial attempts in this field trace back to Fry, who employed enantioenriched silicon-stereogenic hydrosilanes as chiral hydride donors to asymmetrically reduce carbocations.^9^ Our laboratory seized this idea and combined it with our continued interest in cyclohexadiene-based transfer reagents^10^ to develop a reagent-controlled asymmetric transfer hydrogenation.^11^ We recently succeeded in utilizing asymmetrically substituted cyclohexa-1,4-dienes as chiral dihydrogen surrogates in good yields and with promising enantioselectivities (Scheme 1).

**Scheme 1:**
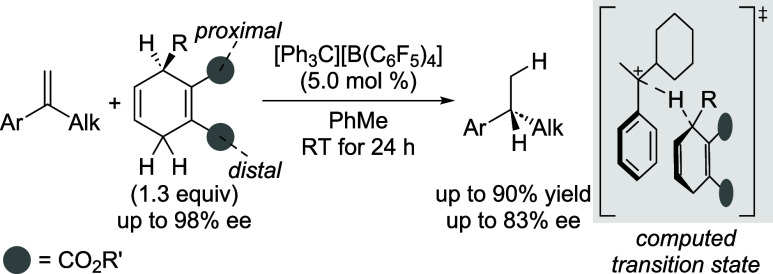
State-of-the-Art of Reagent-Controlled Transfer Hydrogenation Using Chiral Cyclohexa-1,4-dienes^a^

a R = alkyl. R′ = alkyl or aryl.

Computations by Qu and Grimme provided a deeper understanding of the mechanism where a hydride is enantioselectively transferred from the cyclohexadiene to the protonated alkene, i.e., the prochiral carbenium ion (gray box).^11^ These calculations have revealed that even though the proximal ester moiety (gray circle) is beneficial for preorganization of the two reactants, it is actually not a necessary functional group. The main factor for enantiofacial control is the steric bulk of that proximal group with no substitution of the other proximal position. The enantioselectivity of the hydride transfer was expected to improve with an increasing size of that substituent. The distal ester group (gray circle) did not seem to exert any significant effect. We therefore decided to verify both of these predictions experimentally.

One of the challenges of our initial study was the enantioselective synthesis of cyclohexa-1,4-dienes with an asymmetrically substituted tertiary (3°) carbon atom. Despite the availability of such synthetic transformations,^12,13^ none were suitable for our demands as certain substitution patterns are required to suppress undesirable side reactions.^14,15^ A method recently reported by Zheng^16^ then enabled the preparation of chiral cyclohexadienes with identical substituents in both the proximal and distal positions (Scheme 2, left). While we had been involved in initial work,^11^ RajanBabu and co-workers disclosed a more flexible methodology that gives access to a substantially greater variety of these cyclohexadiene-based dihydrogen surrogates (Scheme 2, right).^17^ In our hands, RajanBabu’s protocol was not without problems. The purification of the title compounds proved to be tedious, mainly due to the similar polarities of several isomers and other byproducts. The enantiomeric excesses of the obtained cyclohexa-1,4-dienes were ranging from 90 to 96% in most cases (Figure 1). It ought to be mentioned that a phenyl group was tolerated neither at the tertiary (3°) carbon atom nor in the distal position.

**Figure 1:**
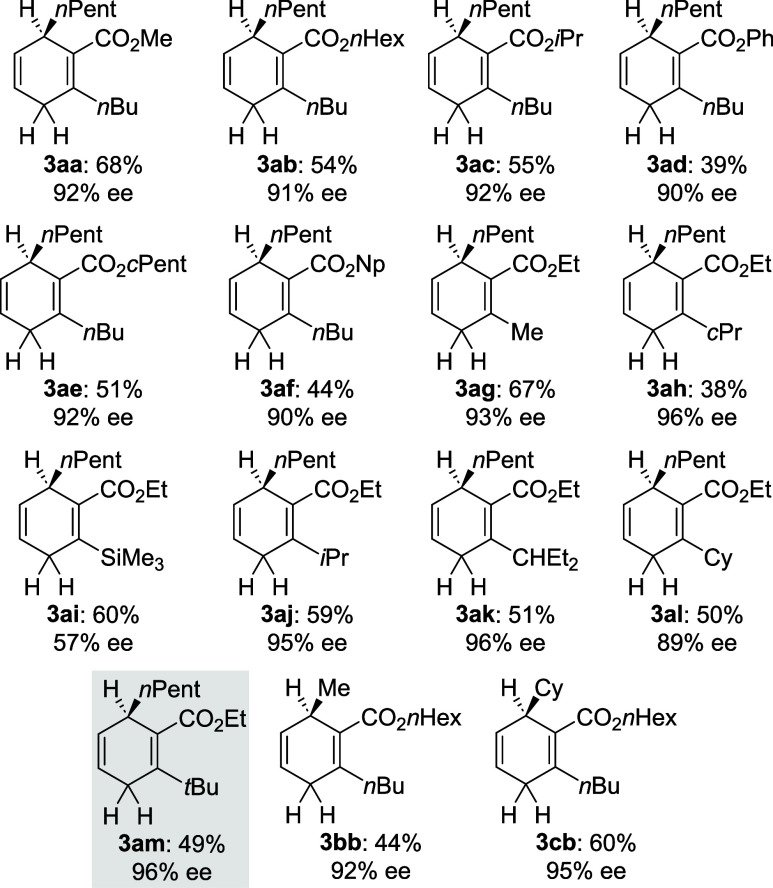
Chiral cyclohexa-1,4-dienes prepared by RajanBabu’s method. Enantiomeric excesses were determined by GLC or HPLC analysis on chiral stationary phases. Purification was routinely performed by flash column chromatography on silica gel and subsequent automatic reversed-phase flash column chromatography (see the Supporting Information for details). Np = neopentyl.

**Scheme 2:**
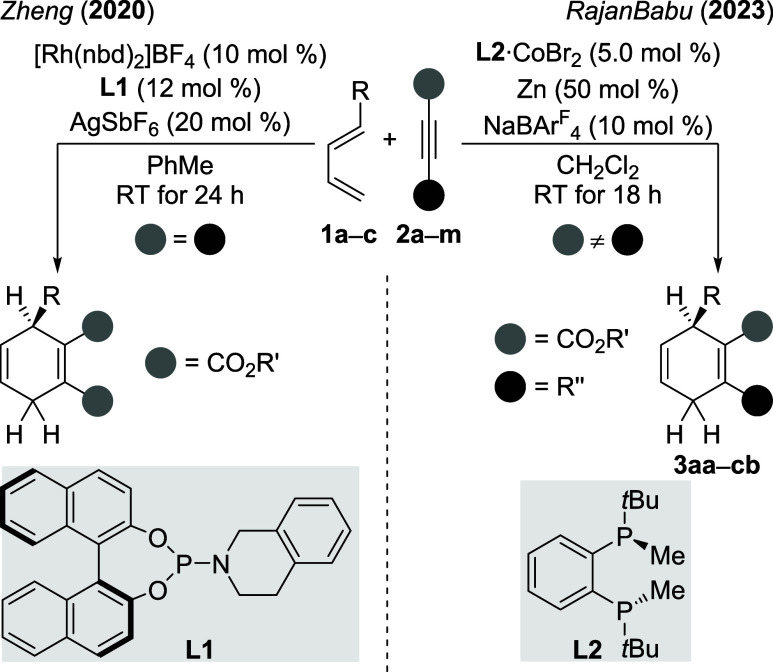
Used Methods for Chiral Cyclohexa-1,4-diene Synthesis^a^

a R = alkyl. R′ = alkyl or aryl. R″ = alkyl.

We subsequently tested the dihydrogen surrogates **3aa**–**cb** under the reported, optimized reaction conditions of our earlier model reaction of 1,1-disubstituted alkene **4a** to afford product **5a** ^11^ (Table 1). As the cyclohexadienes **3aa**–**cb** were not enantiomerically pure, we added the enantiospecificity (e.s.) of the reaction to the table. We first varied the R′ group of the proximal ester while keeping the distal substituent an *n*-butyl group (**3aa**–**af**, entries 1–6). There was no noticeable effect on either reactivity or selectivity; the only exception was phenylester **3ad** furnishing a slightly diminished yield (entry 4). Next, we investigated the effect of different alkyl-substituents in the distal position, now keeping the ester group the same (**3ag**–**am**, entries 7–13). We observed that groups in this position with increasing steric demand show a small but measurable positive effect on the enantioinduction. The lowest is found with a cyclopropyl group (**3ah**, entry 8) and the best is obtained with a *tert*-butyl group (**3am**, entry 13). The enantiospecificity of 83% compared with that obtained in our lead study, where both substituents were CO_2_ *n*Hex groups.^11^ In turn, the Me_3_Si-substituted surrogate did not bring about any improvement (**3ai**, entry 9), and the pent-3-yl group is an outlier (**3ak**, entry 11). The yields do not follow any obvious pattern and are ranging from 67 to 95%. Changing the R group at the asymmetrically substituted carbon atom from *n*-pentyl in **3ab** (entry 2) to methyl in **3bb** (entry 14) and cyclohexyl in **3cb** (entry 15) did lead to lower and almost the same enantioinduction, respectively.

**Table 1:**
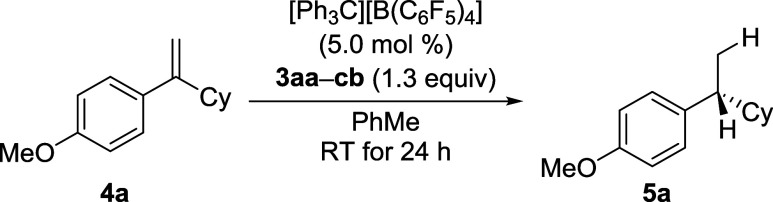
Surrogate Screening

entry	surrogate **3** (ee)	R	proximal substituent CO_2_R′	distal substituent R″	yield (%)^a^	ee (%)^b^	e.s. (%)^c^
1	**3aa** (92)	*n*Pent	CO_2_Me	*n*Bu	89	69	75
2	**3ab** (91)	*n*Pent	CO_2_ *n*Hex	*n*Bu	92	68	75
3	**3ac** (92)	*n*Pent	CO_2_ *i*Pr	*n*Bu	93	69	75
4	**3ad** (90)	*n*Pent	CO_2_Ph	*n*Bu	73	69	77
5	**3ae** (92)	*n*Pent	CO_2_ *c*Pent	*n*Bu	98	67	73
6	**3af** (90)	*n*Pent	CO_2_Np	*n*Bu	95	70	78
7	**3ag** (93)	*n*Pent	CO_2_Et	Me	95	64	69
8	**3ah** (96)	*n*Pent	CO_2_Et	*c*Pr	67	58	60
9	**3ai** (57)	*n*Pent	CO_2_Et	SiMe_3_	82	40	70
10	**3aj** (95)	*n*Pent	CO_2_Et	*i*Pr	94	71	75
11	**3ak** (96)	*n*Pent	CO_2_Et	CHEt_2_	72	66	69
12	**3al** (89)	*n*Pent	CO_2_Et	Cy	74	69	78
13	**3am** (96)	*n*Pent	CO_2_Et	*t*Bu	95	80	83
14	**3bb** (92)	Me	CO_2_ *n*Hex	*n*Bu	95	56	61
15	**3cb** (95)	Cy	CO_2_ *n*Hex	*n*Bu	75	68	72

a Determined by GLC analysis with *n*-dodecane as an internal standard.

b Determined by GLC analysis on a chiral stationary phase. The absolute configuration was assigned by comparison with reported data.^11^

c The enantiospecificity was estimated by the following formula: e.s. = ee­(product **5**)/ee­(surrogate **3**)·100.

Lastly, a representative substrate scope was tested with the *best* surrogate **3am** (Scheme 3). The α-substituted styrene derivatives were selected on the basis of our earlier study^11^ to allow for comparability; enantiospecificity values from our previous report are given in parentheses.^11^ While the model substrate **4a** had given very similar results for both the dihydrogen surrogate **3am** and the previously reported cyclohexa-1,4-diene (gray box), the outcomes were quite different for **4b** (superior for cycloheptyl instead of cyclohexyl) and **4c** (inferior neopentyl instead of cyclohexyl). No difference was seen for functionalized derivative **4d**, but a benzyl group as in **4e** was again less compatible. In turn, when replacing the 4-anisyl by a phenyl group as in **4f**, both chiral cyclohexa-1,4-dienes performed equally well.

**Scheme 3:**
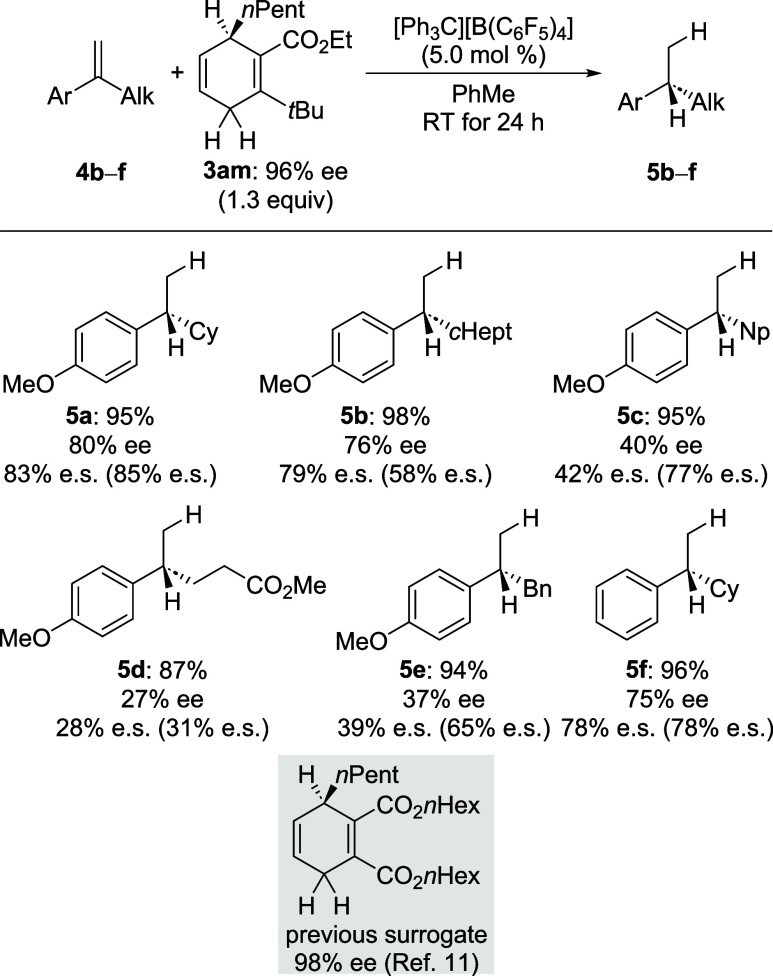
Representative Substrate Scope^a^

a Yields were determined by ^1^H NMR spectroscopy using 1,4-dinitrobenzene as an internal standard. Enantiomeric excesses were determined by HPLC analysis on chiral stationary phases. The ee value in parentheses is the result with the previously used surrogate.^11^ Np = neopentyl.

In summary, this study was meant to further substantiate the computational predictions made in our earlier investigation.^11^ We prepared a larger set of chiral cyclohexa-1,4-dienes **3aa**–**3cb** with variation of the R′ group in the proximal ester (gray circle) and replacement of the distal ester group by an alkyl/silyl group R″ (black circle; eight examples). It was that group that had a rather weak effect on enantioinduction for a given alkene substrate, eventually arriving at 83% e.s. for R″ = *tert*-butyl in **3am**. This did, however, not lead to a generally improved chiral dihydrogen surrogate, emphasizing that the design of a reliable reagent-controlled transfer hydrogenation remains a challenge.

## Supplementary Material



## Data Availability

The data underlying this study are available in the published article and its Supporting Information.

## Notes

General procedures, experimental details, and characterization and spectral data of all new compounds (PDF)
