# Supplementary material for: Analysis of Cyclohexa-1,4-dienes with an Asymmetrically Substituted, 3° Carbon Atom as Chiral Dihydrogen Surrogates in Reagent-Controlled Transfer Hydrogenation of Alkenes
Source: J Org Chem. 2025 Oct 2;90(41):14856–9. doi: 10.1021/acs.joc.5c02009 (PMC12538592; doi:10.1021/acs.joc.5c02009)
Supplement: Supplementary file 1 [file jo5c02009_si_001.pdf]

# Analysis of Cyclohexa-1,4-dienes with an Asymmetrically Substituted, 3° Carbon Atom as Chiral Dihydrogen Surrogates in Reagent-Controlled Transfer Hydrogenation of Alkenes

Paul E. Rucker and Martin Oestreich

*Institut für Chemie, Technische Universität Berlin*

*Straße des 17. Juni 115, 10623 Berlin, Germany*

*martin.oestreich@tu-berlin.de*

# Supporting Information

## Table of Contents

1 General Information S2
2 Experimental Details for the Synthesis of Ketones S3
2.1 General Procedure for the Synthesis of Ketones (**GP 1**) S3
2.2 Preparation and Characterization of the Ketones S3
3 Experimental Details for the Synthesis of Alkenes S6
3.1 General Procedure for the Synthesis of Alkenes (**GP 2**) S6
3.2 Preparation and Characterization of the Alkenes S6
4 Experimental Details for the Synthesis of (*E*)-1,3-Dienes S10
4.1 Preparation and Characterization of the Dienes S10
5 Experimental Details for the Synthesis of Alkynes S12
5.2 General Procedures for the Synthesis of Alkynes (**GP 3–6**) S12
5.3 Preparation and Characterization of Alkynes S13
6 Experimental Details for the Synthesis of $H_2$-Surrogates S22
6.1 General Procedure for the Synthesis of $H_2$-Surrogates (**GP 7**) S22
6.2 Preparation and Characterization of $H_2$-Surrogates S22
7 Asymmetric Transfer Hydrogenation of Styrene Derivatives S35
7.1 General Procedure for the Asymmetric Transfer Hydrogenation of Styrene Derivatives (**GP 8**) S35
7.2 Characterization Data of the Enantioenriched Hydrogenation Products S35
8 GLC and HPLC Traces S40
8.1 GLC Traces S40
8.2 HPLC Traces S43
9 NMR Spectra S61
10 References S159

# 1 General Information

Unless otherwise stated, all reactions were performed in flame-dried glassware under an atmosphere of argon using an *MBraun* glovebox ($O_2$ < 0.5 ppm, $H_2O$ < 0.5 ppm) or by applying conventional Schlenk techniques under a static pressure of nitrogen (fume hood) unless otherwise stated. Standard solvents and reagents were obtained from commercial suppliers and used as received, unless otherwise stated. Technical grade solvents for extraction and chromatography were distilled prior to use. Dichloromethane and tetrahydrofurane were dried over calcium hydride and sodium, respectively, and freshly distilled prior to use. Toluene was dried over sodium, distilled, degassed by three freeze-pump-thaw cycles, and stored in a glovebox over thermally activated 4 Å molecular sieves. The used catalysts [Co((*R*,*R*)-BenzP*)$Br_2$] (**L2**·$CoBr_2$) and [Co(DPPP)$Br_2$] were synthesized according to a reported procedure.[1] Analytical thin-layer chromatography (TLC) was performed on ALUGRAM® Xtra Sil G/UV254 TLC-Sheets by *Macherey-Nagel*. Flash column chromatography (FCC) was performed on silica gel 60 (40–63 µm, 230–400 mesh ASTM) by *VWR Chemicals* using the indicated solvents. Automatic column chromatography was performed using *Biotage* Isolera 1 and *Interchim* PF-15C18AQ-F0040 as stationary phase. $^1$H, $^{13}$C, $^{19}$F and $^{29}$Si NMR spectra were recorded in $CDCl_3$ on *Bruker* AV400 and *Bruker* AV500 instruments. Chemical shifts are reported in parts per million (ppm) and are referenced to the residual solvent resonance as the internal standard ($CHCl_3$: δ = 7.26 ppm for $^1$H NMR and $CDCl_3$: δ = 77.16 ppm for $^{13}$C NMR). Data are reported as follows: chemical shift, multiplicity (s = singlet, d = doublet, t = triplet, q = quartet, quint = quintet, sext = sextet, sept = septet, m = multiplet, $m_c$ = centrosymmetric multiplet, br = broad, p = pseudo and combinations thereof), coupling constants (Hz), and integration. Analytical gas liquid chromatography (GLC) was performed on an *Agilent Technologies* GC 8860 gas chromatograph equipped with an *Agilent Technologies* J&W HP-5 capillary column (30 m × 0.32 mm, 0.25 µm film thickness) using the following program: $N_2$ carrier gas, injection temperature 250 °C, detector temperature 275 °C, flow rate isobaric 11 psi; temperature program: start temperature 40 °C, heating rate 20 °C/min, end temperature 250 °C for 10 min, unless otherwise stated. Analytical high performance liquid chromatography (HPLC) was performed on an *Agilent Technologies* 1200 or 1200 infinity instruments with the indicated chiral stationary phase from *Daicel*, using *n*-heptane:isopropanol mixtures as the mobile phase. Infrared (IR) spectra were recorded on an *Agilent Technologies* Cary 630 FT-IR spectrometer equipped with a diamond ATR unit and selected signals are reported in wavenumbers ($cm^{-1}$). High resolution mass spectra (HRMS) were obtained from the *Laboratory of Mass Spectrometry* at the *Institut für Chemie*, *Technische Universität Berlin*. Optical rotations were measured on a *Schmidt & Haensch* Polatronic H532 polarimeter. $[\alpha]_\lambda^T$ values are reported in $10^{-1}$ (° $cm^2$ $g^{-1}$); with the concentration g/100 mL, the Temperature T, and the wavelength λ indicated.

# 2 Experimental Details for the Synthesis of Ketones

## 2.1 General Procedure for the Synthesis of Ketones (GP 1)

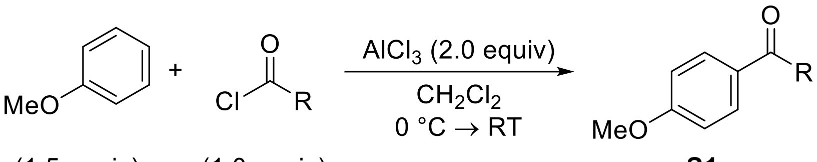


According to a literature procedure,[2] anisole (1.5 equiv) is dissolved in $CH_2Cl_2$ (3 mL/mmol) followed by addition of $AlCl_3$ (2.0 equiv) and the acyl chlorides (1.0 equiv) at 0 °C. The mixture is stirred for 5 min, then allowed to warm up to RT and stirred for 20 h. The reaction is then quenched with ice water, the layers are separated and the aqueous layer is extracted using $CH_2Cl_2$ (3 x). The combined organic layers washed with saturated aqueous $Na_2CO_3$ solution and dried over $MgSO_4$. All volatiles were removed under reduced pressure, yielding the products **S1a–e** in analytically pure form.

## 2.2 Preparation and Characterization of the Ketones

### 2.2.1 Cyclohexyl(4-methoxyphenyl)methanone (S1a)

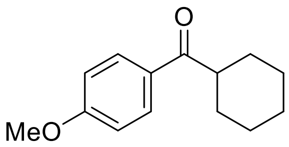


**S1a**
$C_{14}H_{18}O_2$
M = 218.30 g/mol

Prepared from anisole (3.00 g, 27.7 mmol, 1.50 equiv), cyclohexanecarbonyl chloride (2.71 g, 2.50 mL, 18.5 mmol, 1.00 equiv) and $AlCl_3$ (4.93 g, 37.0 mmol, 2.00 equiv) according to **GP 1**. Removal of all volatiles afforded the product **S1a** in analytically pure form (3.99 g, 18.3 mmol, 99%) as a colorless solid.

**$^1$H NMR** (400 MHz, $CDCl_3$, 298 K): δ/ppm = 7.94 ($m_c$, 2H), 6.93 ($m_c$, 2H), 3.87 (s, 3H), 3.22 (t, $J$ = 11.2 Hz, 1H), 1.89–1.82 (m, 4H), 1.75–1.72 (m, 1H), 1.54–1.25 (m, 5H). **$^{13}$C{$^1$H} NMR** (100 MHz, $CDCl_3$, 298 K): δ/ppm = 202.6, 163.4, 130.6 (2C), 129.4, 113.8 (2C), 55.6, 45.5, 29.7 (2C), 26.1, 26.1 (2C).

The NMR spectroscopic data are in accordance with those reported.[2]

### 2.2.2 Cycloheptyl(4-methoxyphenyl)methanone (S1b)

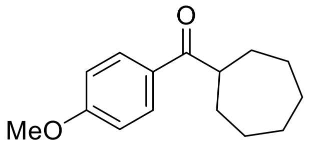


**S1b**
$C_{15}H_{20}O_2$
M = 232.32 g/mol

Cycloheptanecarboxylic acid (569 mg, 4.00 mmol, 1.00 equiv) was dissolved in thionyl chloride (2.4 g, 1.5 mL, 20 mmol, 5.0 equiv) and stirred until gas evolution ceased. It is then heated to 50 °C for 1 h before excess thionyl chloride is removed under reduced pressure. This affords cycloheptancarbonyl chloride which is used for the next step without further purification. The final product is then prepared from anisole (324 mg, 3.00 mmol, 1.50 equiv), cycloheptanecarbonyl chloride (321 mg, 2.00 mmol, 1.00 equiv) and $AlCl_3$ (533 mg, 4.00 mmol, 2.00 equiv) according to **GP 1**. Removal of all volatiles afforded the product **S1b** in analytically pure form (430 mg, 1.85 mmol, 93%) as a colorless oil.

**$^1$H NMR** (400 MHz, $CDCl_3$, 298 K): δ/ppm = 7.92 ($m_c$, 2H), 6.93 ($m_c$, 2H), 3.87 (s, 3H), 3.38 ($m_c$, 1H), 1.94–1.87 (m, 2H), 1.83–1.76 (m, 2H), 1.74–1.50 (m, 8H). **$^{13}$C{$^1$H} NMR** (100 MHz, $CDCl_3$, 298 K): δ/ppm = 203.1, 163.3, 130.7 (2C), 129.4, 113.8 (2C), 55.6, 46.5, 31.1 (2C), 28.5 (2C), 27.0 (2C).

The NMR spectroscopic data are in accordance with those reported.[2]

### 2.2.3 1-(4-Methoxyphenyl)-3,3-dimethylbutan-1-one (S1c)

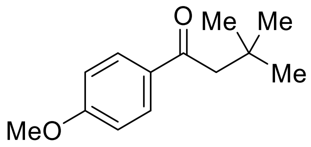


**S1c**
$C_{13}H_{18}O_2$
M = 206.29 g/mol

Prepared from anisole (324 mg, 3.00 mmol, 1.50 equiv), 3,3-dimethylbutanoyl chloride (269 mg, 2.00 mmol, 1.00 equiv) and $AlCl_3$ (533 mg, 4.00 mmol, 2.00 equiv) according to **GP 1**. Removal of all volatiles afforded the product **S1c** in analytically pure form (358 mg, 1.74 mmol, 87%) as a slightly yellow solid.

**$^1$H NMR** (400 MHz, $CDCl_3$, 298 K): δ/ppm = 7.93 ($m_c$, 2H), 6.91 ($m_c$, 2H), 3.87 (s, 3H), 2.80 (s, 2H), 1.06 (s, 9H). **$^{13}$C{$^1$H} NMR** (100 MHz, $CDCl_3$, 298 K): δ/ppm = 199.1, 163.3, 131.8, 130.7 (2C), 113.7 (2C), 55.6, 49.9, 31.6, 30.3 (3C).

The NMR spectroscopic data are in accordance with those reported.[2]

### 2.2.4 Methyl 4-(4-methoxyphenyl)-4-oxobutanoate (S1d)

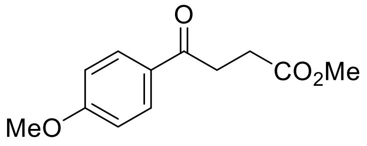


**S1d**
$C_{12}H_{14}O_4$
M = 222.24 g/mol

Prepared from anisole (324 mg, 3.00 mmol, 1.50 equiv), methyl succinyl chloride (301 mg, 2.00 mmol, 1.00 equiv) and $AlCl_3$ (533 mg, 4.00 mmol, 2.00 equiv) according to **GP 1**. Removal of all volatiles afforded the product **S1d** in analytically pure form (389 mg, 1.75 mmol, 88%) as a colorless solid.

**$^1$H NMR** (400 MHz, $CDCl_3$, 298 K): δ/ppm = 7.97 ($m_c$, 2H), 6.94 ($m_c$, 2H), 3.87 (s, 3H), 3.71 (s, 3H), 3.27 (t, $J$ = 6.7 Hz, 2H), 2.76 (t, $J$ = 6.7 Hz, 2H). **$^{13}$C{$^1$H} NMR** (100 MHz, $CDCl_3$, 298 K): δ/ppm = 196.7, 173.7, 163.7, 130.4 (2C), 129.8, 113.9 (2C), 55.6, 51.9, 33.2, 28.3.

The NMR spectroscopic data are in accordance with those reported.[2]

### 2.2.5 1-(4-Methoxyphenyl)-2-phenylethan-1-one (S1e)

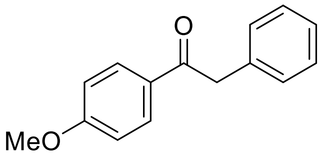


**S1e**
$C_{15}H_{14}O_2$
M = 226.27 g/mol

Prepared from anisole (324 mg, 3.00 mmol, 1.50 equiv), benzoyl chloride (309 mg, 2.00 mmol, 1.00 equiv) and $AlCl_3$ (533 mg, 4.00 mmol, 2.00 equiv) according to **GP 1**. Removal of all volatiles afforded the product **S1e** in analytically pure form (435 mg, 1.92 mmol, 96%) as a colorless solid.

**$^1$H NMR** (400 MHz, $CDCl_3$, 298 K): δ/ppm = 7.80 ($m_c$, 2H), 7.34–7.33 (m, 5H), 6.93 ($m_c$, 2H), 4.24 (s, 2H), 3.86 (s, 3H). **$^{13}$C{$^1$H} NMR** (100 MHz, $CDCl_3$, 298 K): δ/ppm = 196.4, 163.7, 135.1, 131.1 (2C), 129.8, 129.5 (2C), 128.8 (2C), 126.9, 113.9 (2C), 55.6, 45.4.

The NMR spectroscopic data are in accordance with those reported.[2]

# 3 Experimental Details for the Synthesis of Alkenes

## 3.1 General Procedure for the Synthesis of Alkenes (GP 2)

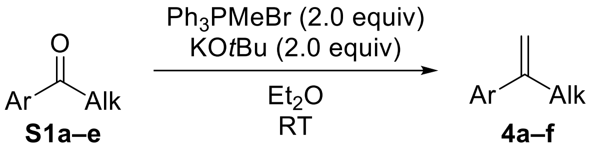


According to a modified literature procedure,[2] $Ph_3PMeBr$ (2.0 equiv) is suspended in THF (3 mL/mmol) and KO*t*Bu (1.95 equiv) is added. After stirring for 1 h at RT, the ketones **S1a–e** (1.00 equiv) are added and the mixture is stirred for 18 h. The suspension is then diluted with *n*-pentane, filtered through a plug of silica and all volatiles are removed under reduced pressure. If necessary, the crude product is purified by FCC on silica gel using *n*-pentane:$Et_2O$ or *n*-pentane:$CH_2Cl_2$ mixtures as eluent to afford the alkenes **4a–f**.

## 3.2 Preparation and Characterization of the Alkenes

### 3.2.1 1-(1-Cyclohexylvinyl)-4-methoxybenzene (4a)

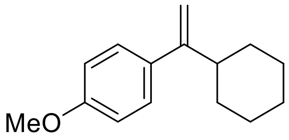


**4a**
$C_{15}H_{20}O$
M = 216.32 g/mol

Prepared from ketone **S1a** (5.46 mg, 25.0 mmol, 1.00 equiv), $Ph_3PMeBr$ (17.9 g, 50.0 mmol, 2.00 equiv) and KO*t*Bu (5.47 g, 48.8 mmol, 1.95 equiv) according to **GP 2**. Removal of all volatiles afforded the product **4a** in analytically pure form (3.65 g, 16.8 mmol, 67%) as a colorless oil.

**$^1$H NMR** (400 MHz, $CDCl_3$, 298 K): δ/ppm = 7.37 ($m_c$, 2H), 6.89 ($m_c$, 2H), 5.33 (d, $J$ = 1.3 Hz, 1H), 5.11 (t, $J$ = 1.3 Hz, 1H), 3.44 (s, 3H), 2.49 ($m_c$, 1H), 2.05–1.98 (m, 2H), 1.83–1.78 (m, 2H), 1.76–1.70 (m, 1H), 1.40–1.30 (m, 2H), 1.27–1.16 (m, 3H). **$^{13}$C{$^1$H} NMR** (100 MHz, $CDCl_3$, 298 K): δ/ppm = 158.9, 154.5, 135.5, 127.7 (2C), 113.6 (2C), 109.2, 55.4, 42.7, 32.9 (2C), 27.0 (2C), 26.6.

The NMR spectroscopic data are in accordance with those reported.[2]

### 3.2.2 (1-(4-Methoxyphenyl)vinyl)cycloheptane (4b)

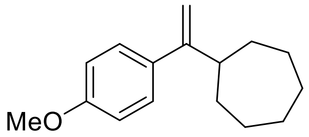

**4b**
$C_{16}H_{22}O$
M = 230.35 g/mol

Prepared from ketone **S1b** (279 mg, 1.20 mmol, 1.00 equiv), $Ph_3PMeBr$ (857 mg, 2.40 mmol, 2.00 equiv) and KO*t*Bu (263 mg, 2.34 mmol, 1.95 equiv) according to **GP 2**. Removal of all volatiles afforded the product **4b** in analytically pure form (226 mg, 981 µmol, 82%) as a colorless oil.

**$^1$H NMR** (400 MHz, $CDCl_3$): δ/ppm = 7.30 ($m_c$, 2H), 6.86 ($m_c$, 2H), 5.08–5.07 (m, 1H), 4.96–4.95 (m, 1H), 3.81 (s, 3H), 2.60–2.55 (m, 1H), 1.91–1.85 (m, 2H), 1.77–1.41 (m, 10H). **$^{13}$C{$^1$H} NMR** (100 MHz, $CDCl_3$, 298 K): δ/ppm = 158.9, 155.7, 135.7, 127.7 (2C), 113.6 (2C), 109.2, 55.4, 44.7, 34.9 (2C), 28.1 (2C), 27.1 (2C).

The NMR spectroscopic data are in accordance with those reported.[2]

### 3.2.3 (1-(4,4-Dimethylpent-1-en-2-yl)-4-methoxybenzene (4c)

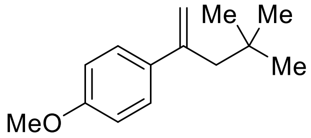

**4c**
$C_{14}H_{20}O$
M = 204.31 g/mol

Prepared from ketone **S1c** (248 mg, 1.20 mmol, 1.00 equiv.), $Ph_3PMeBr$ (857 mg, 2.40 mmol, 2.00 equiv) and KO*t*Bu (263 mg, 2.34 mmol, 1.95 equiv.) according to **GP 2**. The crude product was purified by FCC on silica gel using *n*-pentane:$CH_2Cl_2$ (19:1) as eluent to afford the product **4c** (170 mg, 832 µmol, 69%) as a colorless oil.

**$^1$H NMR** (400 MHz, $CDCl_3$, 298 K): δ/ppm = 7.31 ($m_c$, 2H), 6.83 ($m_c$, 2H), 5.19–5.18 (m, 1H), 4.93–4.92 (m, 1H), 3.81 (s, 3H), 2.43 (s, 2H), 0.80 (s, 9H). **$^{13}$C{$^1$H} NMR** (100 MHz, $CDCl_3$, 298 K): δ/ppm = 158.9, 147.0, 136.3, 127.7 (2C), 115.0, 115.6 (2C), 55.4, 49.0, 31.8, 30.2 (3C).

The NMR spectroscopic data are in accordance with those reported.[2]

### 3.2.4 Methyl 4-(4-methoxyphenyl)pent-4-enoate (4d)

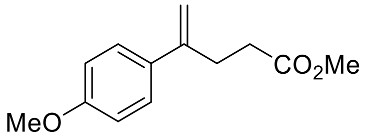


**4d**
$C_{13}H_{16}O_3$
M = 220.27 g/mol

Prepared from ketone **S1d** (267 mg, 1.20 mmol, 1.00 equiv), $Ph_3PMeBr$ (857 mg, 2.40 mmol, 2.00 equiv) and KO*t*Bu (263 mg, 2.34 mmol, 1.95 equiv) according to **GP 2**. The crude product was purified by FCC on silica gel using *n*-pentane:$Et_2O$ (9:1) as eluent to afford the product **4d** (153 mg, 694 µmol, 58%) as a colorless oil.

**$^1$H NMR** (400 MHz, $CDCl_3$, 298 K): δ/ppm = 7.35 ($m_c$, 2H), 6.87 ($m_c$, 2H), 5.23 (m, 1H), 5.00 (m, 1H), 3.81 (s, 3H), 3.66 (s, 3H), 2.81 (t, *J* = 7.8 Hz, 2H), 2.48 (t, *J* = 7.8 Hz, 2H). **$^{13}$C{$^1$H} NMR** (100 MHz, $CDCl_3$, 298 K): δ/ppm = 173.8, 159.3, 146.3, 133.0, 127.3 (2C), 113.9 (2C), 111.4, 55.4, 51.7, 33.3, 30.6.

The NMR spectroscopic data are in accordance with those reported.[2]

### 3.2.5 1-Methoxy-4-(3-phenylprop-1-en-2-yl)benzene (4e)

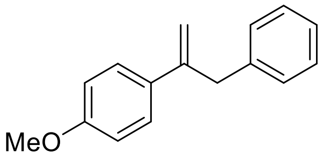


**4e**
$C_{16}H_{16}O$
M = 224.30 g/mol

Prepared from ketone **S1e** (272 mg, 1.20 mmol, 1.00 equiv), $Ph_3PMeBr$ (857 mg, 2.40 mmol, 2.00 equiv) and KO*t*Bu (263 mg, 2.34 mmol, 1.95 equiv) according to **GP 2**. The crude product was purified by FCC on silica gel using *n*-pentane:$CH_2Cl_2$ (9:1) as eluent to afford the product **4e** (113 mg, 504 µmol, 42%) as a colorless oil.

**$^1$H NMR** (400 MHz, $CDCl_3$, 298 K): δ/ppm 7.38 ($m_c$, 2H), 7.28–7.17 (m, 5H), 6.81 ($m_c$, 2H), 5.43–5.42 (m, 1H), 4.95–4.94 (m, 1H), 3.81 (brs, 2H), 3.78 (s, 3H). **$^{13}$C{$^1$H} NMR** (100 MHz, $CDCl_3$, 298 K): δ/ppm = 159.2, 146.2, 139.8, 133.3, 129.0 (2C), 128.5 (2C), 127.4 (2C), 126.2, 113.7 (2C), 113.1, 55.4, 41.8.

The NMR spectroscopic data are in accordance with those reported.[2]

### 3.2.6 (1-Cyclohexylvinyl)benzene (4f)

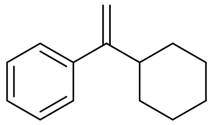

**4f**
$C_{14}H_{18}$
M = 186.30 g/mol

Prepared from phenylcyclohexylketone (226 mg, 1.20 mmol, 1.00 equiv), $Ph_3PMeBr$ (857 mg, 2.40 mmol, 2.00 equiv) and KO*t*Bu (263 mg, 2.34 mmol, 1.95 equiv) according to **GP 2**. Removal of all volatiles afforded the product **4f** in analytically pure form (162 mg, 870 µmol, 72%) as a colorless oil.

**$^1$H NMR** (400 MHz, $CDCl_3$, 298 K): δ/ppm = 7.35–7.23 (m, 5H), 5.14–5.13 (m, 1H), 5.02–5.01 (m, 1H), 2.42 ($m_c$, 1H), 1.85–1.70 (m, 5H), 1.37–1.12 (m, 5H). **$^{13}$C{$^1$H} NMR** (100 MHz, $CDCl_3$, 298 K): δ/ppm = 155.2, 143.1, 128.2 (2C), 127.1, 126.8 (2C), 110.5, 42.7, 32.9 (2C), 27.0 (2C), 26.6.

The NMR spectroscopic data are in accordance with those reported.[2]

# 4 Experimental Details for the Synthesis of (*E*)-1,3-Dienes

## 4.1 Preparation and Characterization of the Dienes

### 4.1.1 (*E*)-Nona-1,3-diene (1a)

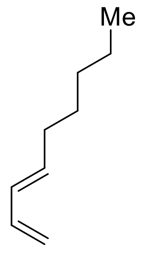

**1a**
$C_9H_{16}$
M = 124.23 g/mol

According to a modified literature procedure,[2] to a solution of $Ph_3PMeBr$ (7.14 g, 20.0 mmol, 2.00 equiv) in THF (50 mL) is added KO*t*Bu (2.19 g, 19.5 mmol, 1.95 equiv). The mixture is stirred at room temperature for 1 h and (*E*)-oct-2-enal (1.51 mL, 10.0 mmol, 1.00 equiv) is added. The mixture is stirred for 18 h, concentrated to about 10% of its volume and diluted with cyclohexane. Filtration through a plug of silica and subsequent removal of all volatiles yields the title compound **1a** in analytically pure (1.19 g, 9.58 mmol, 96%) form as a colorless liquid.

**$^1$H NMR** (400 MHz, $CDCl_3$, 298 K): δ/ppm = 6.34–6.27 (m, 1H), 6.07–6.02 (m, 1H), 5.74–5.68 (m, 1H), 5.08 (d, *J* = 16.5 Hz, 1H), 4.95 (d, *J* = 10.1 Hz, 1H), 2.08 (q, *J* = 7.2 Hz, 2H), 1.43–1.36 (m, 2H), 1.34–1.25 (m, 4H), 0.89 (t, *J* = 7.2 Hz, 3H). **$^{13}$C{$^1$H} NMR** (100 MHz, $CDCl_3$, 298 K): δ/ppm = 137.5, 135.8, 131.0, 114.7, 32.7, 31.6, 29.0, 22.7, 14.2.

The NMR spectroscopic data are in accordance with those reported.[2]

### 4.1.2 (*E*)-Buta-1,3-dien-1-ylcyclohexane (1c)

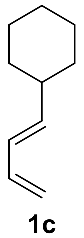

**1c**
$C_{10}H_{16}$
M =136.24 g/mol

According to a modified literature procedure,[3] a solution of diethyl allyl phosphonate (1.7 g, 1.7 mL, 9.6 mmol, 1.2 equiv) in THF (4 mL) was cooled to –78 °C and *n*BuLi (3.8 mL, 9.6

mmol, 1.2 equiv, 2.5 M in hexanes) was added dropwise. The mixture was stirred at –78 °C for 1 h and then cyclohexanecarbaldehyde (0.90 g, 0.96 mL, 8.0 mmol, 1.0 equiv) and DMPU (1.0 g, 0.97 mL, 8.0 mmol, 1.0 equiv) were added sequentially. The mixture was stirred at –78 °C for 2 h and then for another 18 h while slowly warming up to RT. The reaction solution was then quenched with saturated aqueous $NH_4Cl$ solution, the phases were separated and the aqueous layer was extracted with MTBE (3 x). The combined organic extracts were washed with brine, dried over $MgSO_4$, filtered and concentrated under reduced pressure. The residue was taken up in *n*-pentane and filtered through a plug of silica. The crude product was purified by FCC on silica gel using *n*-pentane as eluent to afford the product **1c** (496 mg, 6.64 mmol, 46%) as a colorless oil.

**$R_f$** = 0.83 (*n*-pentane). **$^1$H NMR** (400 MHz, $CDCl_3$, 298 K): δ/ppm = 6.30 (dt, *J* = 17.0, 10.3 Hz, 1H), 6.01 (dd, *J* = 15.2, 10.3 Hz, 1H), 5.66 (dd, *J* = 15.4, 6.9 Hz, 1H), 5.10 (d, *J* = 16.9 Hz, 1H), 4.96 (d, *J* = 10.1 Hz, 1H), 2.04–1.97 (m, 1H), 1.74–1.63 (m, 5H), 1.33–1.05 (m, 5H). **$^{13}$C{$^1$H} NMR** (100 MHz, $CDCl_3$, 298 K): δ/ppm = 141.4, 137.8, 128.4, 114.8, 40.8, 32.9 (2C), 26.3, 26.1 (2C). **HRMS** (APCI): calculated for $C_{10}H_{17}^+$ [(M+H)$^+$]: 137.1325, found: 137.1326. **IR** (ATR): $\tilde{\nu}$/cm$^{-1}$ = 3085, 3035, 2999, 2921, 2850, 2662, 2616, 2043, 1959, 1790, 1650, 1602, 1446, 1349, 1286, 1259, 1215, 1153, 1112, 1000, 949, 968, 891, 840.

# 5 Experimental Details for the Synthesis of Alkynes

## 5.1 General Procedures for the Synthesis of Alkynes (GP 3–6)

### 5.1.1 General Procedure for the Synthesis of Alkynes in the Corresponding Alcohol (GP 3)

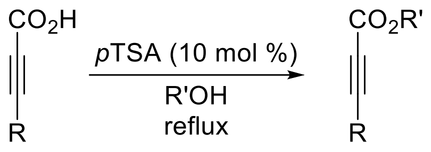


**2a–c, 2g, 2i**

Open to air, the acid (1.0 equiv) is dissolved in the corresponding alcohol (5.0 mL) and *p*TSA (10 mol %) is added. The mixture is refluxed for 24 h, filtered through a plug of silica and all volatiles are removed under reduced pressure yielding the product in analytically pure form.

### 5.1.2 General Procedure for the Synthesis of Alkynes in Toluene (GP 4)

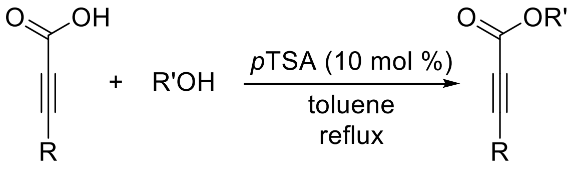


(5.0 equiv) **2e–f, 2n**

Open to air, 1-hexynoic acid (1.0 equiv), the corresponding alcohol (5.0 equiv) and *p*TSA (10 mol %) are dissolved in toluene (0.1 mmol/mL) and heated to reflux for 24 h using a Dean-Stark-apparatus. The mixture is concentrated to about 10% of its volume and then diluted with cyclohexane. Filtration through a plug of silica and subsequent removal of all volatiles yields the product in analytically pure form.

### 5.1.3 General Procedure for the Synthesis of Alkynes from Terminal Alkynes (GP 5)

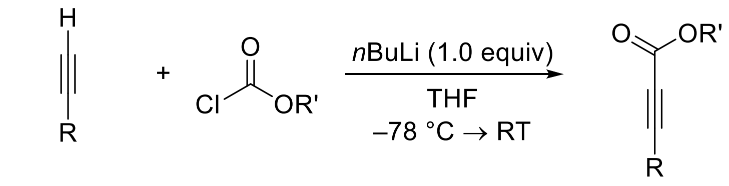


(1.1 equiv) (1.5 equiv) **2d, 2h, 2m**

A solution of the indicated alkyne (1.1 equiv) in THF (0.1 mmol/mL) is cooled to –78 °C and *n*BuLi (1.0 equiv) is added dropwise. The mixture is stirred for 1 h and the chloroformate (1.5 equiv) is added. The mixture is stirred for 18 h while slowly being allowed to warm up to room temperature. The solution is quenched with saturated aqueous $NH_4Cl$ solution (10 mL), extracted using MTBE (3 x), the combined organic layers washed with brine (20 mL) and dried

over $MgSO_4$. The crude product is either pure enough for further synthesis or purified by FCC if necessary.

### 5.1.4 General Procedure for the Synthesis of Alkynes from Aldehydes (GP 6)

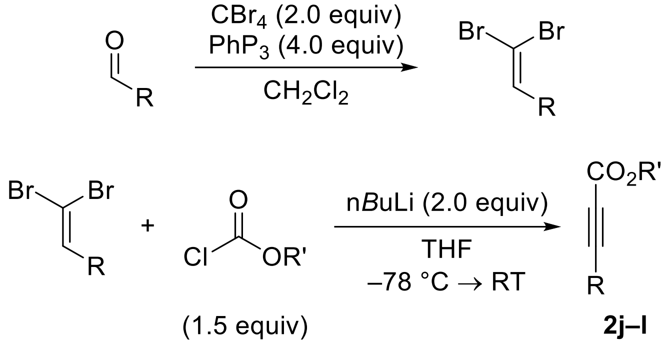

**2j–l**

According to a modified literature procedure,[4] $PhP_3$ (4.0 equiv), and $CBr_4$ (2.0 equiv) are dissolved in $CH_2Cl_2$ (6.7 mmol/mL with respect to the aldehyde) and stirred for 10 min. The solution is cooled to 0 °C and the aldehyde (1.0 equiv) is added and stirred for 1 h. All volatiles are removed under reduced pressure and the residue is dissolved in as little $CH_2Cl_2$ as possible and then diluted with cyclohexane. The suspension is filtered through a plug of silica, washed with *n*-pentane:$Et_2O$ = 95:5 and stripped of all volatiles. The dibromide is used for the next step without further purification.

The dibromide (1.0 equiv) is dissolved in THF (0.1 mmol/mL) and cooled to –78 °C. *n*BuLi (2.0 equiv) is added and the mixture is stirred for 5 min. The mixture is allowed to warm up to RT over 1 h and then cooled to –78 °C again. The chloroformate (1.5 equiv) is added and stirred overnight while slowly warming up to RT. The crude product is either pure enough for further synthesis or purified by FCC if necessary.

## 5.2 Preparation and Characterization of Alkynes

### 5.2.1 2-Heptynoic Acid (S2a)

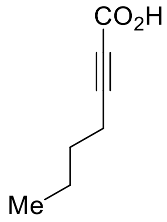

**S2a**
$C_7H_{10}O_2$
M = 126.16 g/mol

The compound **S2a** was obtained following a modified literature procedure.[5] Hexyne (2.0 g, 2.8 mL, 24 mmol, 1.1 equiv) was dissolved in THF (125 mL). The mixture was cooled to –78°C

and BuLi (8.9 mL, 22 mmol, 1.0 equiv, 2.5 M in hexanes) was added dropwise. The mixture was stirred for 1 h after which $CO_2$ generated from dry ice was bubbled through the solution for 30 min. The solution was then allowed to warm up to RT, aqueous HCl (2.0 M, 20 mL) was added and the phases were separated. The aqueous phase was extracted using $CH_2Cl_2$ (3 x 50 mL), the combined organic phases washed with brine and dried over $MgSO_4$. Removal of all volatiles yielded the product **S2a** (2.3 g, 18 mmol, 82%) as a lightly yellow oil.

**$^1$H NMR** (400 MHz, $CDCl_3$, 298 K): δ/ppm = 10.14 (s, 1H), 2.36 (t, *J* = 7.1 Hz, 2H), 1.61–1.55 (m. 2H), 1.48–1.40 (m, 2H), 0.93 (t, *J* = 7.3 Hz, 3H). **$^{13}$C{$^1$H} NMR** (100 MHz, $CDCl_3$, 298 K): δ/ppm = 158.2, 92.9, 72.7, 29.5, 22.1, 18.6, 13.6.

The NMR spectroscopic data are in accordance with those reported.[5]

### 5.2.2 3-(Trimethylsilyl)propiolic acid (S2b)

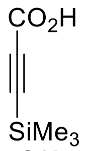


**S2b**

$C_6H_{10}O_2Si$

M = 142.23 g/mol

Propiolic acid (0.35 g, 0.31 mL, 5.0 mmol, 1.0 equiv) was dissolved in THF (50 mL) and cooled to 0 °C. MeMgBr (3.3 mL, 10 mmol, 2.0 equiv, 3 M in $Et_2O$) was added dropwise and stirred for 30 min followed by addition of TMSCl (0.65 mg, 0.76 mL, 6.0 mmol, 1.2 equiv). The mixture was stirred for 30 min and then allowed to warm up to RT. Aqueous HCl (1 M, 8 mL) was added and the product was extracted using EtOAc (3 x 20 mL). The combined organic phases were washed with brine and removal of all volatiles yielded the product **S2b** (578 mg, 4.06 mmol, 81%) as a colorless oil.

**$^1$H NMR** (400 MHz, $CDCl_3$, 298 K): δ/ppm = 9.84 (brs, 1H), 0.26 (s, 9H). **$^{13}$C{$^1$H} NMR** (100 MHz, $CDCl_3$, 298 K): δ/ppm = 157.5, 87.6, 93.8, –0.9 (3C).

The NMR spectroscopic data are in accordance with those reported.[6]

**5.2.3 Methyl-2-heptynoate (2a)**

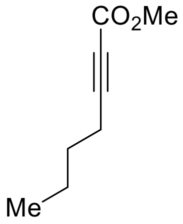


**2a**
$C_8H_{12}O_2$
M = 140.18 g/mol

Prepared from **S2a** (442 mg, 3.50 mmol, 1.00 equiv), *p*TSA (60 mg, 0.35 mmol, 10 mol %) and methanol (5.0 mL) according to **GP 3**. The product **2a** (332 mg, 2.37 mmol, 68%) was obtained as a colorless oil.

**$^1$H NMR** (400 MHz, $CDCl_3$, 298 K): δ/ppm = 3.75 (s, 3H), 2.33 (t, *J* = 7.1 Hz, 2H), 1.60–1.53 (m, 2H), 1.48–1.38 (m, 2H), 0.92 (t, *J* = 7.3 Hz, 3H). **$^{13}$C{$^1$H} NMR** (100 MHz, $CDCl_3$, 298 K): δ/ppm = 154.4, 90.1, 73.0, 52.7, 29.7, 22.1, 18.5, 13.6.

The NMR spectroscopic data are in accordance with those reported.[7]

**5.2.4 Hexyl-2-heptynoate (2b)**

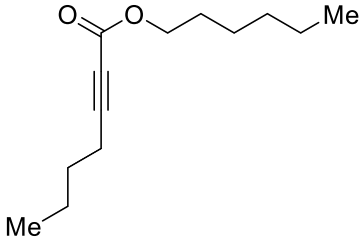


**2b**
$C_9H_{14}O_2$
M = 154.21 g/mol

Prepared from **S2a** (442 mg, 3.50 mmol, 1.00 equiv), *p*TSA (60 mg, 0.35 mmol, 10 mol %) and hexanol (5.0 mL) according to **GP 4**. The product **2b** (631 mg, 3.00 mmol, 86%) was obtained as a colorless oil.

**$^1$H NMR** (400 MHz, $CDCl_3$, 298 K): δ/ppm = 4.14 (t, *J* = 6.8 Hz, 2H), 2.33 (t, *J* = 7.1 Hz, 2H), 1.69–1.62 (m, 2H), 1.60–1.53 (m, 2H), 1.47–1.40 (m, 2H), 1.38–1.27 (m, 6H), 0.94–0.87 (m, 6H). **$^{13}$C{$^1$H} NMR** (100 MHz, $CDCl_3$, 298 K): δ/ppm = 154.2, 89.6, 73.3, 66.1, 31.5, 29.7, 28.5, 25.6, 22.7, 22.1, 18.5, 14.1, 13.6.

The NMR spectroscopic data are in accordance with those reported.[8]

### 5.2.5 Isopropyl-2-heptynoate (2c)

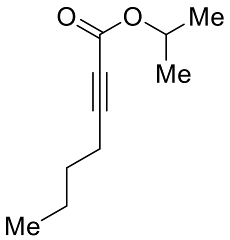


**2c**
$C_{10}H_{16}O_2$
M = 168.24 g/mol

Prepared from **S2a** (442 mg, 3.50 mmol, 1.00 equiv), *p*TSA (60 mg, 0.35 mmol, 10 mol %) and isopropanol (5.0 mL) according to **GP 3**. The product **2c** (346 mg, 2.06 mmol, 59%) was obtained as a colorless oil.

**$^1$H NMR** (500 MHz, $CDCl_3$, 298 K): δ/ppm = 5.07 (ps, *J* = 6.3 Hz, 1H), 2.32 (t, *J* = 7.2 Hz, 2H), 1.59–1.53 (m, 2H), 1.47–1.39 (m, 2H), 1.28 (d, *J* = 6.3 Hz, 6H), 0.92 (t, *J* = 7.3 Hz, 3H). **$^{13}$C{$^1$H} NMR** (100 MHz, $CDCl_3$, 298 K): δ/ppm = 153.6, 89.2, 73.6, 69.7, 29.7, 22.1, 21.8 (2C), 18.5, 13.6.

The NMR spectroscopic data are in accordance with those reported.[9]

### 5.2.6 Phenyl-2-heptynoate (2d)

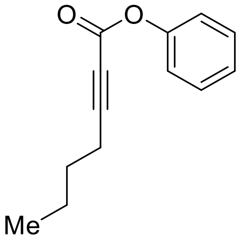


**2d**
$C_{13}H_{14}O_2$
M = 202.25 g/mol

Prepared from 1-hexyne (0.27 g, 0.38 mL, 3.3 mmol, 1.1 equiv), *n*BuLi (1.2 mL, 3.0 mmol, 1.0 equiv, 2.5 M in hexanes) and phenylchloroformate (0.71 g, 0.56 mL, 4.5 mmol, 1.5 equiv) according to **GP 5**. Purification by FCC on silica gel using cyclohexane:EtOAc (95:5) as eluent mixture afforded the product **2d** (600 mg, 2.97 mmol, 99%) as a yellow oil.

**$^1$H NMR** (400 MHz, $CDCl_3$, 298 K): δ/ppm = 7.40–7.37 (m, 2H), 7.25–7.23 (m, 1H), 7.14–7.12 (m, 2H), 2.40 (t, *J* = 7.1 Hz, 2H) 1.64–1.58 (m, 2H), 1.50–1.43 (m, 2H), 0.94 (t, *J* = 7.3 Hz, 3H). **$^{13}$C{$^1$H} NMR** (100 MHz, $CDCl_3$, 298 K): δ/ppm = 152.2, 150.3, 129.7 (2C), 126.4, 121.6 (2C), 92.5, 72.9, 29.6, 22.1, 18.7, 12.6.

The NMR spectroscopic data are in accordance with those reported.[10]

### 5.2.7 Cycopentyl-2-heptynoate (2e)

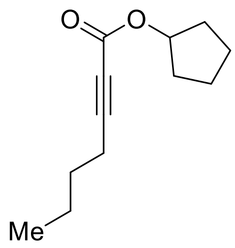


**2e**
$C_{12}H_{18}O_2$
M = 194.27 g/mol

Prepared from **S2a** (442 mg, 3.50 mmol, 1.00 equiv), *p*TSA (60 mg, 0.35 mmol, 10 mol %) and cyclopentanol (5.0 mL) according to **GP 4**. The product **2e** (456 mg, 2.35 mmol, 67%) was obtained as a colorless oil.

**$^1$H NMR** (400 MHz, $CDCl_3$, 298 K): δ/ppm = 5.24–5.19 (m, 1H), 2.32 (t, *J* = 7.1 Hz, 2H), 1.92–1.83 (m, 2H), 1.78–1.72 (m, 4H), 1.62–1.52 (m, 4H), 1.47–1.37 (m, 2H), 0.91 (t, *J* = 7.3 Hz, 3H). **$^{13}$C{$^1$H} NMR** (100 MHz, $CDCl_3$, 298 K): δ/ppm = 154.0, 89.1, 78.9, 73.6, 32.7 (2C), 29.7, 23.8 (2C), 22.1, 18.5, 13.6. **HRMS** (APCI) m/z: [M]$^+$ calculated for $C_{12}H_{19}O_2^+$ [(M+H)$^+$]: 195.1379; found: 195.1381. **IR** (ATR): $\tilde{\nu}$/cm$^{-1}$= 2958, 2871, 2233, 2123, 2064, 1701, 1456, 1360, 1321, 1245, 1164, 1072, 956, 900, 843, 752.

### 5.2.8 Neopentyl-2-heptynoate (2f)

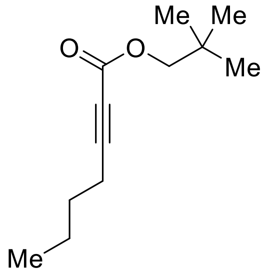


**2f**
$C_{12}H_{20}O_2$
M = 196.29 g/mol

Prepared from **S2a** (442 mg, 3.50 mmol, 1.00 equiv), *p*TSA (60 mg, 0.35 mmol, 10 mol %) and neopentanol (5.0 mL) according to **GP 4**. The product **2f** (231 mg, 1.18 mmol, 34%) was obtained as a colorless oil.

**$^1$H NMR** (400 MHz, $CDCl_3$, 298 K): δ/ppm = 3.85 (s, 2H), 2.34 (t, *J* = 7.1 Hz, 2H), 1.61–1.54 (m, 2H), 1.46–1.41 (m, 2H), 0.96 (s, 9H), 0.92 (t, *J* = 7.1 Hz, 3H). **$^{13}C\{^1H\}$ NMR** (100 MHz, $CDCl_3$, 298 K): δ/ppm = 154.4, 89.6, 75.0, 73.3, 31.5, 29.7, 26.5 (3C), 22.1, 18.6, 13.6. **HRMS** (APCI) m/z: $[M]^+$ calculated for $C_{12}H_{21}O_2^+$ $[(M+H)^+]$: 197.1356; found: 197.1358. **IR** (ATR): $\tilde{\nu}/cm^{-1}$= 3419, 2973, 2934, 2905, 2220, 2127, 2000, 1888, 1706, 1459, 1365, 1271, 1218, 1094, 1032, 931, 849, 792.

### 5.2.9 Ethyl but-2-ynoate (2g)

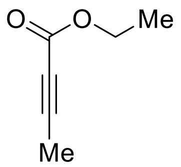


**2g**

$C_6H_8O_2$

M = 112.83 g/mol

Prepared from butynoic acid (442 mg, 3.50 mmol, 1.00 equiv), *p*TSA (60 mg, 0.35 mmol, 10 mol %) and ethanol (5.0 mL) according to **GP 3**. The product **2g** (437 mg, 2.83 mmol, 81%) was obtained as a colorless oil.

**$^1$H NMR** (400 MHz, $CDCl_3$, 298 K): δ/ppm = 4.21 (q, *J* = 7.1 Hz, 2H), 1.98 (s, 3H), 1.30 (t, *J* = 7.1 Hz, 3H). **$^{13}C\{^1H\}$ NMR** (100 MHz, $CDCl_3$, 298 K): δ/ppm = 153.9, 85.4, 72.6, 61.9, 14.2, 3.9.

The NMR spectroscopic data are in accordance with those reported.[11]

### 5.2.10 Ethyl 3-cyclopropylpropiolate (2h)

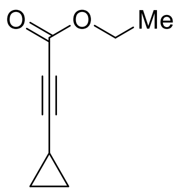


**2h**

$C_8H_{10}O_2$

M = 138.17 g/mol

Prepared from cyclopropylacetylene (0.29 g, 0.37 mL, 4.4 mmol, 1.1 equiv), *n*BuLi (1.5 mL, 4.0 mmol, 1.0 equiv, 2.7 M in hexanes) and ethylchloroformate (0.65 g, 0.57 mL, 6.0 mmol, 1.5 equiv) according to **GP 5**. Purification by FCC on silica gel using *n*-pentane:MTBE (98:2 → 96:4) as eluent afforded the product **2h** (459 mg, 3.32 mmol, 83%) as a colorless oil.

**$^{1}$H NMR** (400 MHz, $CDCl_3$, 298 K): δ/ppm = 4.20 (q, *J* = 7.1 Hz, 2H), 1.41–1.34 (m, 1H) 1.29 (t, *J* = 7.1 Hz, 3H), 0.95–0.90 (m, 4H). **$^{13}$C{$^{1}$H} NMR** (100 MHz, $CDCl_3$, 298 K): δ/ppm = 154.0, 93.2, 68.7, 61.8, 24.2, 9.3 (2C), –0.5.

The NMR spectroscopic data are in accordance with those reported.[12]

### 5.2.11 Ethyl 3-(trimethylsilyl)propiolate (2i)

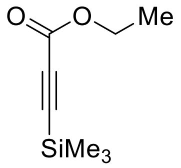


**2i**
$C_8H_{14}O_2Si$
M = 170.28 g/mol

Prepared from **S2b** (0.50 g, 3.5 mmol, 1.0 equiv), *p*TSA (60 mg, 0.35 mmol, 10 mol %) and ethanol (5.0 mL) according to **GP 3**. The product **2i** (488 mg, 2.87 mmol, 82%) was obtained as a colorless oil.

**$^{1}$H NMR** (400 MHz, $CDCl_3$, 298 K): δ/ppm = 4.23 (q, *J* = 7.2 Hz, 2H), 1.31 (q, *J* = 7.2 Hz, 3H), 0.24 (s, 9H). **$^{13}$C{$^{1}$H} NMR** (100 MHz, $CDCl_3$, 298 K): δ/ppm = 153.2, 94.9, 93.8, 62.2, 14.2, –0.7 (3C).

The NMR spectroscopic data are in accordance with those reported.[13]

### 5.2.12 Ethyl 4-methylpent-2-ynoate (2j)

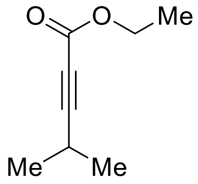


**2j**
$C_8H_{12}O_2$
M = 140.18 g/mol

Prepared from isobutyraldehyde (0.54 g, 0.68 mL, 7.5 mmol, 1.0 equiv), $CBr_4$ (5.0 g, 15 mmol, 2.0 equiv) and $PhP_3$ (7.9 g, 30 mmol, 4.0 equiv) according to **GP 6**. In the second step, the bromide (1.14 g, 5.00 mmol, 1.00 equiv), *n*BuLi (3.7 mL, 10 mmol, 2.0 equiv, 2.7 M in hexanes) and ethylchloroformate (0.81 g, 0.71 mL, 7.5 mmol, 1.5 equiv) were used. The crude product was purified by FCC on silica using cyclohexane:MTBE (94:6) as eluent to afford the product **2j** (567 mg, 4.04 mmol, 81%) as a red oil.

**$R_f$** = 0.43 (cyclohexane:MTBE = 94:6). **$^1$H NMR** (400 MHz, $CDCl_3$, 298 K): δ/ppm = 4.21 (q, *J* = 7.1 Hz, 2H), 2.69 (psept, *J* = 6.9 Hz, 1H), 1.30 (t, *J* = 7.1 Hz, 3H), 1.23 (d, *J* = 6.9 Hz, 6H). **$^{13}$C{$^1$H} NMR** (100 MHz, $CDCl_3$, 298 K): δ/ppm = 154.2, 94.1, 72.5, 61.9, 21.9 (2C), 20.6, 14.2.

The NMR spectroscopic data are in accordance with those reported.[14]

### 5.2.13 Ethyl 4-ethylhex-2-ynoate (2k)

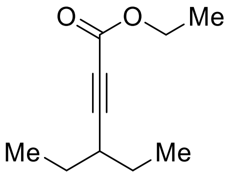


**2k**
$C_{10}H_{16}O_2$
M = 168.24 g/mol

Prepared from 2-ethylbutanal (0.83 g, 1.0 mL, 8.3 mmol, 1.0 equiv), $CBr_4$ (5.50 g, 16.6 mmol, 2.00 equiv) and $PhP_3$ (8.70 g, 33.2 mmol, 4.00 equiv) according to **GP 6**. In the second step, the bromide (1.28 g, 5.00 mmol, 1.00 equiv), *n*BuLi (3.7 mL, 10 mmol, 2.0 equiv, 2.7 M in hexanes) and ethylchloroformate (0.81 g, 0.71 mL, 7.5 mmol, 1.5 equiv) were used. The crude product was purified by FCC on silica using cyclohexane:MTBE (97:3) as eluent to afford the product **2k** (625 mg, 3.72 mmol, 80%) as a red oil.

**$R_f$** = 0.34 (cyclohexane:MTBE = 97:3). **$^1$H NMR** (400 MHz, $CDCl_3$, 298 K): δ/ppm = 4.22 (q, *J* = 7.2 Hz, 2H), 2.36–2.32 (m, 1H), 1.58–1.51 (m, 4H), 1.31 (t, *J* = 7.1 Hz, 3H), 1.01 (t, *J* = 7.4 Hz, 6H). **$^{13}$C{$^1$H} NMR** (100 MHz, $CDCl_3$, 298 K): δ/ppm = 154.2, 92.5, 74.5, 61.9, 35.2, 26.9 (2C), 14.2, 11.9 (2C). **HRMS** (APCI): calculated for $C_{10}H_{17}O_2^+$ [$(M+H)^+$]: 169.1223; found: 169.1222. **IR** (ATR): $\tilde{\nu}$/cm$^{-1}$ = 3414, 2967, 2934, 2876, 2221, 2094, 2008, 1886, 1706, 1460, 1365, 1237, 1148, 1113, 1051, 921, 858, 751.

### 5.2.14 Ethyl 3-cyclohexylpropiolate (2l)

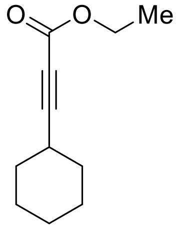


**2l**
$C_{11}H_{16}O_2$
M = 180.25 g/mol

Prepared from cyclohexylcarbaldehyde (0.93 g, 8.3 mmol, 1.0 equiv), $CBr_4$ (5.5 g, 17 mmol, 2.0 equiv) and $PhP_3$ (8.7 g, 33 mmol, 4.0 equiv) according to **GP 6**. In the second step, the bromide (1.34 g, 5.00 mmol, 1.00 equiv), *n*BuLi (4.0 mL, 10 mmol, 2.0 equiv, 2.5 M in hexanes) and ethylchloroformate (0.81 g, 0.71 mL, 7.5 mmol, 1.5 equiv) were used. The crude product was purified by FCC on silica using cyclohexane:MTBE (95:5) as eluent to afford the product **2l** (875 mg, 4.75 mmol, 95%) as a colorless oil.

**$^1$H NMR** (400 MHz, $CDCl_3$, 298 K): δ/ppm = 4.21 (q, *J* = 7.1 Hz, 2H), 2.56–2.48 (m, 1H), 1.87–1.80 (m, 2H), 1.76–1.67 (m, 2H), 1.57–1.47 (m, 4H), 1.37–1.29 (m, 2H), 1.30 (t, *J* = 7.1 Hz, 3H). **$^{13}$C{$^1$H} NMR** (100 MHz, $CDCl_3$, 298 K): δ/ppm = 154.2, 93.0, 73.2, 61.9, 31.6 (2C), 29.0, 25.7, 24.8 (2C), 14.2.

The NMR spectroscopic data are in accordance with those reported.[12]

### 5.2.15 Ethyl 4,4-dimethylpent-2-ynoate (2m)

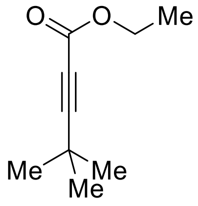


**2m**
$C_9H_{14}O_2$
M = 154.21 g/mol

Prepared from 3,3-dimethyl-1-butyne (0.36, g, 0.54 mL, 4.4 mmol, 1.1 equiv), *n*BuLi (1.5 mL, 4.0 mmol, 1.0 equiv, 2.7 M in hexanes) and ethylchloroformate (0.65 g, 0.57 mL, 4.5 mmol, 1.5 equiv) according to **GP 5**. Evaporation of all volatiles afforded the product **2m** (600 mg, 2.97 mmol, 99%) without further purification as a colorless oil.

**$^1$H NMR** (400 MHz, $CDCl_3$, 298 K): δ/ppm = 4.21 (q, *J* = 7.1 Hz, 2H), 1.30 (t, *J* = 7.1 Hz, 3H), 1.28 (s, 9H). **$^{13}$C{$^1$H} NMR** (100 MHz, $CDCl_3$, 298 K): δ/ppm = 154.3, 96.5, 72.0, 61.9, 30.1 (3C), 27.7, 14.2.

The NMR spectroscopic data are in accordance with those reported.[12]

# 6 Experimental Details for the Synthesis of $H_2$-Surrogates

## 6.1 General Procedure for the Synthesis of $H_2$-Surrogates (GP 7)

The cyclohexadienes are synthesized following a modified literature procedure.[15] In an argon-filled glovebox, **L2**·$CoBr_2$ (5 mol %), Zn (0.5 equiv) and $NaBAr^F$ (0.1 equiv) are dissolved in $CH_2Cl_2$ (10 mL/mmol) and stirred for 5 min at room temperature. The indicated dienes **1a**–**c** (2 equiv) and alkynes **2a**–**m** (1 equiv) are added and stirred until GLC indicated complete consumption of the alkyne (4–72 h). The reaction vessel is then transferred out of the glovebox, diluted with *n*-pentane and filtered through a plug of silica. All volatile components are removed and the residue is purified by FCC on silica gel using mixtures of *n*-pentane:$Et_2O$ as eluent. Further purification is carried out by automatic reverse phase FCC using mixtures of acetonitrile:water as eluent.

Racemic surrogates are prepared using achiral [Co(DPPP)$Br_2$] as catalyst following the same procedure. For some racemic surrogates, the first purification step is sufficient.

## 6.2 Preparation and Characterization of $H_2$-Surrogates

### 6.2.1 Methyl (*R*)-6-pentyl-2-methylcyclohexa-1,4-diene-1-carboxylate (3aa)

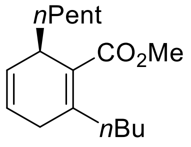


**3aa**
$C_{17}H_{28}O_2$
M = 264.21 g/mol

Prepared from **L2·**$CoBr_2$ (38 mg, 75 µmol, 5.0 mol %), Zn (49 mg, 0.75 mmol, 0.50 equiv), $NaBAr^F$ (133 mg, 150 µmol, 0.100 equiv), (*E*)-nona-1,3-diene (**1a**, 373 mg, 3.00 mmol, 2.00 equiv) and methyl-2-heptynoate (**2a**, 210 mg, 1.50 mmol, 1.00 equiv) according to **GP 7**. The residue was purified by FCC on silica gel using *n*-pentane:$Et_2O$ (98:2) as eluent. The resulting mixture was then purified by automatic reverse phase FCC using water:acetonitrile (35:65 → 0:100) as eluent to afford the product **3aa** (200 mg, 756 µmol, 68%, 92% ee) as a colorless oil.

**$R_f$** = 0.28 (*n*-pentane:$Et_2O$ = 98:2). **$^1$H NMR** (500 MHz, $CDCl_3$, 298 K): δ/ppm = 5.73–5.67 (m, 2H), 3.73 (s, 3H), 3.25–3.22 ($m_c$, 1H), 2.82–2.62 (m, 2H), 2.32–2.30 (m, 2H), 1.42–1.20 (m, 12H), 0.90 (t, *J* = 7.2 Hz, 3H), 0.86 (t, *J* = 7.0 Hz, 3H). **$^{13}C\{^1H\}$ NMR** (100 MHz, $CDCl_3$, 298 K): δ/ppm = 169.8, 144.3, 129.0, 127.4, 122.9, 51.2, 37.2, 35.3, 34.8, 32.1, 31.9, 30.8, 25.2, 22.9, 22.7, 14.2, 14.1. **HRMS** (APCI): calculated for $C_{17}H_{29}O_2^+$ [$(M+H)^+$]: 265.2162; found: 265.2165.

**IR** (ATR): ṽ/cm$^{-1}$ = 3421, 3027, 2954, 2926, 2857, 2669, 2505, 2215, 2155, 2151, 2117, 2064, 2023, 1953, 1715, 1627, 1458, 1432, 1376, 1228, 1189, 1079, 1045, 966, 805, 710. **Optical Rotation**: $[\alpha]_D^{RT}$ = –0.91 (*c* 0.53, $CHCl_3$).

The enantiomeric ratio of **3aa** was determined by HPLC analysis on a chiral stationary phase (*Daicel* Chiralcel OD-H column, column temperature 20 °C, mobile phase *n*-heptane:isopropanol = 99.9:0.1, flow rate 0.8 mL/min, λ = 254 nm): $t_R$ = 9.5 min (major), $t_R$ = 13.5 min (minor).

### 6.2.2 Hexyl (*R*)-6-pentyl-2-methylcyclohexa-1,4-diene-1-carboxylate (3ab)

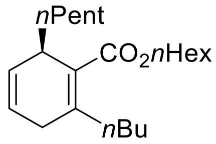


**3ab**
$C_{22}H_{38}O_2$
M = 324.54 g/mol

Prepared from **L2**·$CoBr_2$ (38 mg, 75 µmol, 5.0 mol %), Zn (49 mg, 0.75 mmol, 0.50 equiv), $NaBAr^F$ (133 mg, 150 µmol, 0.100 equiv), (*E*)-nona-1,3-diene (**1a**, 373 mg, 3.00 mmol, 2.00 equiv) and hexyl-2-heptynoate (**2b**, 315 mg, 1.50 mmol, 1.00 equiv) according to **GP 7**. The residue was purified by FCC on silica gel using *n*-pentane:$Et_2O$ (99:1) as eluent. The resulting mixture was then purified by automatic reverse phase FCC using water:acetonitrile (20:80 → 0:100) to afford the product **3ab** (268 mg, 801 µmol, 54%, 91% ee) as a colorless oil.

**$R_f$** = 0.40 (*n*-pentane:$Et_2O$ = 99:1). **$^1$H NMR** (400 MHz, $CDCl_3$, 298 K): δ/ppm = 5.74–5.67 (m, 2H), 4.14 (t, *J* = 6.7 Hz, 2H), 3.26–3.21 (m, 1H), 2.82–2.62 (m, 2H), 2.32–2.21 (m, 2H), 1.69–1.63 (m, 2H), 1.44–1.20 (m, 18H), 0.91–0.88 (m, 6H), 0.86 (t, *J* = 7.0 Hz, 3H). **$^{13}$C{$^1$H} NMR** (100 MHz, $CDCl_3$, 298 K): δ/ppm = 170.0, 143.5, 129.0, 127.8, 122.9, 64.4, 37.3, 35.3, 34.8, 32.2, 31.8, 31.6, 30.9, 28.9, 26.0, 25.2, 23.0, 22.8, 22.7, 14.2, 14.2 (2C). **HRMS** (APCI): calculated for $C_{22}H_{39}O_2^+$ [$(M+H)^+$]: 335.2945; found: 335.2948. **IR** (ATR): ṽ/cm$^{-1}$ = 3423, 3028, 2954, 2925, 2857, 2408, 2222, 2115, 1711, 1624, 1459, 1377, 1223, 1183, 1136, 1080, 1039, 968, 911, 804, 726. **Optical Rotation**: $[\alpha]_D^{RT}$ = –0.68 (*c* 0.49, $CHCl_3$).

The enantiomeric ratio of **3ab** was determined by HPLC analysis on a chiral stationary phase (*Daicel* Chiralcel OD-H column, column temperature 20 °C, mobile phase *n*-heptane, flow rate 1.0 mL/min, λ = 211 nm): $t_R$ = 7.2 min (major), $t_R$ = 11.3 min (minor).

### 6.2.3 Isopropyl (*R*)-6-pentyl-2-methylcyclohexa-1,4-diene-1-carboxylate (3ac)

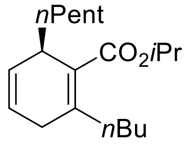


**3ac**
$C_{19}H_{32}O_2$
M = 292.46 g/mol

Prepared from **L2**·$CoBr_2$ (38 mg, 75 µmol, 5.0 mol %), Zn (49 mg, 0.75 mmol, 0.50 equiv), $NaBAr^F$ (133 mg, 150 µmol, 0.100 equiv), (*E*)-nona-1,3-diene (**1a**, 373 mg, 3.00 mmol, 2.00 equiv) and isopropyl-2-heptynoate (**2c**, 252 mg, 1.50 mmol, 1.00 equiv) according to **GP 7**. The residue was purified by FCC on silica gel using *n*-pentane:$Et_2O$ (98:2) as eluent. The resulting mixture was then purified by automatic reverse phase FCC using water:acetonitrile (30:70 → 0:100) to afford the product **3ac** (215 mg, 735 µmol, 55%, 92% ee) as a colorless oil.

**$R_f$** = 0.32 (*n*-pentane:$Et_2O$ = 98:2). **$^1$H NMR** (400 MHz, $CDCl_3$, 298 K): δ/ppm = 5.72–5.66 (m, 2H), 5.12 (psept, *J* = 6.3 Hz, 1H), 3.24–3.20 (m, 1H), 2.80–2.60 (m, 2H), 2.30–2.19 (m, 2H), 1.49–1.19 (m, 12H), 1.28 (dd, *J* = 8.9 Hz, 6.3 Hz, 6H), 0.90 (t, *J* = 7.2 Hz, 3H), 0.86 (t, *J* = 7.0 Hz, 3H). **$^{13}$C{$^1$H} NMR** (100 MHz, $CDCl_3$, 298 K): δ/ppm = 169.1, 142.5, 129.0, 128.0, 122.9, 67.4, 37.2, 35.1, 34.7, 32.2, 31.7, 30.9, 25.2, 23.0, 22.7, 22.1, 22.0, 14.2, 14.2. **HRMS** (APCI): calculated for $C_{19}H_{33}O_2^+$ [$(M+H)^+$]: 293.2475; found: 293.2478 **IR** (ATR): $\tilde{\nu}$/cm$^{-1}$ = 3421, 3028, 2956, 2926, 2858, 2279, 2207, 2111, 1709, 1631, 1463, 1372, 1228, 1178, 1143, 1108, 1078, 1036, 968, 917, 838, 800, 708. **Optical Rotation**: $[\alpha]_D^{RT}$ = –0.81 (*c* 0.46, $CHCl_3$).

The enantiomeric ratio of **3ac** was determined by HPLC analysis on a chiral stationary phase (*Daicel* Chiralcel OD-H column, column temperature 20 °C, mobile phase *n*-heptane:isopropanol = 99.9:0.1, flow rate 0.8 mL/min, λ = 254 nm): $t_R$ = 7.7 min (major), $t_R$ = 11.5 min (minor).

### 6.2.4 Phenyl (*R*)-6-pentyl-2-methylcyclohexa-1,4-diene-1-carboxylate (3ad)

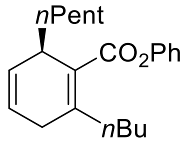


**3ad**
$C_{22}H_{30}O_2$
M = 326.48 g/mol

Prepared from **L2**·$CoBr_2$ (38 mg, 75 µmol, 5.0 mol %), Zn (49 mg, 0.75 mmol, 0.50 equiv), $NaBAr^F$ (133 mg, 150 µmol, 0.100 equiv), (*E*)-nona-1,3-diene (**1a**, 373 mg, 3.00 mmol, 2.00 equiv) and phenyl-2-heptynoate (**2d**, 303 mg, 1.50 mmol, 1.00 equiv) according to **GP 7**. The residue was purified by FCC on silica gel using *n*-pentane:$Et_2O$ (96:4) as eluent. The resulting mixture was then purified by automatic reverse phase FCC using water:acetonitrile (30:70 → 0:100) to afford the product **3ad** (198 mg, 606 µmol, 39%, 90% ee) as a colorless oil.

**$R_f$** = 0.16 (*n*-pentane:$Et_2O$ = 96:4). **$^1$H NMR** (400 MHz, $CDCl_3$, 298 K): δ/ppm = 7.42–7.38 (m, 2H), 7.25–7.21 (m, 1H), 7.11–7.09 (m, 2H), 5.80–5.72 (m, 2H), 3.46–3.43 (m, 1H), 2.93–2.70 (m, 2H), 2.50–2.32 (m, 2H), 1.60–1.42 (m, 4H), 1.40–1.26 (m, 10H), 0.90 (t, *J* = 7.2 Hz, 3H), 0.87 (t, *J* = 6.8 Hz, 3H). **$^{13}$C{$^1$H} NMR** (100 MHz, $CDCl_3$, 298 K): δ/ppm = 167.3, 151.0, 147.5, 129.6 (2C), 129.0, 126.6, 125.8, 122.8, 121.9 (2C), 37.3, 35.6, 35.1, 32.4, 32.2, 31.0, 25.2, 23.0, 22.8, 14.2, 14.2. **HRMS** (APCI): calculated for $C_{22}H_{31}O_2^+$ [(M+H)$^+$]: 327.2319; found: 327.2322. **IR** (ATR): ṽ/cm$^{-1}$ = 3040, 2955, 2926, 2858, 2229, 2118, 1986, 1945, 1725, 1636, 1591, 1490, 1458, 1377, 1224, 1185, 1114, 1070, 1039, 1019, 910, 841, 739, 687. **Optical Rotation**: $[\alpha]_D^{RT}$ = +0.40 (*c* 0.46, $CHCl_3$).

The enantiomeric ratio of **3ad** was determined by HPLC analysis on a chiral stationary phase (*Daicel* Chiralpak AD-H column, column temperature 20 °C, mobile phase *n*-heptane:isopropanol = 99.8:0.2, flow rate 0.8 mL/min, λ = 254 nm): $t_R$ = 11.6 min (major), $t_R$ = 13.2 min (minor).

### 6.2.5 Cyclopentyl (*R*)-6-pentyl-2-methylcyclohexa-1,4-diene-1-carboxylate (3ae)

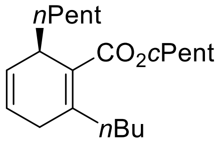


**3ae**

$C_{21}H_{34}O_2$

M = 318.50 g/mol

Prepared from **L2**·$CoBr_2$ (38 mg, 75 μmol, 5.0 mol %), Zn (49 mg, 0.75 mmol, 0.50 equiv), $NaBAr^F$ (133 mg, 150 μmol, 0.100 equiv), (*E*)-nona-1,3-diene (**1a**, 373 mg, 3.00 mmol, 2.00 equiv) and cyclopentyl-2-heptynoate (**2e**, 291 mg, 1.50 mmol, 1.00 equiv) according to **GP 7**. The residue was purified by FCC on silica gel using *n*-pentane:$Et_2O$ (97.5:2.5) as eluent. The resulting mixture was then purified by automatic reverse phase FCC using water:acetonitrile (25:75 → 0:100) to afford the product **3ae** (264 mg, 829 μmol, 51%, 92% ee) as a colorless oil.

$\mathbf{R_f}$ = 0.35 (*n*-pentane:$Et_2O$ = 97.5:2.5). **$^1$H NMR** (400 MHz, $CDCl_3$, 298 K): δ/ppm = 5.69 ($m_c$, 2H), 5.26 ($m_c$, 1H), 3.26–3.19 (m, 1H), 2.82–2.59 (m, 2H). 2.32–2.18 (m, 2H), 1.93–1.82 (m, 2H), 1.77–1.58 (m, 6H), 1.46–1.12 (m, 12H) 0.90 (t, *J* = 7.1 Hz, 3H), 0.86 (t, *J* = 7.0 Hz, 3H). **$^{13}$C{$^1$H} NMR** (100 MHz, $CDCl_3$, 298 K): δ/ppm = 169.3, 142.9, 129.0 (2C), 127.9, 122.9 (2C), 37.3, 35.2, 34.8, 32.9, 32.9, 32.2, 31.7, 30.9, 25.2, 23.9 (2C), 23.1, 22.7, 14.2. **HRMS** (APCI): calculated for $C_{21}H_{35}O_2^+$ [$(M+H)^+$]: 319.2632; found: 319.2632. **IR** (ATR): $\tilde{\nu}$/cm$^{-1}$ = 3417, 3027, 2955, 2925, 2857, 2319, 2117, 2061, 1995, 1707, 1626, 1458, 1374, 1226, 1165, 1080, 1037, 963, 900, 843, 803, 709. **Optical Rotation**: $[\alpha]_D^{RT}$ = –0.57 (*c* 0.32, $CHCl_3$).

The enantiomeric ratio of **3ae** was determined by HPLC analysis on a chiral stationary phase (*Daicel* Chiralcel OD-H column, column temperature 20 °C, mobile phase *n*-heptane:isopropanol = 99.9:0.1, flow rate 0.8 mL/min, λ = 254 nm): $t_R$ = 9.4 min (major), $t_R$ = 15.0 min (minor).

### 6.2.6 Neopentyl (*R*)-6-pentyl-2-methylcyclohexa-1,4-diene-1-carboxylate (3af)

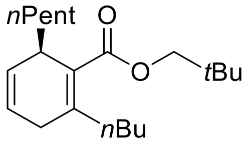


**3af**

$C_{21}H_{36}O_2$

M = 320.52 g/mol

Prepared from **L2**·$CoBr_2$ (38 mg, 75 µmol, 5.0 mol %), Zn (49 mg, 0.75 mmol, 0.50 equiv), $NaBAr^F$ (133 mg, 150 µmol, 0.100 equiv), (*E*)-nona-1,3-diene (**1a**, 373 mg, 3.00 mmol, 2.00 equiv) and neopentyl-2-heptynoate (**2f**, 294 mg, 1.50 mmol, 1.00 equiv) according to **GP 7**. The residue was purified by FCC on silica gel using *n*-pentane:$Et_2O$ (97:3) as eluent. The resulting mixture was then purified by automatic reverse phase FCC using water:acetonitrile (25:75 → 0:100) to afford the product **3af** (252 mg, 786 µmol, 44%, 90% ee) as a colorless oil.

$R_f$ = 0.50 (*n*-pentane:$Et_2O$ = 97:3). **$^1$H NMR** (400 MHz, $CDCl_3$, 298 K): δ/ppm = 5.75–5.68 (m, 2H), 3.89–3.77 (m, 2H), 3.29–3.24 (m, 1H), 2.84–2.62 (m, 2H), 2.32–2.25 (m, 2H), 1.45–1.21 (m, 12H), 0.97 (s, 9H), 0.89 (t, *J* = 7.2 Hz, 3H), 0.85 (t, *J* = 7.0, 3H). **$^{13}$C{$^1$H} NMR** (100 MHz, $CDCl_3$, 298 K): δ/ppm = 169.6, 143.9, 129.0, 127.6, 123.0, 73.9, 37.3, 35.4, 34.8, 32.3, 31.9, 31.4, 30.9, 26.7 (3C), 25.2, 23.0, 22.8, 14.2, 14.2. **HRMS** (APCI): calculated for $C_{21}H_{37}O_2^+$ [$(M+H)^+$]: 321.2788; found: 321.2782. **IR** (ATR): $\tilde{\nu}$/cm$^{-1}$ = 3419, 3029, 2955, 2926, 2859, 2330, 2129, 2064, 1900, 1712, 1626, 1463, 1366, 1225, 1144, 1079, 1039, 981, 937, 805, 709. **Optical Rotation**: $[\alpha]_D^{RT}$ = –0.41 (*c* 0.41, $CHCl_3$).

The enantiomeric ratio of **3af** was determined by HPLC analysis on a chiral stationary phase (*Daicel* Chiralcel OD-H column, column temperature 20 °C, mobile phase *n*-heptane:isopropanol = 99.9:0.1, flow rate 0.8 mL/min, λ = 254 nm): $t_R$ = 7.1 min (major), $t_R$ = 10.8 min (minor).

### 6.2.7 Ethyl (*R*)-6-pentyl-2-methylcyclohexa-1,4-diene-1-carboxylate (3ag)

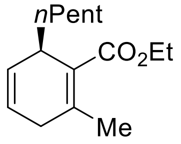


**3ag**
$C_{15}H_{24}O_2$
M = 236.36 g/mol

Prepared from **L2**·$CoBr_2$ (38 mg, 75 µmol, 5.0 mol %), Zn (49 mg, 0.75 mmol, 0.50 equiv), $NaBAr^F$ (133 mg, 150 µmol, 0.100 equiv), (*E*)-nona-1,3-diene (**1a**, 373 mg, 3.00 mmol, 2.00 equiv) and ethyl but-2-ynoate (**2g**, 189 mg, 1.50 mmol, 1.00 equiv) according to **GP 7**. The residue was purified by FCC on silica gel using *n*-pentane:$Et_2O$ (96:4) as eluent. The resulting mixture was then purified by automatic reverse phase FCC using water:acetonitrile (35:65 → 0:100) to afford the product **3ag** (184 mg, 778 µmol, 67%, 93% ee) as a colorless oil.

$\mathbf{R_f}$ = 0.29 (*n*-pentane:$Et_2O$ = 96:4). $^1$**H NMR** (400 MHz, $CDCl_3$, 298 K): δ/ppm = 5.75–5.65 (m, 2H), 4.26–4.18 (m, 2H) 3.26–3.21 (m, 1H), 2.80–2.59 (m, 2H), 1.94 (s, 3H), 1.47–1.19 (m, 8H), 1.31 (t, *J* = 7.1 Hz, 3H), 0.86 (t, *J* = 6.9 Hz, 3H). $^{13}$**C{$^1$H} NMR** (100 MHz, $CDCl_3$, 298 K): δ/ppm = 169.3, 140.1, 129.1, 127.6, 122.6, 60.1, 37.0, 35.4, 34.2, 32.2, 25.3, 22.7, 21.2, 14.5, 12.2. **HRMS** (APCI): calculated for $C_{15}H_{25}O_2^+$ [$(M+H)^+$]: 237.1849; found: 237.1850. **IR** (ATR): $\tilde{\nu}$/cm$^{-1}$ = 3419, 3029, 2925, 2856, 2319, 2259, 2087, 1711, 1635, 1460, 1372, 1230, 1097, 1059, 966, 906, 840, 803, 708. **Optical Rotation**: $[\alpha]_D^{RT}$ = –0.84 (*c* 0.35, $CHCl_3$).

The enantiomeric ratio of **3ag** was determined by chiral GLC analysis (*Sigma-Aldrich* Astec Chiraldex B-DM column (30 m × 0.25 mm, 0.12 µm film thickness), column temperature 60 °C (30 min), ramp of 1 °C/min to 175 °C (10 min), ramp of 3 °C/min to 200 °C (10 min), flow rate isobaric 14 psi): $t_R$ = 102.8 min (minor), $t_R$ = 103.4 min (major).

### 6.2.8 Ethyl (*R*)-2-cyclopropyl-6-pentylcyclohexa-1,4-diene-1-carboxylate (3ah)

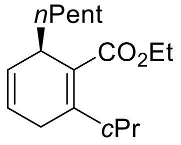


**3ah**
$C_{17}H_{26}O_2$
M = 262.39 g/mol

Prepared from **L2**·$CoBr_2$ (38 mg, 75 µmol, 5.0 mol %), Zn (49 mg, 0.75 mmol, 0.50 equiv), $NaBAr^F$ (133 mg, 150 µmol, 0.100 equiv), (*E*)-nona-1,3-diene (**1a**, 373 mg, 3.00 mmol, 2.00 equiv) and ethyl 3-cyclopropylpropiolate (**2h**, 207 mg, 1.50 mmol, 1.00 equiv) according to **GP 7**. The residue was purified by FCC on silica gel using *n*-pentane:$Et_2O$ (96:4) as eluent. The

resulting mixture was then purified by automatic reverse phase FCC using water:acetonitrile (35:65 → 0:100) to afford the product **3ah** (182 mg, 694 μmol, 38%, 96% ee) as a colorless oil.

**$R_f$** = 0.32 (*n*-pentane:$Et_2O$ = 96:4). **$^1$H NMR** (400 MHz, $CDCl_3$, 298 K): δ/ppm = 5.74–5.71 (m, 2H), 4.24 (q, *J* = 7.1 Hz, 2H), 3.25–3.21 (m, 1H), 2.45–2.20 (m, 3H), 1.49–1.24 (m, 8H), 1.31 (t, *J* = 7.1 Hz, 3H), 0.86 (t, *J* = 6.8 Hz, 3H), 0.73–0.57 (m, 4H). **$^{13}$C{$^1$H} NMR** (100 MHz, $CDCl_3$, 298 K): δ/ppm = 170.1, 141.7, 129.0, 128.9, 122.3, 60.2, 37.7, 35.3, 32.2, 26.1, 25.4, 22.7, 14.7, 14.4, 14.2, 5.2, 5.1. **HRMS** (APCI): calculated for $C_{17}H_{27}O_2^+$ [(M+H)$^+$]: 263.2006; found: 263.2006. **IR** (ATR): $\tilde{\nu}$/cm$^{-1}$ = 3085, 2925, 2856, 2665, 2134, 1987, 1910, 1703, 1613, 1459, 1374, 1218, 1102, 1048, 964, 919, 864, 814, 784, 713. **Optical Rotation**: $[\alpha]_D^{RT}$ = –0.28 (*c* 0.65, $CHCl_3$).

The enantiomeric ratio of **3ah** was determined by HPLC analysis on a chiral stationary phase (*Daicel* Chiralpak AD-H column, column temperature 20 °C, mobile phase *n*-heptane:isopropanol = 99.9:0.1, flow rate 0.8 mL/min, λ = 254 nm): $t_R$ = 12.9 min (major), $t_R$ = 15.9 min (minor).

### 6.2.9 Ethyl (*R*)-6-pentyl-2-(trimethylsilyl)cyclohexa-1,4-diene-1-carboxylate (3ai)

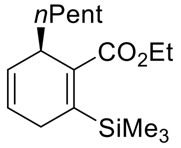


**3ai**
$C_{17}H_{30}O_2Si$
M = 394.51 g/mol

Prepared from **L2**·$CoBr_2$ (38 mg, 75 μmol, 5.0 mol %), Zn (49 mg, 0.75 mmol, 0.50 equiv), NaBAr$^F$ (133 mg, 150 μmol, 0.100 equiv), (*E*)-nona-1,3-diene (**1a**, 373 mg, 3.00 mmol, 2.00 equiv) and ethyl 3-(trimethylsilyl)propiolate (**2i**, 255 mg, 1.50 mmol, 1.00 equiv) according to **GP 7**. The residue was purified by FCC on silica gel using *n*-pentane:$Et_2O$ (99:1) as eluent. The resulting mixture was then purified by automatic reverse phase FCC using water:acetonitrile (33:67 → 0:100) to afford the product **3ai** (258 mg, 876 μmol, 60%, 57% ee) as a colorless oil.

**$R_f$** = 0.21 (*n*-pentane:$Et_2O$ = 99:1). **$^1$H NMR** (500 MHz, $CDCl_3$, 298 K): δ/ppm = 5.77–5.72 (m, 2H), 4.24–4.13 (m, 2H), 3.23–3.20 (m, 1H), 2.91–2.73 (m, 2H). 1.50–1.22 (m, 8H), 1.31 (t, *J* = 7.2 Hz, 3H), 0.86 (t, *J* = 6.9 Hz, 3H), 0.14 (s, 9H). **$^{13}$C{$^1$H} NMR** (126 MHz, $CDCl_3$, 298 K): δ/ppm = 169.7 145.6, 141.1, 128.8, 123.8, 60.5, 36.9, 35.4, 32.1, 31.5, 25.2, 22.7, 14.4, 14.2, 0.3 (3C). **$^1$H/$^{29}$Si HMQC NMR** (500/99 MHz, $CDCl_3$, 298 K, optimized for *J* = 7 Hz): δ/ppm =

0.14/–5.7. **HRMS** (APCI): calculated for $C_{17}H_{31}O_2Si^+$ [(M+H)$^+$]: 295.2088; found: 295.2093. **IR** (ATR): ṽ/cm$^{-1}$ = 3422, 3026, 2954, 2927, 2857, 2347, 2256, 2127, 2082, 1904, 1712, 1587, 1461, 1392, 1366, 1237, 1174, 1140, 1097, 1048, 956, 922, 837, 762, 708, 679. **Optical Rotation**: $[\alpha]_D^{RT}$ = –0.43 (*c* 0.35, $CHCl_3$).

The enantiomeric ratio of **3ai** was determined by HPLC analysis on a chiral stationary phase (*Daicel* Chiralpak IC column, column temperature 20 °C, mobile phase *n*-heptane:isopropanol = 99.9:0.1, flow rate 0.8 mL/min, λ = 254 nm): $t_R$ = 12.4 min (major), $t_R$ = 14.1 min (minor).

### 6.2.10 Ethyl (*R*)-6-pentyl-2-isopropylcyclohexa-1,4-diene-1-carboxylate (3aj)

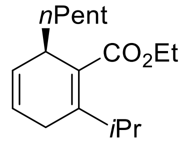


**3aj**
$C_{17}H_{28}O_2$
M = 264.41 g/mol

Prepared from **L2**·$CoBr_2$ (38 mg, 75 μmol, 5.0 mol %), Zn (49 mg, 0.75 mmol, 0.50 equiv), NaBAr$^F$ (133 mg, 150 μmol, 0.100 equiv), (*E*)-nona-1,3-diene (**1a**, 373 mg, 3.00 mmol, 2.00 equiv) and ethyl 4-methylpent-2-ynoate (**2j**, 210 mg, 1.50 mmol, 1.00 equiv) according to **GP 7**. The residue was purified by FCC on silica gel using *n*-pentane:$Et_2O$ (98:2) as eluent. The resulting mixture was then purified by automatic reverse phase FCC using water:acetonitrile (35:65 → 0:100) to afford the product **3aj** (188 mg, 711 μmol, 59%, 95% ee) as a colorless oil.

$\mathbf{R_f}$ = 0.35 (*n*-pentane:$Et_2O$ = 98:2). **$^1$H NMR** (400 MHz, $CDCl_3$, 298 K): δ/ppm = 5.74–5.67 (m, 2H), 4.21 (t, *J* = 7.1 Hz, 2H), 3.19–3.15 (m, 1H), 3.06 (pq, *J* = 6.8 Hz, 1H), 2.75–2.58 (m, 2H), 1.49–1.19 (m, 8H), 1.39 (t, *J* = 7.1 Hz, 3H), 1.05 (d, *J* = 6.8 Hz, 3H), 0.98 (d, *J* = 6.9 Hz, 3H), 0.86 (t, *J* = 6.9 Hz, 3H). **$^{13}$C{$^1$H} NMR** (100 MHz, $CDCl_3$, 298 K): δ/ppm = 170.2, 145.2, 128.6, 127.1, 123.1, 60.2, 37.4, 34.9, 32.2, 31.5, 25.0, 24.6, 22.7, 21.1, 20.8, 14.4, 14.2. **HRMS** (APCI): calculated for $C_{17}H_{29}O_2^+$ [(M+H)$^+$]: 265.2162; found: 265.2157. **IR** (ATR): ṽ/cm$^{-1}$ = 3027, 2957, 2926, 2860, 2393, 2327, 2125, 1993, 1900, 1713, 1621, 1463, 1365, 1298, 1229, 1095, 1055, 965, 933, 868, 832, 795, 710. **Optical Rotation**: $[\alpha]_D^{RT}$ = –0.68 (*c* 0.48, $CHCl_3$).

The enantiomeric ratio of **3aj** was determined by HPLC analysis on a chiral stationary phase (*Daicel* Chiralpak IC column, column temperature 20 °C, mobile phase *n*-heptane:isopropanol = 99.8:0.2, flow rate 0.8 mL/min, λ = 254 nm): $t_R$ = 16.0 min (major), $t_R$ = 18.7 min (minor).

### 6.2.11 Ethyl (*R*)-6-pentyl-2-(pentan-3-yl)cyclohexa-1,4-diene-1-carboxylate (3ak)

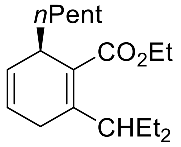

**3ak**
$C_{19}H_{32}O_2$
M = 292.46 g/mol

Prepared from **L2**·$CoBr_2$ (38 mg, 75 µmol, 5.0 mol %), Zn (49 mg, 0.75 mmol, 0.50 equiv), $NaBAr^F$ (133 mg, 150 µmol, 0.100 equiv), (*E*)-nona-1,3-diene (**1a**, 373 mg, 3.00 mmol, 2.00 equiv) and ethyl 4-ethylhex-2-ynoate (**2k**, 252 mg, 1.50 mmol, 1.00 equiv) according to **GP 7**. The residue was purified by FCC on silica gel using *n*-pentane:$Et_2O$ (98:2) as eluent. The resulting mixture was then purified by automatic reverse phase FCC using water:acetonitrile (40:60 → 0:100) to afford the product **3ak** (223 mg, 762 µmol, 51%, 96% ee) as a colorless oil.

**$R_f$** = 0.47 (*n*-pentane:$Et_2O$ = 98:2). **$^1$H NMR** (400 MHz, $CDCl_3$, 298 K): δ/ppm = 5.74–5.66 (m, 2H), 4.20 (q, *J* = 7.1 Hz, 2H). 3.25–3.22 (m, 1H), 2.61–2.51 (m, 3H), 1.45–1.19 (m, 12H), 1.29 (t, *J* = 7.1 Hz, 3H), 0.86 (1, *J* = 7.6 Hz, 6H), 0.74 (t, *J* = 7.4 Hz, 3H). **$^{13}C\{^1H\}$ NMR** (100 MHz, $CDCl_3$, 298 K): δ/ppm = 170.6, 140.9, 130.6, 128.6, 123.1, 60.1, 45.8, 37.7, 34.6, 32.2, 26.3, 26.1, 24.9, 24.1, 22.7, 14.5, 14.2, 12.3, 12.2. **HRMS** (APCI): calculated for $C_{19}H_{33}O_2^+$ [$(M+H)^+$]: 293.2475; found: 293.2476. **IR** (ATR): ṽ/$cm^{-1}$ = 3027, 2958, 2927, 2861, 2592, 2425, 2247, 2125, 2069, 1716, 1637, 1458, 1375, 1298, 1225, 1197, 1075, 1044, 966, 940, 870, 794, 767, 710. **Optical Rotation**: $[\alpha]_D^{RT}$ = –0.58 (*c* 0.44, $CHCl_3$).

The enantiomeric ratio of **3ak** was determined by HPLC analysis on a chiral stationary phase (*Daicel* Chiralpak IC column, column temperature 20 °C, mobile phase *n*-heptane, flow rate 1.2 mL/min, λ = 254 nm): $t_R$ = 17.9 min (major), $t_R$ = 20.1 min (minor).

### 6.2.12 Ethyl (*R*)-3-pentyl-[1,1'-bi(cyclohexane)]-1,4-diene-2-carboxylate (3al)

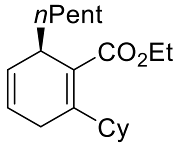

**3al**
$C_{20}H_{32}O_2$
M = 304.47 g/mol

Prepared from **L2**·$CoBr_2$ (38 mg, 75 µmol, 5.0 mol %), Zn (49 mg, 0.75 mmol, 0.50 equiv), $NaBAr^F$ (133 mg, 150 µmol, 0.100 equiv), (*E*)-nona-1,3-diene (**1a**, 373 mg, 3.00 mmol, 2.00

equiv) and ethyl 3-cyclohexylpropiolate (**2l**, 270 mg, 1.50 mmol, 1.00 equiv) according to **GP 7**. The residue was purified by FCC on silica gel using *n*-pentane:$Et_2O$ (98:2) as eluent. The resulting mixture was then purified by automatic reverse phase FCC using water:acetonitrile (25:75 → 0:100) to afford the product **3al** (198 mg, 650 μmol, 50%, 89% ee) as a colorless oil.

$\mathbf{R_f}$ = 0.45 (*n*-pentane:$Et_2O$ = 98:2). $^1$**H NMR** (400 MHz, $CDCl_3$, 298 K): δ/ppm = 5.72–5.65 (m, 2H), 4.27–4.17 (m, 2H), 3.18 ($m_c$, 1H), 2.76–2.59 (m, 3H), 1.74–1.65 (m, 4H), 1.45–1.15 (m, 14H), 1.31 (t, *J* = 7.1 Hz, 3H), 0.86 (t, *J* = 6.9 Hz, 3H). $^{13}$**C{$^1$H} NMR** (100 MHz, $CDCl_3$, 298 K): δ/ppm = 170.3, 145.0, 128.6, 127.2, 123.2, 60.1, 42.7, 37.4, 35.0, 32.2, 31.4, 31.0, 26.7, 26.6, 26.3, 26.3, 25.0, 22.7, 14.5, 14.2. **HRMS** (APCI): calculated for $C_{20}H_{33}O_2^+$ [(M+H)$^+$]: 305.2475; found: 305.2470. **IR** (ATR): $\tilde{\nu}$/cm$^{-1}$ = 3421, 3026, 2923, 2851, 2667, 2323, 2121, 1896, 1710, 1618, 1447, 1368, 1300, 1225, 1192, 1093, 1048, 995, 966, 939, 888, 853, 790, 712. **Optical Rotation**: $[\alpha]_D^{RT}$ = –0.60 (*c* 0.40, $CHCl_3$).

The enantiomeric ratio of **3al** was determined by HPLC analysis on a chiral stationary phase (*Daicel* Chiralpak AD-H column, column temperature 20 °C, mobile phase *n*-heptane:isopropanol = 99.9:0.1, flow rate 0.8 mL/min, λ = 254 nm): $t_R$ = 9.2 min (major), $t_R$ = 10.6 min (minor).

### 6.2.13 Ethyl (*R*)-2-(tert-butyl)-6-pentylcyclohexa-1,4-diene-1-carboxylate (3am)

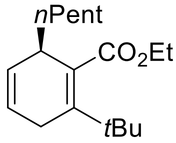


**3am**
$C_{18}H_{30}O_2$
M = 278.44 g/mol

Prepared from **L2**·$CoBr_2$ (38 mg, 75 μmol, 5.0 mol %), Zn (49 mg, 0.75 mmol, 0.50 equiv), NaBAr$^F$ (133 mg, 150 μmol, 0.100 equiv), (*E*)-nona-1,3-diene (**1a**, 373 mg, 3.00 mmol, 2.00 equiv) and ethyl 4,4-dimethylpent-2-ynoate (**2m**, 231 mg, 1.50 mmol, 1.00 equiv) according to **GP 7**. The residue was purified by FCC on silica gel using *n*-pentane:$Et_2O$ (98:2) as eluent. The resulting mixture was then purified by automatic reverse phase FCC using water:acetonitrile (35:65 → 0:100) to afford the product **3am** (102 mg, 366 μmol, 49%, 96% ee) as a colorless oil.

$\mathbf{R_f}$ = 0.24 (*n*-pentane:$Et_2O$ = 98:2). $^1$**H NMR** (400 MHz, $CDCl_3$, 298 K): δ/ppm = 5.76–5.64 (m, 2H), 4.25–4.11 (m, 2H), 2.98–2.93 (m, 1H), 2.81–2.60 (m, 2H), 1.67 (t, *J* = 7.1 Hz, 3H), 1.50–1.19 (m, 8H), 1.13 (s, 9H), 0.86 (t, *J* = 7.0 Hz, 3H). $^{13}$**C{$^1$H} NMR** (100 MHz, $CDCl_3$, 298 K): δ/ppm = 173.0, 141.1, 127.8, 127.4, 124.2, 64.7, 39.5, 32.2, 31.6, 29.7 (3C), 28.5, 27.9, 25.9,

22.8, 22.7, 14.2. **HRMS** (APCI): calculated for $C_{18}H_{31}O_2^+$ [(M+H)$^+$]: 279.2319; found: 279.2321. **IR** (ATR): ṽ/cm$^{-1}$ = 3026, 2954, 2926, 2859, 2191, 1843, 1720, 1634, 1464, 1395, 1363, 1301, 1227, 1141, 1110, 1044, 969, 907, 786, 725, 664. **Optical Rotation**: $[\alpha]_D^{RT}$ = –0.70 (*c* 0.16, $CHCl_3$).

The enantiomeric ratio of **3am** was determined by HPLC analysis on a chiral stationary phase (*Daicel* Chiralpak ID column, column temperature 20 °C, mobile phase *n*-heptane:isopropanol = 99.8:0.2, flow rate 1.0 mL/min, λ = 250 nm): $t_R$ = 5.7 min (minor), $t_R$ = 6.2 min (major).

### 6.2.14 Hexyl (*R*)-2-butyl-6-methylcyclohexa-1,4-diene-1-carboxylate (3bb)

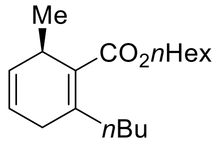


**3bb**
$C_{18}H_{30}O_2$
M = 278.44 g/mol

Prepared from **L2**·$CoBr_2$ (38 mg, 75 µmol, 5.0 mol %), Zn (49 mg, 0.75 mmol, 0.50 equiv), $NaBAr^F$ (133 mg, 150 µmol, 0.100 equiv), (*E*)-penta-1,3-diene (204 mg, 3.00 mmol, 2.00 equiv) and hexyl hept-2-ynoate (**2b**, 315 mg, 1.50 mmol, 1.00 equiv) according to **GP 7**. The residue was purified by FCC on silica gel using *n*-pentane:$Et_2O$ (98:2) as eluent. The resulting mixture was then purified by automatic reverse phase FCC using water:acetonitrile (33:67 → 0:100) to afford the product **3bb** (200 mg, 718 µmol, 44%, 92% ee) as a colorless oil.

**$R_f$** = 0.35 (*n*-pentane:$Et_2O$ = 98:2). **$^1$H NMR** (400 MHz, $CDCl_3$, 298 K): δ/ppm = 5.69–5.60 (m, 2H), 4.15 (t, *J* = 6.7 Hz, 2H), 3.23–3.19 (m, 1H), 2.83–2.63 (m, 2H), 2.26 (t, *J* = 7.5 Hz, 2H), 1.67 (pq, *J* = 7.0 Hz, 2H), 1.45–1.30 (m, 8H), 1.33–1.30 (m, 2H), 1.05 (d, *J* = 7.0 Hz, 3H), 0.92–0.88 (m, 6H). **$^{13}$C{$^1$H} NMR** (100 MHz, $CDCl_3$, 298 K): δ/ppm = 169.5, 142.8, 130.6, 128.8, 121.8, 64.4, 34.7, 32.3, 31.6, 31.5, 30.9, 28.8, 25.9, 23.2, 22.7, 21.7, 14.2 (2C). **HRMS** (APCI): calculated for $C_{18}H_{31}O_2^+$ [(M+H)$^+$]: 279.2319; found: 279.2321. **IR** (ATR): ṽ/cm$^{-1}$ = 3421, 3028, 2955, 2927, 2858, 2250, 2110, 1908, 1711, 1626, 1457, 1376, 1223, 1181, 1133, 1094, 1065, 1034, 984, 936, 909, 782, 707. **Optical Rotation**: $[\alpha]_D^{RT}$ = –0.57 (*c* 0.28, $CHCl_3$).

The enantiomeric ratio of **3bb** was determined by chiral GLC analysis (*Sigma-Aldrich* Astec Chiraldex B-DM column (30 m × 0.25 mm, 0.12 µm film thickness), column temperature 60 °C (30 min), ramp of 1 °C/min to 175 °C (10 min), ramp of 3 °C/min to 200 °C (10 min), flow rate isobaric 14 psi): $t_R$ = 132.4 min (major), $t_R$ = 133.2 min (minor).

### 6.2.15 Hexyl (*R*)-3-butyl-[1,1'-bi(cyclohexane)]-2,5-diene-2-carboxylate (3cb)

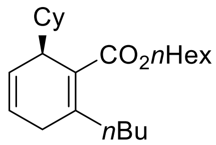


**3cb**
$C_{23}H_{38}O_2$
M = 346.56 g/mol

Prepared from **L2**·$CoBr_2$ (38 mg, 75 µmol, 5.0 mol %), Zn (49 mg, 0.75 mmol, 0.50 equiv), $NaBAr^F$ (133 mg, 150 µmol, 0.100 equiv), (*E*)-buta-1,3-dien-1-ylcyclohexane (**1c**, 409 mg, 3.00 mmol, 2.00 equiv) and hexyl hept-2-ynoate (**2b**, 315 mg, 1.50 mmol, 1.00 equiv) according to **GP 7**. The residue was purified by FCC on silica gel using *n*-pentane:$Et_2O$ (98:2) as eluent. The resulting mixture was then purified by automatic reverse phase FCC using water:acetonitrile (15:85 → 0:100) to afford the product **3cb** (214 mg, 618 µmol, 60%, 95% ee) as a colorless oil.

**$R_f$** = 0.31 (*n*-pentane:$Et_2O$ = 98:2). **$^1$H NMR** (400 MHz, $CDCl_3$, 298 K): δ/ppm = 5.77–5.69 (m, 2H), 4.14 (t, *J* = 6.3 Hz, 2H), 3.23–3.18 (m, 1H), 2.80–2.57 (m, 2H), 2.36–2.16 (m, 2H), 1.73–1.03 (m, 23H), 0.92–0.82 (m, 6H). **$^{13}$C{$^1$H} NMR** (100 MHz, $CDCl_3$, 298 K): δ/ppm = 169.9, 144.0, 127.0, 126.6, 124.2, 64.3, 43.2 (2C), 35.0, 32.1, 31.6, 31.0, 30.9, 28.8, 27.7, 27.1, 26.7 (2C), 26.0, 23.1, 22.7, 14.2 (2C). **HRMS** (APCI): calculated for $C_{23}H_{39}O_2^+$ [$(M+H)^+$]: 347.2945; found: 347.2942. **IR** (ATR): $\tilde{v}$/cm$^{-1}$ = 3421, 3030, 2922, 2852, 2663, 2331, 2120, 2001, 1710, 1634, 1449, 1378, 1342, 1296, 1223, 1087, 1039, 893, 804, 728. **Optical Rotation**: $[\alpha]_D^{RT}$ = –0.72 (*c* 0.26, $CHCl_3$).

The enantiomeric ratio of **3cb** was determined by HPLC analysis on a chiral stationary phase (*Daicel* Chiralcel OD-H column, column temperature 20 °C, mobile phase *n*-heptane, flow rate 1.0 mL/min, λ = 250 nm): $t_R$ = 7.7 min (major), $t_R$ = 11.4 min (minor).

# 7 Asymmetric Transfer Hydrogenation of Styrene Derivatives

## 7.1 General Procedure for the Asymmetric Transfer Hydrogenation of Styrene Derivatives (GP 8)

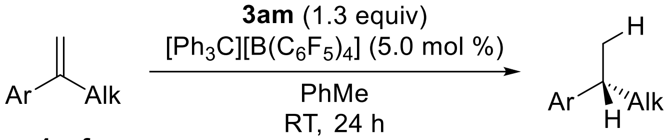


**4a**–**f** **5a**–**f**

According to a modified literature procedure[2] in a glovebox, the indicated alkene **4a**–**f** (0.10 mmol, 1.0 equiv) and the surrogate **3am** (0.13 mmol, 1.3 equiv) are dissolved in toluene (0.25 mL). A second solution of $[Ph_3C][B(C_6F_5)_4]$ (4.6 mg, 5.0 µmol, 5.0 mol %) in toluene (0.25 mL) is prepared and added to the first solution. After stirring at room temperature for 24 h, the vessel is transferred out of the glovebox, filtered through a plug of silica, rinsed with $CH_2Cl_2$ and all volatiles are removed under reduced pressure. The residue is purified by FCC on silica gel using *n*-pentane:$Et_2O$ mixtures as eluent to afford the products **5a–f**.

Racemic products are synthesized following the same procedure using *rac*-**3am**.

## 7.2 Characterization Data of the Enantioenriched Hydrogenation Products

### 7.2.1 (*R*)-1-(1-Cyclohexylethyl)-4-methoxybenzene (5a)

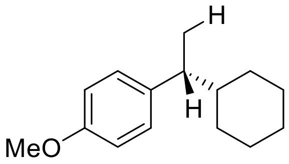


**5a**
$C_{15}H_{22}O$
M = 218.34 g/mol

Prepared from alkene **4a** (22 mg, 0.10 mmol, 1.0 equiv), surrogate **3am** (36 mg, 0.13 mmol, 1.3 equiv) and $[Ph_3C][B(C_6F_5)_4]$ (4.6 mg, 5.0 µmol, 5.0 mol %) according to **GP 8**. The residue was purified by FCC on silica gel using *n*-pentane:$Et_2O$ (100:0 → 80:1) as the eluent to afford the product **5a** (95% GLC yield, 80% ee) as a colorless oil.

**$^1$H NMR** (400 MHz, $CDCl_3$, 298 K): δ/ppm = 7.05 ($m_c$, 2H), 6.82 ($m_c$, 2H), 3.79 (s, 3H), 2.39 (pq, $J$ = 7.3 Hz, 1H), 1.88–1.82 (m, 1H), 1.76–1.70 (m, 1H), 1.65–1.57 (m, 2H), 1.47–1.41 (m, 1H), 1.39–1.29 (m, 1H), 1.25–1.05 (m, 3H), 1.20 (d, $J$ = 7.0 Hz, 3H), 0.97–0.86 (m, 1H), 0.84–0.74 (m, 1H). **$^{13}$C{$^1$H} NMR** (100 MHz, $CDCl_3$, 298 K): δ/ppm = 157.7, 139.3, 128.6 (2C), 113.5 (2C), 55.3, 45.2, 44.5, 31.5, 30.8, 26.7 (2C), 26.7, 19.1.

The NMR spectroscopic data are in accordance with those reported.[2]

The enantiomeric ratio of **5a** was determined by chiral GLC analysis (*Sigma-Aldrich* Astec Chiraldex B-DM column (30 m × 0.25 mm, 0.12 µm film thickness), column temperature 60 °C (30 min), ramp of 1 °C/min to 175 °C (10 min), ramp of 3 °C/min to 200 °C (10 min), flow rate isobaric 14 psi): $t_R$ = 117.6 min (minor), $t_R$ = 118.3 min (major).

### 7.2.2 (*R*)-(1-(4-Methoxyphenyl)ethyl)cycloheptane (5b)

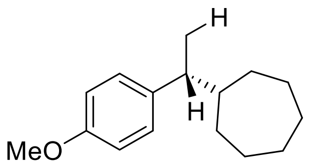


**5b**
$C_{16}H_{24}O$
M = 232.37 g/mol

Prepared from alkene **4b** (22 mg, 0.10 mmol, 1.0 equiv), surrogate **3am** (36 mg, 0.13 mmol, 1.3 equiv) and $[Ph_3C][B(C_6F_5)_4]$ (4.6 mg, 5.0 µmol, 5.0 mol %) according to **GP 8**. The residue was purified by FCC on silica gel using *n*-pentane:$Et_2O$ (60:1) as the eluent to afford the product **5b** (98% NMR yield, 76% ee) as a colorless oil.

**$^1$H NMR** (400 MHz, $CDCl_3$, 298 K): δ/ppm = 7.08 ($m_c$, 2H), 6.82 ($m_c$, 2H), 3.79 (s, 3H), 2.56 (pq, *J* = 7.0 Hz, 1H), 1.77–1.71 (m, 1H), 1.66–1.09 (m, 12H), 1.19 (d, *J* = 7.1 Hz, 3H). **$^{13}$C{$^1$H} NMR** (100 MHz, $CDCl_3$, 298 K): δ/ppm = 157.6, 139.5, 128.7 (2C), 113.5 (2C), 55.3, 45.8, 45.0, 32.6, 31.3, 28.6, 28.4, 27.0, 26.8, 18.6.

The NMR spectroscopic data are in accordance with those reported.[2]

The enantiomeric ratio of **5b** was determined by HPLC analysis on a chiral stationary phase (*Daicel* Chiralpak AD-H column, column temperature 20 °C, mobile phase *n*-heptane:isopropanol = 100:0, flow rate 0.6 mL/min, λ = 280 nm): $t_R$ = 10.4 min (minor), $t_R$ = 11.4 min (major).

### 7.2.3 (*R*)-1-(4,4-Dimethylpentan-2-yl)-4-methoxybenzene (5c)

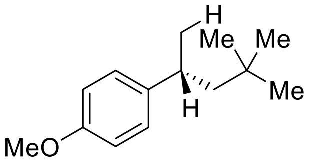


**5c**
$C_{14}H_{22}O$
M = 206.33 g/mol

Prepared from alkene **4c** (20 mg, 0.10 mmol, 1.0 equiv), surrogate **3am** (36 mg, 0.13 mmol, 1.3 equiv) and $[Ph_3C][B(C_6F_5)_4]$ (4.6 mg, 5.0 µmol, 5.0 mol %) according to **GP 8**. The residue was purified by FCC on silica gel using *n*-pentane:$Et_2O$ (97:3) as the eluent to afford the product **5c** (95% NMR yield, 40% ee) as a colorless oil.

**$^1$H NMR** (400 MHz, $CDCl_3$, 298 K): δ/ppm = 7.11 ($m_c$, 2H), 6.82 ($m_c$, 2H), 3.78 (s, 3H), 2.78 ($m_c$, 1H), 1.66 (dd, *J* = 14.0, 8.0 Hz, 1H), 1.45 (dd, *J* = 14.0, 4.5 Hz, 1H), 1.20 (d, *J* = 7.0 Hz, 3H), 0.80 (s, 9H). **$^{13}$C{$^1$H} NMR** (100 MHz, $CDCl_3$, 298 K): δ/ppm = 157.6, 141.9, 128.0 (2C), 113.8 (2C), 55.3, 52.3, 36.1, 31.4, 30.3 (3C), 26.2.

The NMR spectroscopic data are in accordance with those reported.[2]

The enantiomeric ratio of **5c** was determined by HPLC analysis on a chiral stationary phase (*Daicel* Chiralcel OJ-H column, column temperature 20 °C, mobile phase *n*-heptane:isopropanol = 100:0, flow rate 0.6 mL/min, λ = 230 nm): $t_R$ = 10.3 min (major), $t_R$ = 12.9 min (minor).

### 7.2.4 Methyl (*R*)-4-(4-methoxyphenyl)pentanoate (5d)

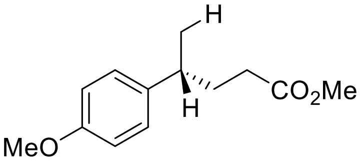


**5d**
$C_{13}H_{18}O_3$
M = 222.28 g/mol

Prepared from alkene **4d** (22 mg, 0.10 mmol, 1.0 equiv), surrogate **3am** (36 mg, 0.13 mmol, 1.3 equiv) and $[Ph_3C][B(C_6F_5)_4]$ (4.6 mg, 5.0 µmol, 5.0 mol %) according to **GP 8**. The residue was purified by FCC on silica gel using *n*-pentane:$Et_2O$ (92:8) as the eluent to afford the product **5d** (87% NMR yield, 27% ee) as a colorless oil.

**$^1$H NMR** (400 MHz, $CDCl_3$, 298 K): δ/ppm = 7.09 ($m_c$, 2H), 6.84 ($m_c$, 2H), 3.79 (s, 3H), 3.62 (s, 3H), 2.71–2.61 (m, 1H), 2.21–2.16 (m, 2H), 1.96–1.79 (m, 2H), 1.24 (d, *J* = 7.0 Hz, 3H).

**$^{13}C\{^{1}H\}$ NMR** (100 MHz, $CDCl_3$, 298 K): δ/ppm = 174.3, 158.1, 138.4, 128.0 (2C), 114.0 (2C), 55.4, 51.6, 38.7, 33.5, 32.5, 22.5.

The NMR spectroscopic data are in accordance with those reported.[2]

The enantiomeric ratio of **5d** was determined by HPLC analysis on a chiral stationary phase (*Daicel* Chiralpak IB column, column temperature 20 °C, mobile phase *n*-heptane:isopropanol = 99:1, flow rate 0.5 mL/min, λ = 230 nm): $t_R$ = 9.7 min (minor), $t_R$ = 10.2 min (major).

### 7.2.5 (*R*)-1-Methoxy-4-(1-phenylpropan-2-yl)benzene (5e)

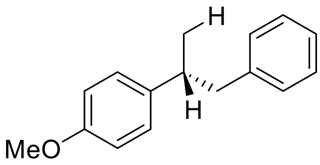


**5e**
$C_{16}H_{18}O$
M = 226.32 g/mol

Prepared from alkene **4e** (22 mg, 0.10 mmol, 1.0 equiv), surrogate **3am** (36 mg, 0.13 mmol, 1.3 equiv) and $[Ph_3C][B(C_6F_5)_4]$ (4.6 mg, 5.0 µmol, 5.0 mol %) according to **GP 8**. The residue was purified by FCC on silica gel using *n*-pentane:$Et_2O$ (95:5) as the eluent to afford the product **5e** (94% NMR yield, 37% ee) as a colorless oil.

**$^{1}H$ NMR** (400 MHz, $CDCl_3$, 298 K): δ/ppm = 7.26–7.21 (m, 2H), 7.18–7.16 (m, 1H), 7.12–7.06 (m, 4H), 6.83 ($m_c$, 2H), 3.79 (s, 3H), 2.99–2.88 (m, 2H), 2.7–2.72 (m, 1H), 1.22 (d, *J* = 6.8 Hz, 3H). **$^{13}C\{^{1}H\}$ NMR** (100 MHz, $CDCl_3$, 298 K): δ/ppm = 157.9, 141.0, 139.2, 129.3 (2C), 128.2 (2C), 128.0 (2C), 125.9, 113.8 (2C), 55.4, 45.4, 41.1, 21.5.

The NMR spectroscopic data are in accordance with those reported.[2]

The enantiomeric ratio of **4e** was determined by HPLC analysis on a chiral stationary phase (*Daicel* Chiralcel OJ-H column, column temperature 20 °C, mobile phase *n*-heptane:isopropanol = 95:5, flow rate 0.5 mL/min, λ = 280 nm): $t_R$ = 15.0 min (major), $t_R$ = 18.2 min (minor).

### 7.2.6 (*R*)-(1-cyclohexylethyl)benzene (5f)

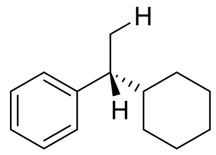

**5f**
$C_{14}H_{20}O$
M = 188.31 g/mol

Prepared from alkene **4f** (19 mg, 0.10 mmol, 1.0 equiv), surrogate **3am** (36 mg, 0.13 mmol, 1.3 equiv) and $[Ph_3C][B(C_6F_5)_4]$ (4.6 mg, 5.0 µmol, 5.0 mol %) according to **GP 8**. The residue was purified by FCC on silica gel using *n*-pentane as the eluent to afford the product **5f** (96% NMR yield, 75% ee) as a colorless oil.

**$^1$H NMR** (400 MHz, $CDCl_3$, 298 K): δ/ppm = 7.28–7.25 (m, 2H), 7.18–7.12 (m, 3H), 2.43 (pq, *J* = 7.2 Hz, 1H), 1.89–1.84 (m, 1H), 1.76–1.70 (m, 1H), 1.64–1.59 (m, 2H), 1.46–1.37 (m 2H), 1.28–1.21 (m, 1H), 1.22 (d, *J* = 7.0 Hz, 3H), 1.14–1.07 (m, 2H), 0.98–0.76 (m, 2H). **$^{13}$C{$^1$H} NMR** (100 MHz, $CDCl_3$, 298 K): δ/ppm = 147.3, 128.1 (2C), 127.9 (2C), 125.8, 46.1, 44.3, 31.6, 30.8, 26.7 (2C), 26.7, 19.0.

The NMR spectroscopic data are in accordance with those reported.[2]

The enantiomeric ratio of **5f** was determined by HPLC analysis on a chiral stationary phase (*Daicel* Chiralcel OJ-H column, column temperature 20 °C, mobile phase *n*-heptane:isopropanol = 100:0, flow rate 0.6 mL/min, λ = 210 nm): $t_R$ = 8.3 min (major), $t_R$ = 9.1min (minor).

# 8 GLC and HPLC Traces

## 8.1 GLC Traces

### 8.1.1 Ethyl (*R*)-6-pentyl-2-methylcyclohexa-1,4-diene-1-carboxylate (3ag)

**Figure S1.** *rac*-Ethyl-6-pentyl-2-methylcyclohexa-1,4-diene-1-carboxylate

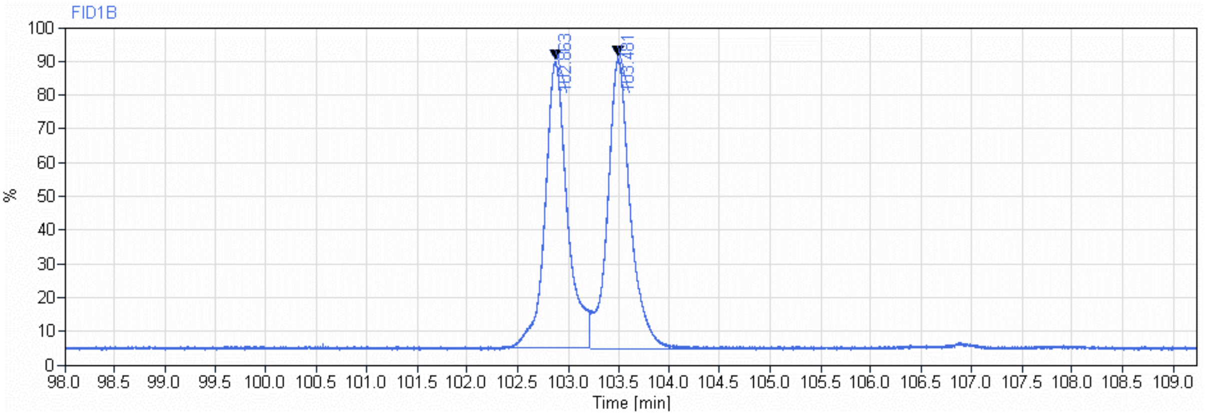


| Signal: | FID1B | | | |
|---|---|---|---|---|
| **RT [min]** | **Width [min]** | **Area** | **Height** | **Area%** |
| 102.863 | 1.10 | 85.02 | 5.50 | 49.17 |
| 103.481 | 2.16 | 87.88 | 5.57 | 50.83 |

**Figure S2.** Ethyl (*R*)-6-pentyl-2-methylcyclohexa-1,4-diene-1-carboxylate

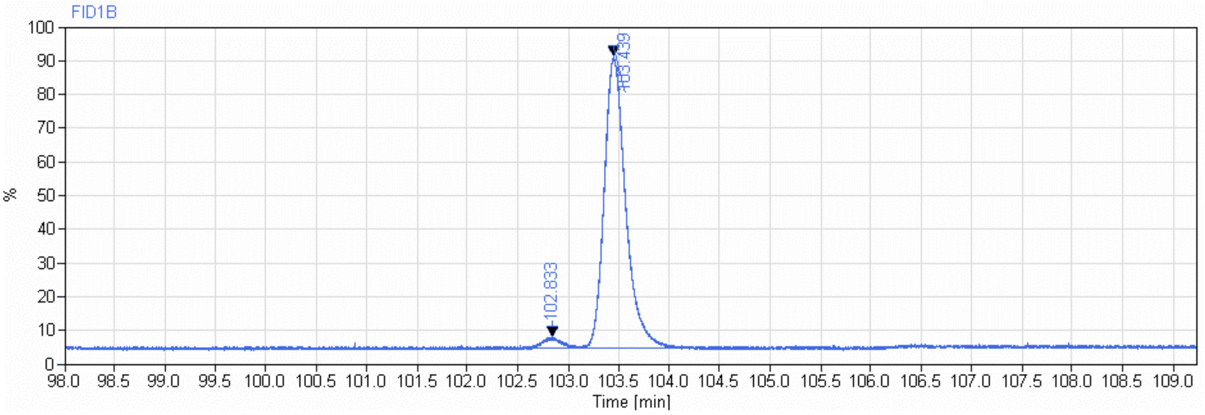


| Signal: | FID1B | | | |
|---|---|---|---|---|
| **RT [min]** | **Width [min]** | **Area** | **Height** | **Area%** |
| 102.833 | 0.57 | 2.72 | 0.19 | 3.30 |
| 103.439 | 0.99 | 79.63 | 5.36 | 96.70 |

### 8.1.2 Hexyl (*R*)-2-butyl-6-methylcyclohexa-1,4-diene-1-carboxylate (3bb)

**Figure S3.** *rac*-Hexyl-2-butyl-6-methylcyclohexa-1,4-diene-1-carboxylate

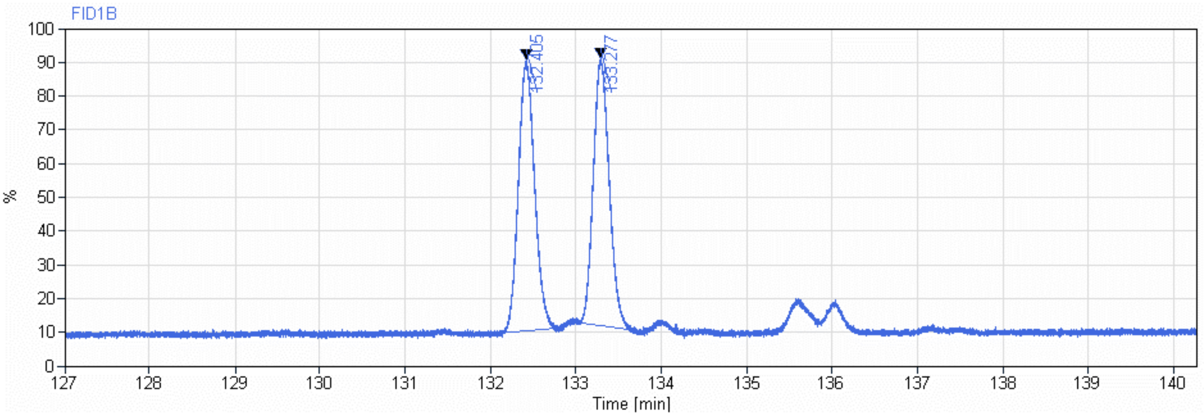


**Signal:** FID1B

| RT [min] | Width [min] | Area | Height | Area% |
|---|---|---|---|---|
| 132.405 | 0.80 | 33.11 | 2.50 | 50.79 |
| 133.277 | 0.72 | 32.08 | 2.46 | 49.21 |

**Figure S4.** Hexyl (*R*)-2-butyl-6-methylcyclohexa-1,4-diene-1-carboxylate

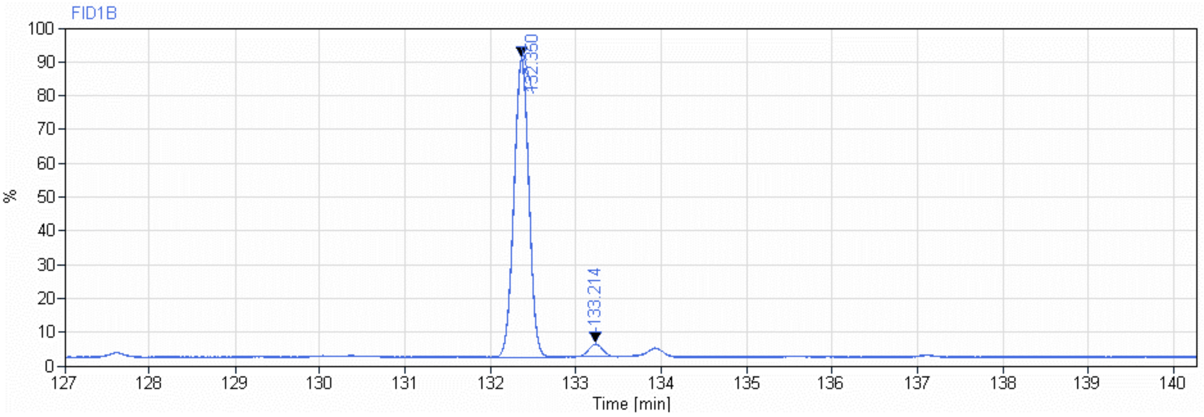


**Signal:** FID1B

| RT [min] | Width [min] | Area | Height | Area% |
|---|---|---|---|---|
| 132.350 | 0.84 | 275.17 | 22.63 | 96.22 |
| 133.214 | 0.71 | 10.81 | 0.96 | 3.78 |

### 8.1.3 (*R*)-1-(1-cyclohexylethyl)-4-methoxybenzene (5a)

**Figure S5.** *rac*-1-(1-cyclohexylethyl)-4-methoxybenzene

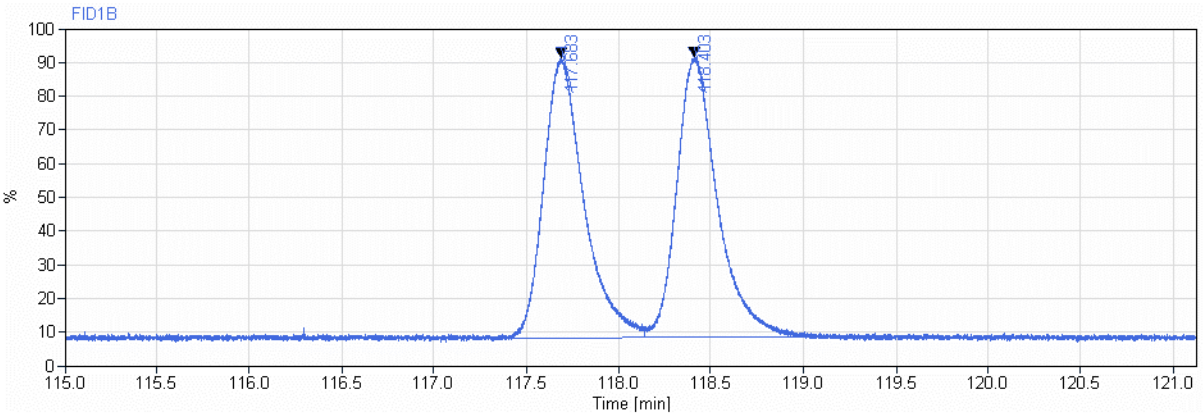


| Signal: | FID1B | | | |
|---|---|---|---|---|
| **RT [min]** | **Width [min]** | **Area** | **Height** | **Area%** |
| 117.683 | 1.07 | 40.36 | 2.65 | 49.63 |
| 118.403 | 1.62 | 40.96 | 2.65 | 50.37 |

**Figure S6.** (*R*)-1-(1-cyclohexylethyl)-4-methoxybenzene

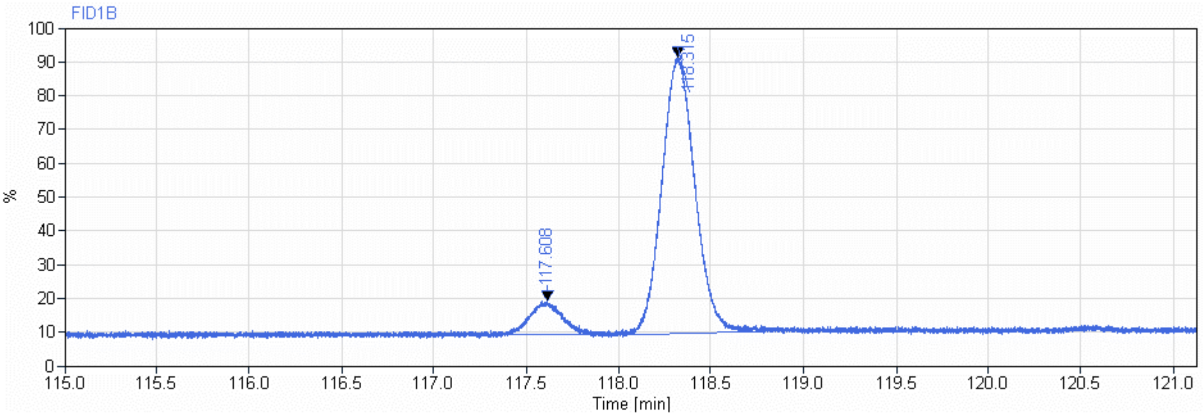


| Signal: | FID1B | | | |
|---|---|---|---|---|
| **RT [min]** | **Width [min]** | **Area** | **Height** | **Area%** |
| 117.608 | 0.56 | 3.50 | 0.28 | 10.15 |
| 118.315 | 0.87 | 31.02 | 2.46 | 89.85 |

## 8.2 HPLC Traces

### 8.2.1 Methyl (*R*)-6-pentyl-2-methylcyclohexa-1,4-diene-1-carboxylate (3aa)

**Figure S7.** *rac*-Methyl-6-pentyl-2-methylcyclohexa-1,4-diene-1-carboxylate

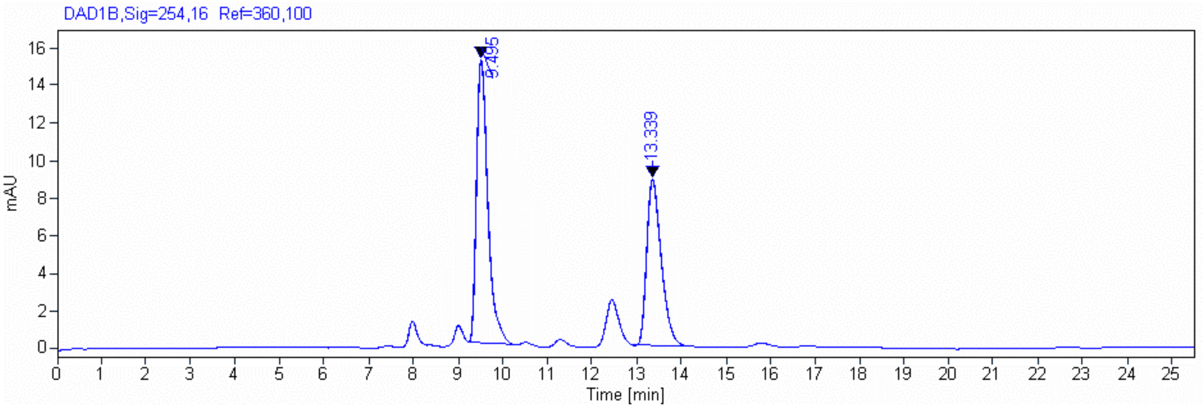


| Signal: | DAD1B,Sig=254,16 Ref=360,100 | | | |
|---|---|---|---|---|
| **RT [min]** | **Width [min]** | **Area** | **Height** | **Area%** |
| 9.495 | 0.99 | 265.32 | 15.07 | 57.24 |
| 13.339 | 1.15 | 198.22 | 8.85 | 42.76 |

**Figure S8.** Methyl (*R*)-6-pentyl-2-methylcyclohexa-1,4-diene-1-carboxylate

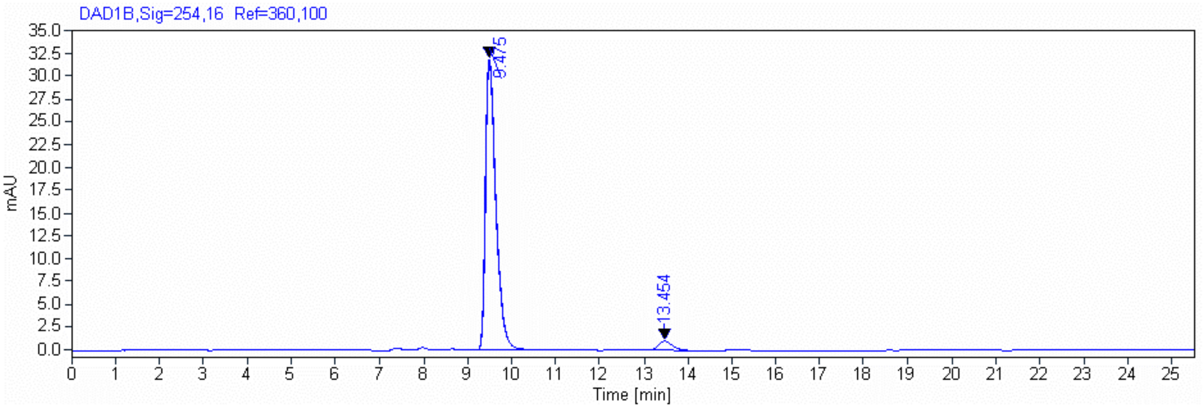


| Signal: | DAD1B,Sig=254,16 Ref=360,100 | | | |
|---|---|---|---|---|
| **RT [min]** | **Width [min]** | **Area** | **Height** | **Area%** |
| 9.475 | 1.27 | 521.55 | 31.86 | 95.92 |
| 13.454 | 1.24 | 22.16 | 1.01 | 4.08 |

### 8.2.2 Hexyl (*R*)-6-pentyl-2-methylcyclohexa-1,4-diene-1-carboxylate (3ab)

**Figure S9.** *rac*-Hexyl-6-pentyl-2-methylcyclohexa-1,4-diene-1-carboxylate

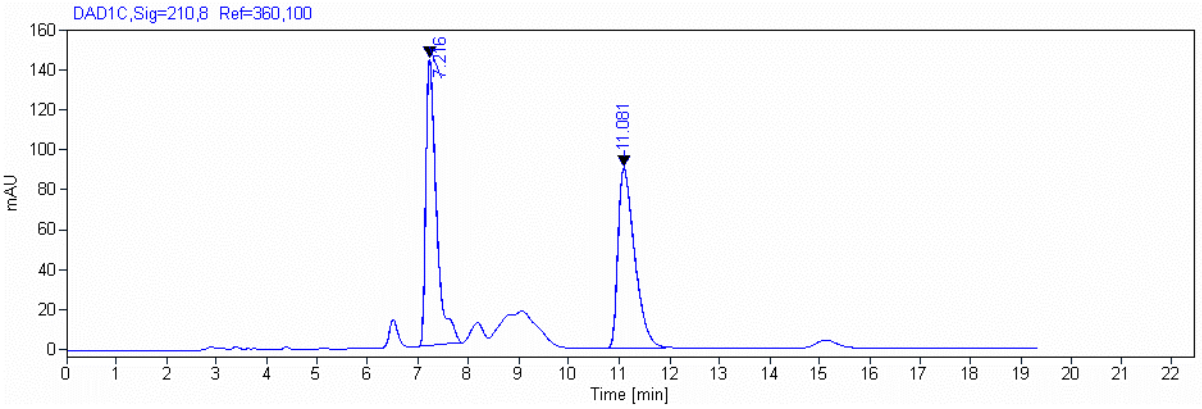


| Signal: | DAD1C,Sig=210,8 Ref=360,100 | | | |
|---|---|---|---|---|
| **RT [min]** | **Width [min]** | **Area** | **Height** | **Area%** |
| 7.216 | 0.83 | 2100.50 | 143.07 | 50.37 |
| 11.081 | 1.29 | 2069.30 | 90.05 | 49.63 |

**Figure S10.** Hexyl (*R*)-6-pentyl-2-methylcyclohexa-1,4-diene-1-carboxylate

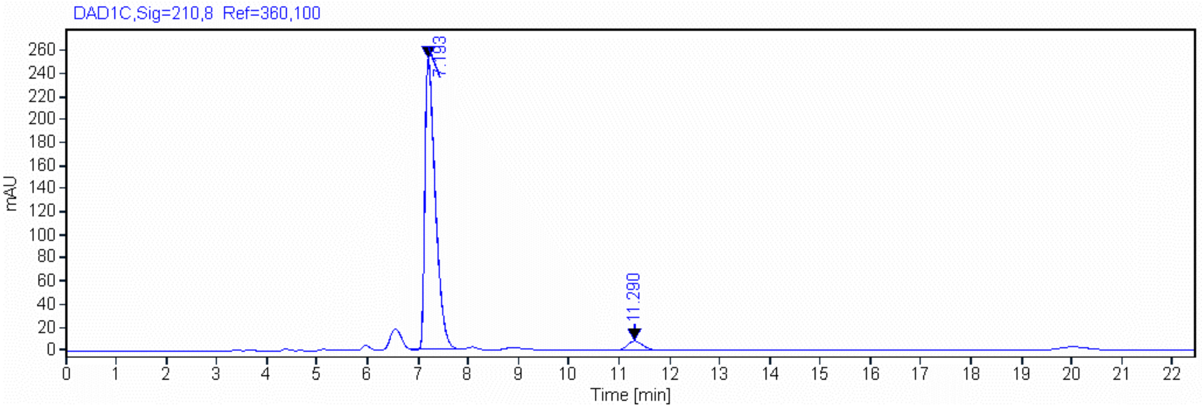


| Signal: | DAD1C,Sig=210,8 Ref=360,100 | | | |
|---|---|---|---|---|
| **RT [min]** | **Width [min]** | **Area** | **Height** | **Area%** |
| 7.193 | 1.00 | 3639.96 | 251.64 | 95.32 |
| 11.290 | 1.61 | 178.61 | 8.12 | 4.68 |

### 8.2.3 Isopropyl (*R*)-6-pentyl-2-methylcyclohexa-1,4-diene-1-carboxylate (3ac)

**Figure S11.** *rac*-Isopropyl-6-pentyl-2-methylcyclohexa-1,4-diene-1-carboxylate

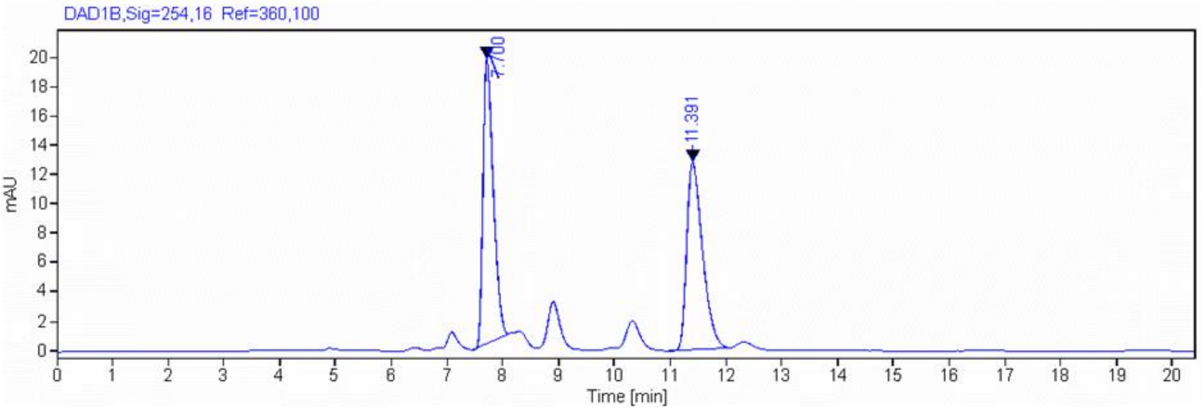


| Signal: | DAD1B,Sig=254,16 Ref=360,100 | | | |
|---|---|---|---|---|
| **RT [min]** | **Width [min]** | **Area** | **Height** | **Area%** |
| 7.700 | 0.70 | 249.30 | 19.40 | 49.82 |
| 11.391 | 1.12 | 251.08 | 12.79 | 50.18 |

**Figure S12.** Isopropyl (*R*)-6-pentyl-2-methylcyclohexa-1,4-diene-1-carboxylate

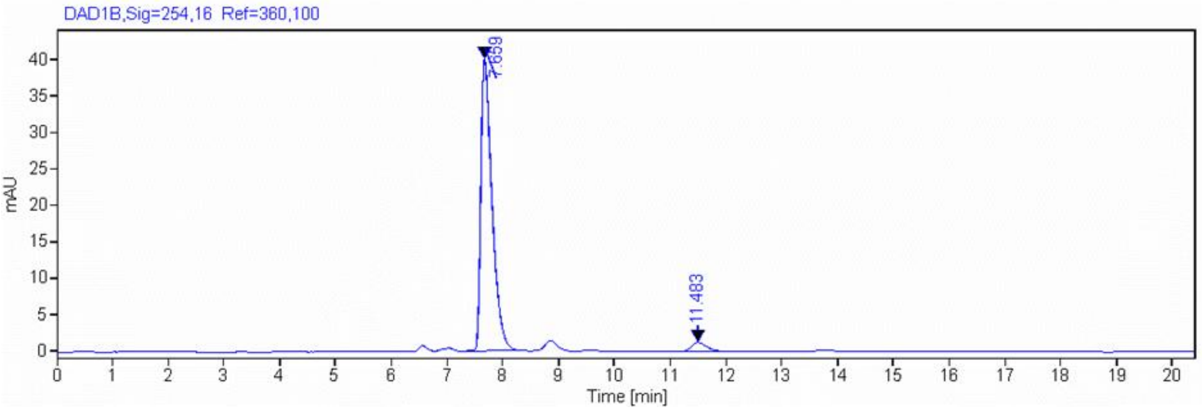


| Signal: | DAD1B,Sig=254,16 Ref=360,100 | | | |
|---|---|---|---|---|
| **RT [min]** | **Width [min]** | **Area** | **Height** | **Area%** |
| 7.659 | 1.00 | 558.80 | 40.05 | 95.81 |
| 11.483 | 1.33 | 24.44 | 1.25 | 4.19 |

### 8.2.4 Phenyl (*R*)-6-pentyl-2-methylcyclohexa-1,4-diene-1-carboxylate (3ad)

**Figure S13.** *rac*-Phenyl-6-pentyl-2-methylcyclohexa-1,4-diene-1-carboxylate

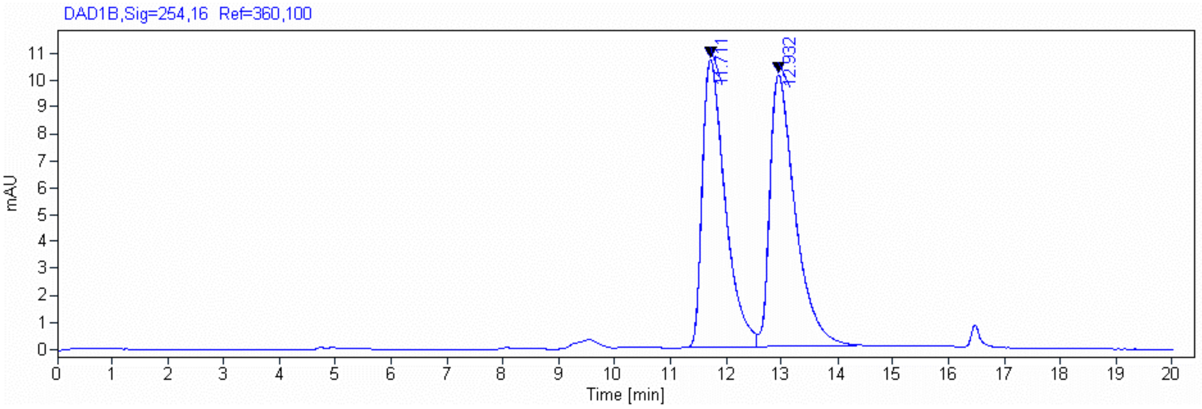


| **Signal:** | DAD1B,Sig=254,16 Ref=360,100 | | | |
|---|---|---|---|---|
| **RT [min]** | **Width [min]** | **Area** | **Height** | **Area%** |
| 11.711 | 1.34 | 305.31 | 10.72 | 48.79 |
| 12.932 | 1.89 | 320.42 | 10.12 | 51.21 |

**Figure S14.** Phenyl (*R*)-6-pentyl-2-methylcyclohexa-1,4-diene-1-carboxylate

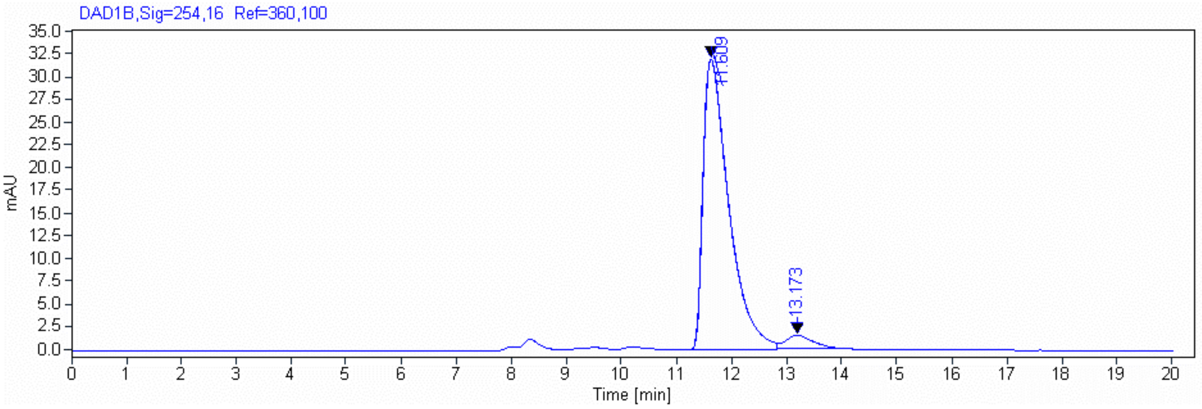


| **Signal:** | DAD1B,Sig=254,16 Ref=360,100 | | | |
|---|---|---|---|---|
| **RT [min]** | **Width [min]** | **Area** | **Height** | **Area%** |
| 11.609 | 1.60 | 1036.99 | 31.97 | 95.18 |
| 13.173 | 1.16 | 52.54 | 1.51 | 4.82 |

### 8.2.5 Cyclopentyl (*R*)-6-pentyl-2-methylcyclohexa-1,4-diene-1-carboxylate (3ae)

**Figure S15.** *rac*-Cyclopentyl-6-pentyl-2-methylcyclohexa-1,4-diene-1-carboxylate

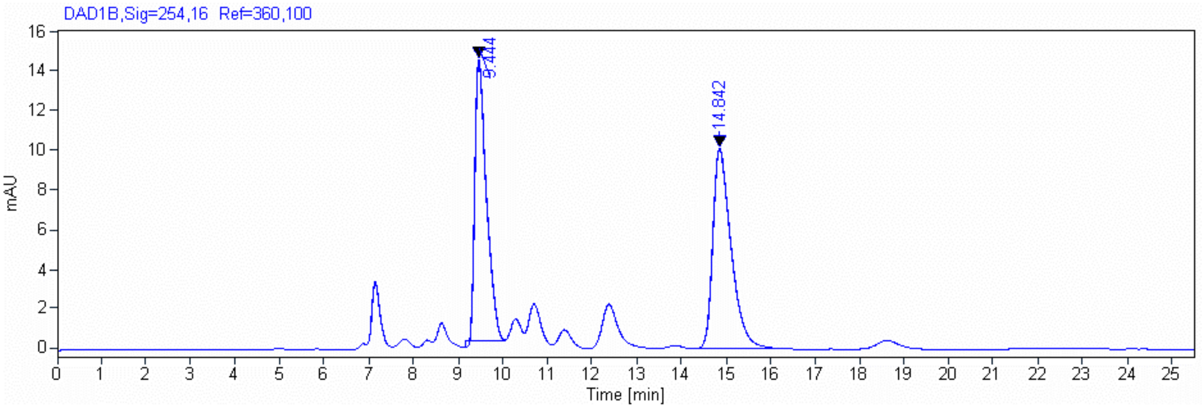


| Signal: | DAD1B,Sig=254,16 Ref=360,100 | | | |
|---|---|---|---|---|
| **RT [min]** | **Width [min]** | **Area** | **Height** | **Area%** |
| 9.444 | 0.88 | 254.13 | 14.20 | 47.18 |
| 14.842 | 1.67 | 284.52 | 10.09 | 52.82 |

**Figure S16.** Cyclopentyl (*R*)-6-pentyl-2-methylcyclohexa-1,4-diene-1-carboxylate

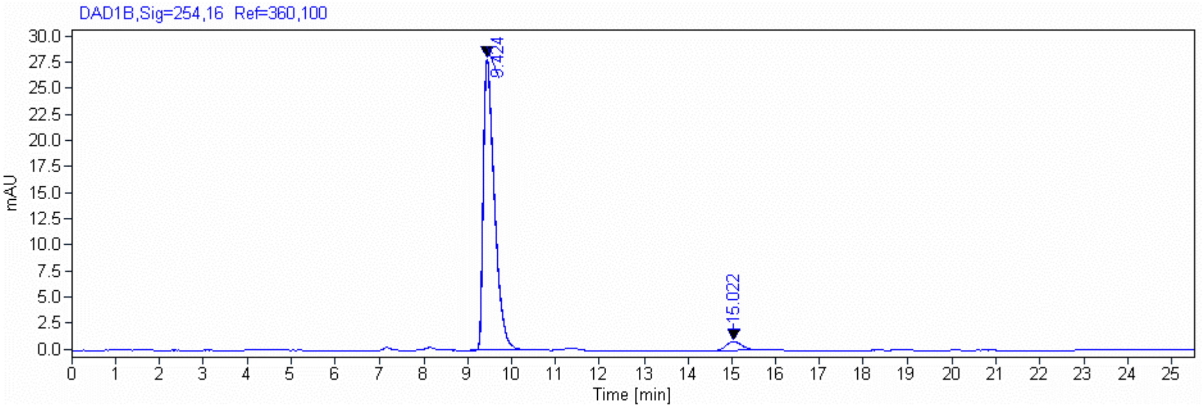


| Signal: | DAD1B,Sig=254,16 Ref=360,100 | | | |
|---|---|---|---|---|
| **RT [min]** | **Width [min]** | **Area** | **Height** | **Area%** |
| 9.424 | 1.49 | 488.58 | 27.93 | 96.02 |
| 15.022 | 1.15 | 20.23 | 0.83 | 3.98 |

### 8.2.6 Neopentyl (*R*)-6-pentyl-2-methylcyclohexa-1,4-diene-1-carboxylate (3af)

**Figure S17.** *rac*-Neopentyl-6-pentyl-2-methylcyclohexa-1,4-diene-1-carboxylate

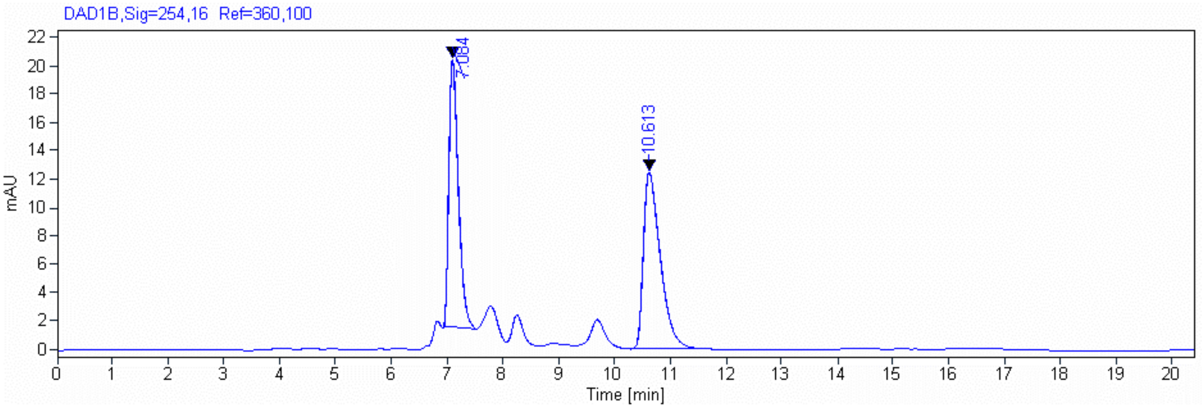


| **Signal:** | DAD1B,Sig=254,16 Ref=360,100 | | | |
|---|---|---|---|---|
| **RT [min]** | **Width [min]** | **Area** | **Height** | **Area%** |
| 7.084 | 0.58 | 222.95 | 18.88 | 45.85 |
| 10.613 | 1.52 | 263.34 | 12.48 | 54.15 |

**Figure S18.** Neopentyl (*R*)-6-pentyl-2-methylcyclohexa-1,4-diene-1-carboxylate

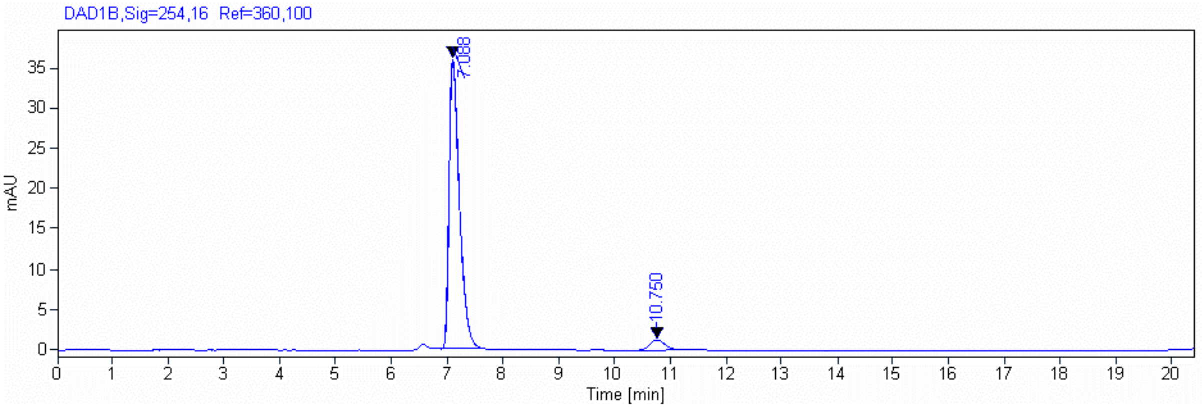


| **Signal:** | DAD1B,Sig=254,16 Ref=360,100 | | | |
|---|---|---|---|---|
| **RT [min]** | **Width [min]** | **Area** | **Height** | **Area%** |
| 7.088 | 0.82 | 465.18 | 36.09 | 95.04 |
| 10.750 | 0.95 | 24.27 | 1.29 | 4.96 |

### 8.2.7 Ethyl (*R*)-2-cyclopropyl-6-pentylcyclohexa-1,4-diene-1-carboxylate (3ah)

**Figure S19.** *rac*-Ethyl-2-cyclopropyl-6-pentylcyclohexa-1,4-diene-1-carboxylate

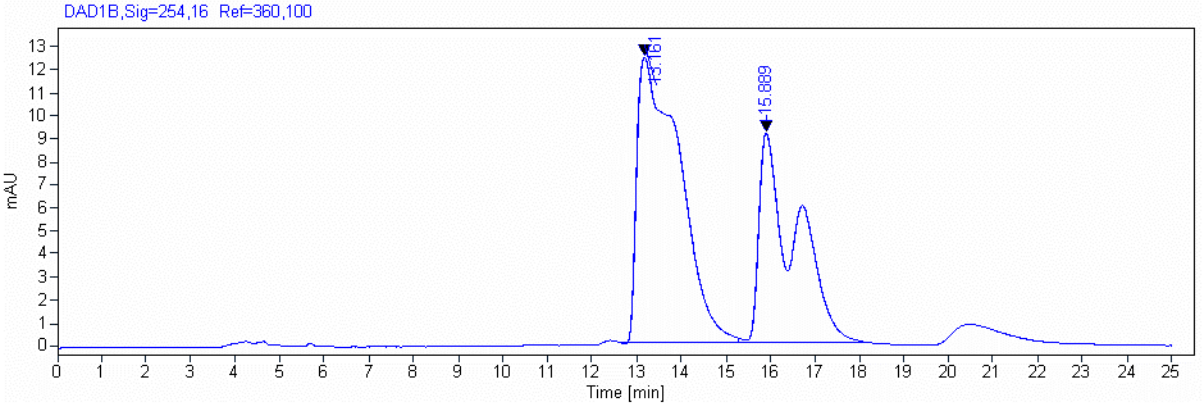


| Signal: | DAD1B,Sig=254,16 Ref=360,100 | | | |
|---|---|---|---|---|
| **RT [min]** | **Width [min]** | **Area** | **Height** | **Area%** |
| 13.161 | 2.69 | 818.58 | 12.38 | 61.62 |
| 15.889 | 2.83 | 509.91 | 9.08 | 38.38 |

**Figure S20.** Ethyl (*R*)-2-cyclopropyl-6-pentylcyclohexa-1,4-diene-1-carboxylate

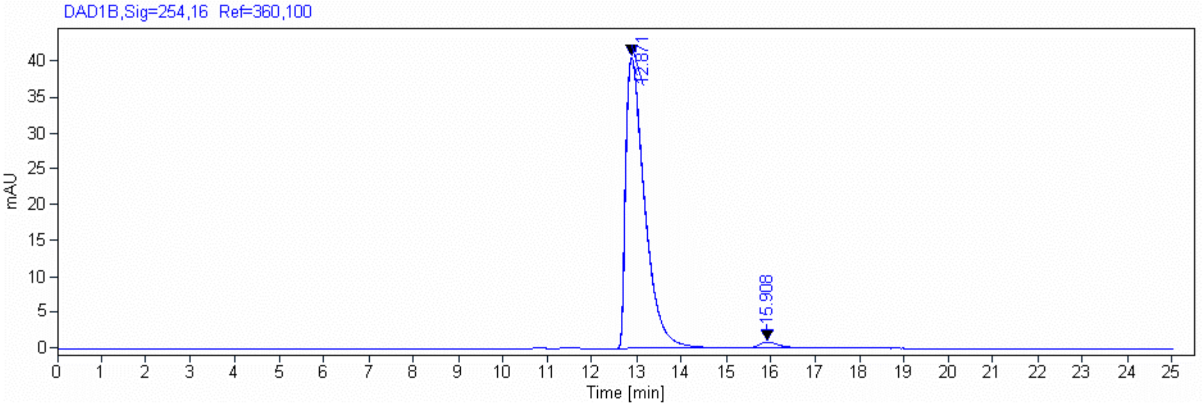


| Signal: | DAD1B,Sig=254,16 Ref=360,100 | | | |
|---|---|---|---|---|
| **RT [min]** | **Width [min]** | **Area** | **Height** | **Area%** |
| 12.871 | 2.47 | 1194.67 | 40.63 | 98.00 |
| 15.908 | 1.59 | 24.42 | 0.80 | 2.00 |

### 8.2.8 Ethyl (*R*)-6-pentyl-2-(trimethylsilyl)cyclohexa-1,4-diene-1-carboxylate (3ai)

**Figure S21.** Ethyl-6-pentyl-2-(trimethylsilyl)cyclohexa-1,4-diene-1-carboxylate

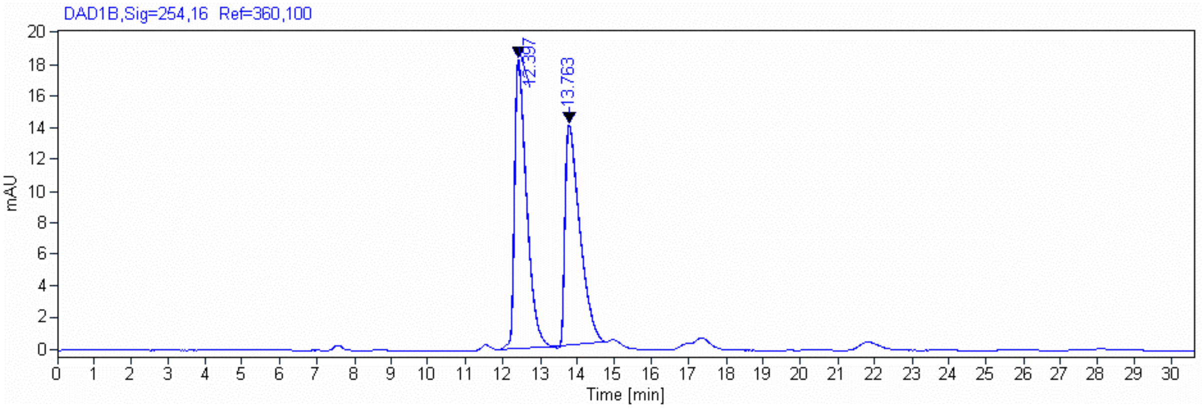


| Signal: | DAD1B,Sig=254,16 Ref=360,100 | | | |
|---|---|---|---|---|
| **RT [min]** | **Width [min]** | **Area** | **Height** | **Area%** |
| 12.397 | 1.40 | 410.69 | 18.26 | 51.81 |
| 13.763 | 1.43 | 382.05 | 13.91 | 48.19 |

**Figure S22.** Ethyl (*R*)-6-pentyl-2-(trimethylsilyl)cyclohexa-1,4-diene-1-carboxylate

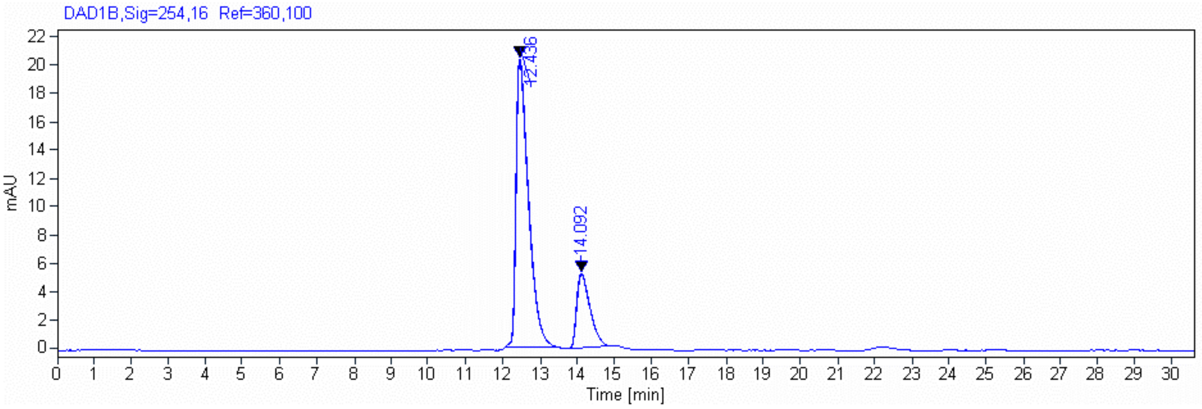


| Signal: | DAD1B,Sig=254,16 Ref=360,100 | | | |
|---|---|---|---|---|
| **RT [min]** | **Width [min]** | **Area** | **Height** | **Area%** |
| 12.436 | 1.42 | 473.37 | 20.33 | 78.46 |
| 14.092 | 1.20 | 129.99 | 5.24 | 21.54 |

### 8.2.9 Ethyl (*R*)-6-pentyl-2-isopropylcyclohexa-1,4-diene-1-carboxylate (3aj)

**Figure S23.** *rac*-Ethyl-6-pentyl-2-isopropylcyclohexa-1,4-diene-1-carboxylate

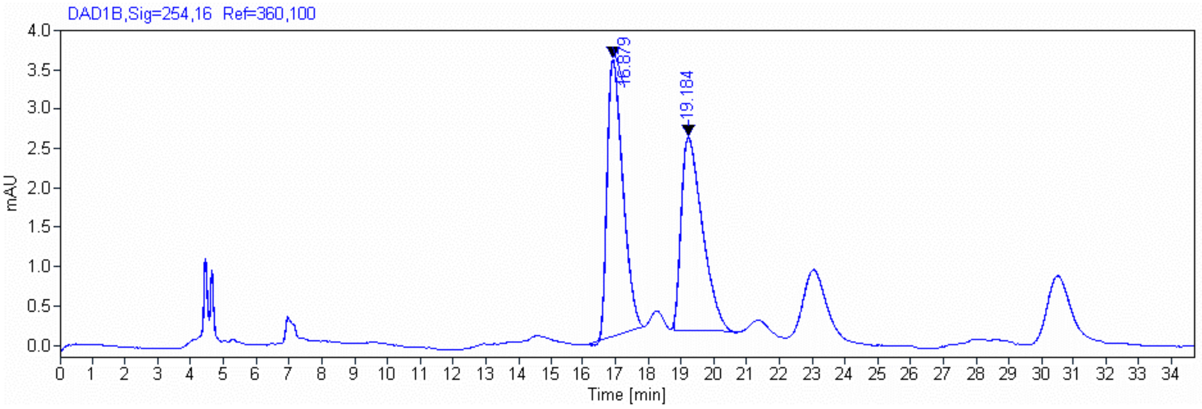


| Signal: | DAD1B,Sig=254,16 Ref=360,100 | | | |
|---|---|---|---|---|
| **RT [min]** | **Width [min]** | **Area** | **Height** | **Area%** |
| 16.879 | 1.83 | 116.70 | 3.51 | 51.32 |
| 19.184 | 2.03 | 110.68 | 2.45 | 48.68 |

**Figure S24.** Ethyl (*R*)-6-pentyl-2-isopropylcyclohexa-1,4-diene-1-carboxylate

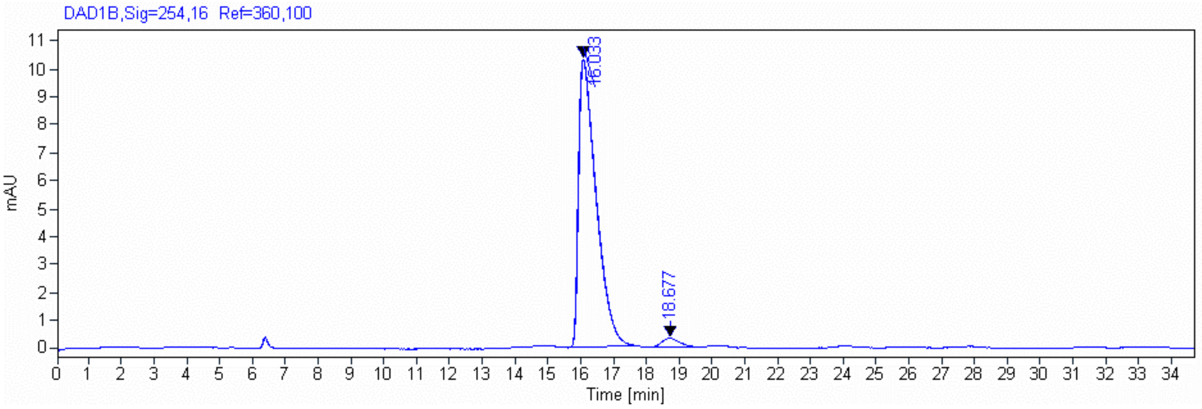


| Signal: | DAD1B,Sig=254,16 Ref=360,100 | | | |
|---|---|---|---|---|
| **RT [min]** | **Width [min]** | **Area** | **Height** | **Area%** |
| 16.033 | 2.20 | 383.10 | 10.31 | 96.97 |
| 18.677 | 1.51 | 11.96 | 0.32 | 3.03 |

### 8.2.10 Ethyl (*R*)-6-pentyl-2-(pentan-3-yl)cyclohexa-1,4-diene-1-carboxylate (3ak)

**Figure S25.** *rac*-Ethyl-6-pentyl-2-(pentan-3-yl)cyclohexa-1,4-diene-1-carboxylate

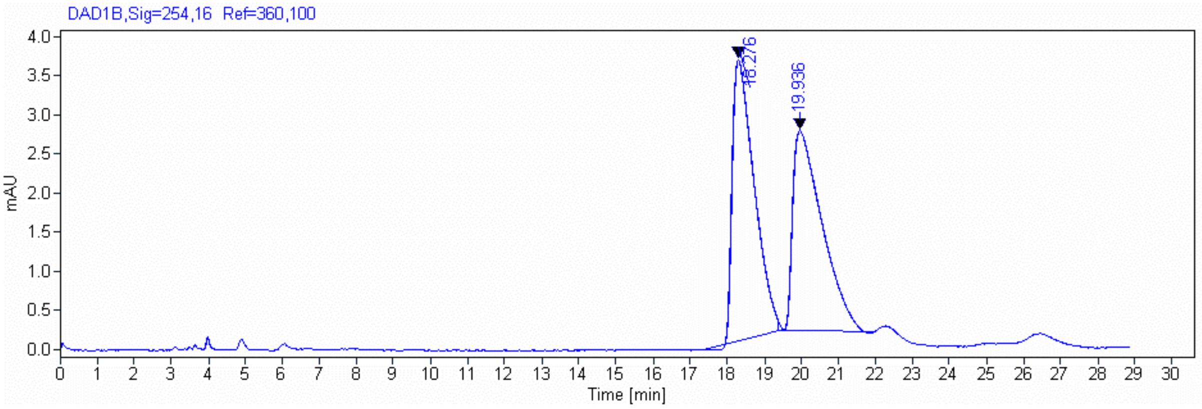


| Signal: | DAD1B,Sig=254,16 Ref=360,100 | | | |
|---|---|---|---|---|
| **RT [min]** | **Width [min]** | **Area** | **Height** | **Area%** |
| 18.276 | 1.97 | 147.00 | 3.61 | 50.95 |
| 19.936 | 2.63 | 141.55 | 2.57 | 49.05 |

**Figure S26.** Ethyl (*R*)-6-pentyl-2-(pentan-3-yl)cyclohexa-1,4-diene-1-carboxylate

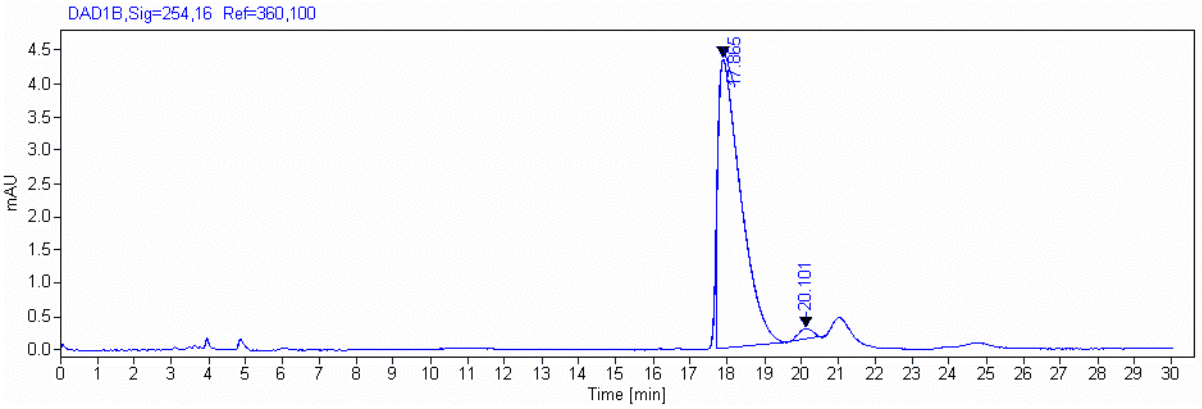


| Signal: | DAD1B,Sig=254,16 Ref=360,100 | | | |
|---|---|---|---|---|
| **RT [min]** | **Width [min]** | **Area** | **Height** | **Area%** |
| 17.865 | 1.84 | 183.98 | 4.36 | 97.97 |
| 20.101 | 1.13 | 3.81 | 0.15 | 2.03 |

### 8.2.11 Ethyl (*R*)-3-pentyl-[1,1'-bi(cyclohexane)]-1,4-diene-2-carboxylate (3al)

**Figure S27.** *rac*-Ethyl-3-pentyl-[1,1'-bi(cyclohexane)]-1,4-diene-2-carboxylate

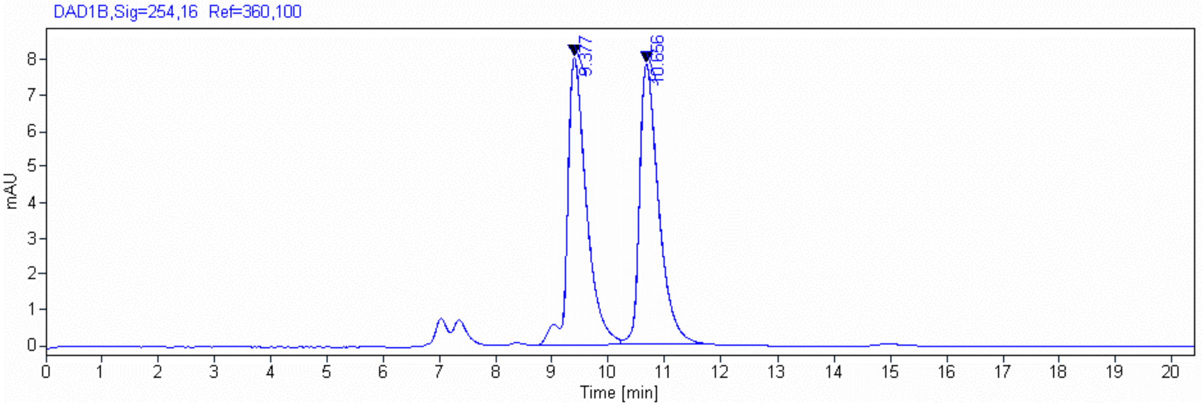


| **Signal:** | DAD1B,Sig=254,16 Ref=360,100 | | | |
|---|---|---|---|---|
| **RT [min]** | **Width [min]** | **Area** | **Height** | **Area%** |
| 9.377 | 1.45 | 187.18 | 8.03 | 50.25 |
| 10.656 | 1.55 | 185.32 | 7.83 | 49.75 |

**Figure S28.** Ethyl (*R*)-3-pentyl-[1,1'-bi(cyclohexane)]-1,4-diene-2-carboxylate

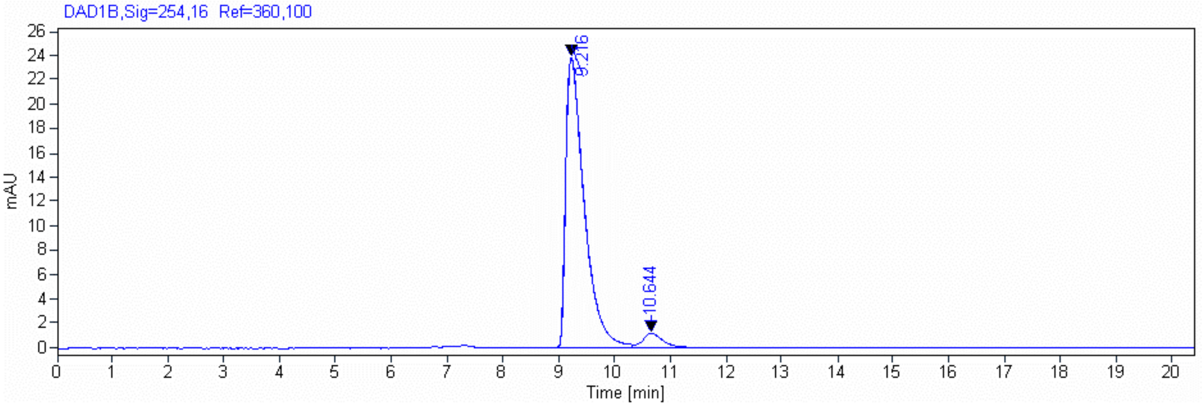


| **Signal:** | DAD1B,Sig=254,16 Ref=360,100 | | | |
|---|---|---|---|---|
| **RT [min]** | **Width [min]** | **Area** | **Height** | **Area%** |
| 9.216 | 1.65 | 553.32 | 23.89 | 94.64 |
| 10.644 | 1.47 | 31.33 | 1.20 | 5.36 |

### 8.2.12 Ethyl (*R*)-2-(tert-butyl)-6-pentylcyclohexa-1,4-diene-1-carboxylate (3am)

**Figure S29.** *rac*-Ethyl-2-(tert-butyl)-6-pentylcyclohexa-1,4-diene-1-carboxylate

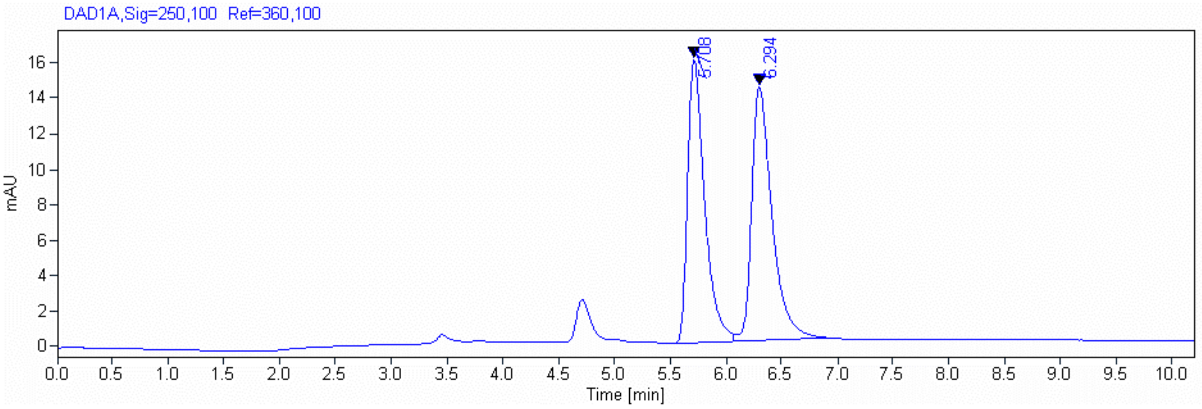


| Signal: | DAD1A,Sig=250,100 Ref=360,100 | | | |
|---|---|---|---|---|
| **RT [min]** | **Width [min]** | **Area** | **Height** | **Area%** |
| 5.708 | 0.57 | 169.77 | 15.97 | 48.36 |
| 6.294 | 0.85 | 181.26 | 14.29 | 51.64 |

**Figure S30.** Ethyl (*R*)-2-(tert-butyl)-6-pentylcyclohexa-1,4-diene-1-carboxylate

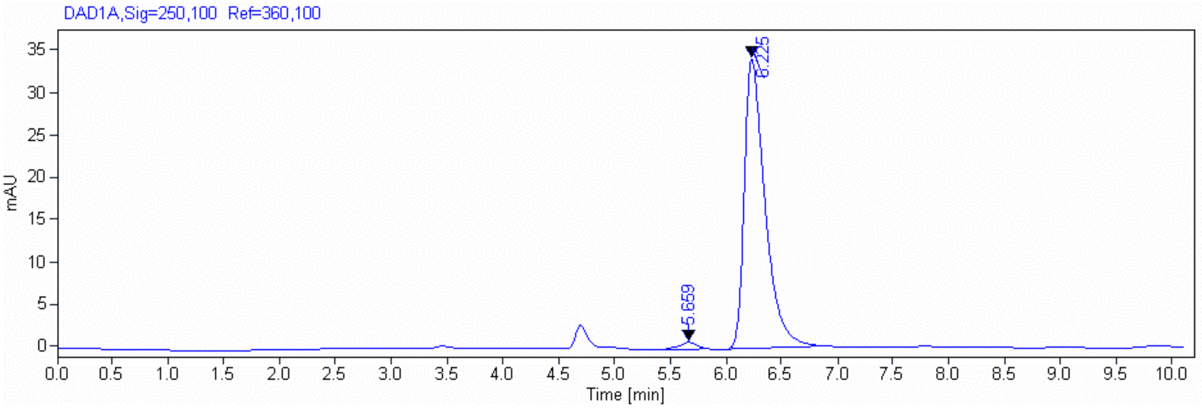


| Signal: | DAD1A,Sig=250,100 Ref=360,100 | | | |
|---|---|---|---|---|
| **RT [min]** | **Width [min]** | **Area** | **Height** | **Area%** |
| 5.659 | 0.52 | 8.38 | 0.78 | 1.86 |
| 6.225 | 0.93 | 443.15 | 34.30 | 98.14 |

### 8.2.13 Hexyl (*R*)-3-butyl-[1,1'-bi(cyclohexane)]-2,5-diene-2-carboxylate (3cb)

**Figure S31.** *rac*-Hexyl-3-butyl-[1,1'-bi(cyclohexane)]-2,5-diene-2-carboxylate

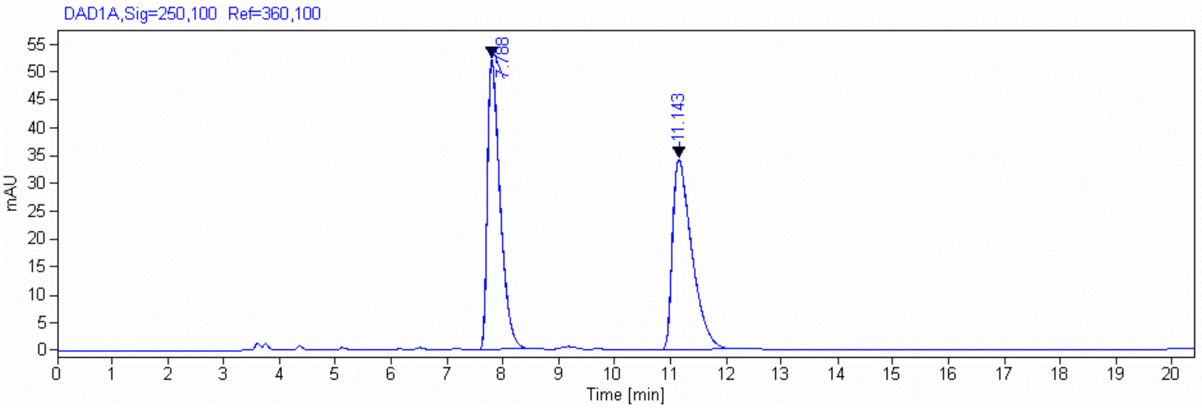


| Signal: | DAD1A,Sig=250,100 Ref=360,100 | | | |
|---|---|---|---|---|
| **RT [min]** | **Width [min]** | **Area** | **Height** | **Area%** |
| 7.788 | 0.98 | 823.59 | 52.17 | 50.03 |
| 11.143 | 1.69 | 822.48 | 34.23 | 49.97 |

**Figure S32.** Hexyl (*R*)-3-butyl-[1,1'-bi(cyclohexane)]-2,5-diene-2-carboxylate

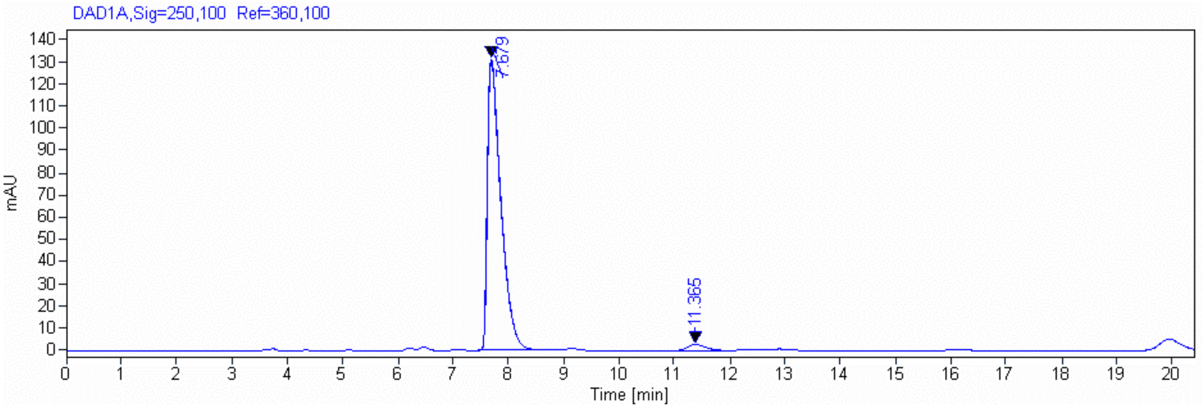


| Signal: | DAD1A,Sig=250,100 Ref=360,100 | | | |
|---|---|---|---|---|
| **RT [min]** | **Width [min]** | **Area** | **Height** | **Area%** |
| 7.679 | 1.27 | 2213.82 | 131.31 | 97.41 |
| 11.365 | 1.42 | 58.76 | 2.75 | 2.59 |

### 8.2.14 (*R*)-(1-(4-methoxyphenyl)ethyl)cycloheptane (5b)

**Figure S33.** *rac*-(1-(4-methoxyphenyl)ethyl)cycloheptane

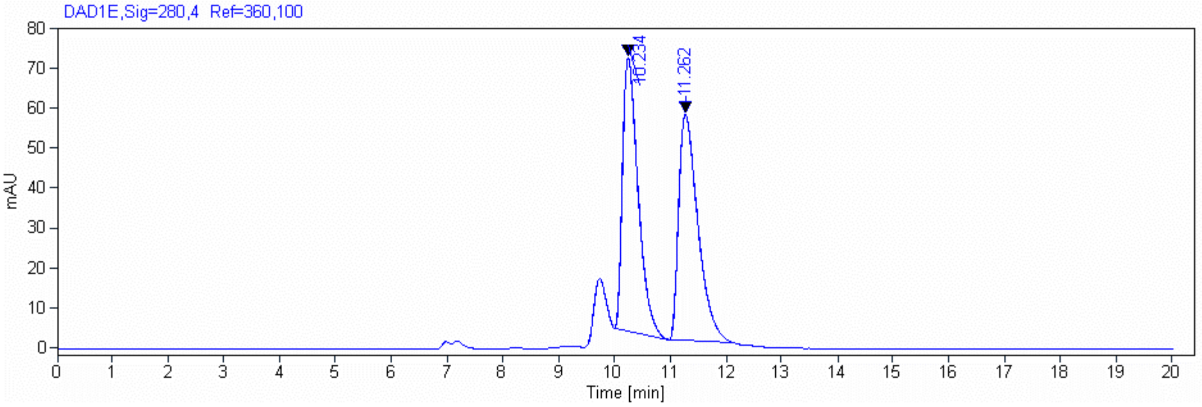


| **Signal:** | DAD1E,Sig=280,4 Ref=360,100 | | | |
|---|---|---|---|---|
| **RT [min]** | **Width [min]** | **Area** | **Height** | **Area%** |
| 10.234 | 0.93 | 1330.45 | 68.39 | 49.56 |
| 11.262 | 1.21 | 1354.06 | 56.49 | 50.44 |

**Figure S34.** (*R*)-(1-(4-methoxyphenyl)ethyl)cycloheptane

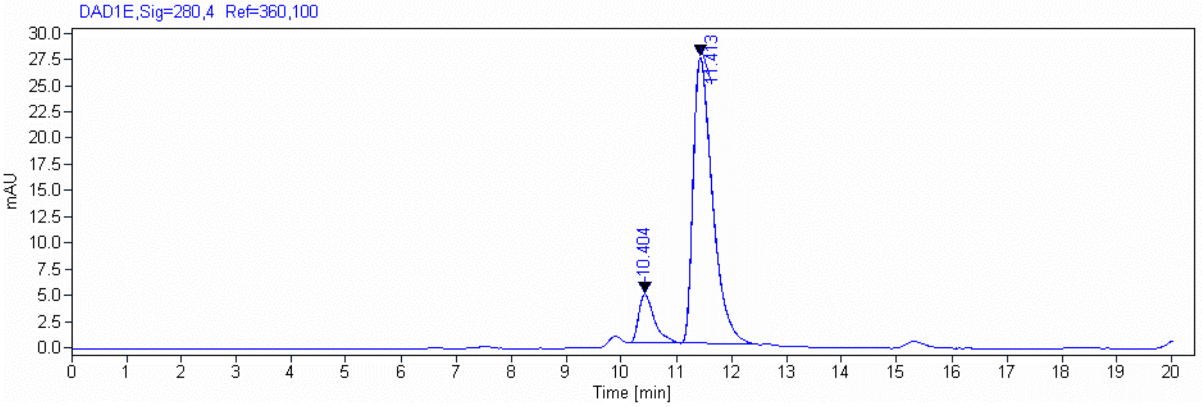


| **Signal:** | DAD1E,Sig=280,4 Ref=360,100 | | | |
|---|---|---|---|---|
| **RT [min]** | **Width [min]** | **Area** | **Height** | **Area%** |
| 10.404 | 0.86 | 90.72 | 4.61 | 12.02 |
| 11.413 | 1.42 | 664.13 | 27.22 | 87.98 |

### 8.2.15 (*R*)-1-(4,4-dimethylpentan-2-yl)-4-methoxybenzene (5c)

**Figure S35.** *rac*-1-(4,4-dimethylpentan-2-yl)-4-methoxybenzene

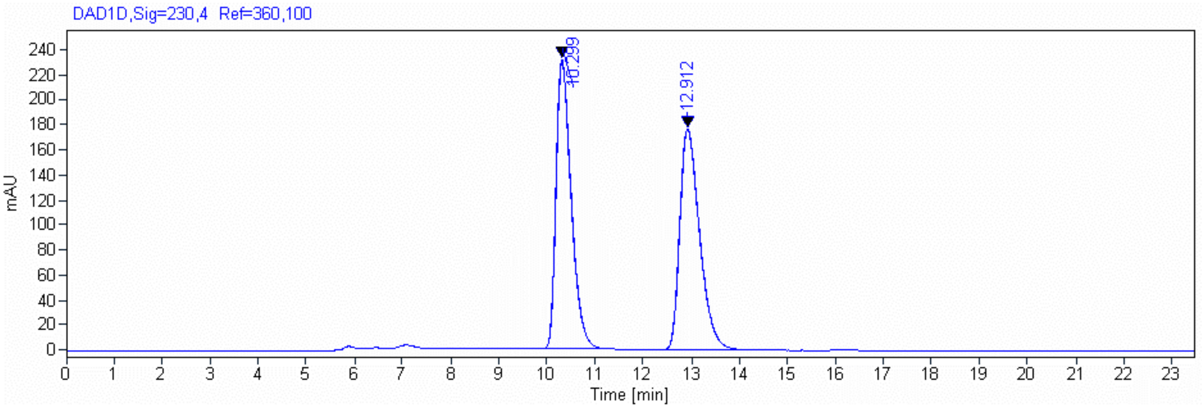


| Signal: | DAD1D,Sig=230,4 Ref=360,100 | | | |
|---|---|---|---|---|
| **RT [min]** | **Width [min]** | **Area** | **Height** | **Area%** |
| 10.299 | 1.59 | 5058.25 | 231.42 | 50.00 |
| 12.912 | 2.48 | 5057.36 | 176.76 | 50.00 |

**Figure S36.** (*R*)-1-(4,4-dimethylpentan-2-yl)-4-methoxybenzene

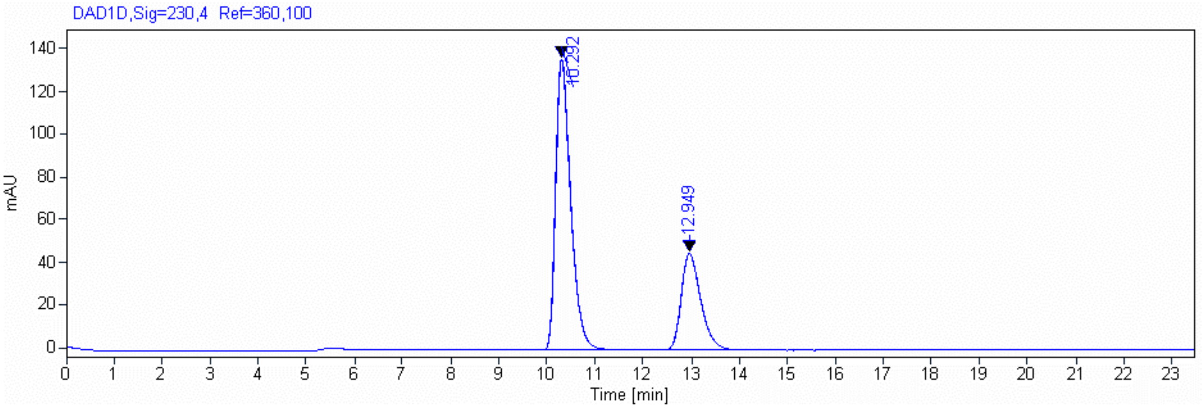


| Signal: | DAD1D,Sig=230,4 Ref=360,100 | | | |
|---|---|---|---|---|
| **RT [min]** | **Width [min]** | **Area** | **Height** | **Area%** |
| 10.292 | 1.55 | 2945.89 | 136.04 | 69.94 |
| 12.949 | 1.70 | 1266.25 | 45.22 | 30.06 |

### 8.2.16 Methyl (*R*)-4-(4-methoxyphenyl)pentanoate (5d)

**Figure S37.** *rac*-Methyl-4-(4-methoxyphenyl)pentanoate

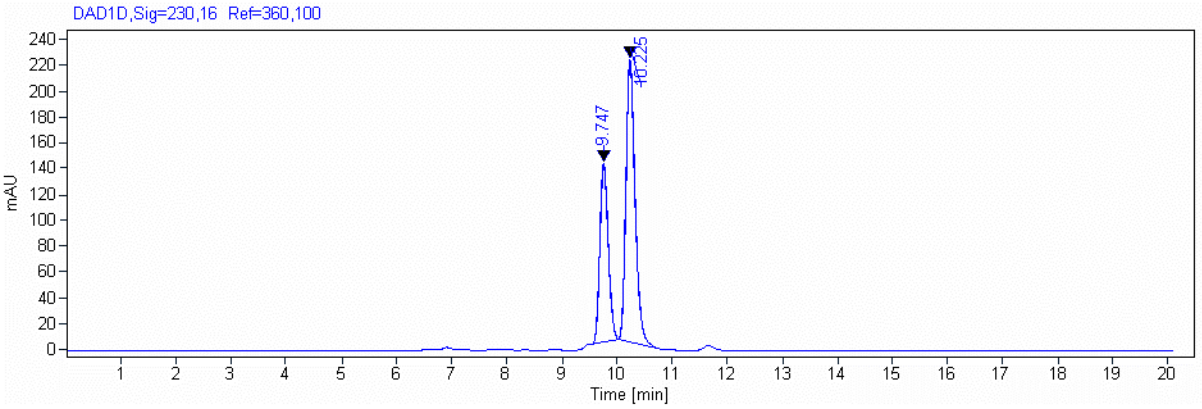


| Signal: | DAD1D,Sig=230,16 Ref=360,100 | | | |
|---|---|---|---|---|
| **RT [min]** | **Width [min]** | **Area** | **Height** | **Area%** |
| 15.252 | 2.27 | 2648.25 | 106.80 | 49.85 |
| 18.343 | 1.66 | 2664.64 | 130.65 | 50.15 |

**Figure S38.** Methyl (*R*)-4-(4-methoxyphenyl)pentanoate

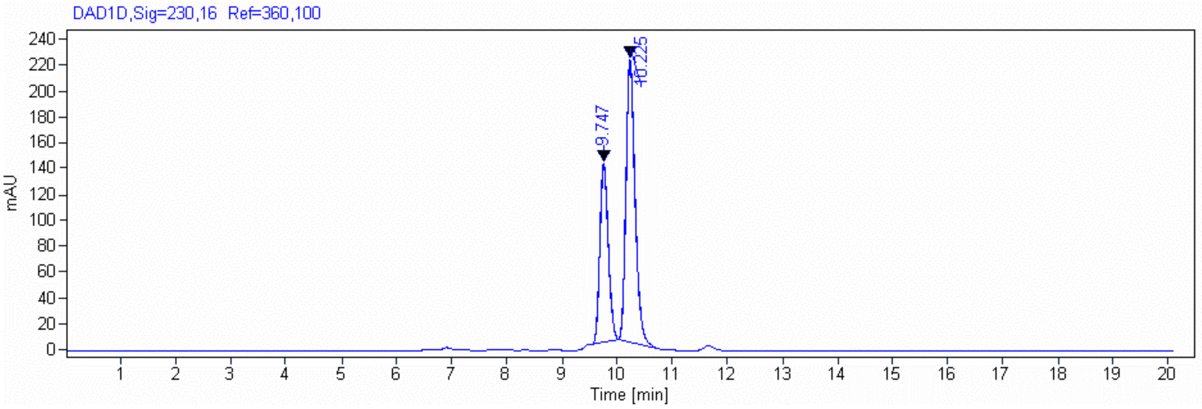


| Signal: | DAD1D,Sig=230,16 Ref=360,100 | | | |
|---|---|---|---|---|
| **RT [min]** | **Width [min]** | **Area** | **Height** | **Area%** |
| 9.747 | 0.51 | 1447.31 | 138.36 | 36.58 |
| 10.225 | 0.73 | 2509.38 | 218.70 | 63.42 |

### 8.2.17 (*R*)-1-methoxy-4-(1-phenylpropan-2-yl)benzene (5e)

**Figure S39.** *rac*-1-methoxy-4-(1-phenylpropan-2-yl)benzene

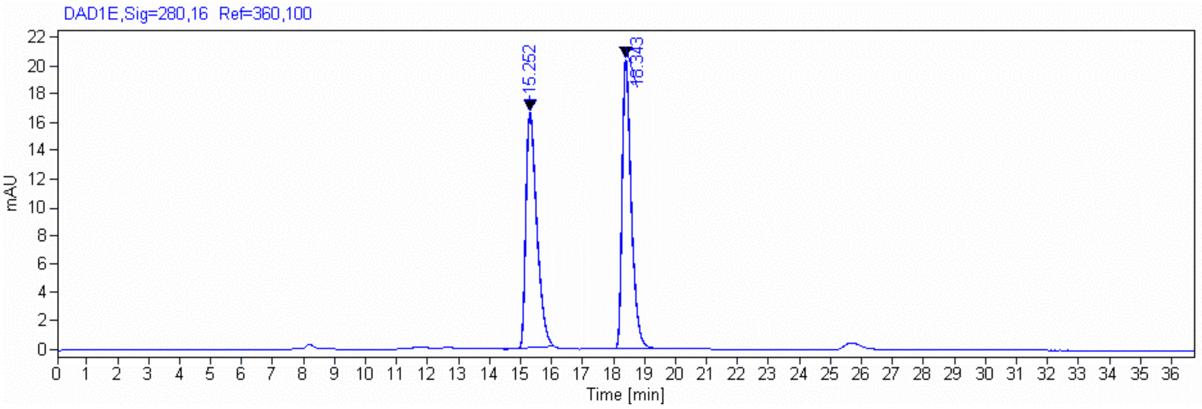


| Signal: | DAD1E,Sig=280,16 Ref=360,100 | | | |
|---|---|---|---|---|
| **RT [min]** | **Width [min]** | **Area** | **Height** | **Area%** |
| 15.252 | 1.53 | 403.84 | 16.61 | 49.27 |
| 18.343 | 1.57 | 415.78 | 20.44 | 50.73 |

**Figure S40.** (*R*)-1-methoxy-4-(1-phenylpropan-2-yl)benzene

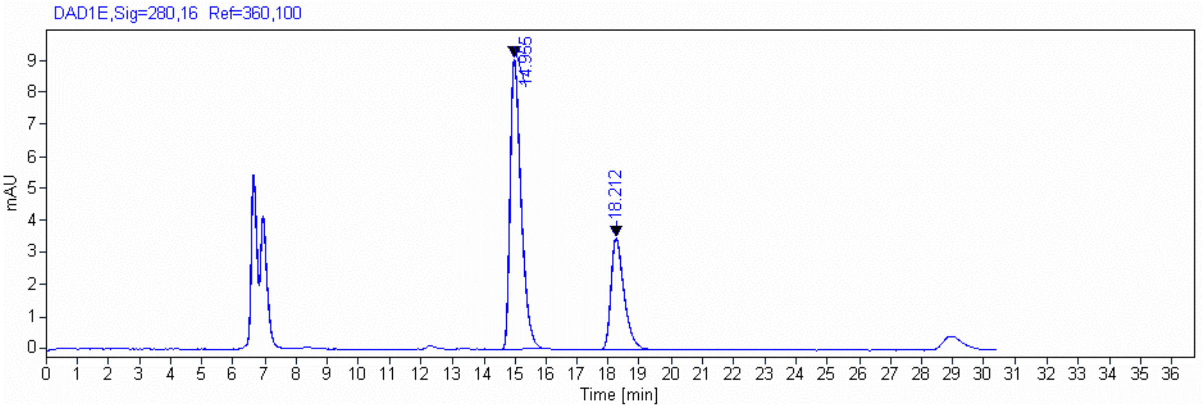


| Signal: | DAD1E,Sig=280,16 Ref=360,100 | | | |
|---|---|---|---|---|
| **RT [min]** | **Width [min]** | **Area** | **Height** | **Area%** |
| 14.955 | 1.39 | 220.68 | 9.07 | 68.67 |
| 18.212 | 2.03 | 100.66 | 3.48 | 31.33 |

### 8.2.18 (*R*)-(1-cyclohexylethyl)benzene (5f)

**Figure S41.** *rac*-(1-cyclohexylethyl)benzene

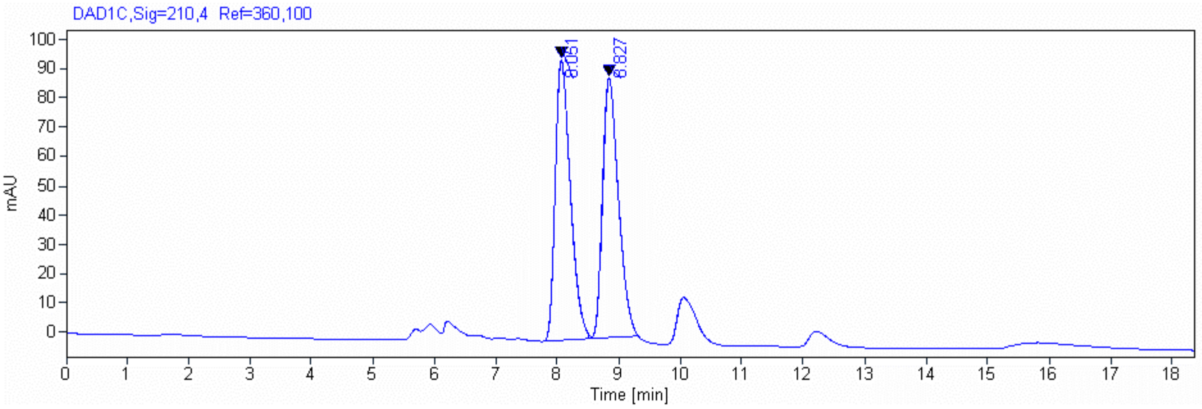


| **Signal:** | DAD1C,Sig=210,4 Ref=360,100 | | | |
|---|---|---|---|---|
| **RT [min]** | **Width [min]** | **Area** | **Height** | **Area%** |
| 8.051 | 0.73 | 1642.23 | 96.22 | 50.65 |
| 8.827 | 0.81 | 1599.99 | 89.01 | 49.35 |

**Figure S42.** (*R*)-(1-cyclohexylethyl)benzene

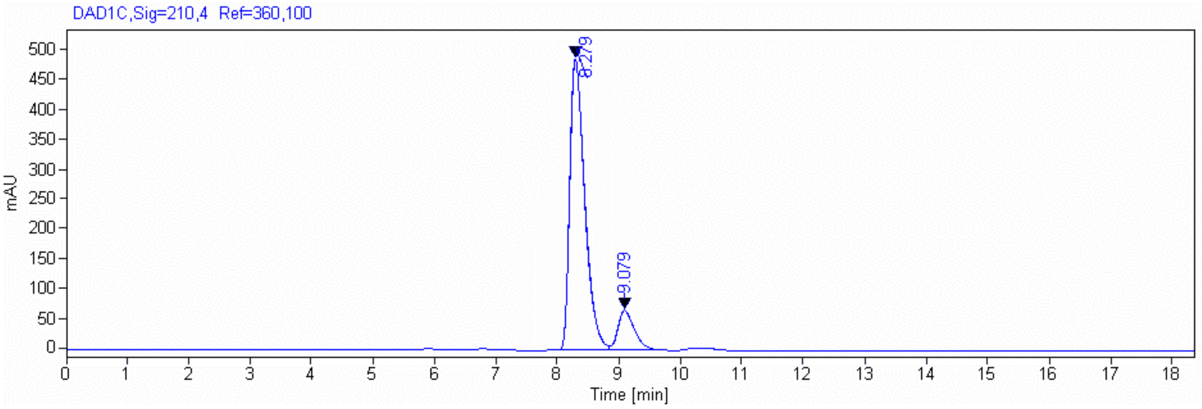


| **Signal:** | DAD1C,Sig=210,4 Ref=360,100 | | | |
|---|---|---|---|---|
| **RT [min]** | **Width [min]** | **Area** | **Height** | **Area%** |
| 8.279 | 0.87 | 8204.16 | 485.46 | 87.25 |
| 9.079 | 0.76 | 1199.30 | 65.65 | 12.75 |

# 9 NMR Spectra

**Figure S43.** $^1$H NMR spectrum (400 MHz, $CDCl_3$, 298 K) of **S1a**.

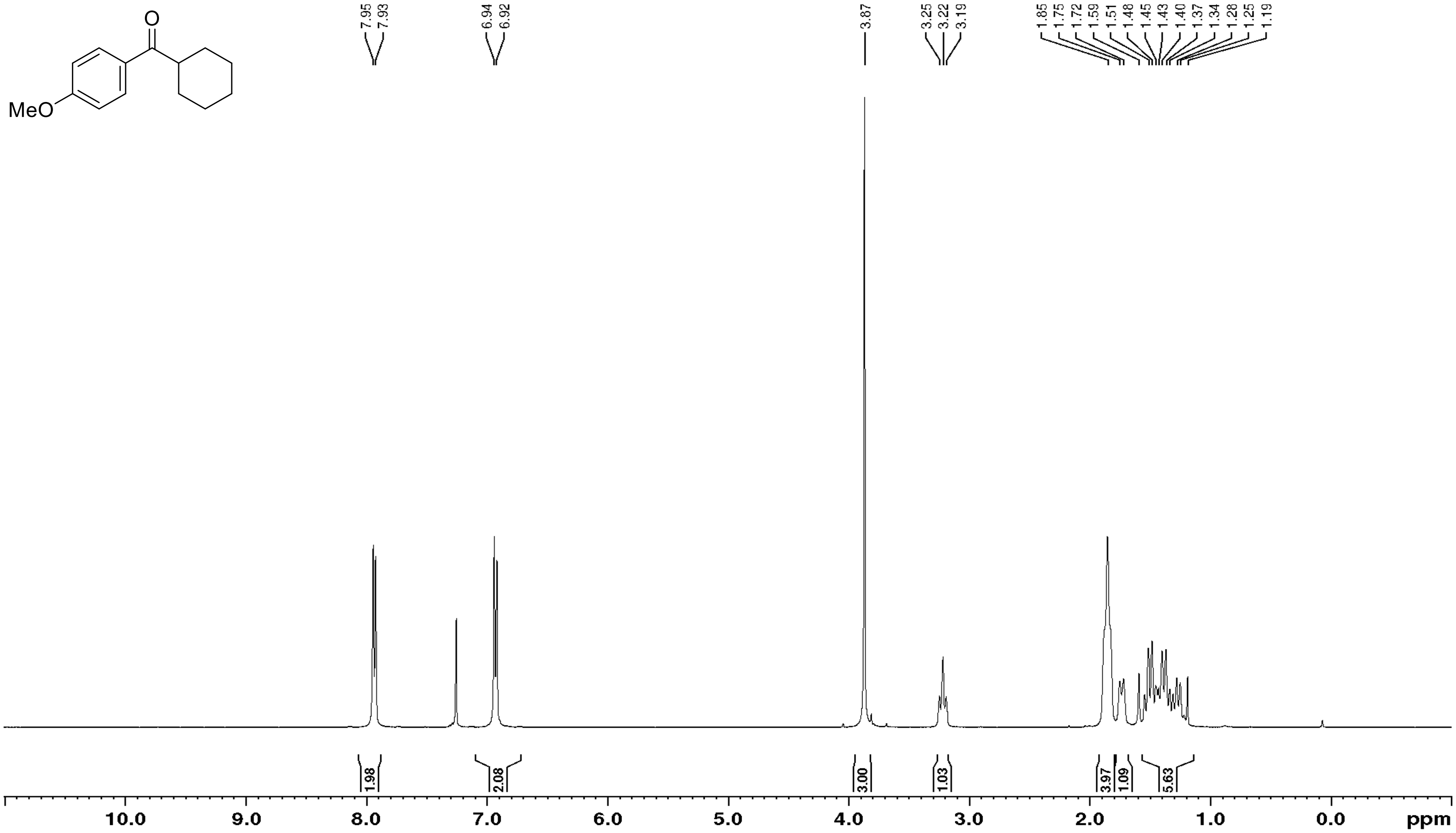

**Figure S44.** $^{13}C\{^1H\}$ NMR spectrum (100 MHz, $CDCl_3$, 298 K) of **S1a**.

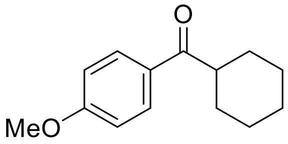


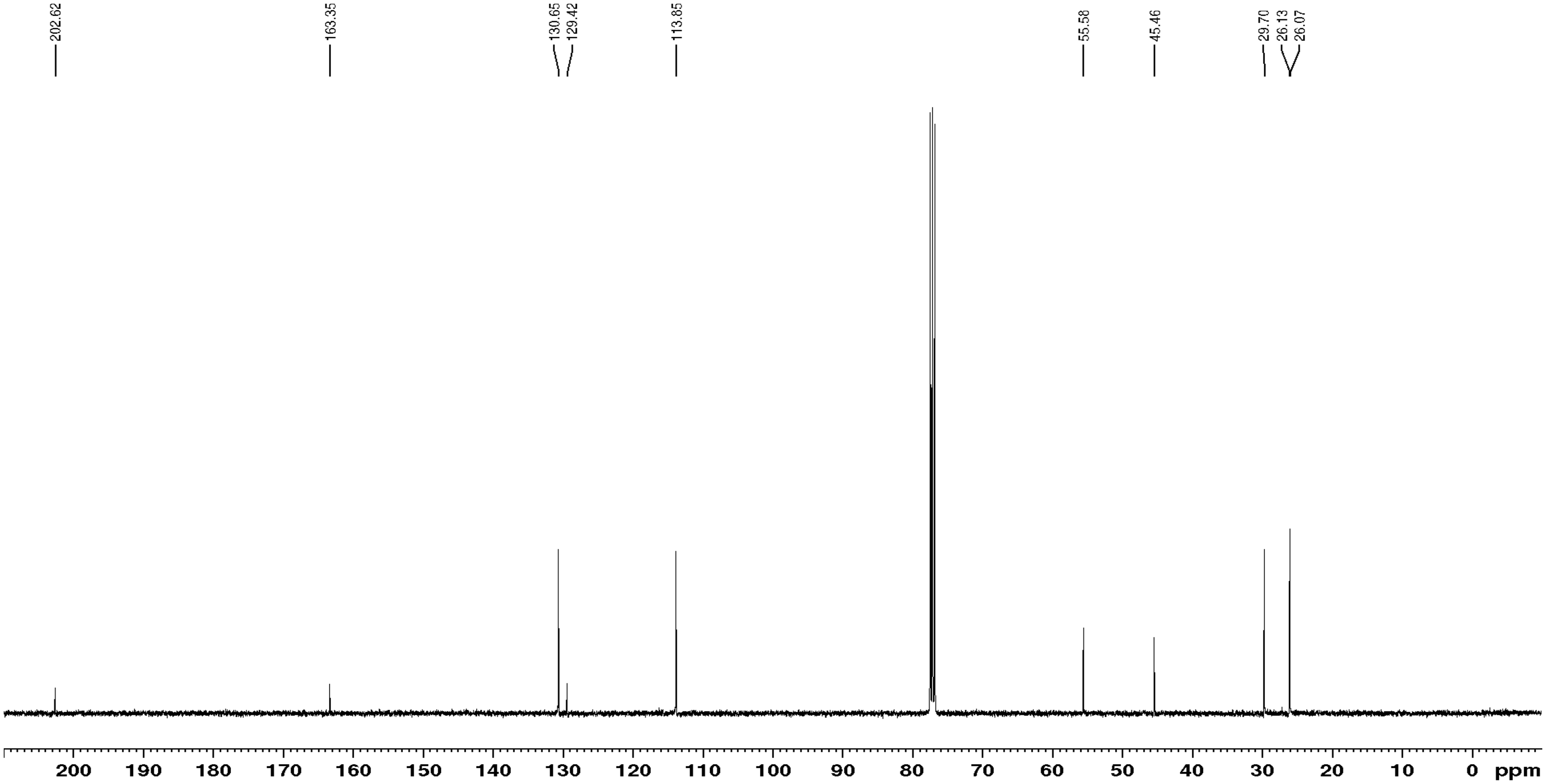

**Figure S45.** $^{1}$H NMR spectrum (400 MHz, $CDCl_3$, 298 K) of **S1b**.

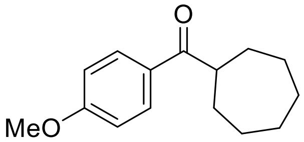


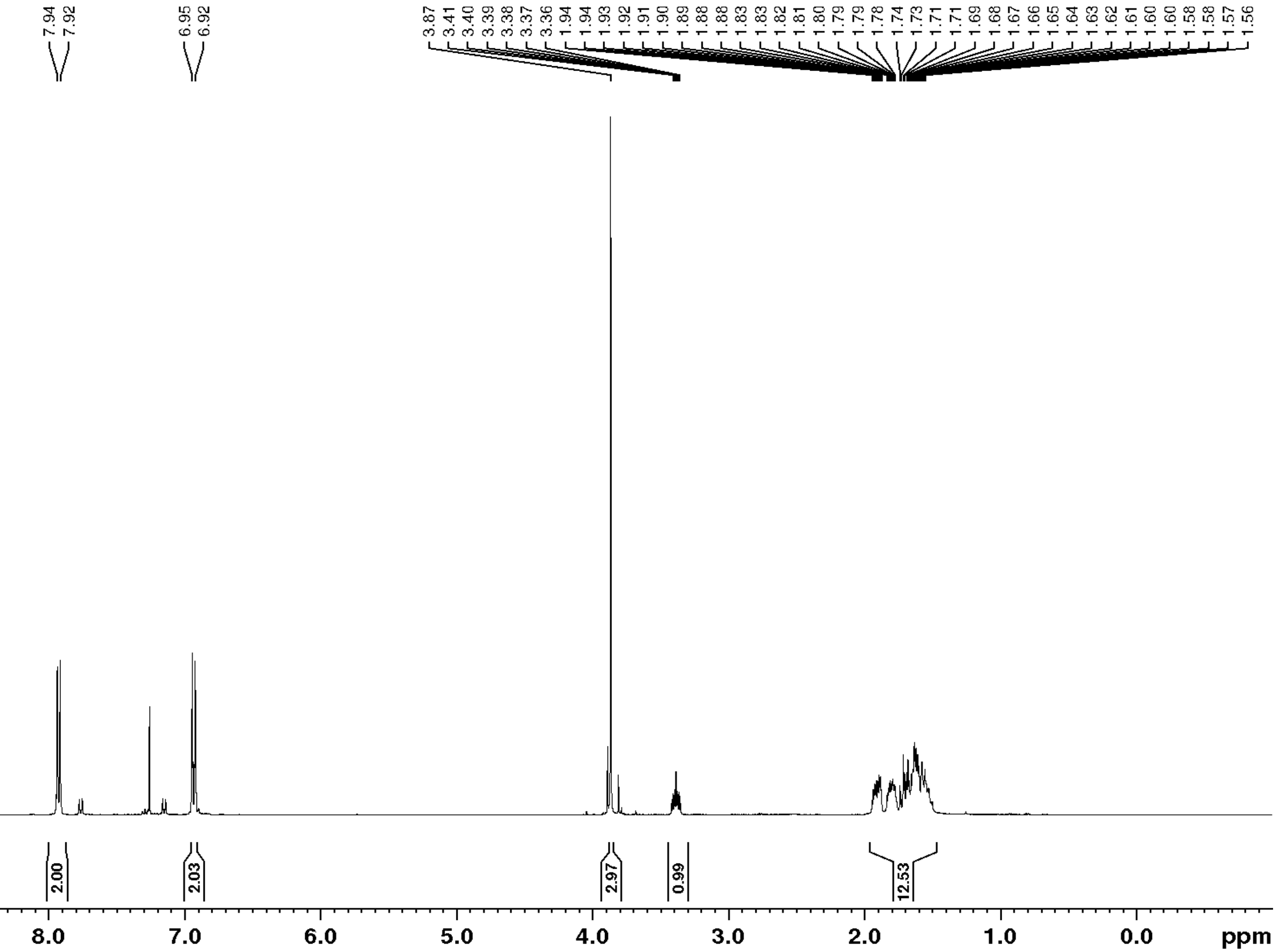

**Figure S46.** $^{13}C\{1H\}$ NMR spectrum (100 MHz, $CDCl_3$, 298 K) of **S1b**

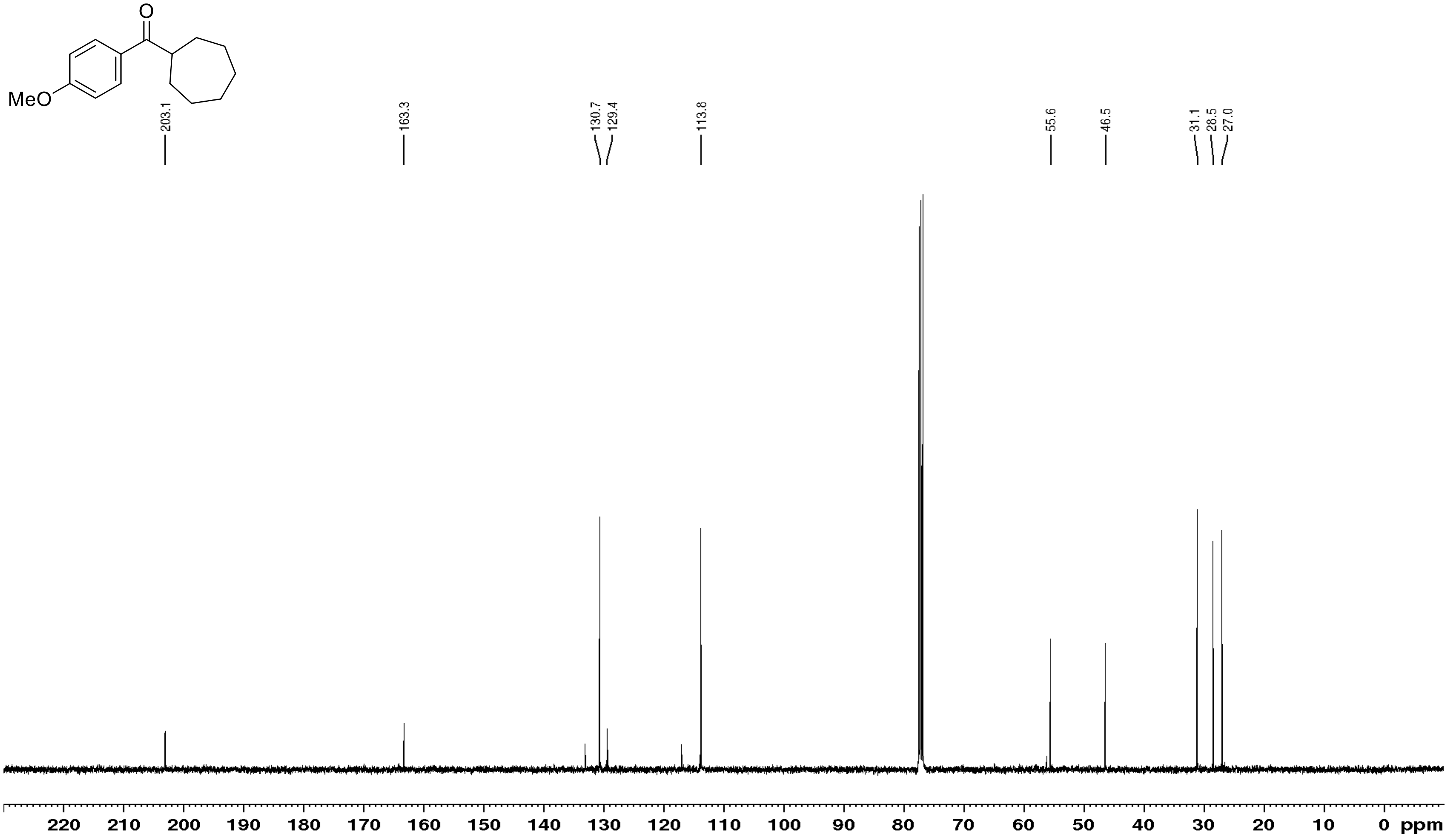

**Figure S47.** $^1$H NMR spectrum (400 MHz, $CDCl_3$, 298 K) of **S1c**.

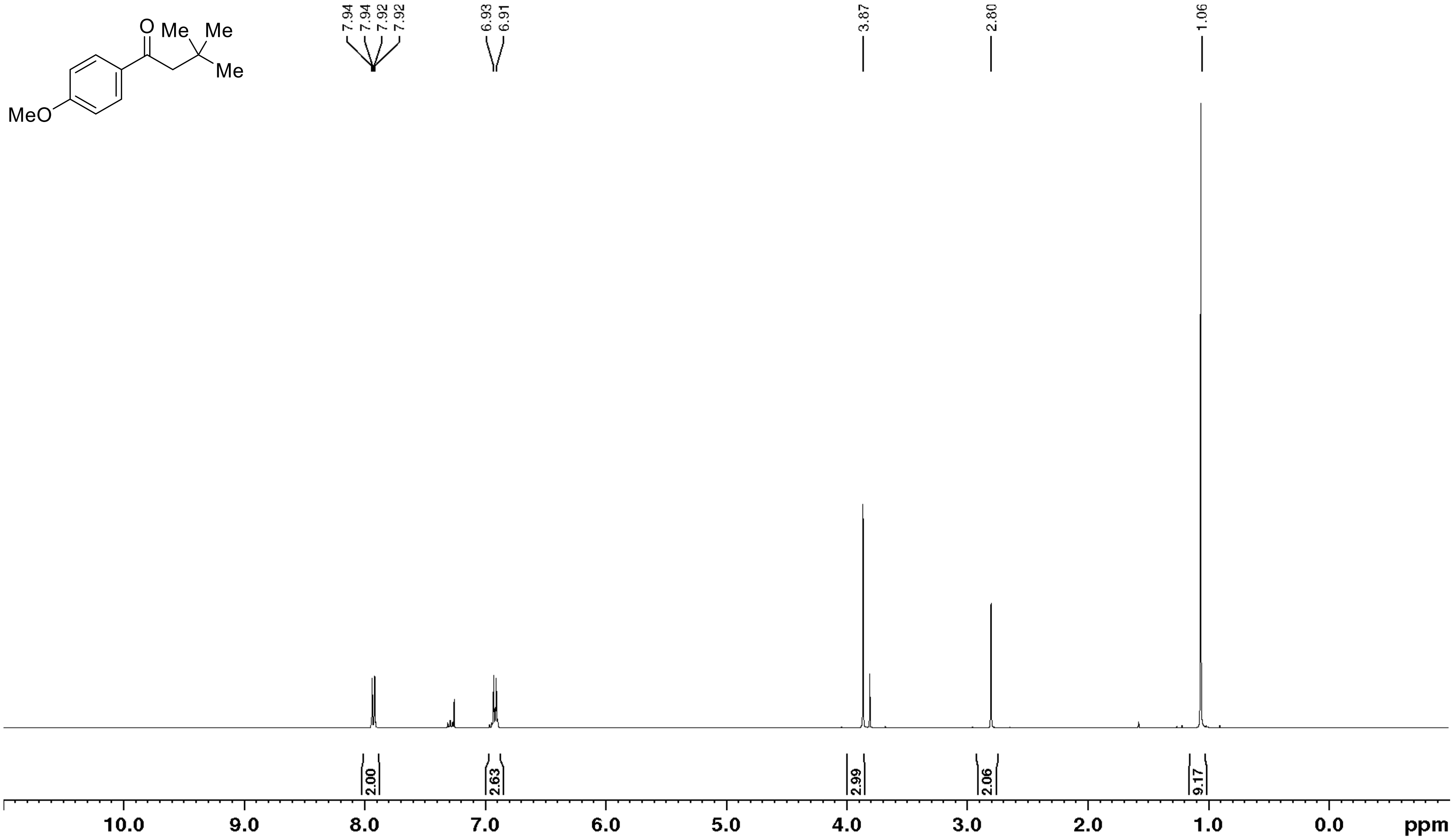

**Figure S48.** $^{13}C\{^1H\}$ NMR spectrum (100 MHz, $CDCl_3$, 298 K) of **Sic**.

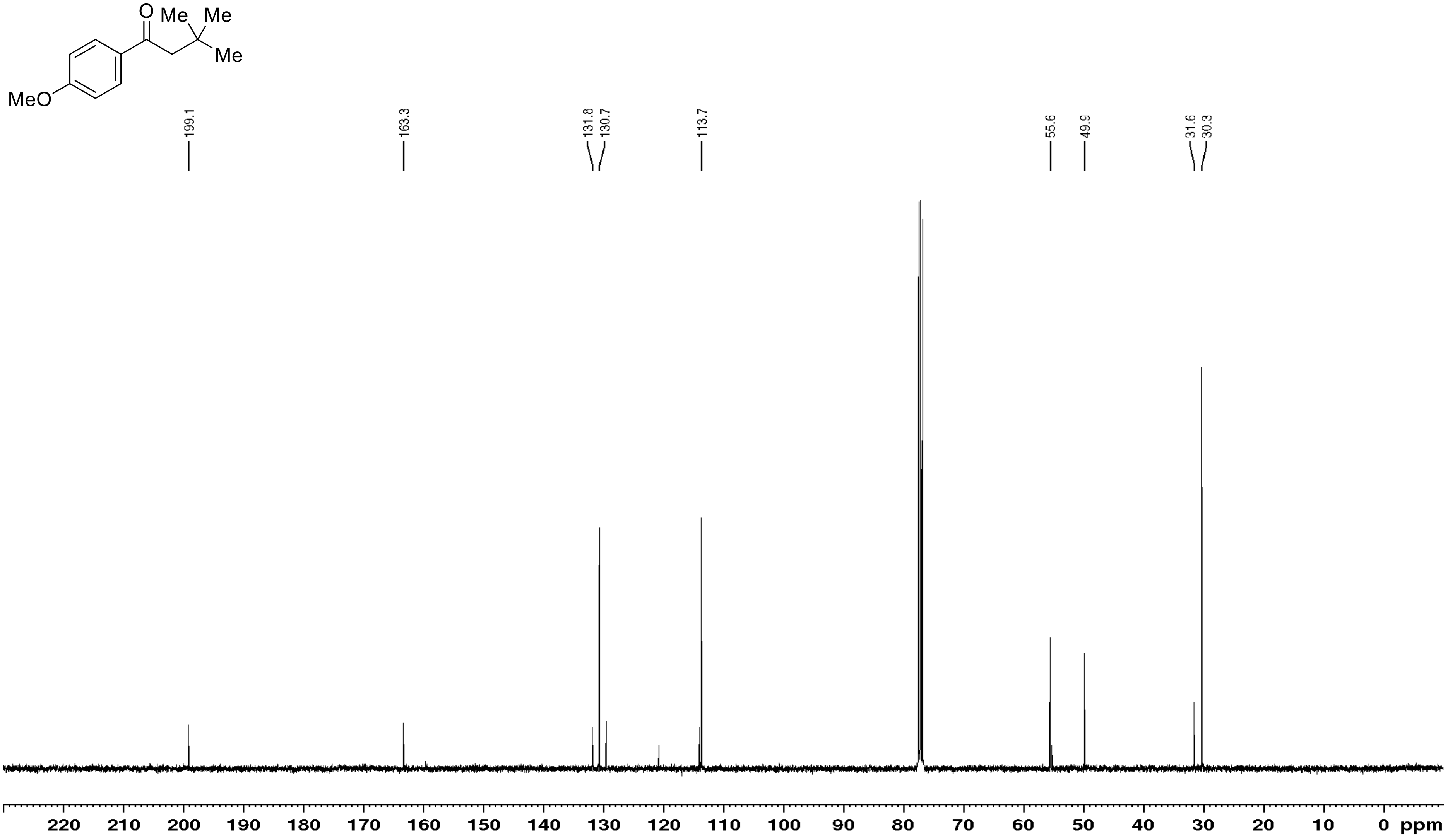

**Figure S49.** $^{1}H$ NMR spectrum (400 MHz, $CDCl_3$, 298 K) of **S1d**.

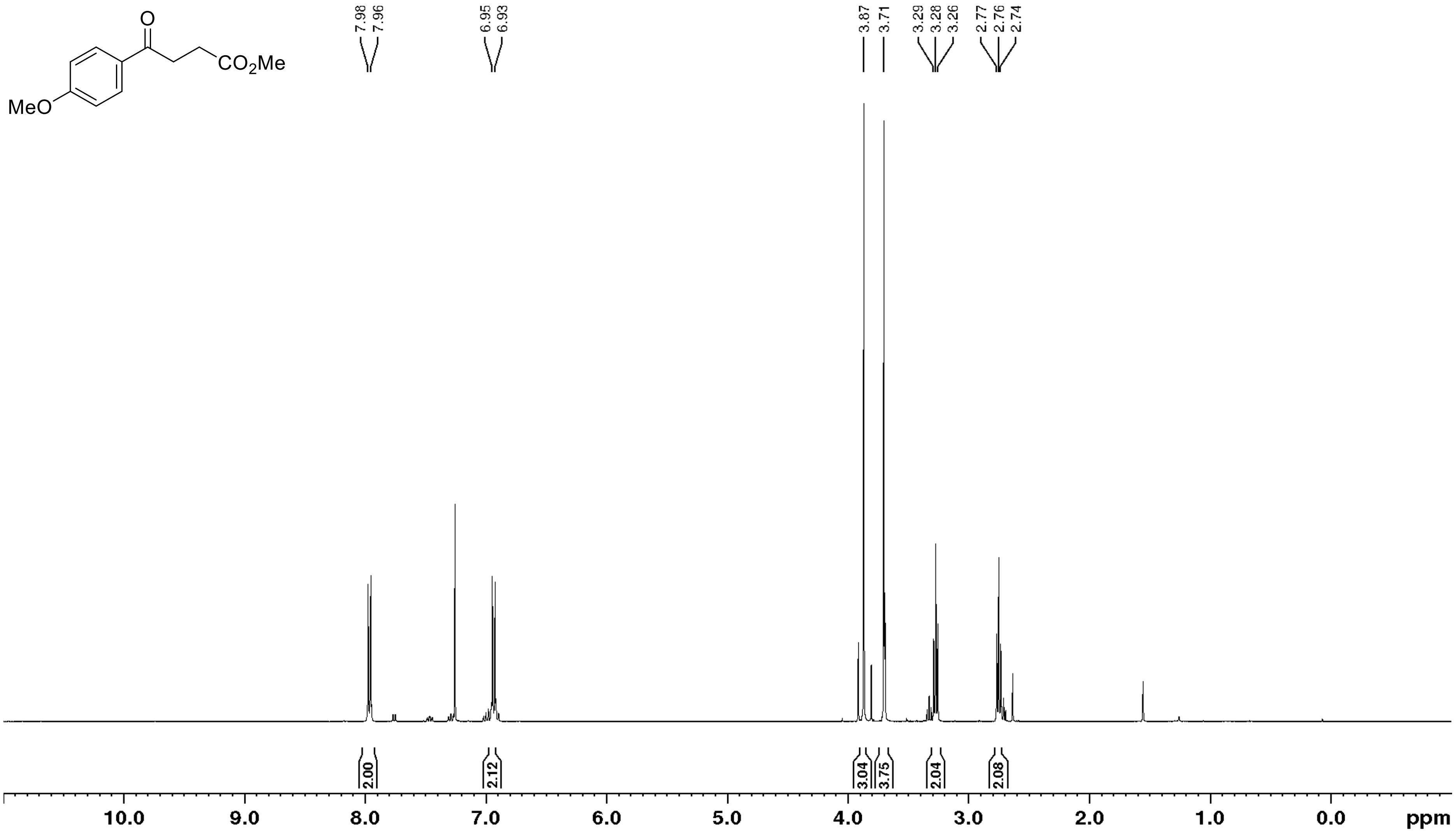

**Figure S50.** $^{13}C\{^1H\}$ NMR spectrum (100 MHz, $CDCl_3$, 298 K) of **S1d**.

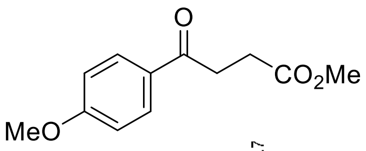


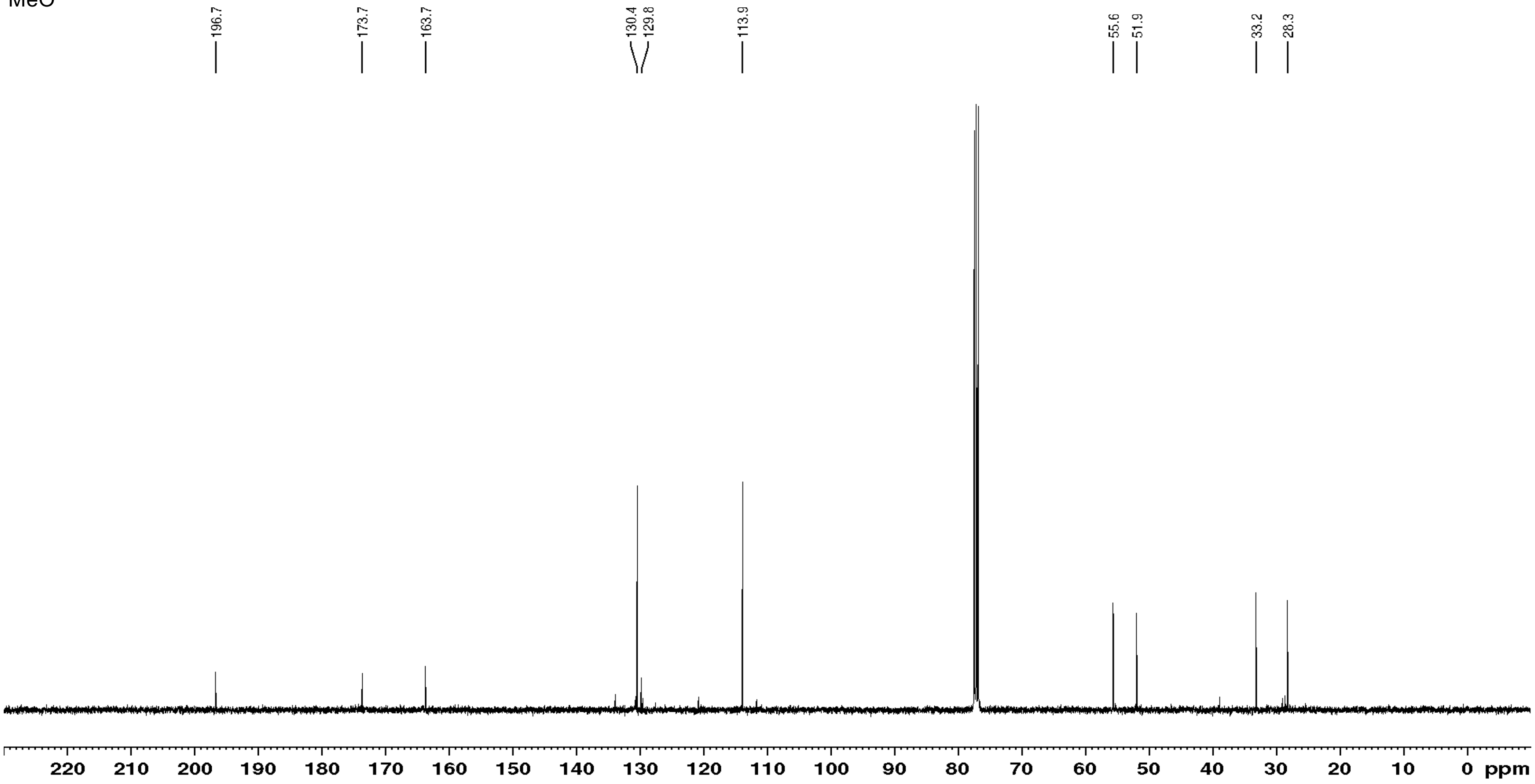

**Figure S51.** $^{1}$H NMR spectrum (400 MHz, $CDCl_3$, 298 K) of **S1e**.

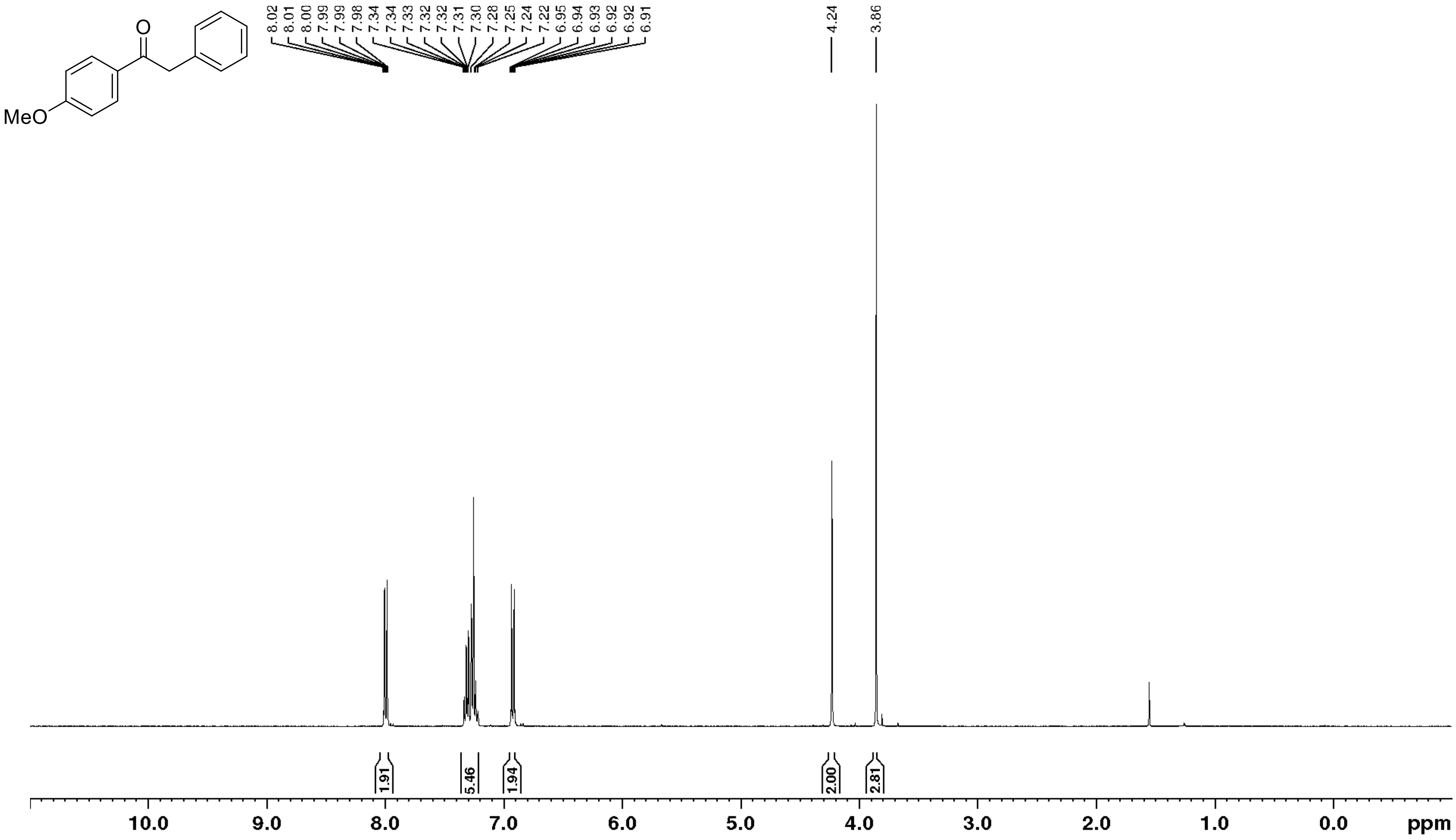

**Figure S52.** $^{13}C\{^1H\}$ NMR spectrum (100 MHz, $CDCl_3$, 298 K) of **S1e**.

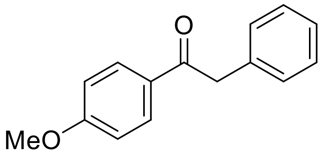


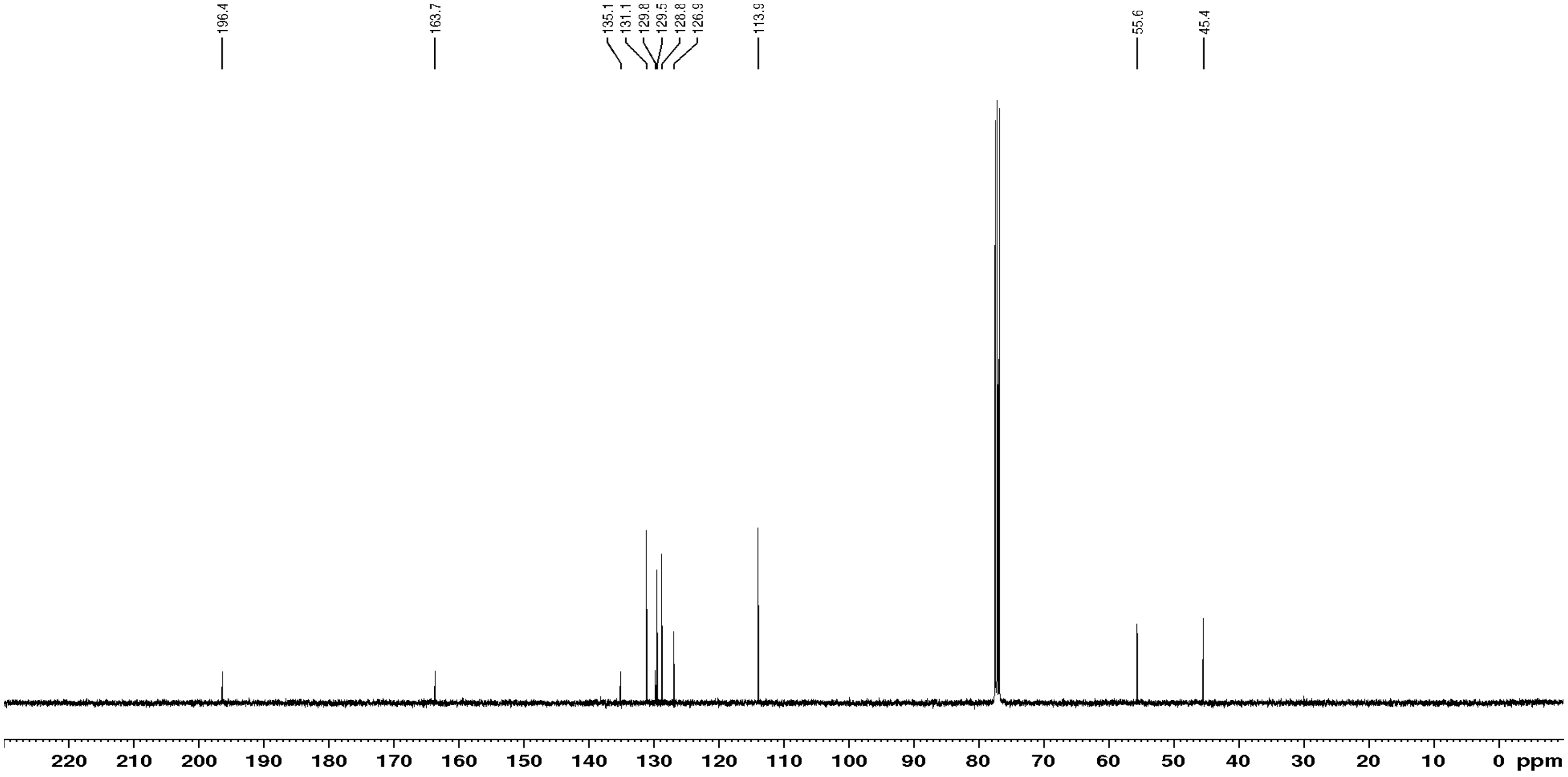

**Figure S53.** $^{1}H$ NMR spectrum (400 MHz, $CDCl_3$, 298 K) of **4a**.

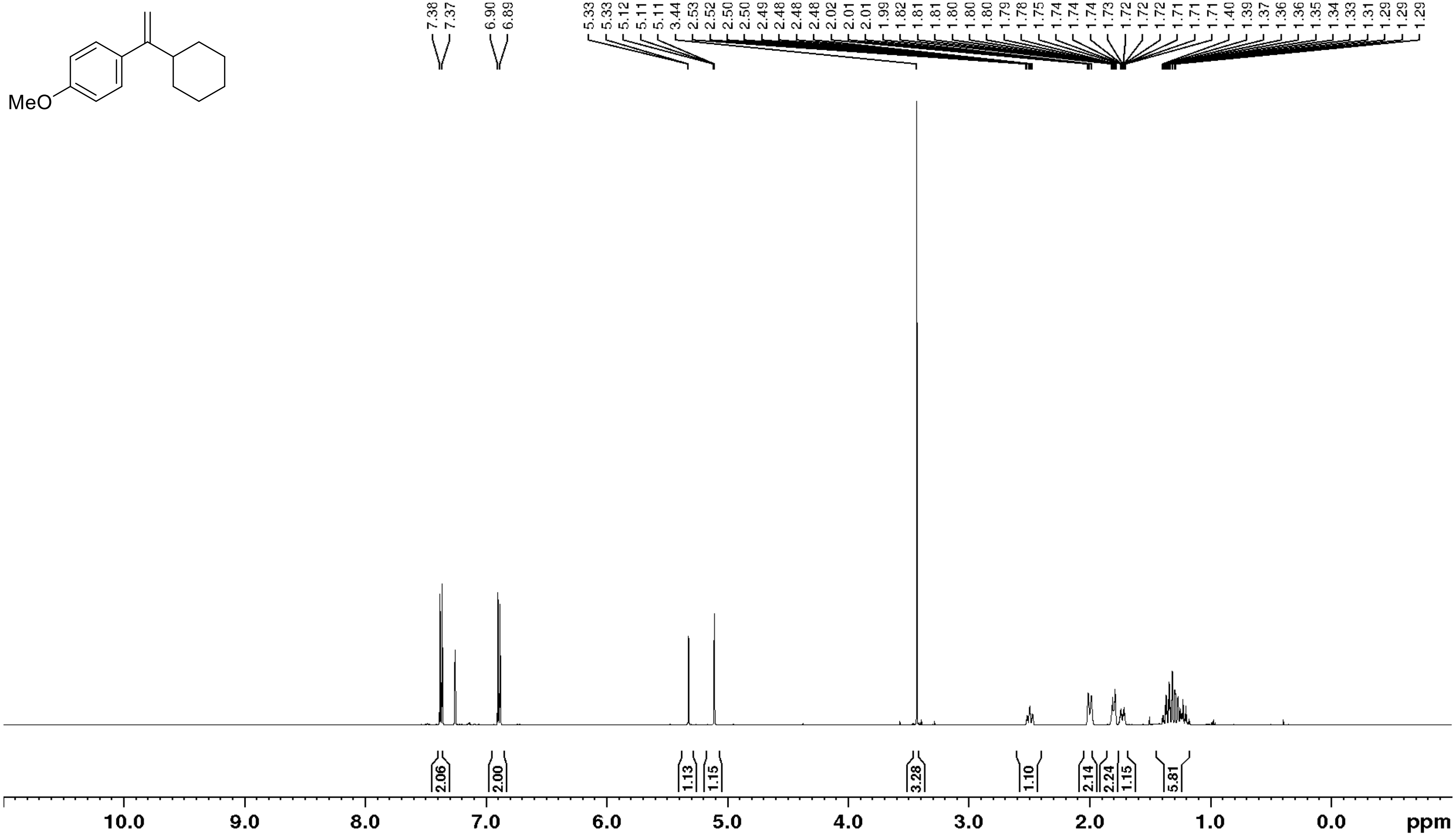

**Figure S54.** $^{13}C\{^1H\}$ NMR spectrum (100 MHz, $CDCl_3$, 298 K) of **4a**.

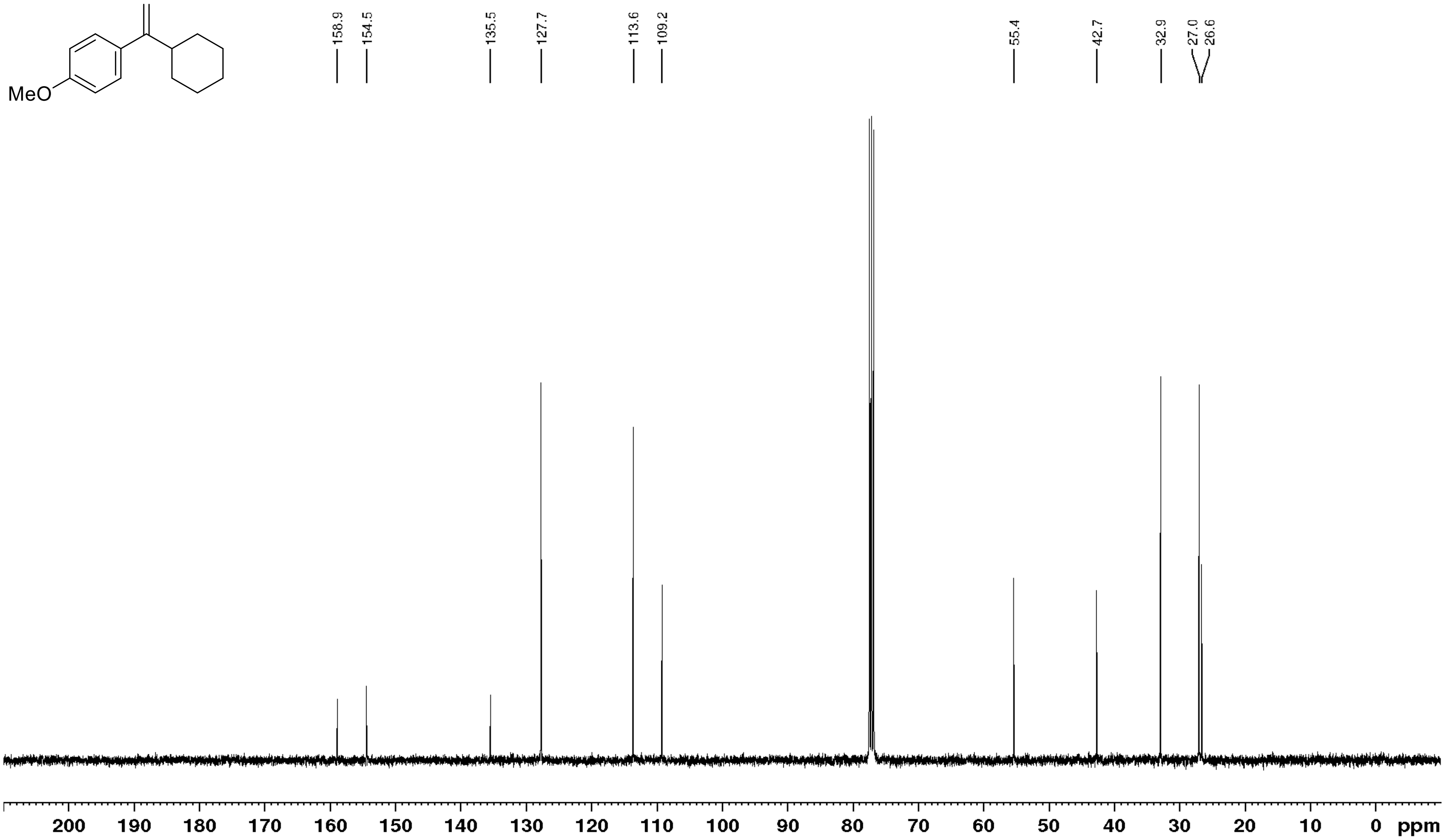

**Figure S55.** $^{1}H$ NMR spectrum (400 MHz, $CDCl_3$, 298 K) of **4b**.

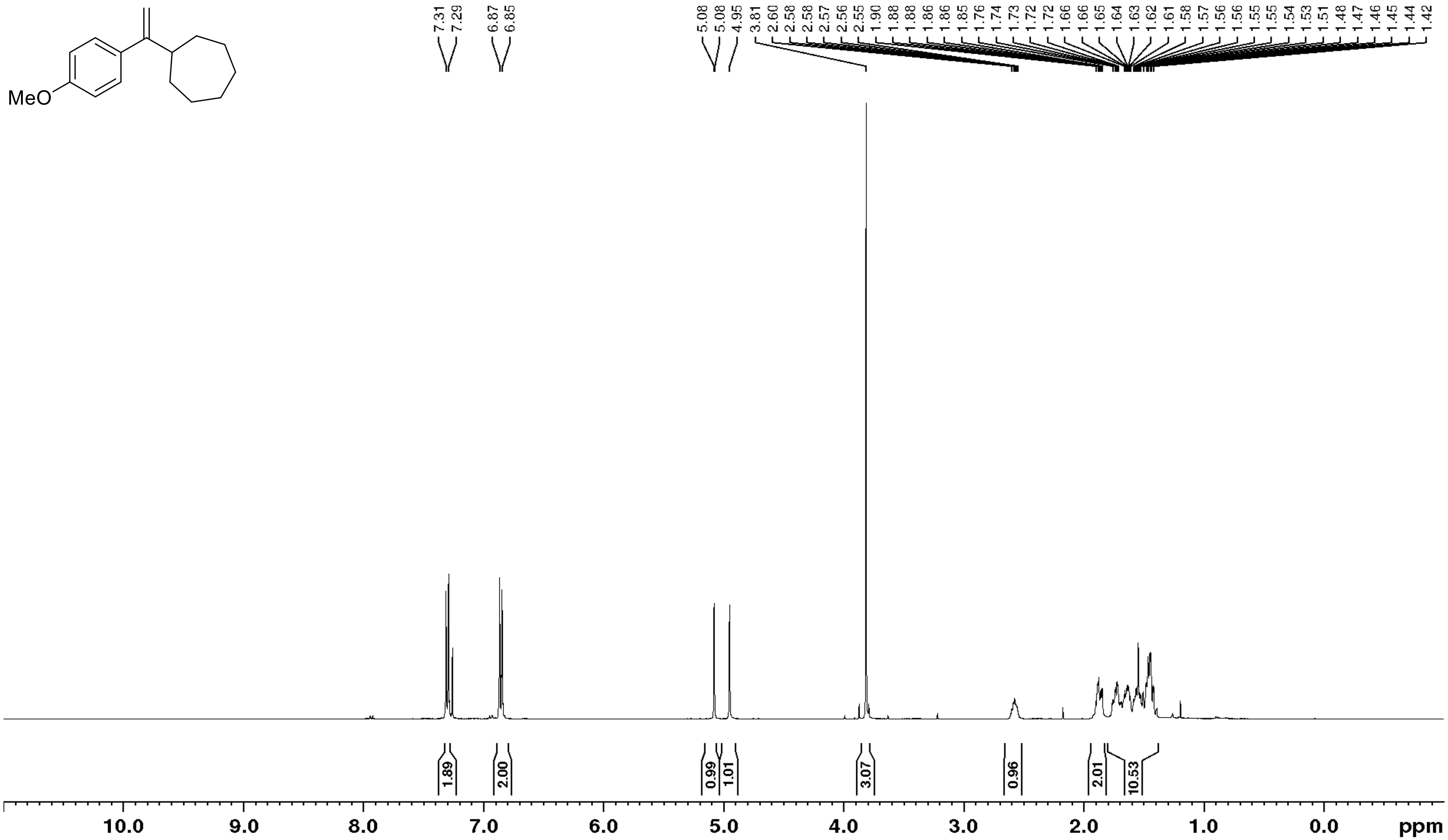

**Figure S56.** $^{13}C\{1H\}$ NMR spectrum (100 MHz, $CDCl_3$, 298 K) of **4b**.

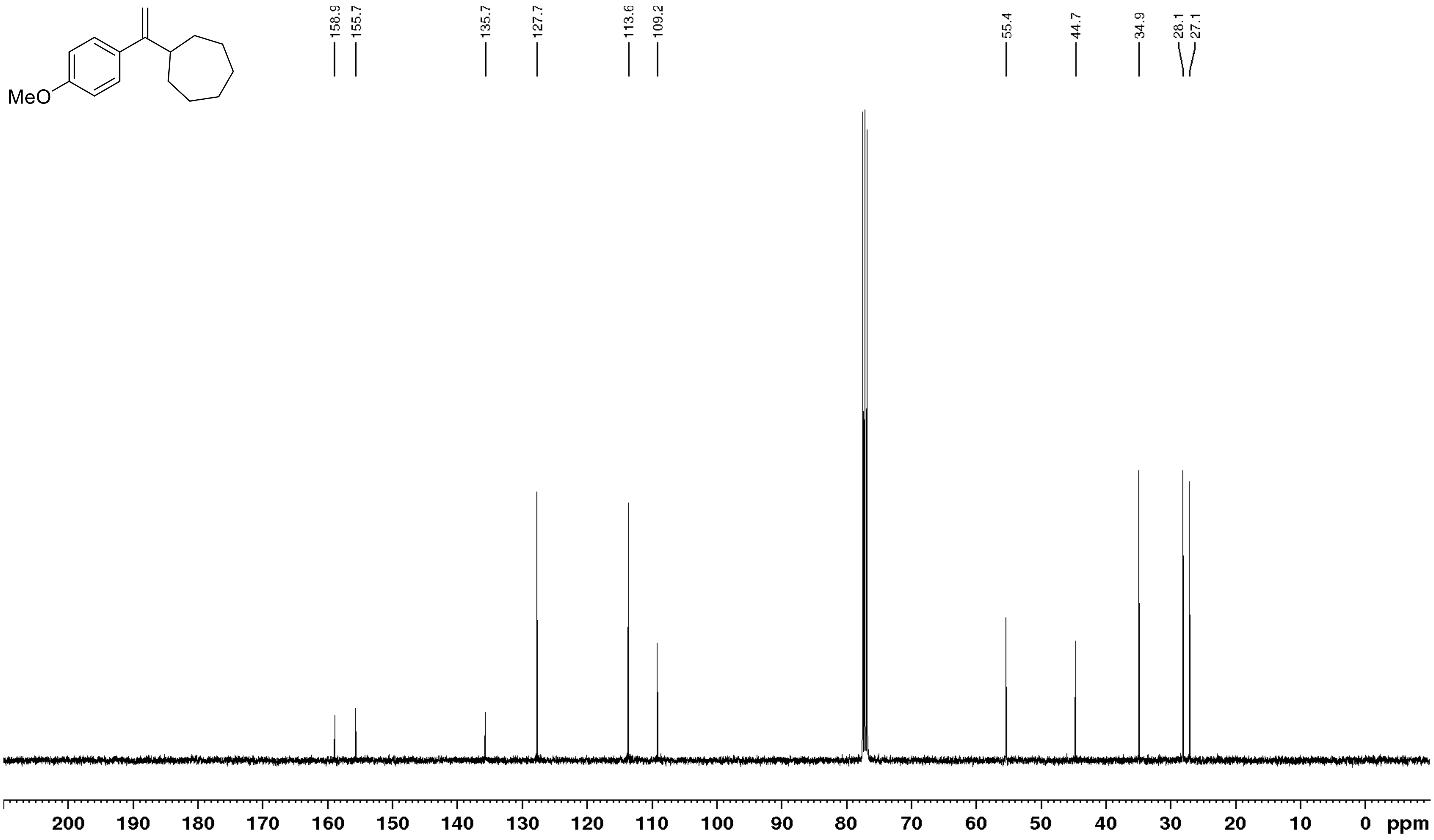

**Figure S57.** ¹H NMR spectrum (400 MHz, $CDCl_3$, 298 K) of **4c**.

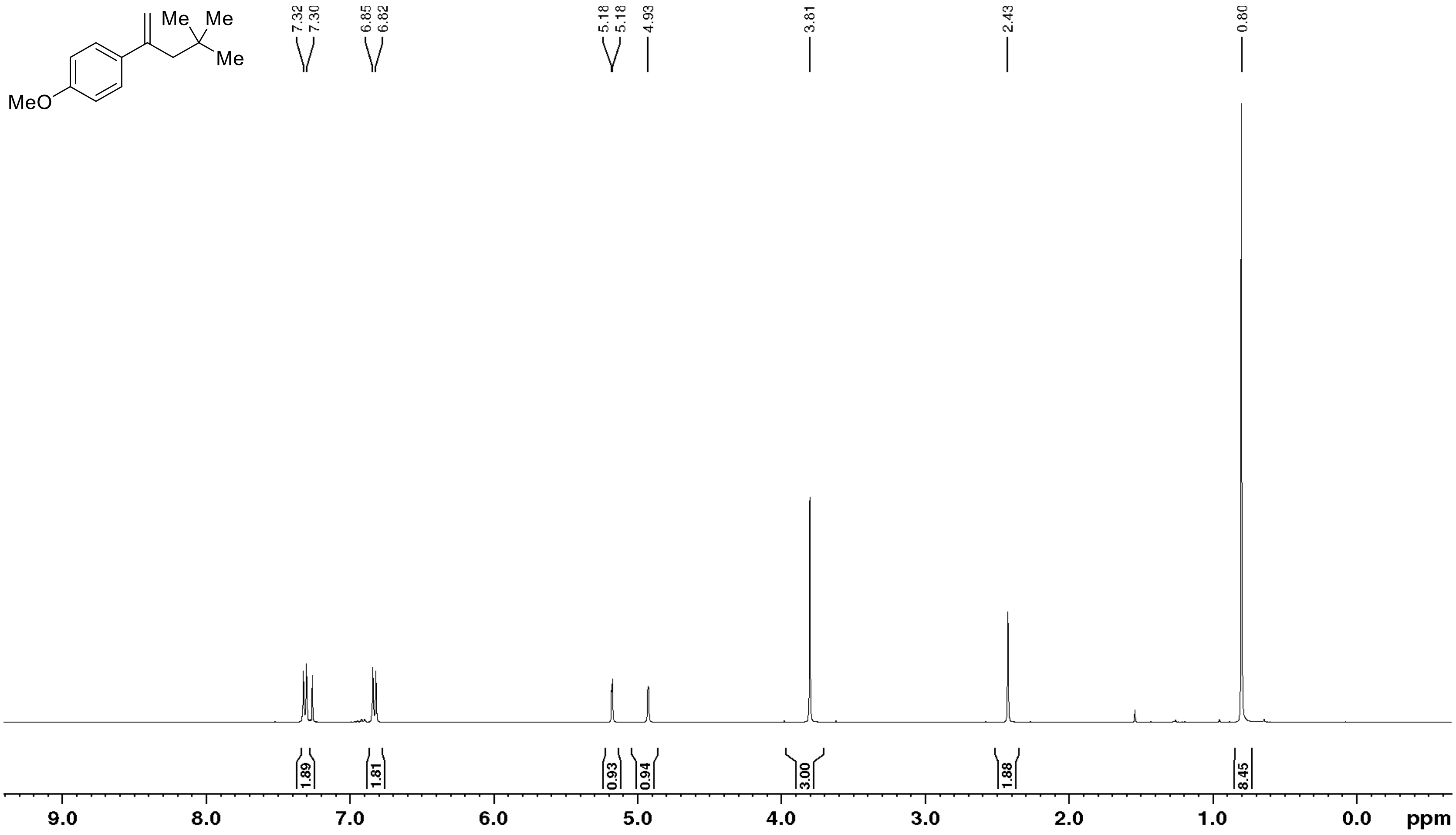

**Figure S58.** $^{13}C\{1H\}$ NMR spectrum (100 MHz, $CDCl_3$, 298 K) of **4c**.

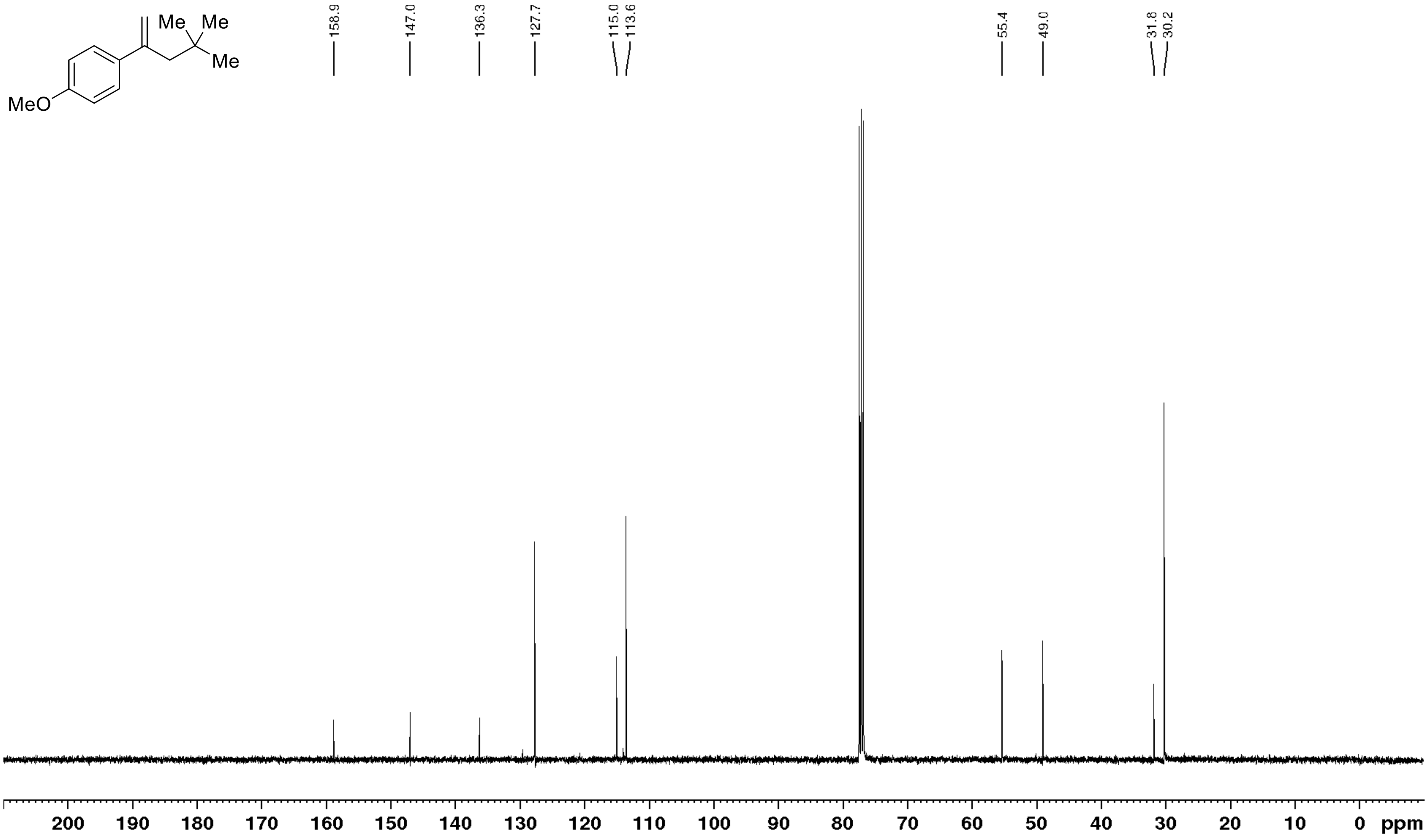

**Figure S59.** $^{1}H$ NMR spectrum (400 MHz, $CDCl_3$, 298 K) of **4d**.

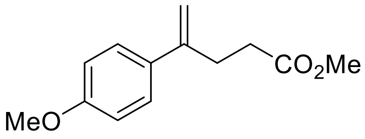


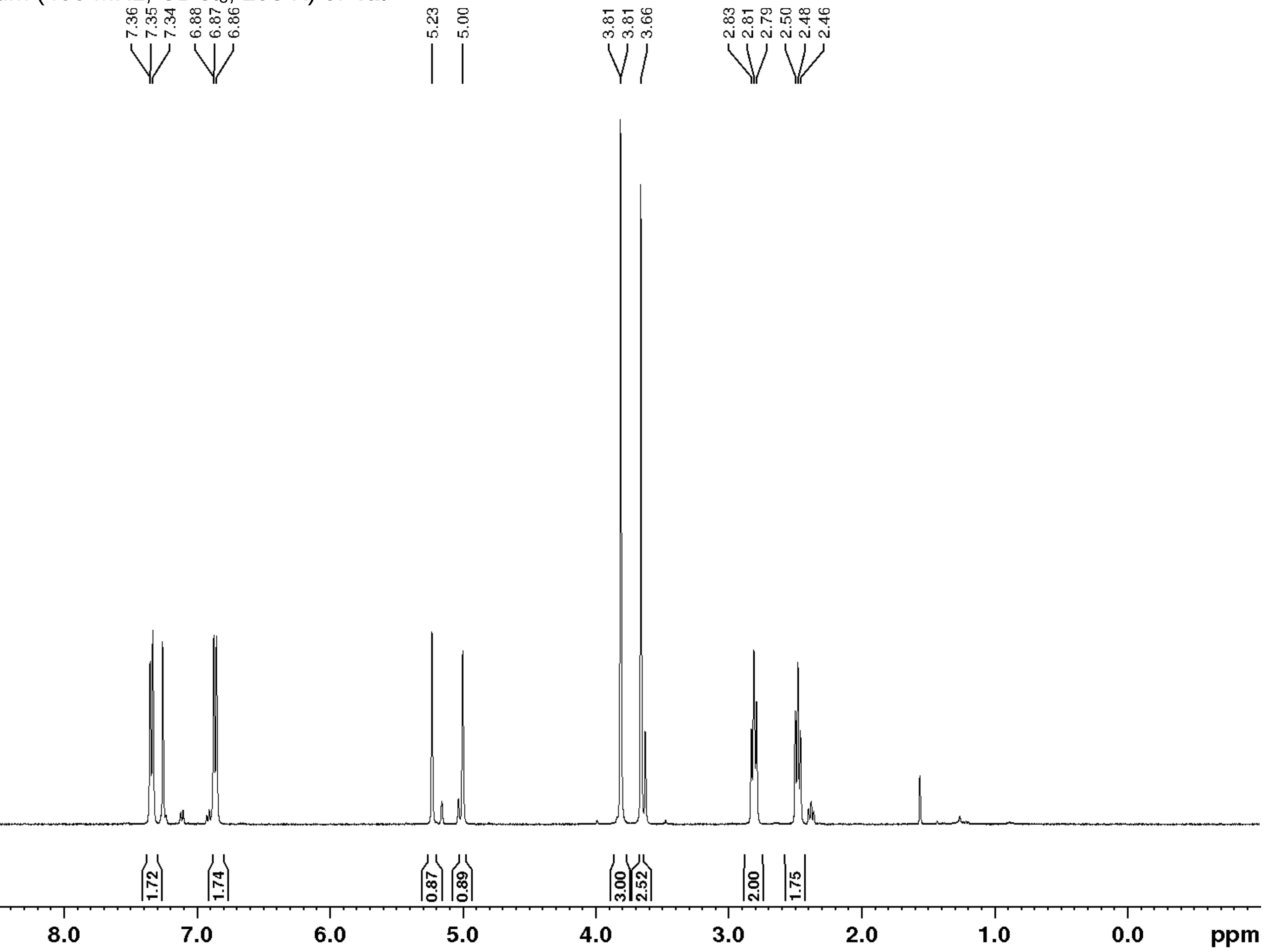

**Figure S60.** $^{13}C\{1H\}$ NMR spectrum (100 MHz, $CDCl_3$, 298 K) of **4d**.

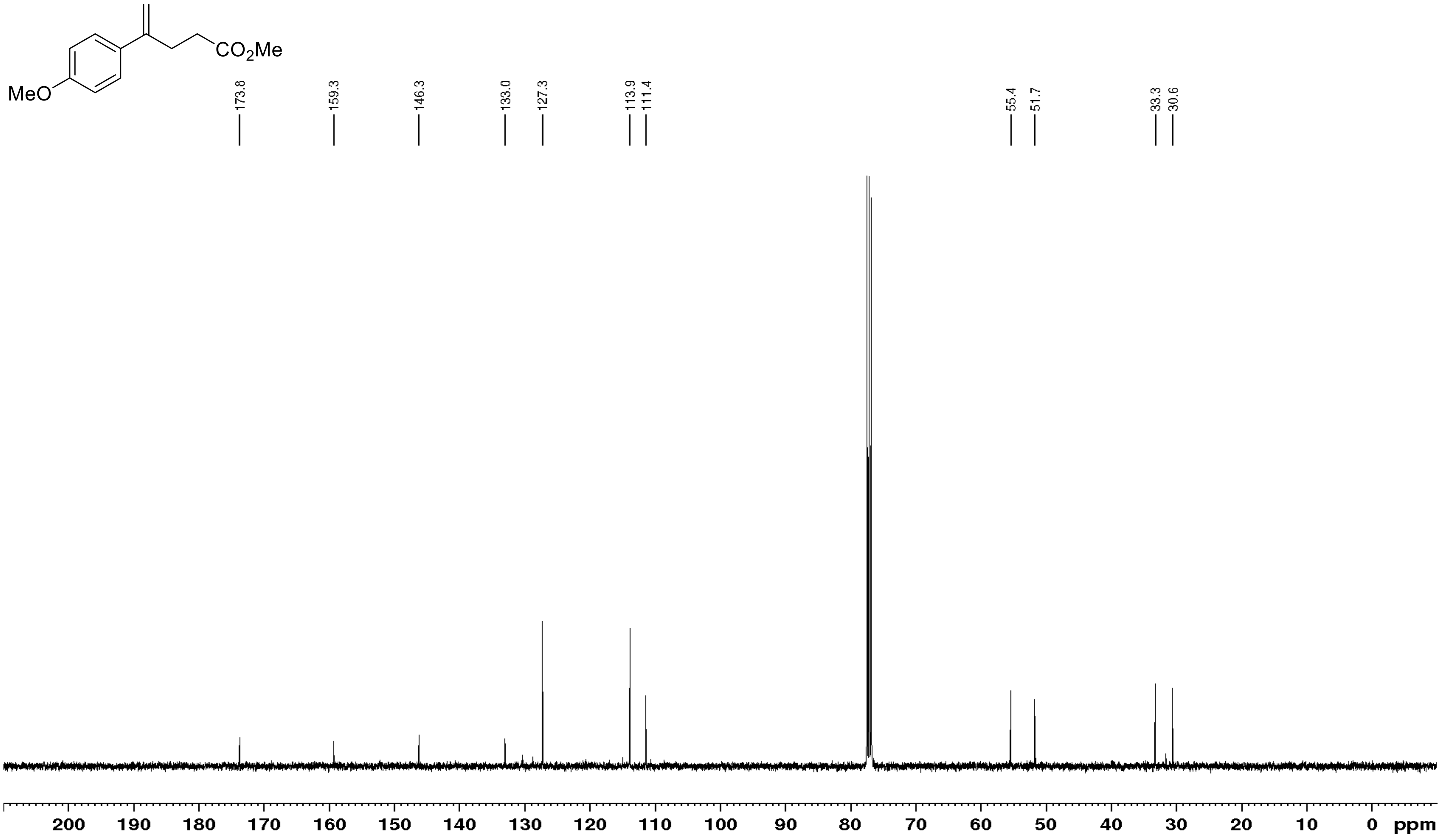

**Figure S61.** $^{1}$H NMR spectrum (400 MHz, $CDCl_3$, 298 K) of **4e**.

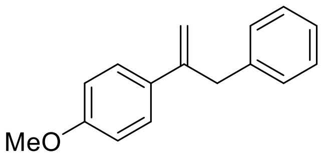


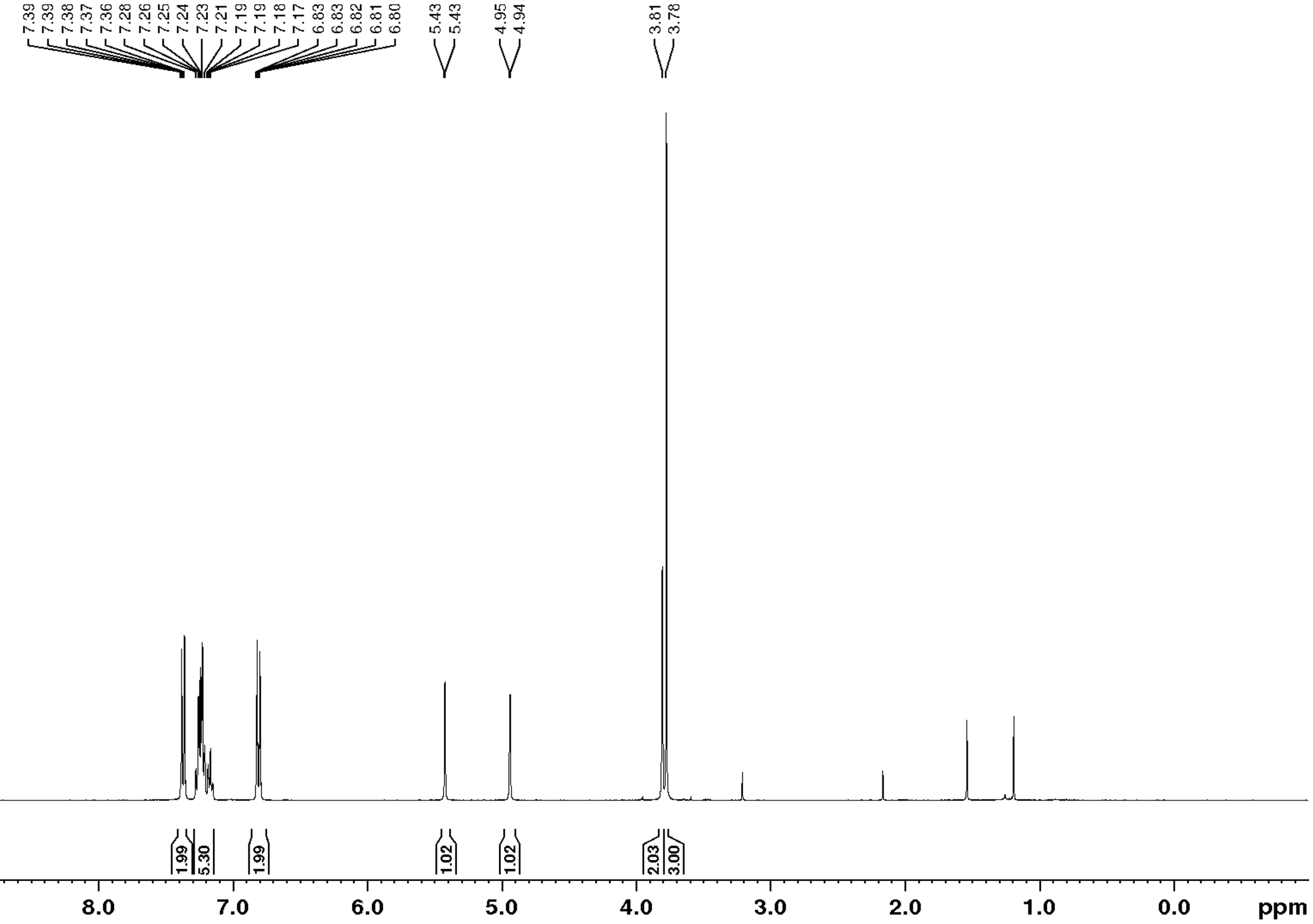

**Figure S62.** $^{13}C\{^1H\}$ NMR spectrum (100 MHz, $CDCl_3$, 298 K) of **4e**.

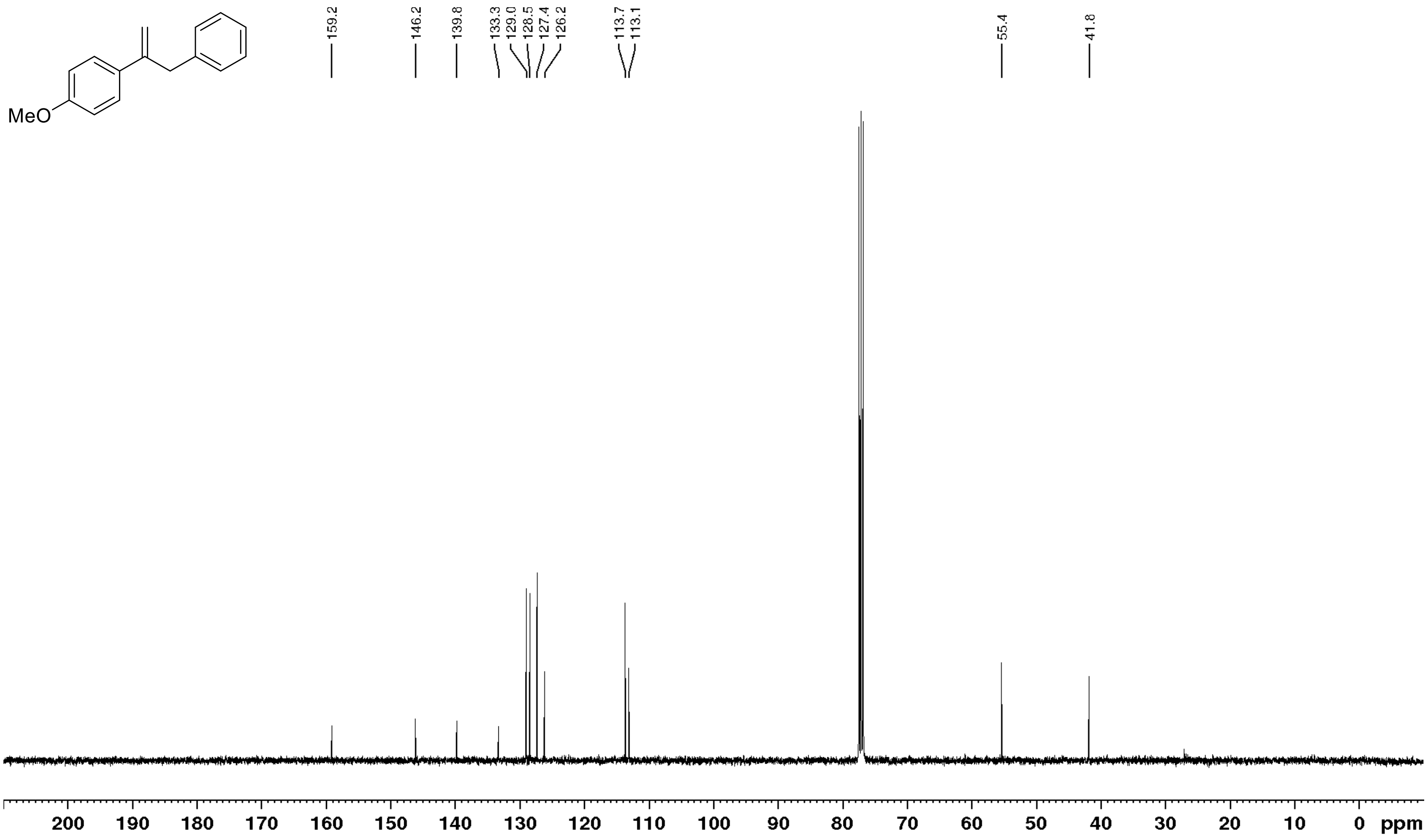

**Figure S63.** $^{1}$H NMR spectrum (400 MHz, $CDCl_3$, 298 K) of **4f**.

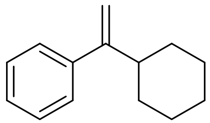

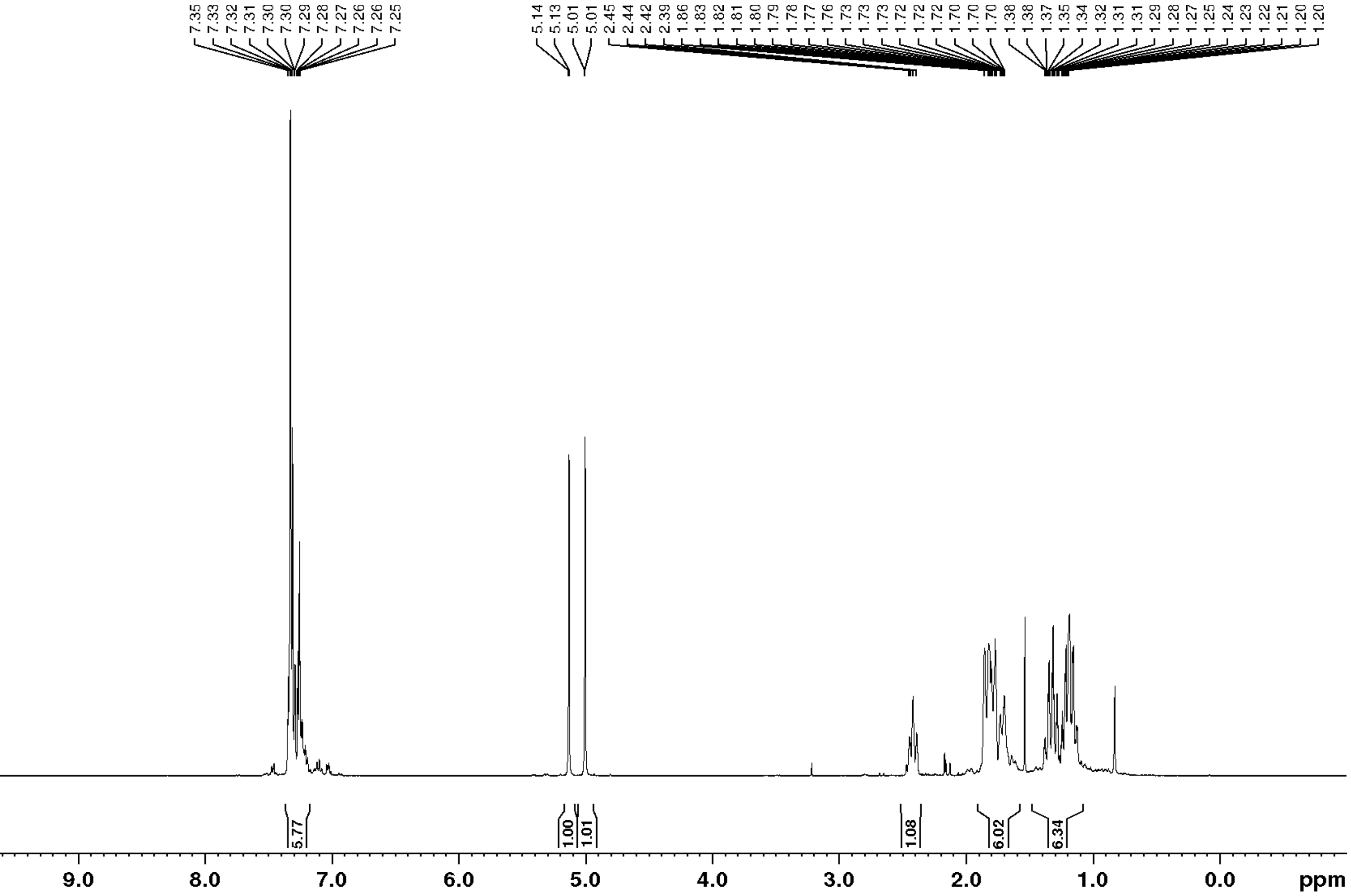

**Figure S64.** $^{13}C\{1H\}$ NMR spectrum (100 MHz, $CDCl_3$, 298 K) of **4f**.

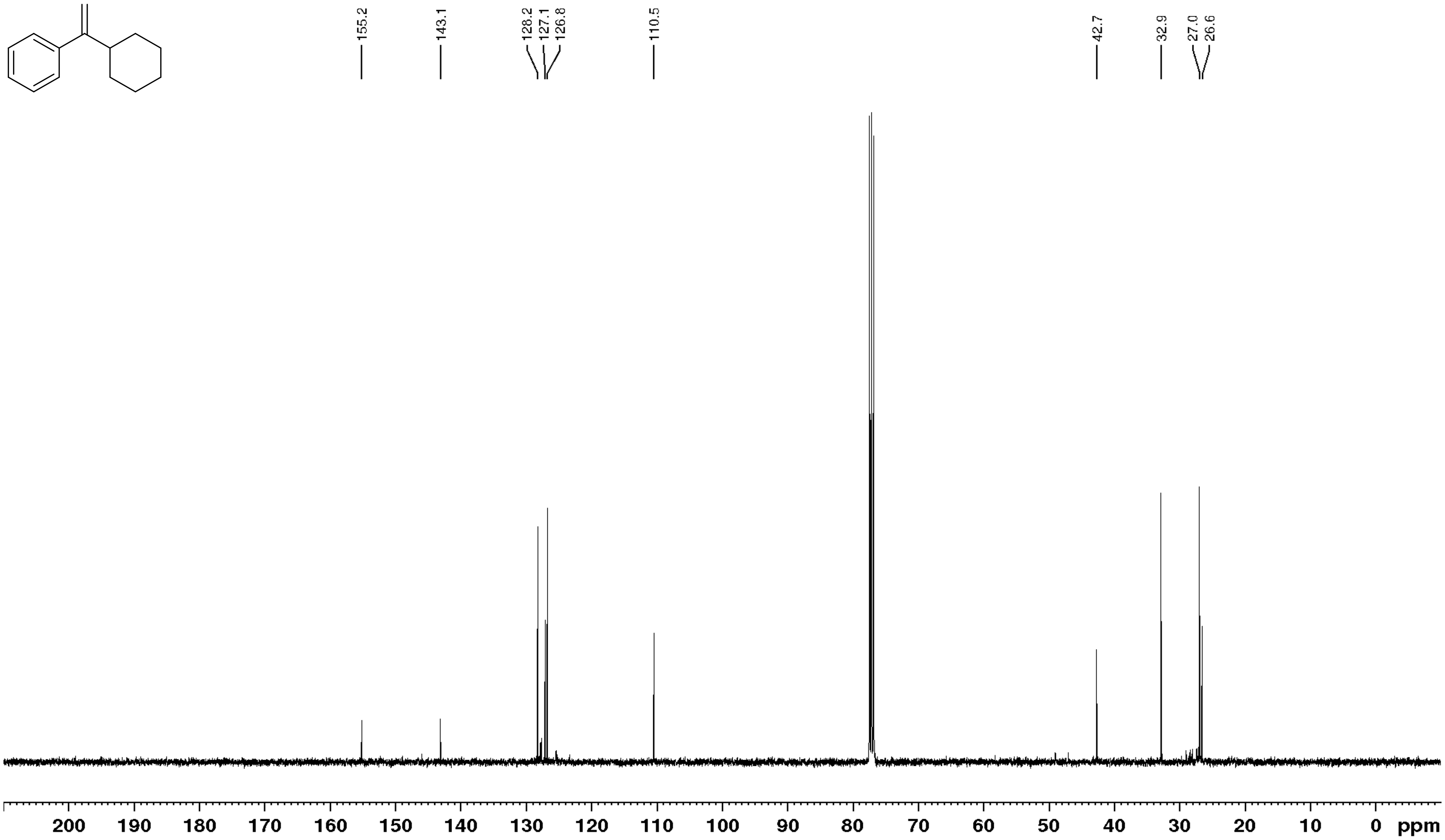

**Figure S65.** $^{1}$H NMR spectrum (400 MHz, $CDCl_3$, 298 K) of **1a**.

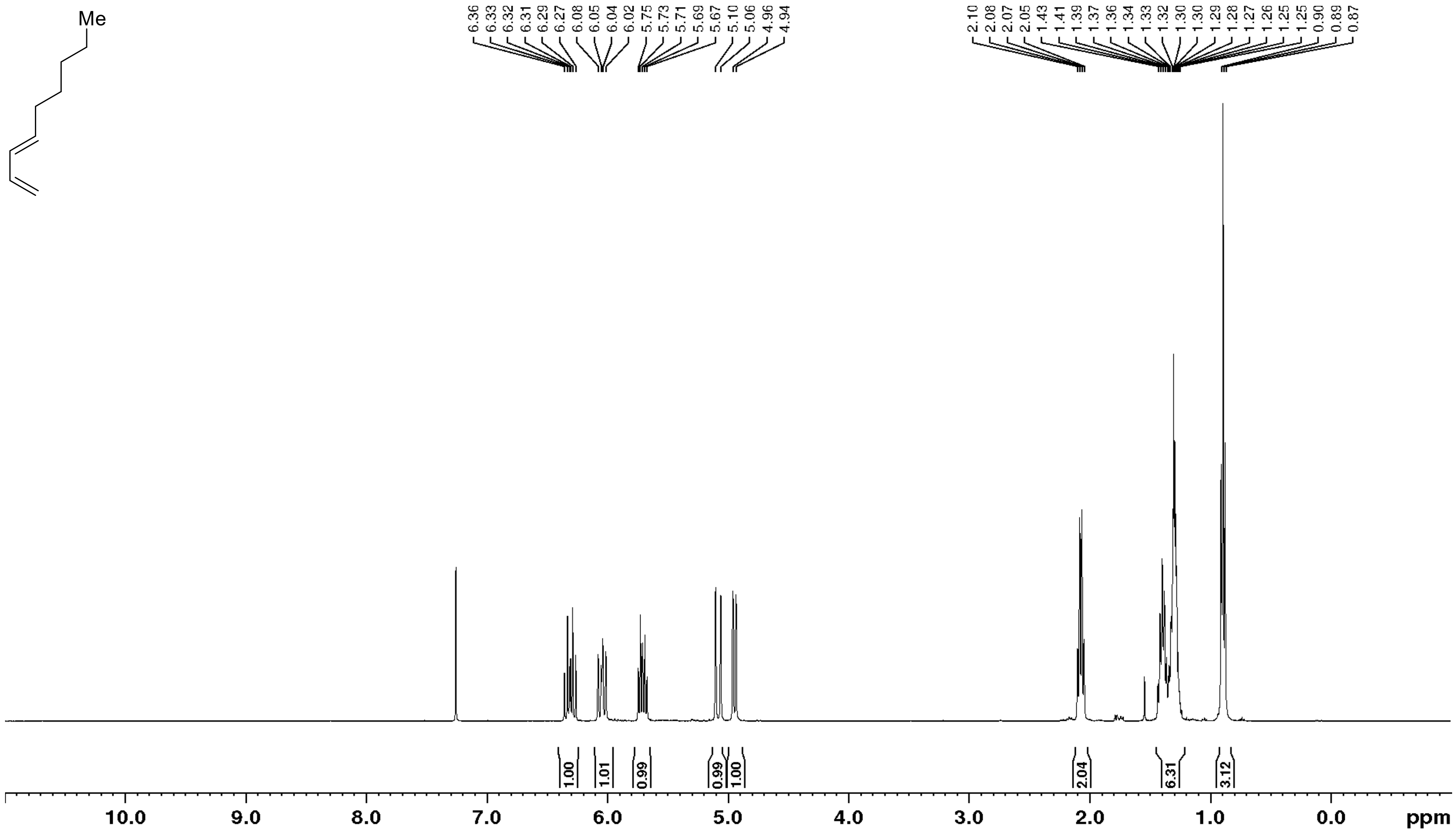

**Figure S66.** $^{13}C\{1H\}$ NMR spectrum (100 MHz, $CDCl_3$, 298 K) of **1a**.

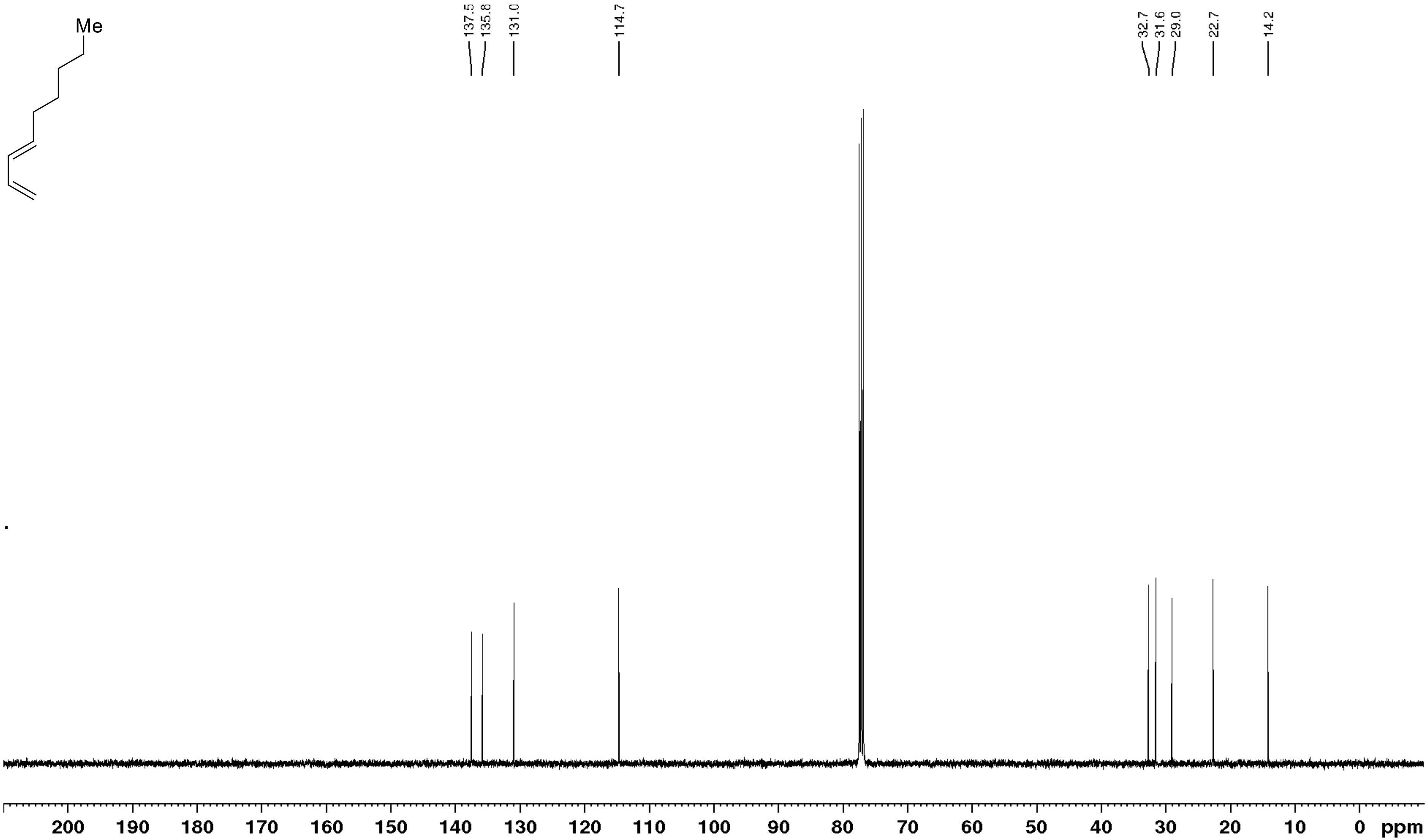

**Figure S67.** $^1$H NMR spectrum (400 MHz, $CDCl_3$, 298 K) of **1c**.

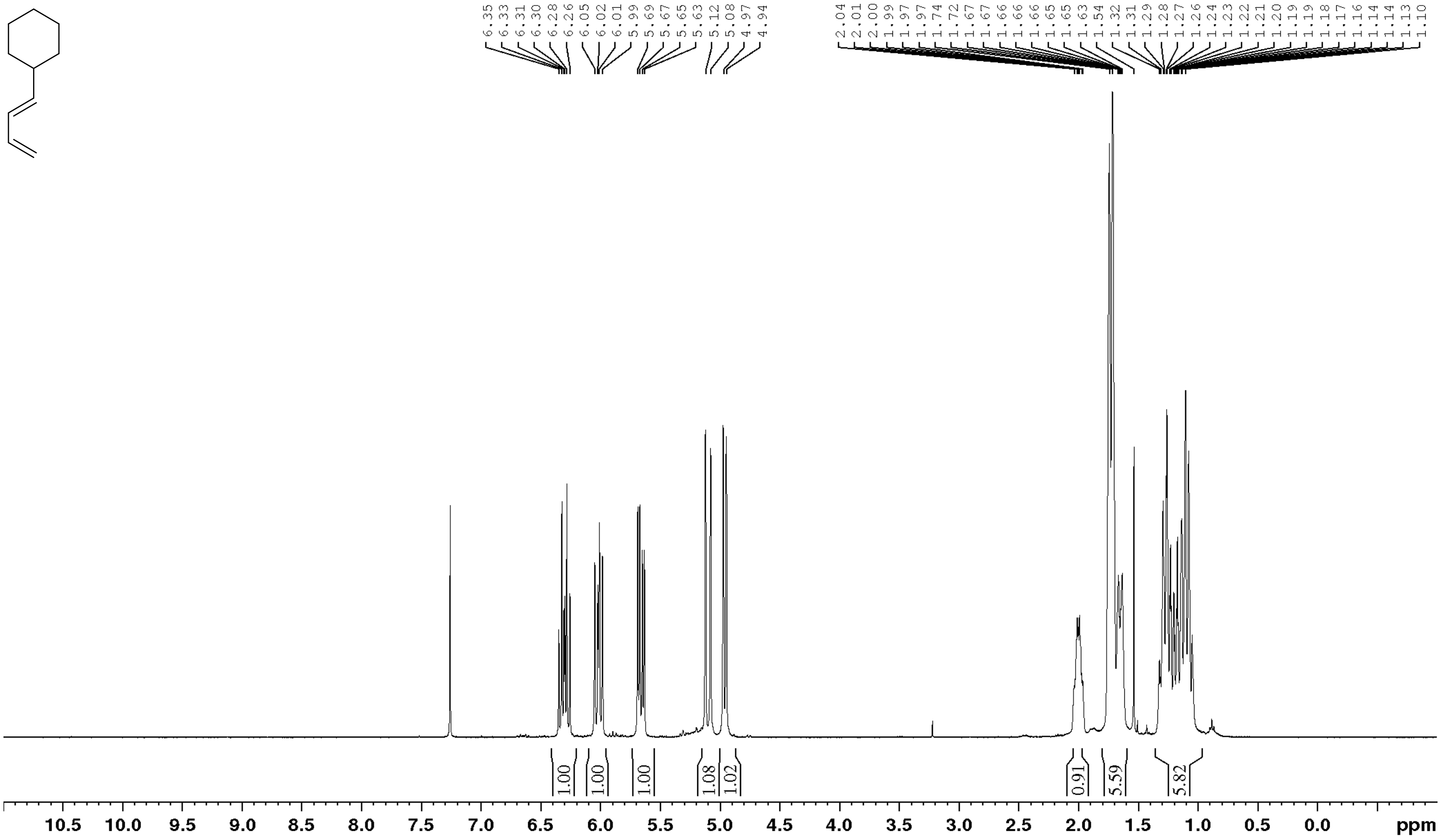

**Figure S68.** $^{13}C\{1H\}$ NMR spectrum (100 MHz, $CDCl_3$, 298 K) of **1c**.

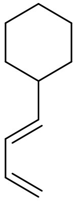

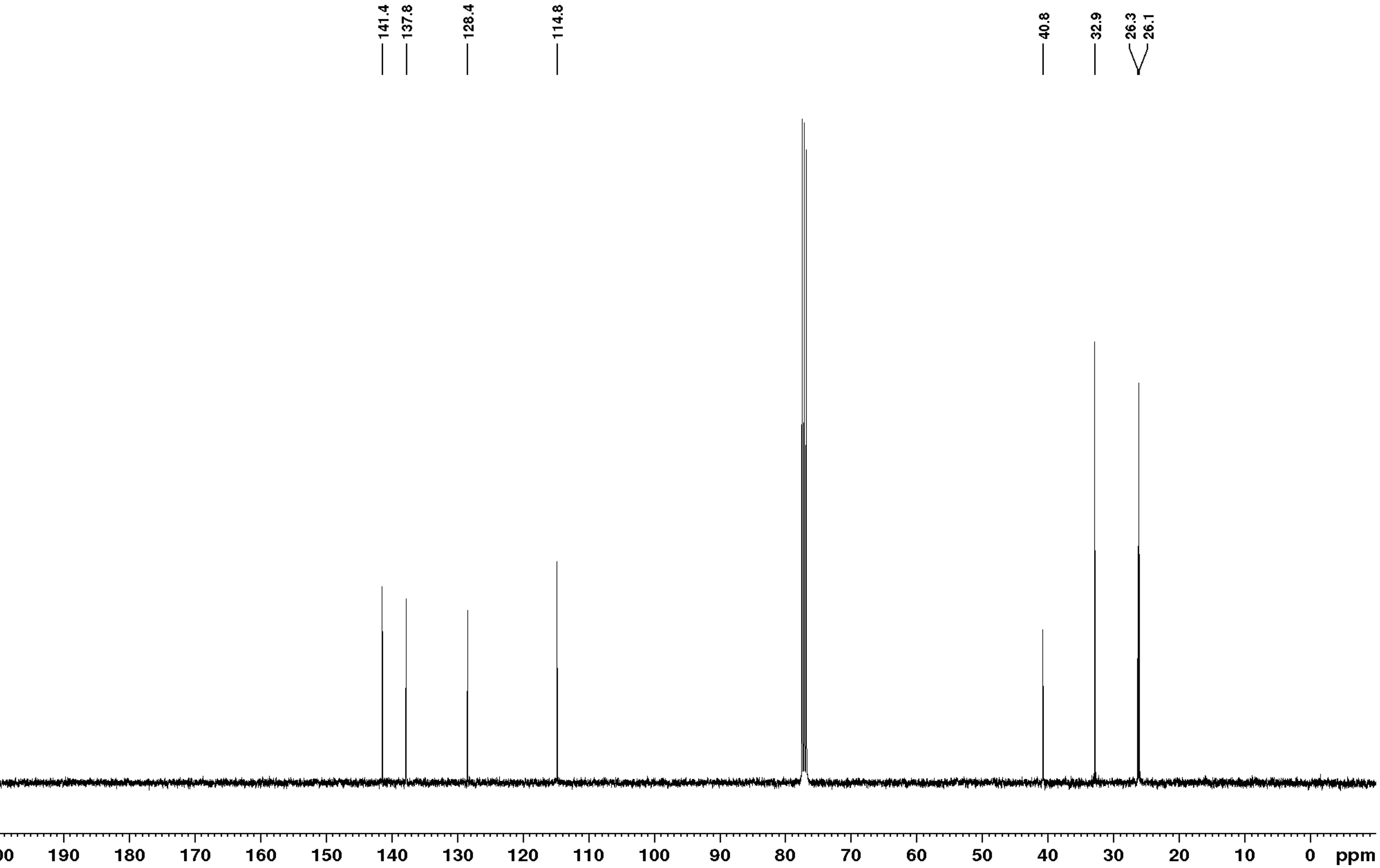

**Figure S69.** $^{1}$H NMR spectrum (400 MHz, $CDCl_3$, 298 K) of **S2a**.

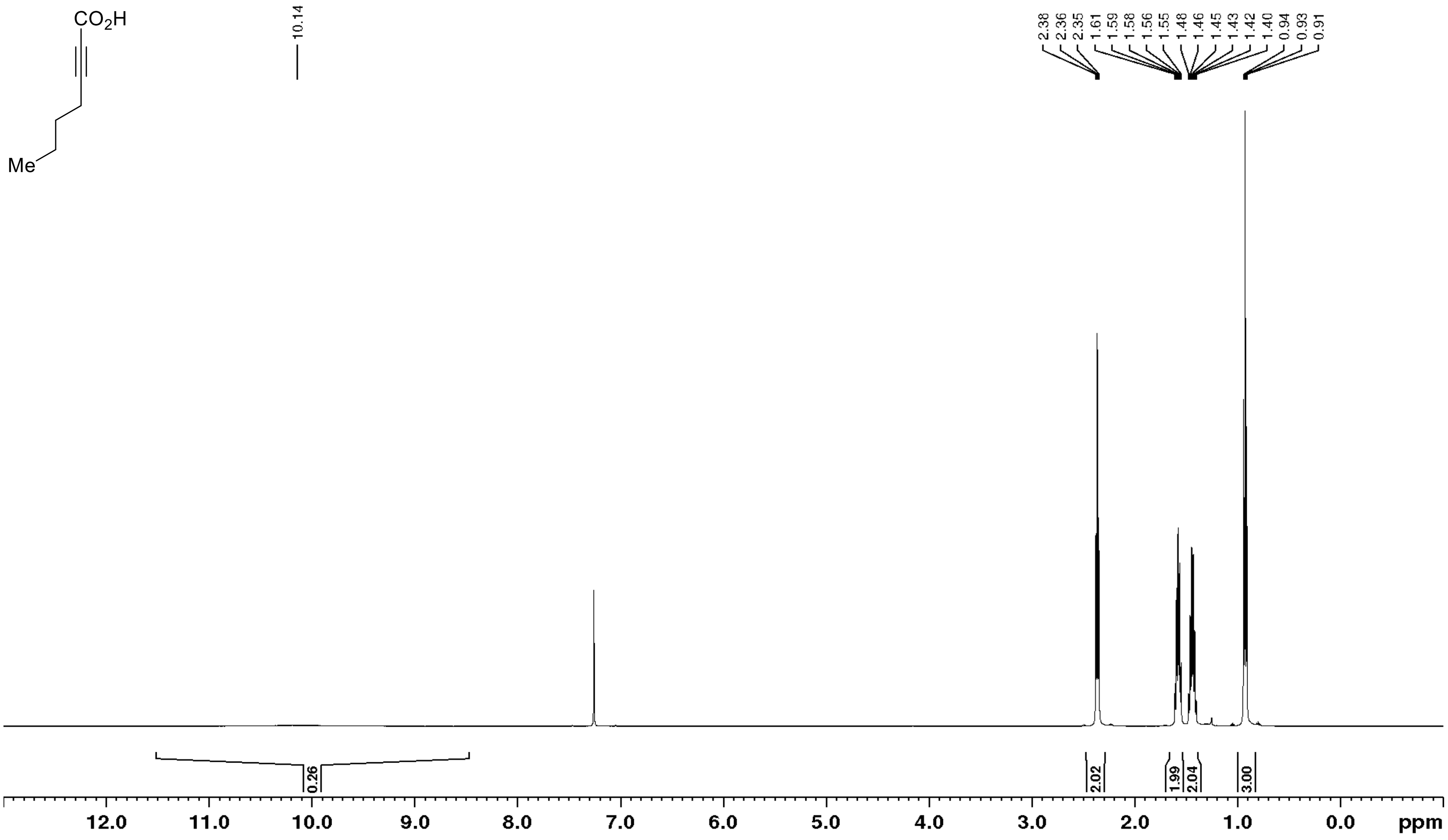

**Figure S70.** $^{13}C\{^1H\}$ NMR spectrum (100 MHz, $CDCl_3$, 298 K) of **S2a**.

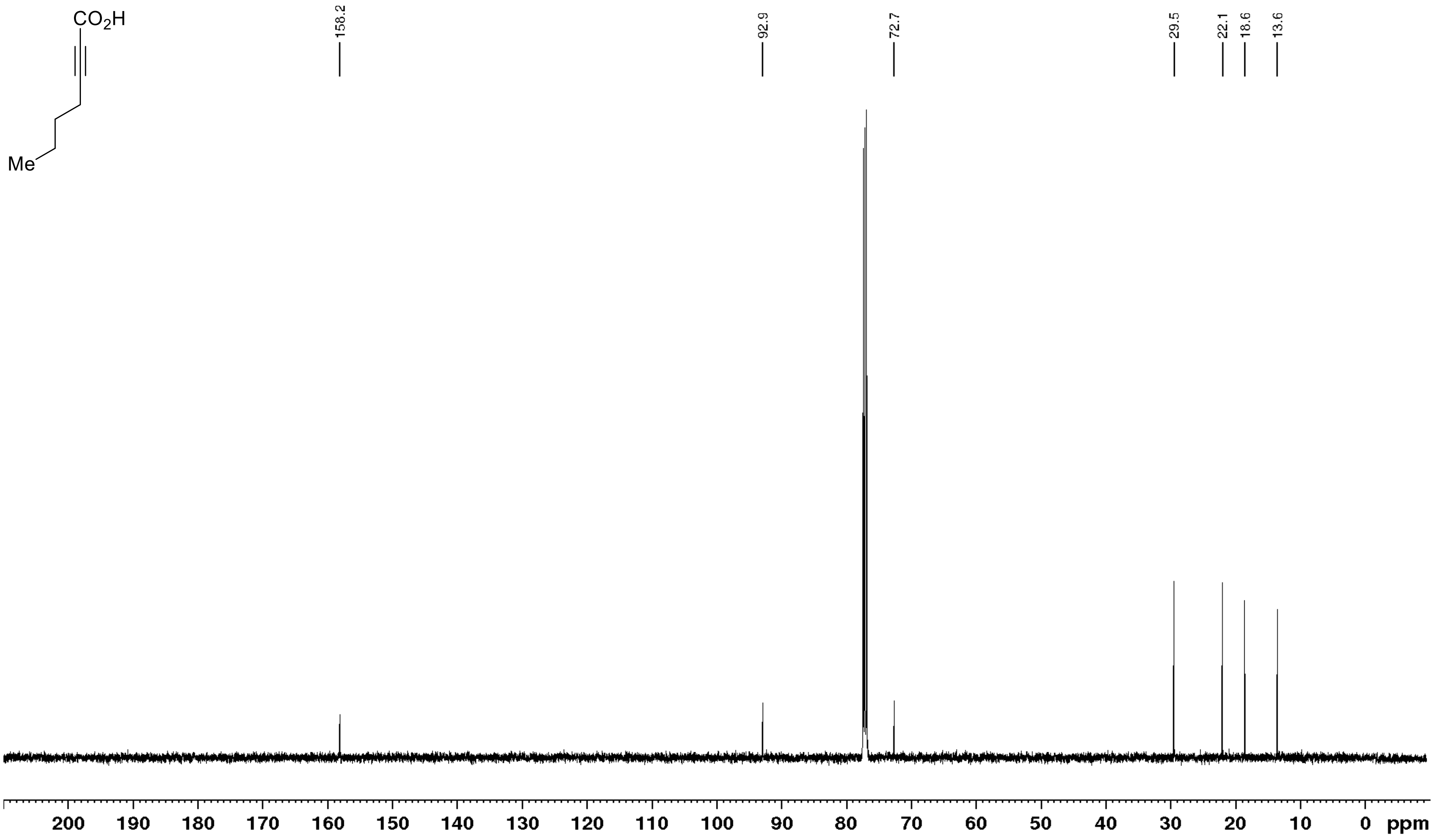

**Figure S71.** $^{1}$H NMR spectrum (400 MHz, $CDCl_3$, 298 K) of **S2b**.

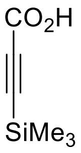


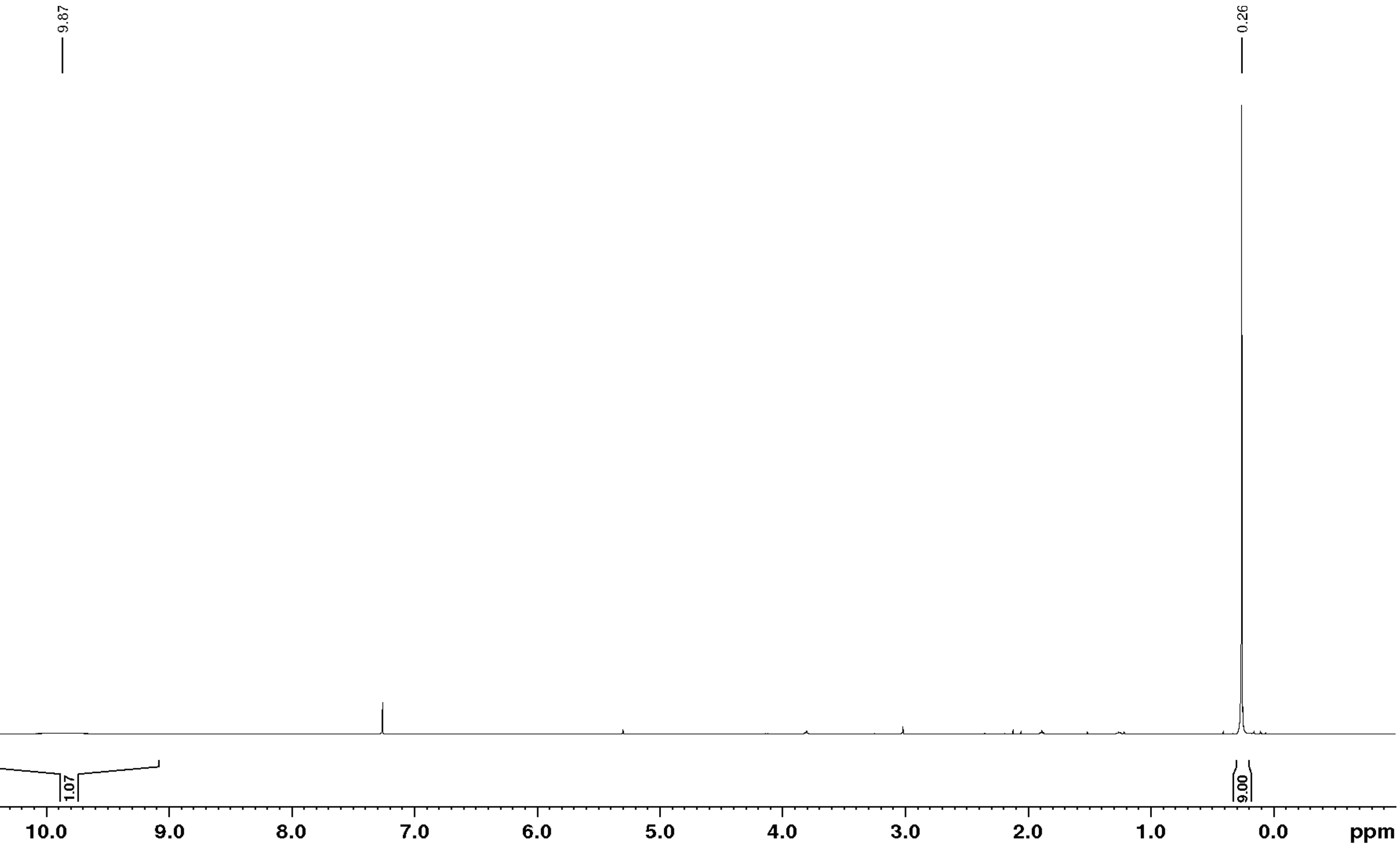

**Figure S72.** $^{13}C\{^1H\}$ NMR spectrum (100 MHz, $CDCl_3$, 298 K) of **S2b**.

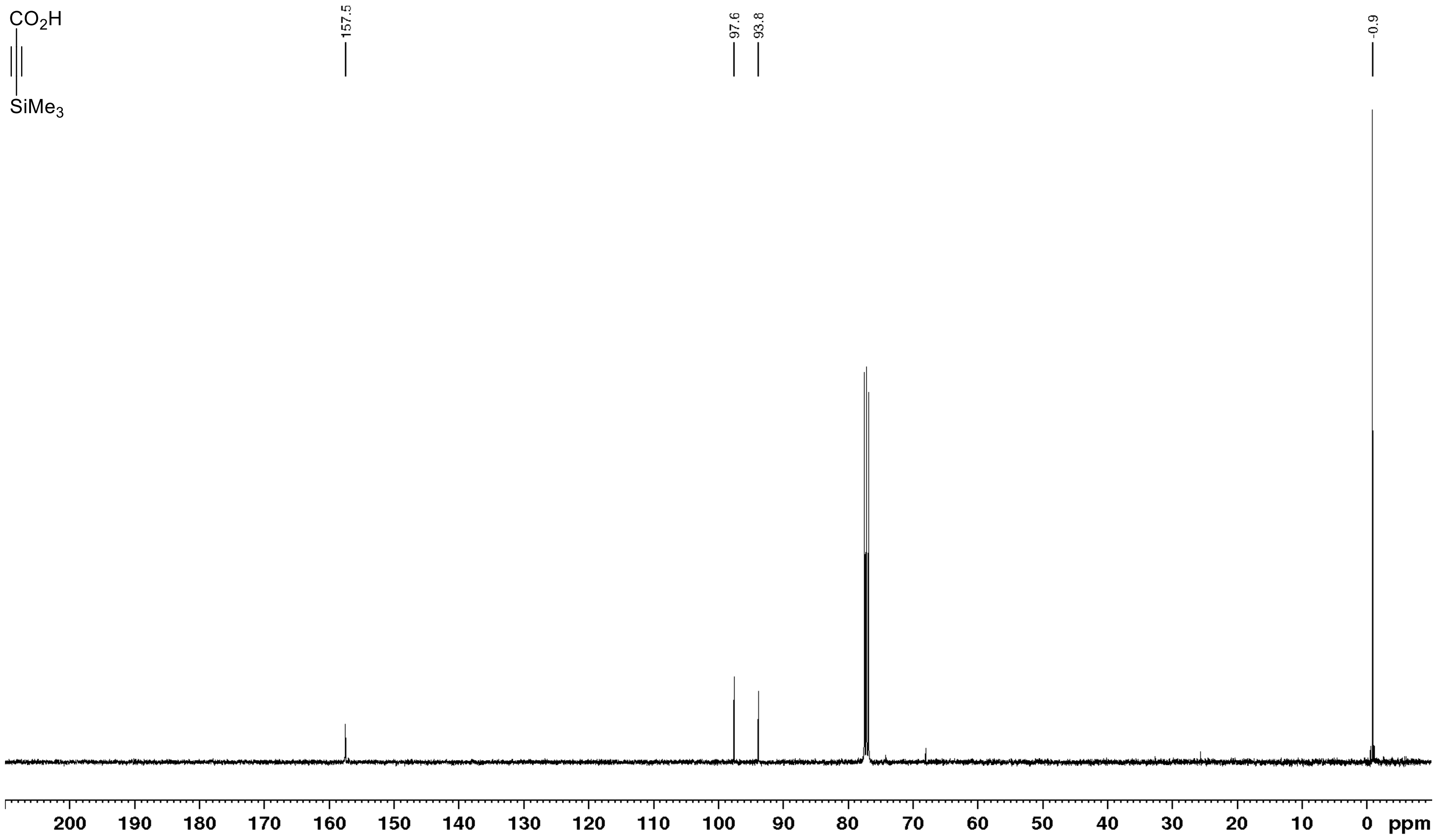

**Figure S73.** $^{1}$H NMR spectrum (400 MHz, $CDCl_3$, 298 K) of **2a**.

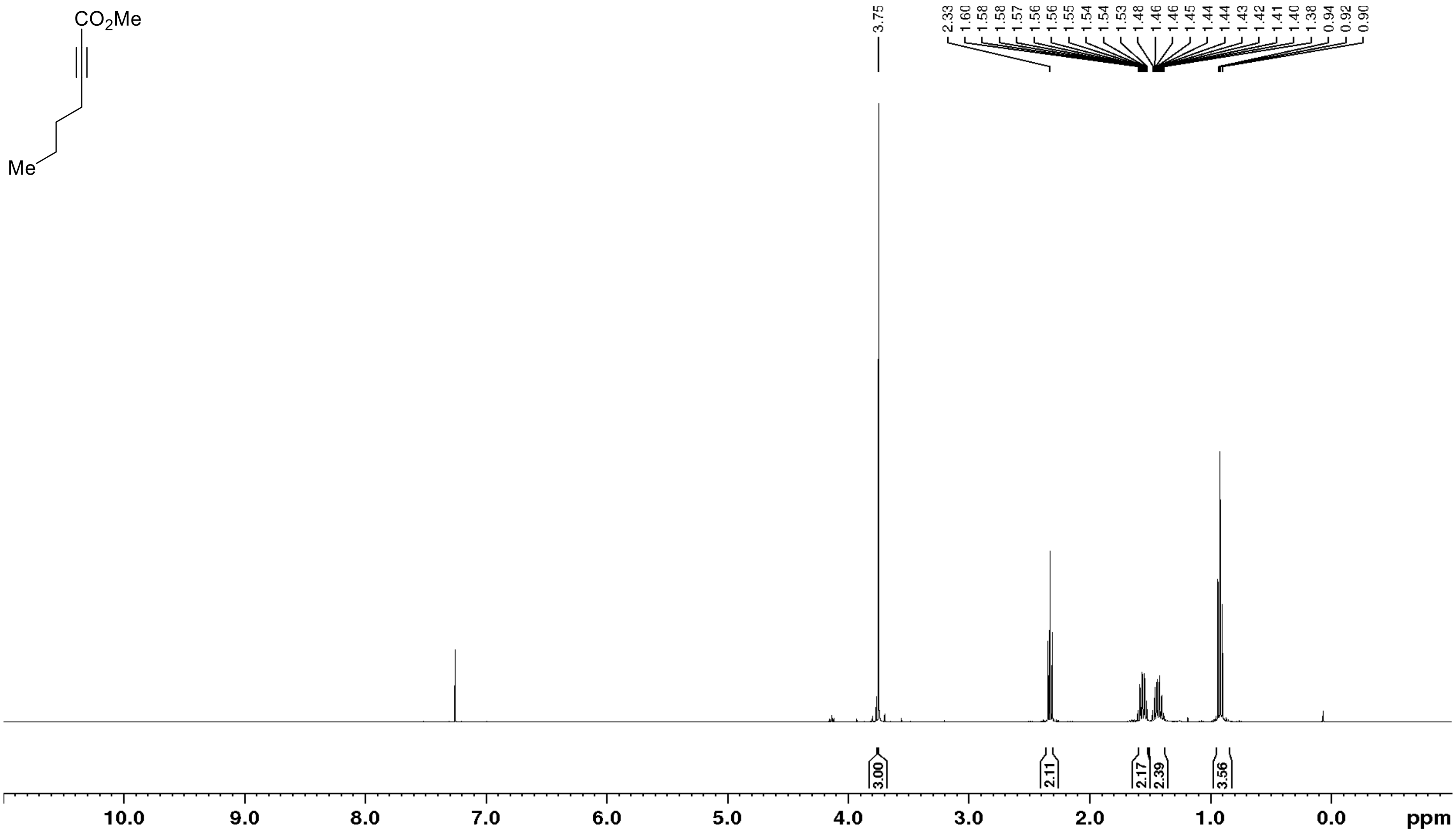

**Figure S74.** $^{13}C\{1H\}$ NMR spectrum (100 MHz, $CDCl_3$, 298 K) of **2a**.

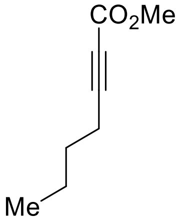


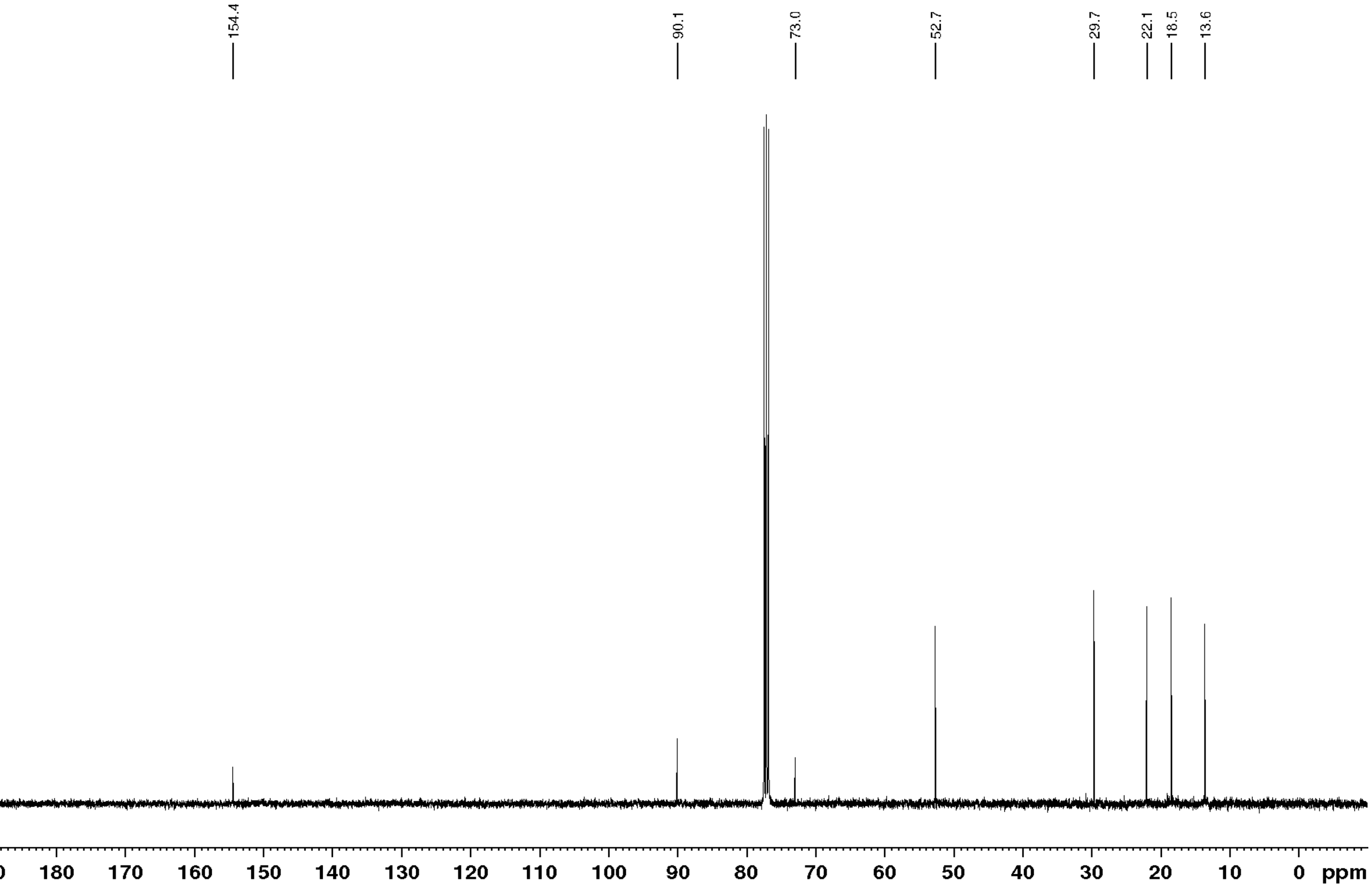

**Figure S75.** $^1$H NMR spectrum (400 MHz, $CDCl_3$, 298 K) of **2b**.

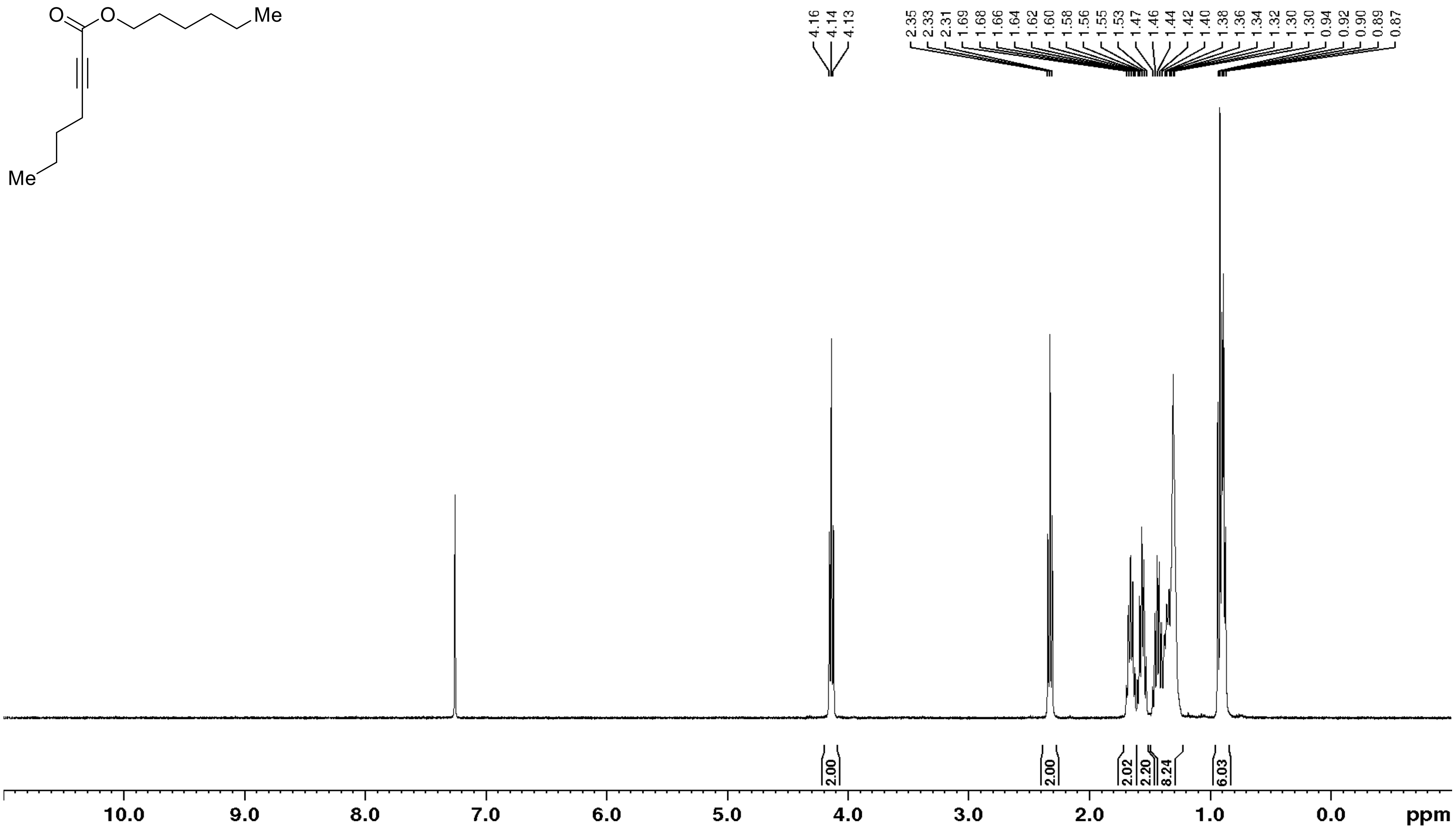

**Figure S76.** $^{13}$C{1H} NMR spectrum (100 MHz, $CDCl_3$, 298 K) of **2b**.

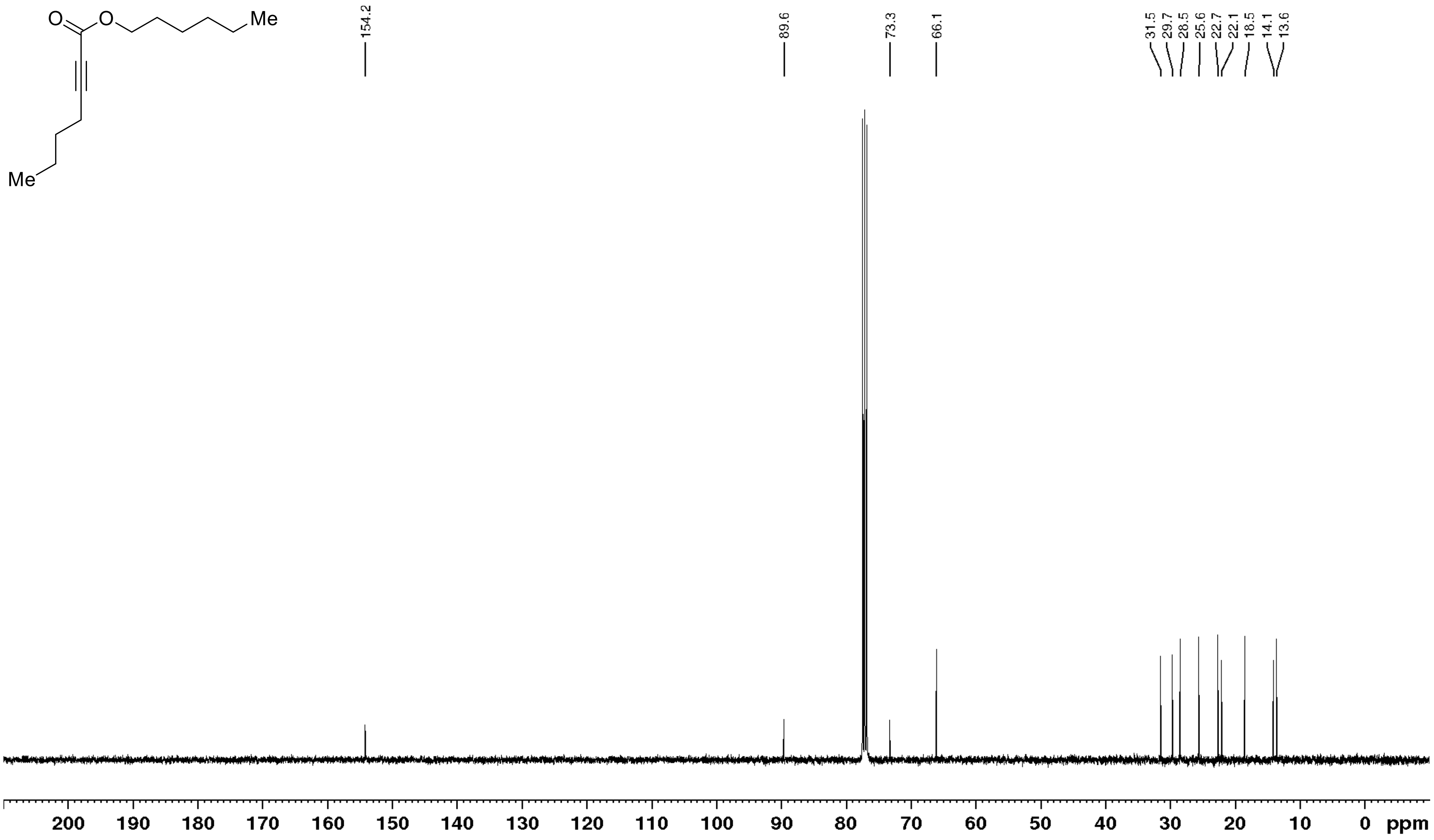

**Figure S77.** $^{1}H$ NMR spectrum (400 MHz, $CDCl_3$, 298 K) of **2c**.

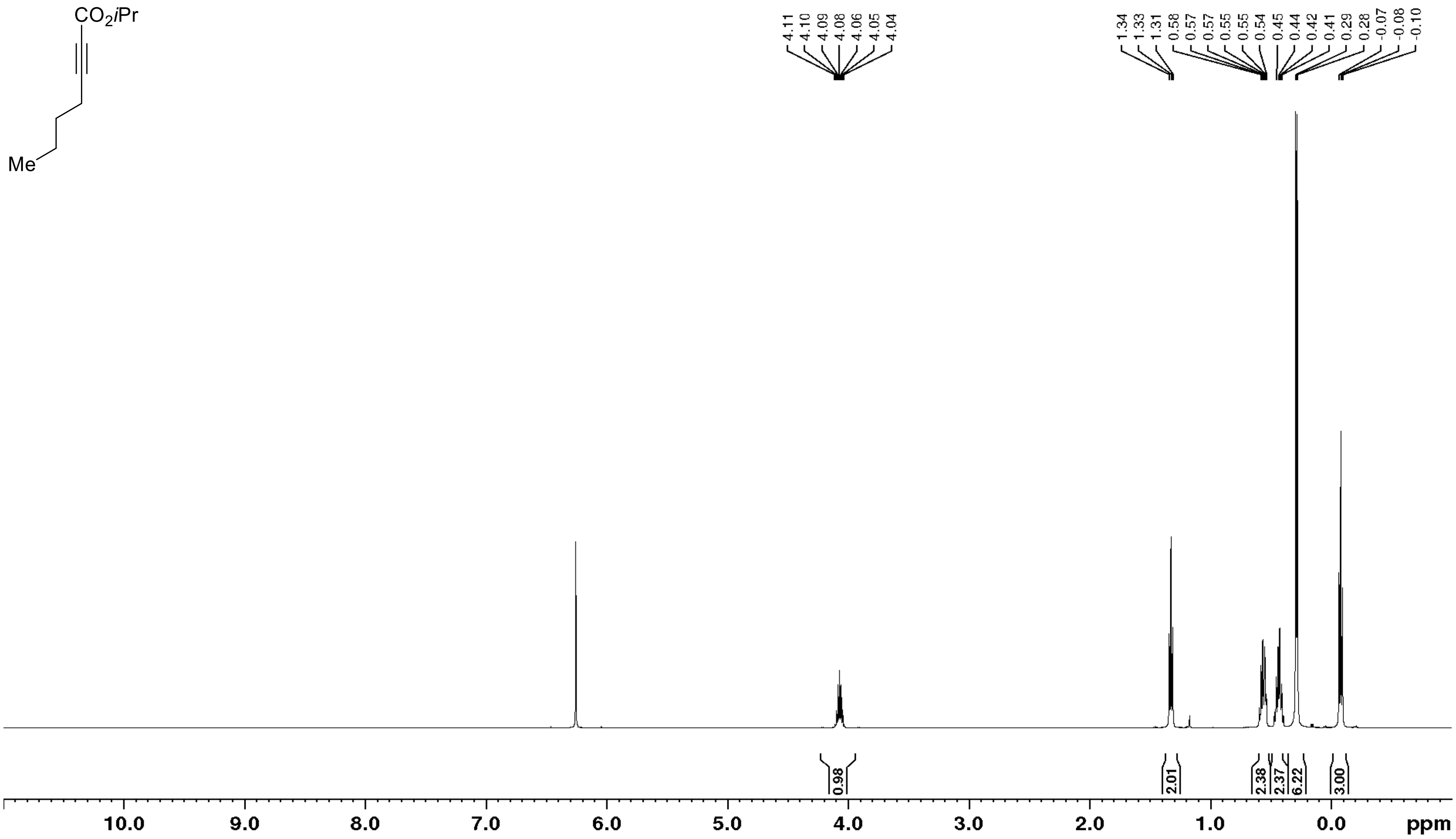

**Figure S78.** $^{13}C\{^1H\}$ NMR spectrum (100 MHz, $CDCl_3$, 298 K) of **2c**.

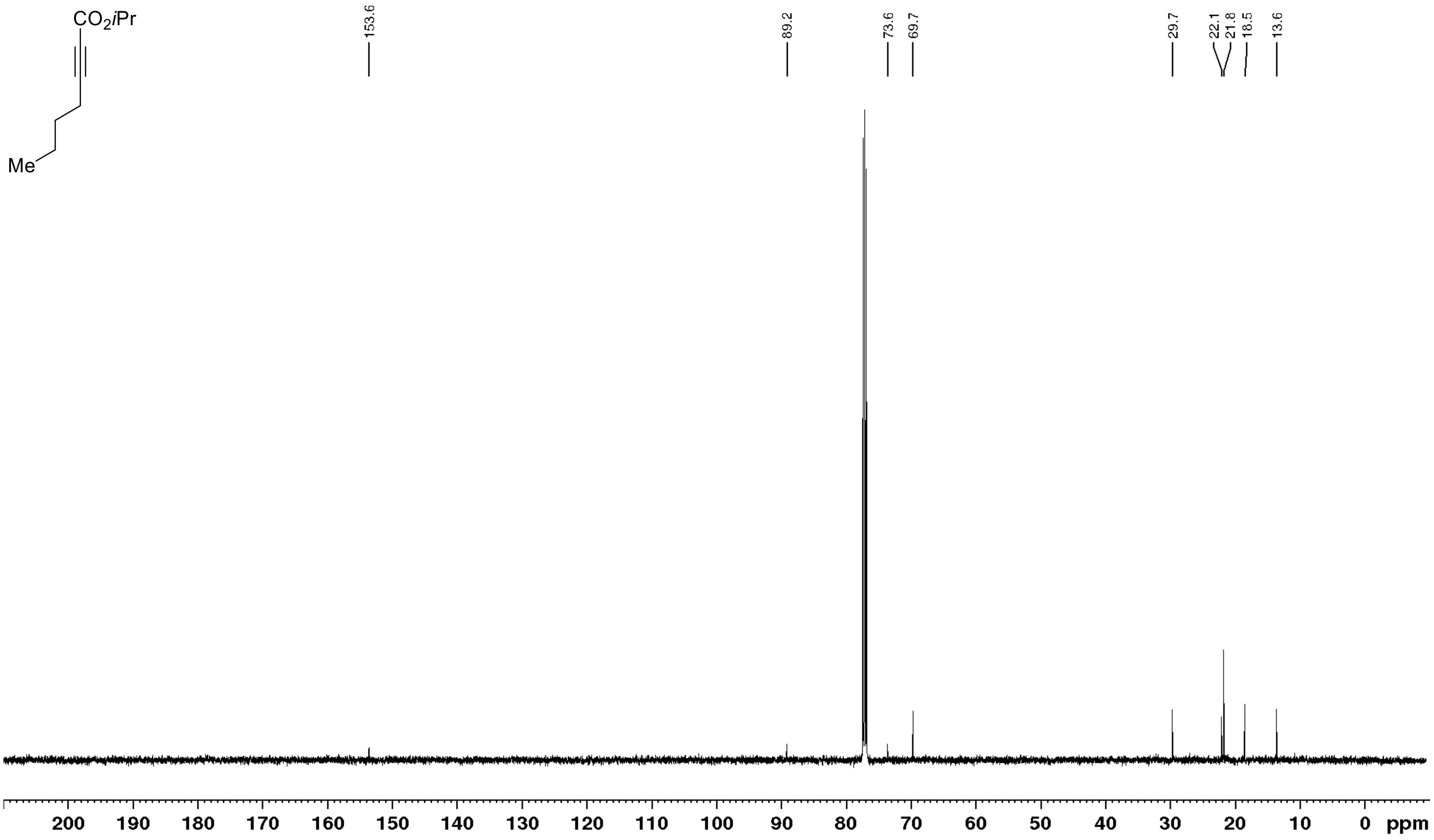

**Figure S79.** $^{1}H$ NMR spectrum (400 MHz, $CDCl_3$, 298 K) of **2d**.

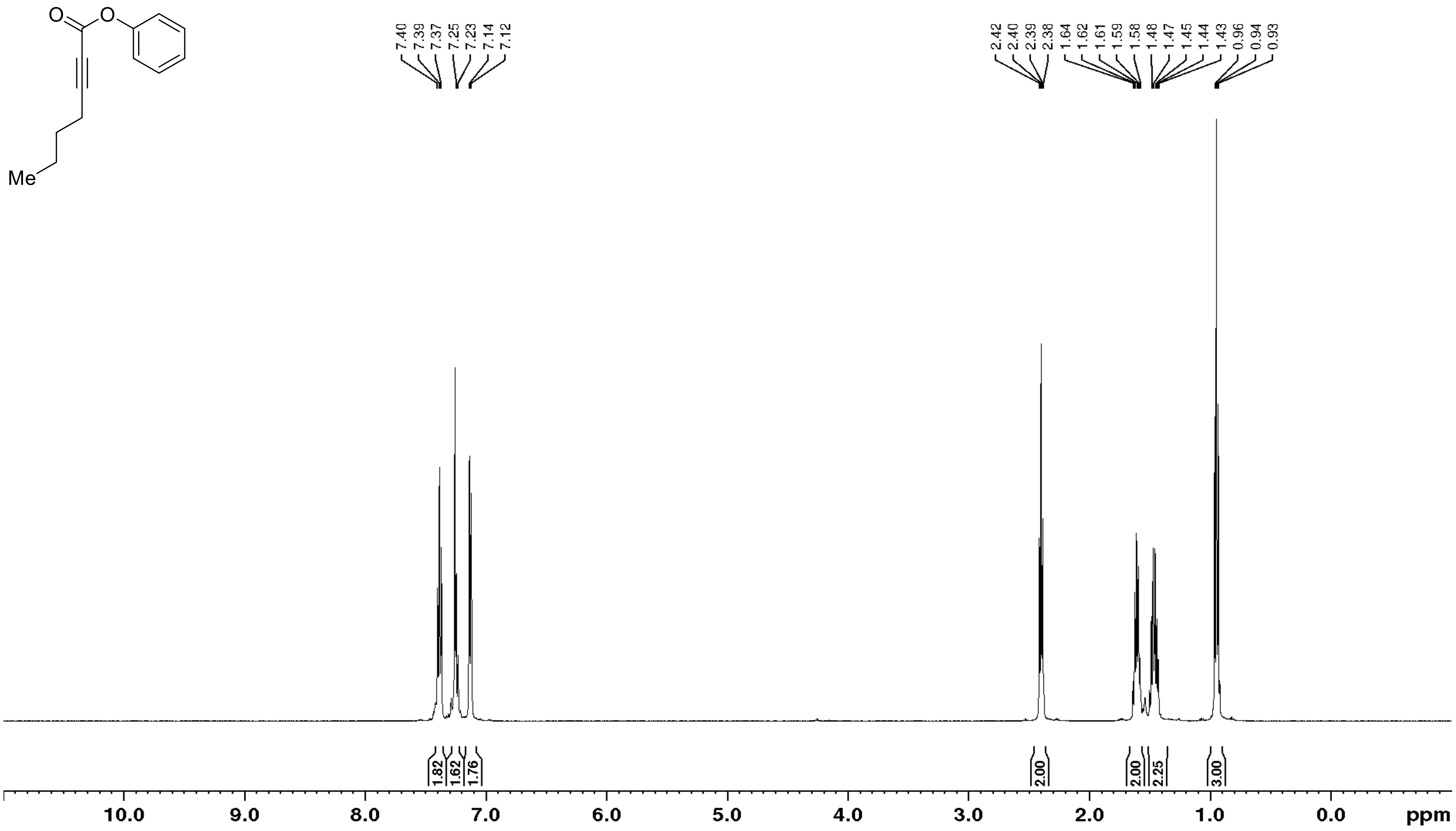

**Figure S80.** $^{13}C\{1H\}$ NMR spectrum (100 MHz, $CDCl_3$, 298 K) of **2d**.

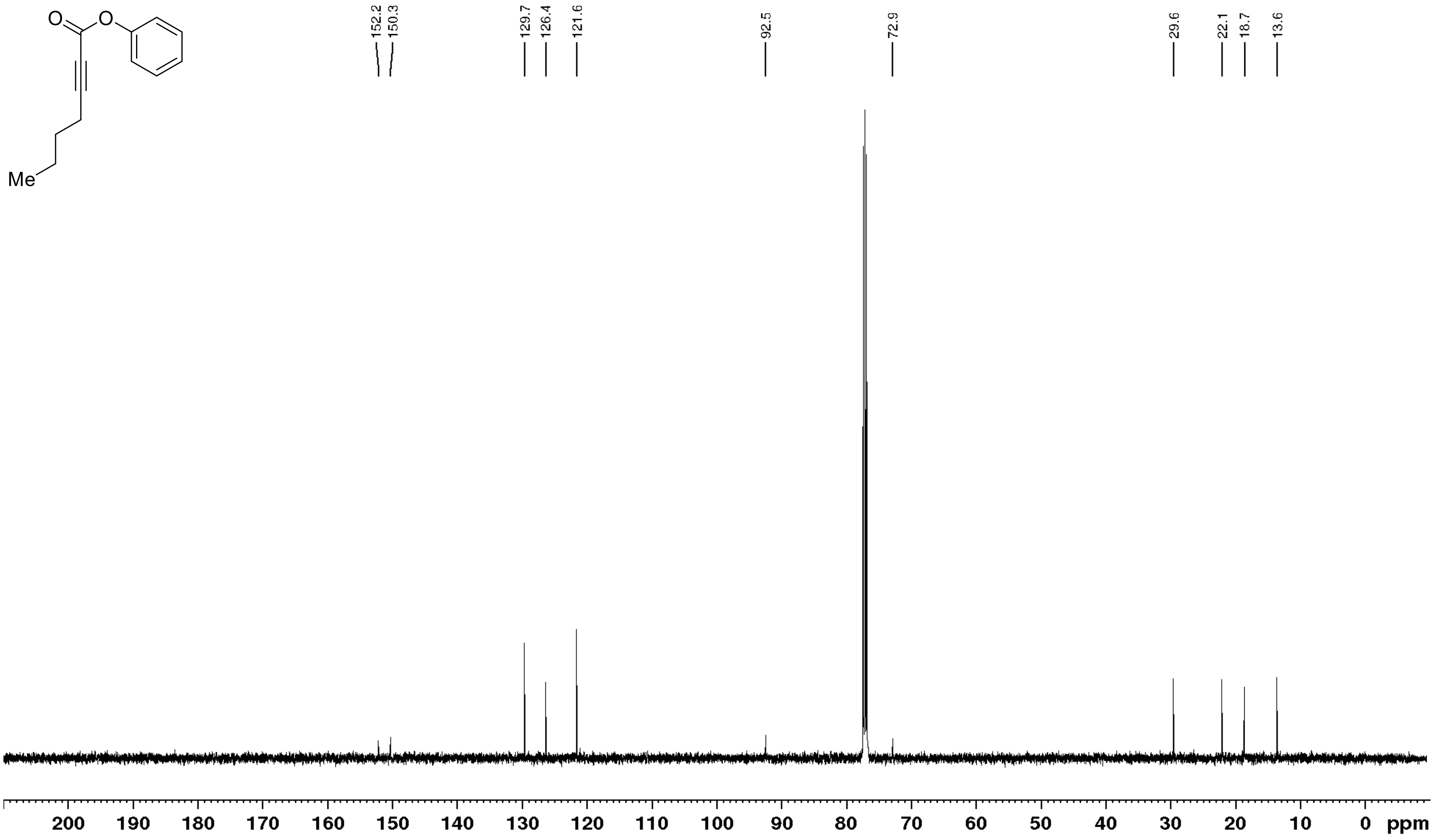

**Figure S81.** $^1$H NMR spectrum (400 MHz, $CDCl_3$, 298 K) of **2e**.

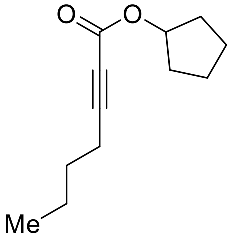


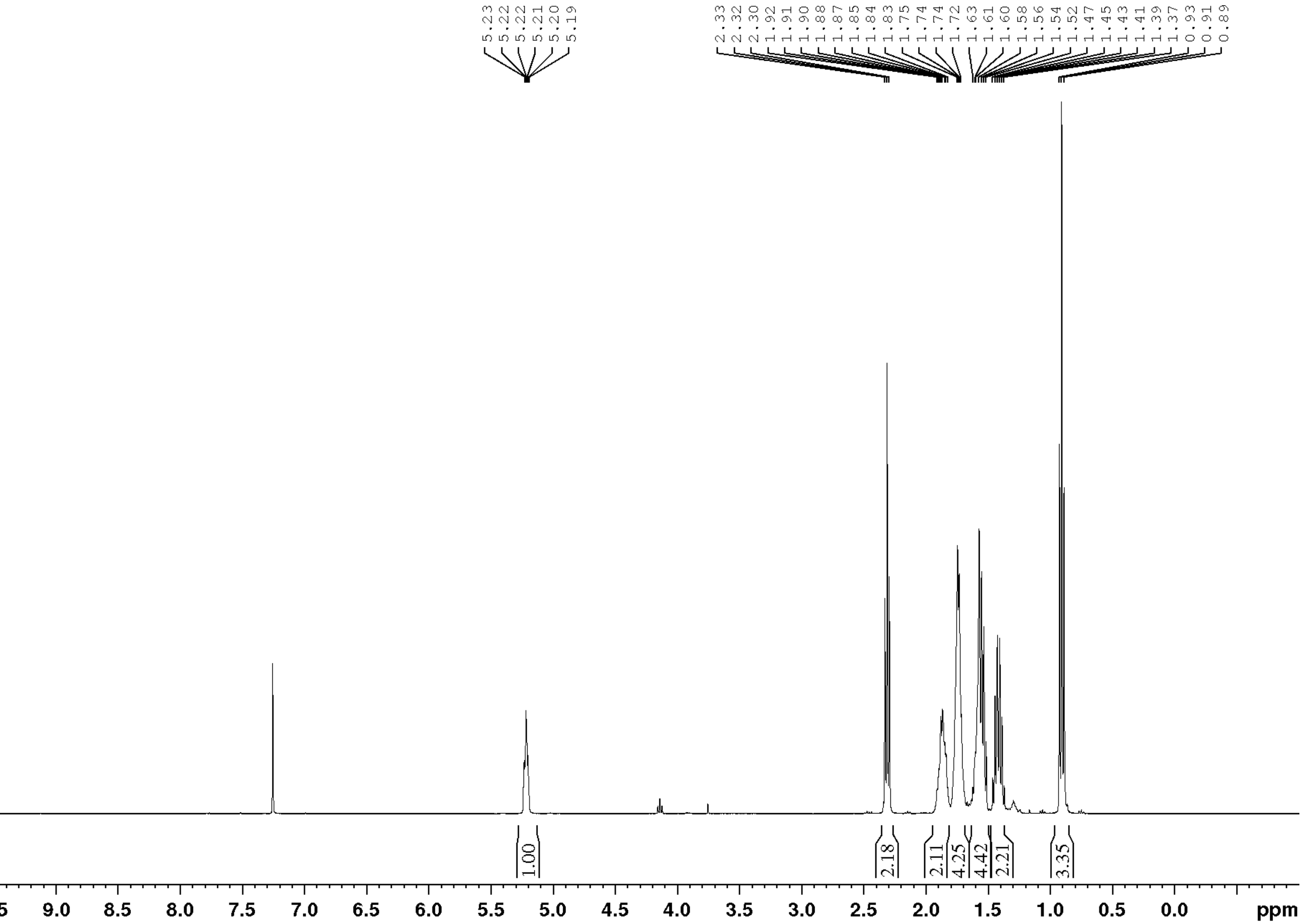

**Figure S82.** $^{13}C\{1H\}$ NMR spectrum (100 MHz, $CDCl_3$, 298 K) of **2e**.

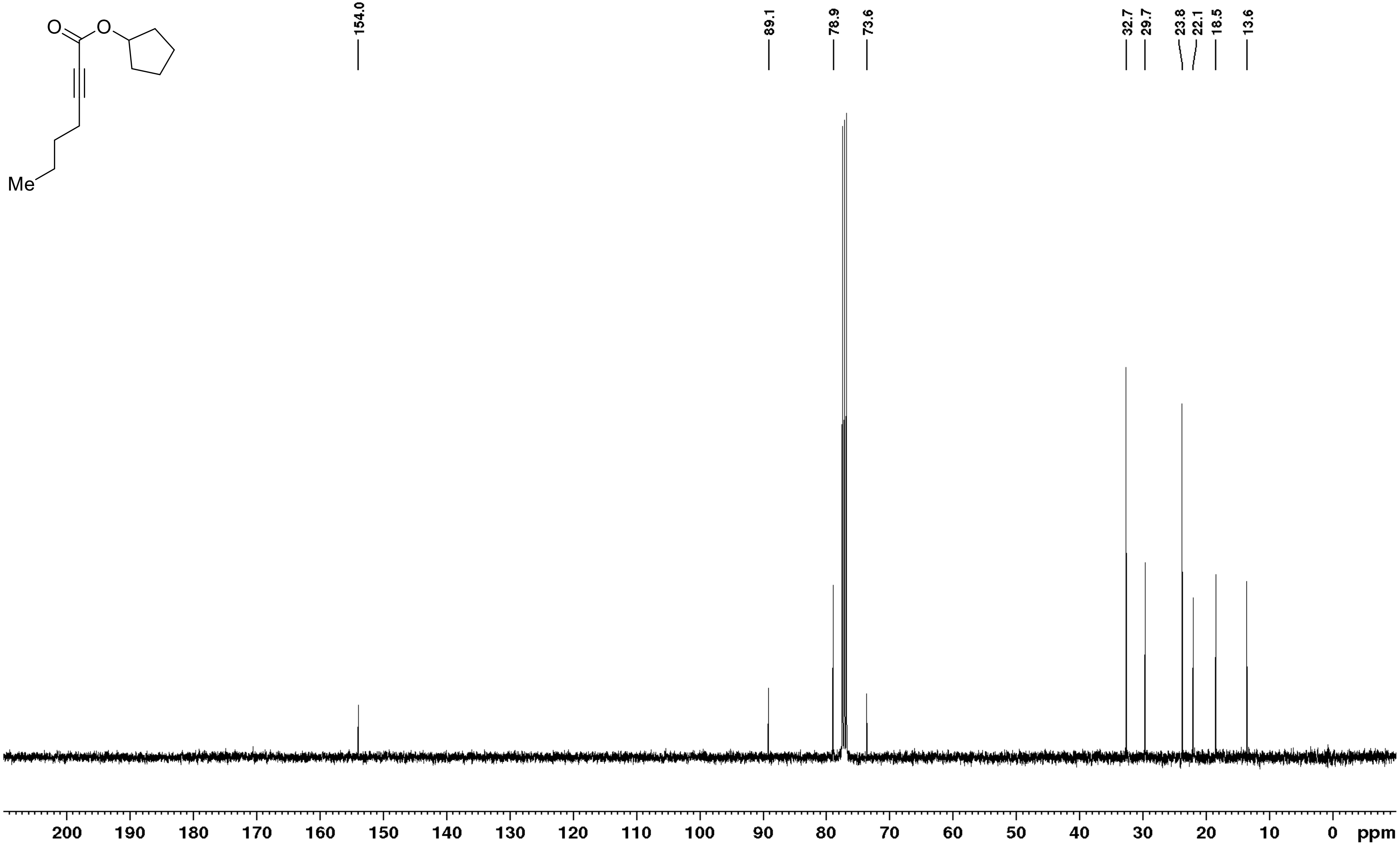

**Figure S83.** $^{1}H$ NMR spectrum (400 MHz, $CDCl_3$, 298 K) of **2f**.

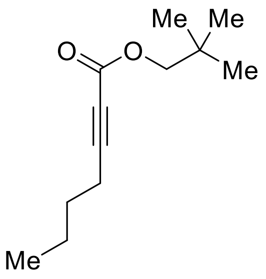


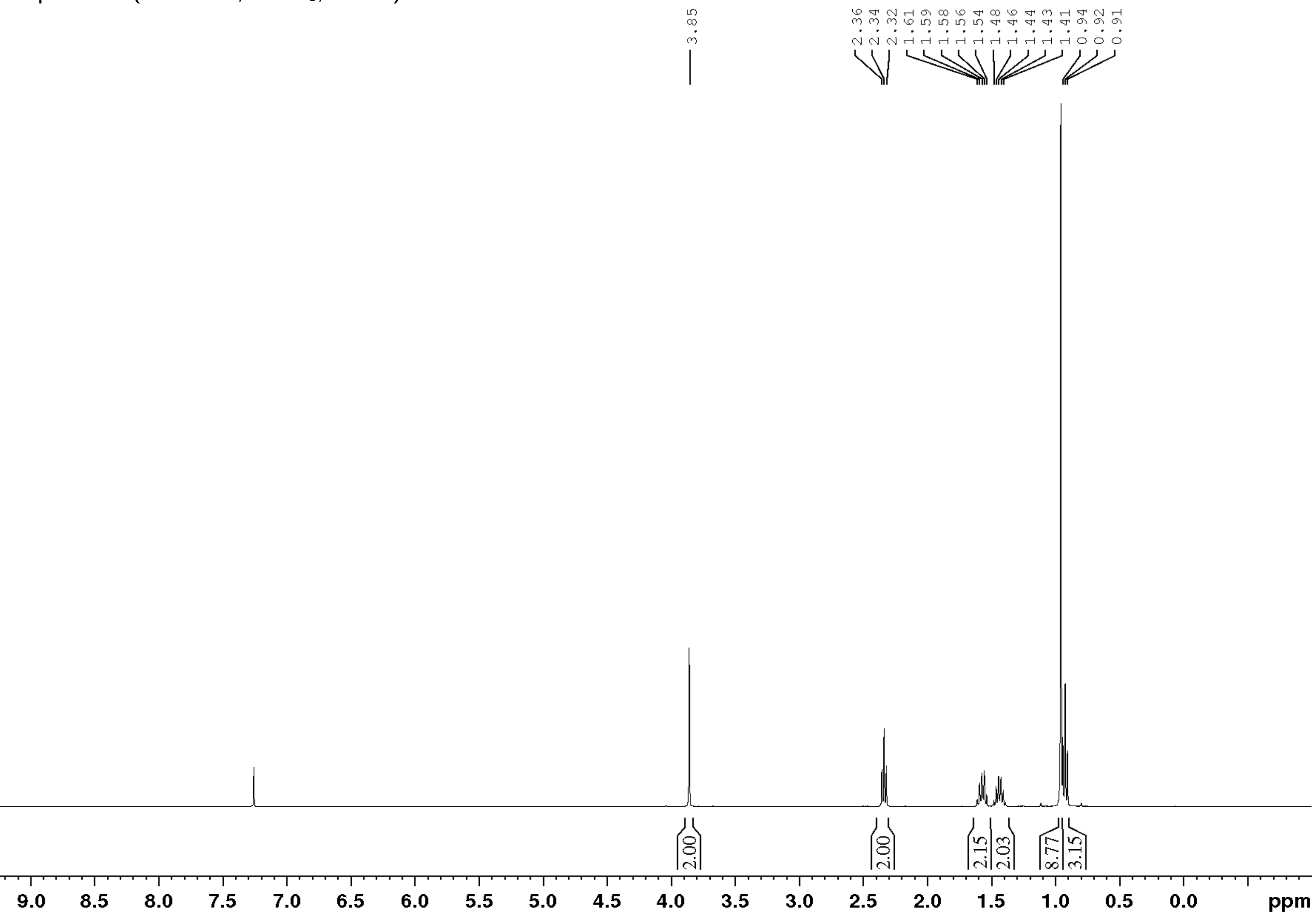

**Figure S84.** $^{13}C\{1H\}$ NMR spectrum (100 MHz, $CDCl_3$, 298 K) of **2f**.

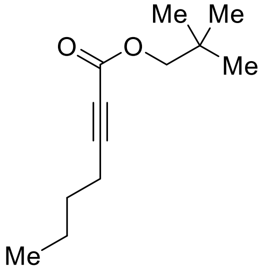


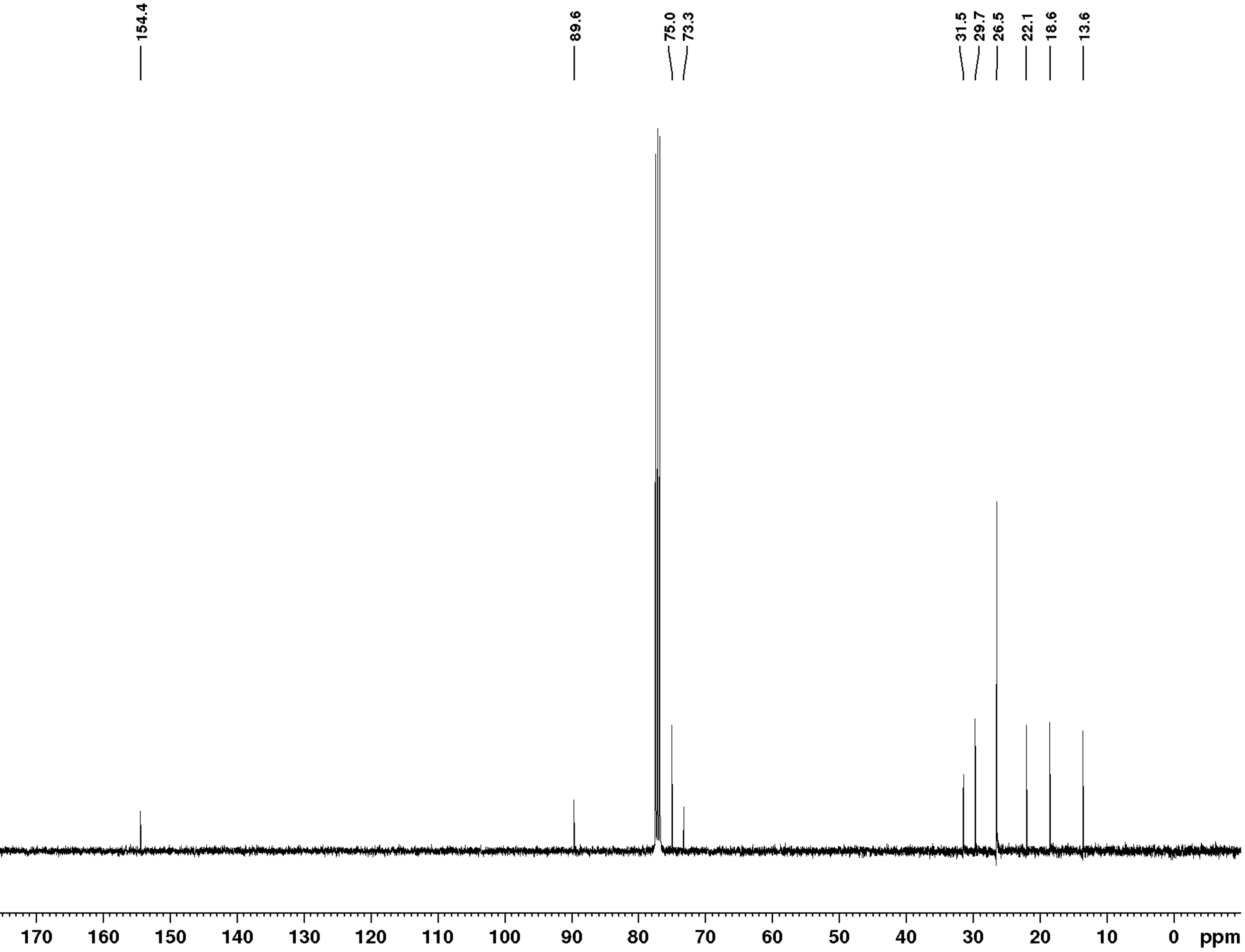

**Figure S85.** $^1$H NMR spectrum (400 MHz, $CDCl_3$, 298 K) of **2g**.

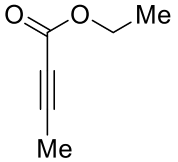


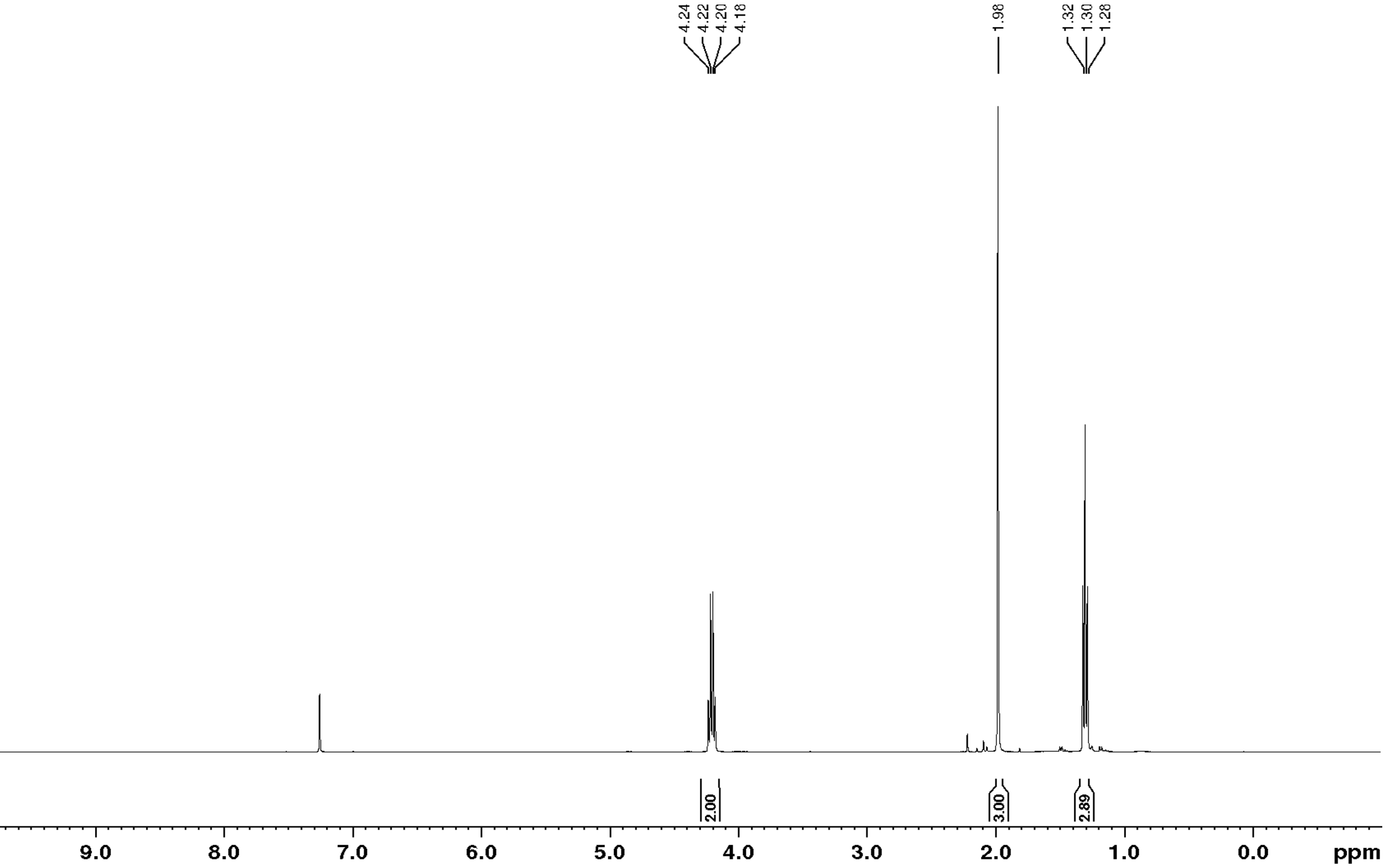

**Figure S86.** $^{13}C\{^1H\}$ NMR spectrum (100 MHz, $CDCl_3$, 298 K) of **2g**.

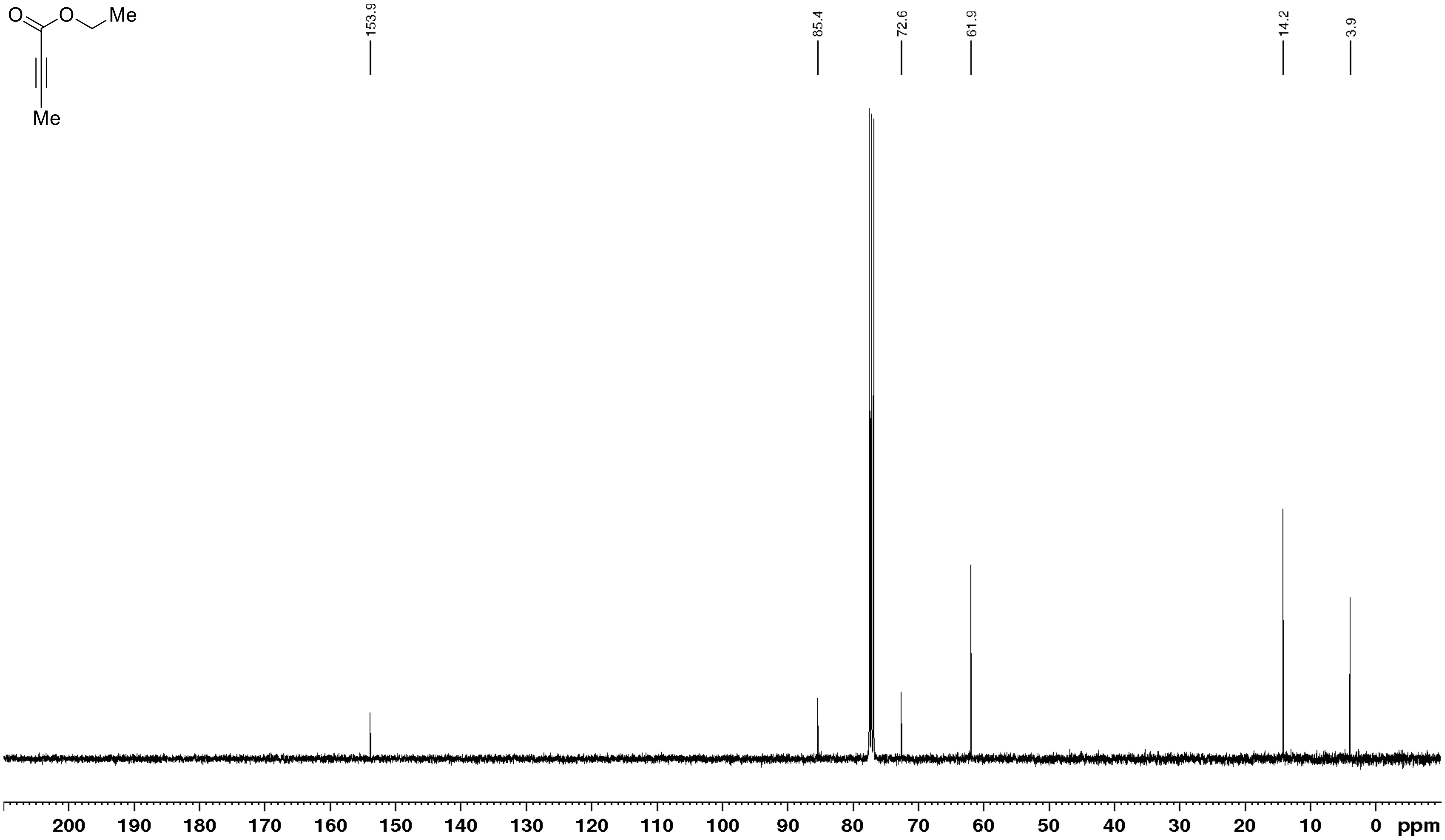

**Figure S87.** $^{1}$H NMR spectrum (400 MHz, $CDCl_3$, 298 K) of **2h**.

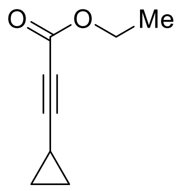


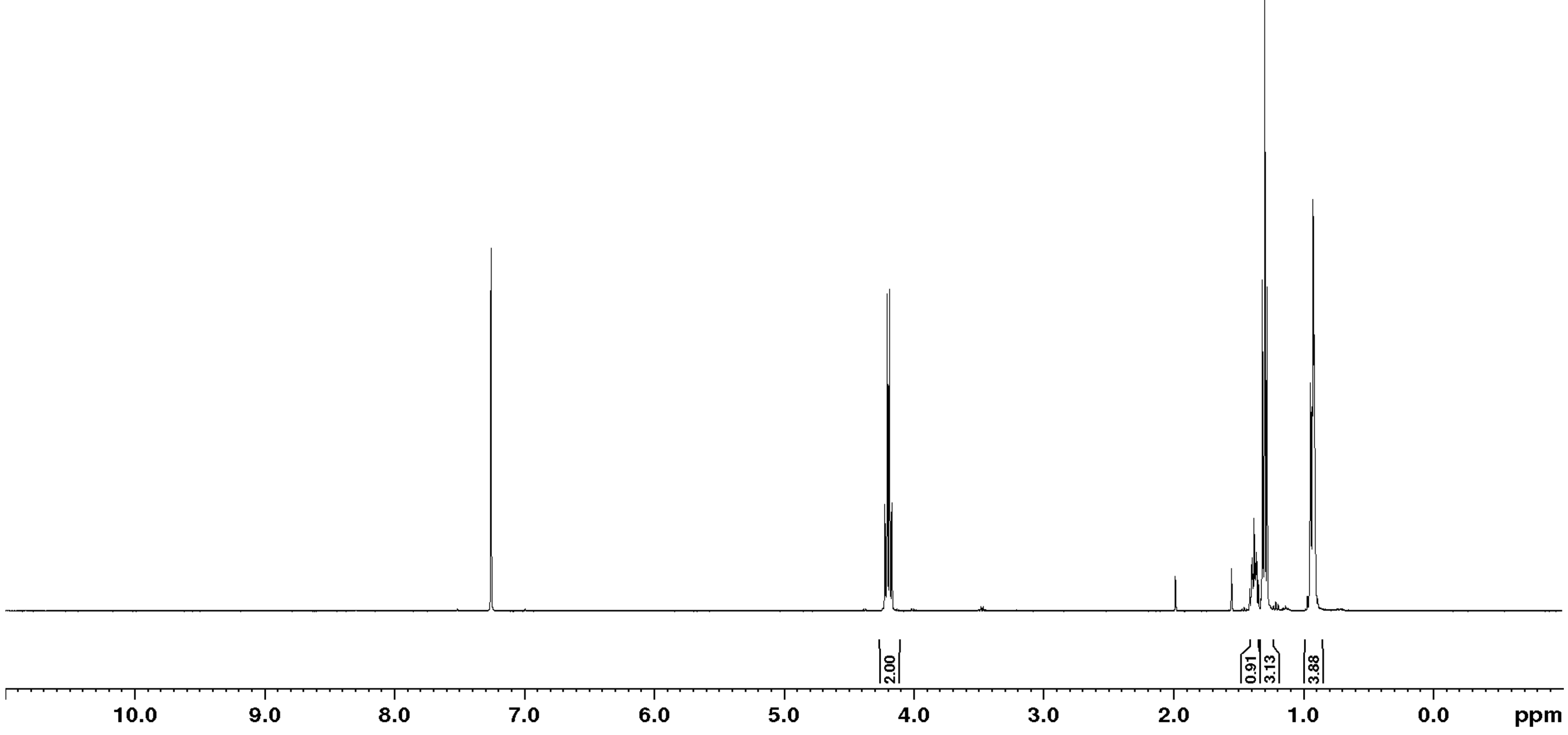

**Figure S88.** $^{13}C\{1H\}$ NMR spectrum (100 MHz, $CDCl_3$, 298 K) of **2h**.

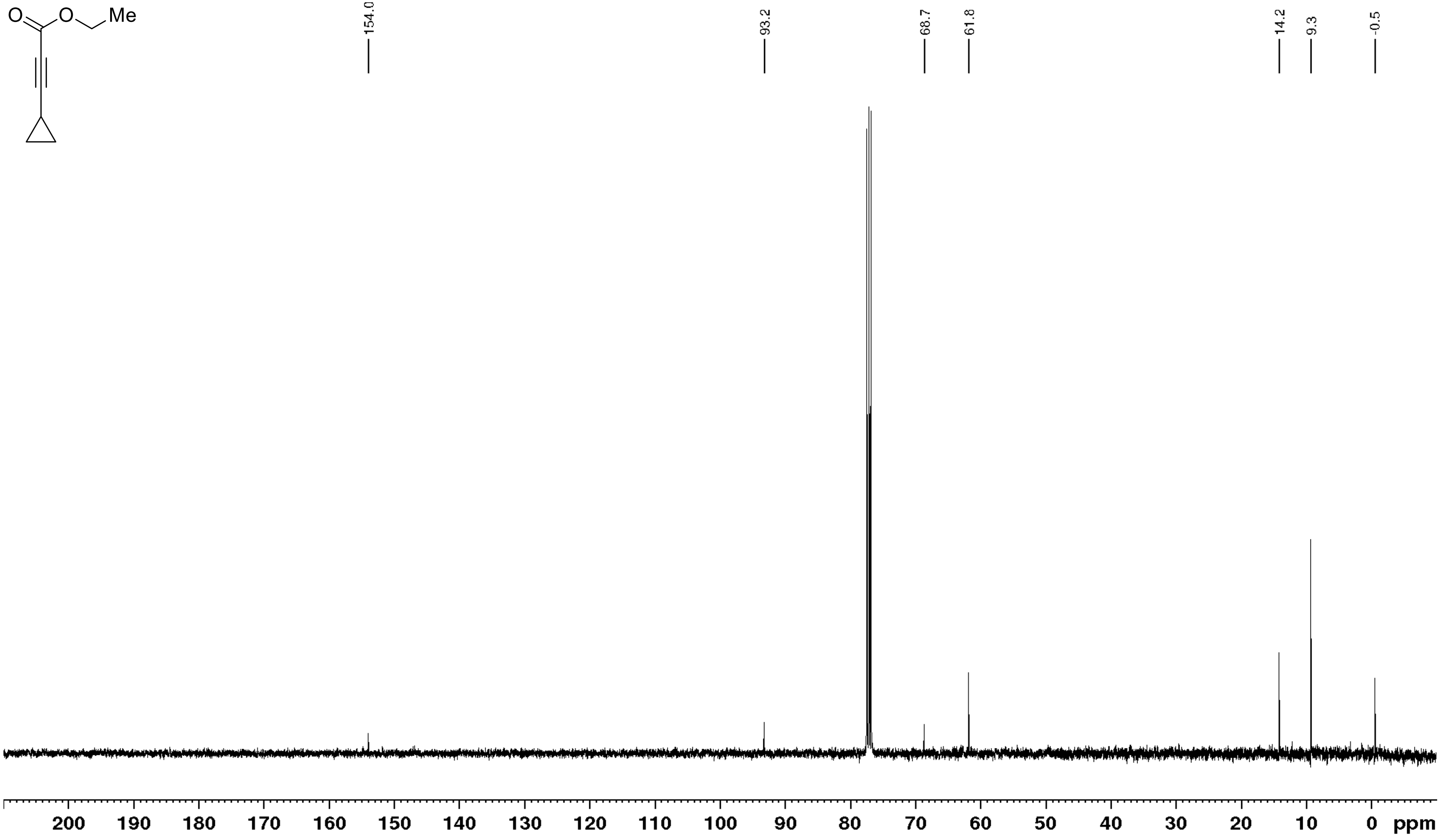

**Figure S89.** $^{1}$H NMR spectrum (400 MHz, $CDCl_3$, 298 K) of **2i**.

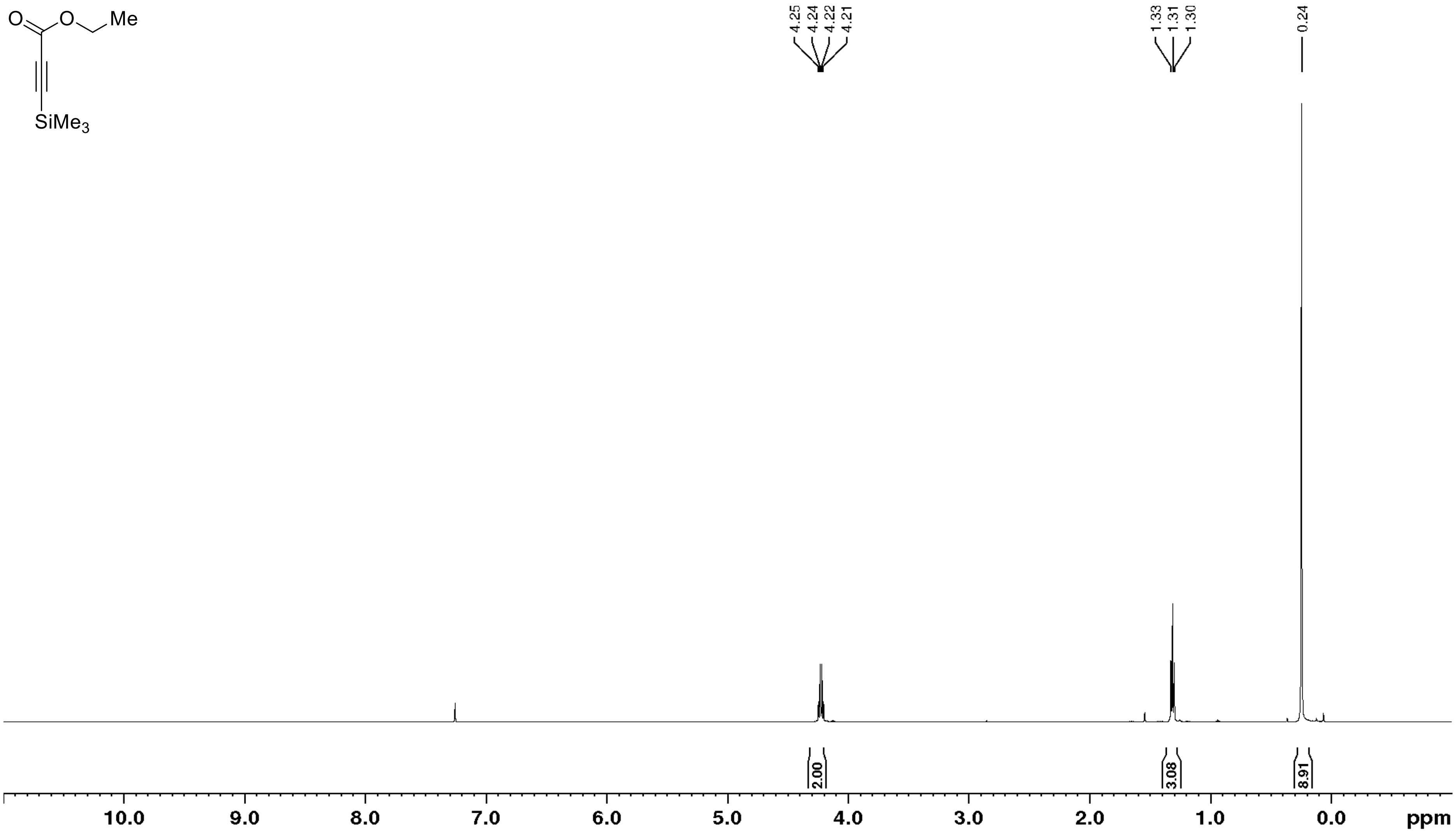

**Figure S90.** $^{13}C\{1H\}$ NMR spectrum (100 MHz, $CDCl_3$, 298 K) of **2i**.

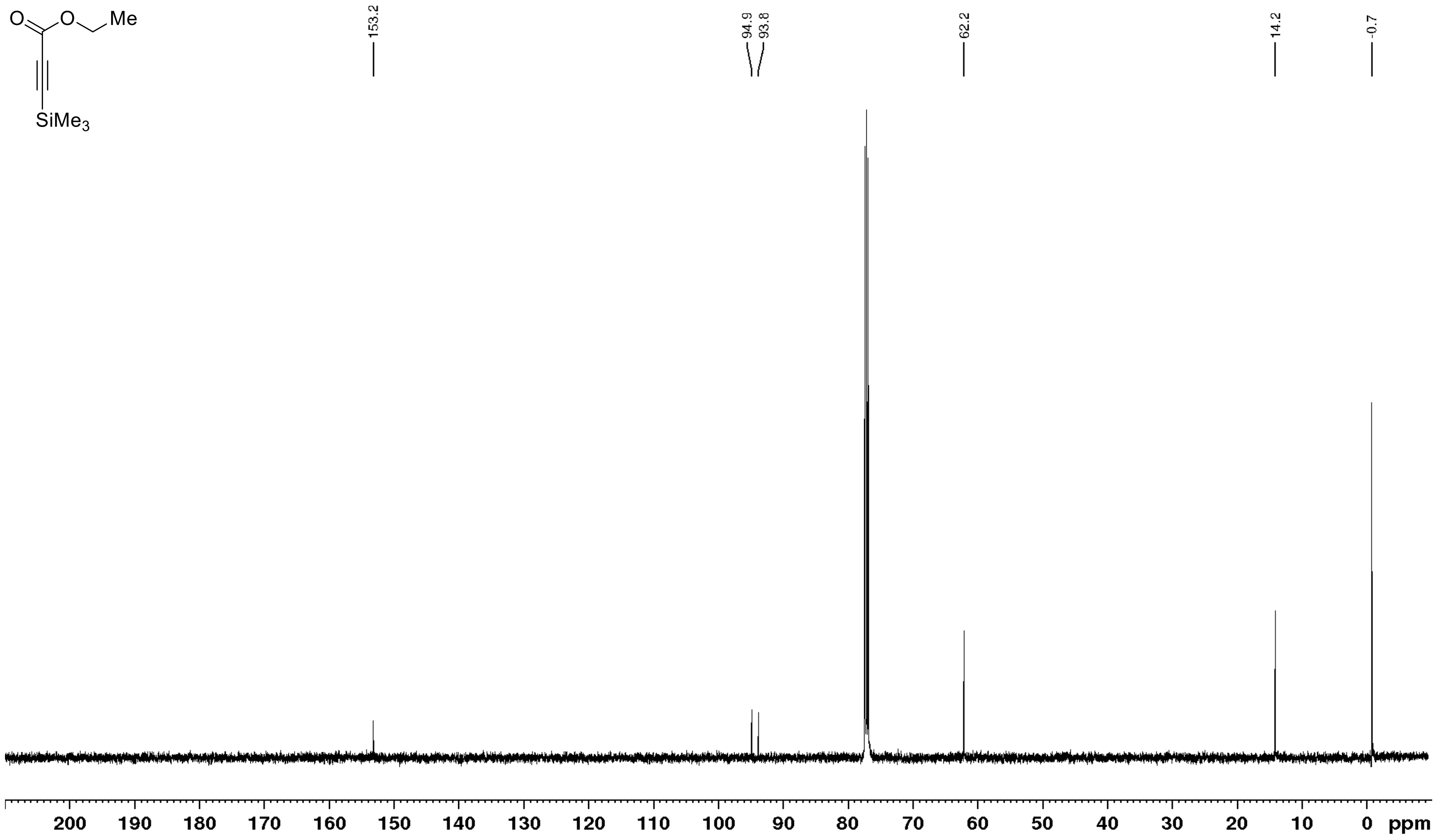

**Figure S91.** $^{1}$H NMR spectrum (400 MHz, $CDCl_3$, 298 K) of **2j**.

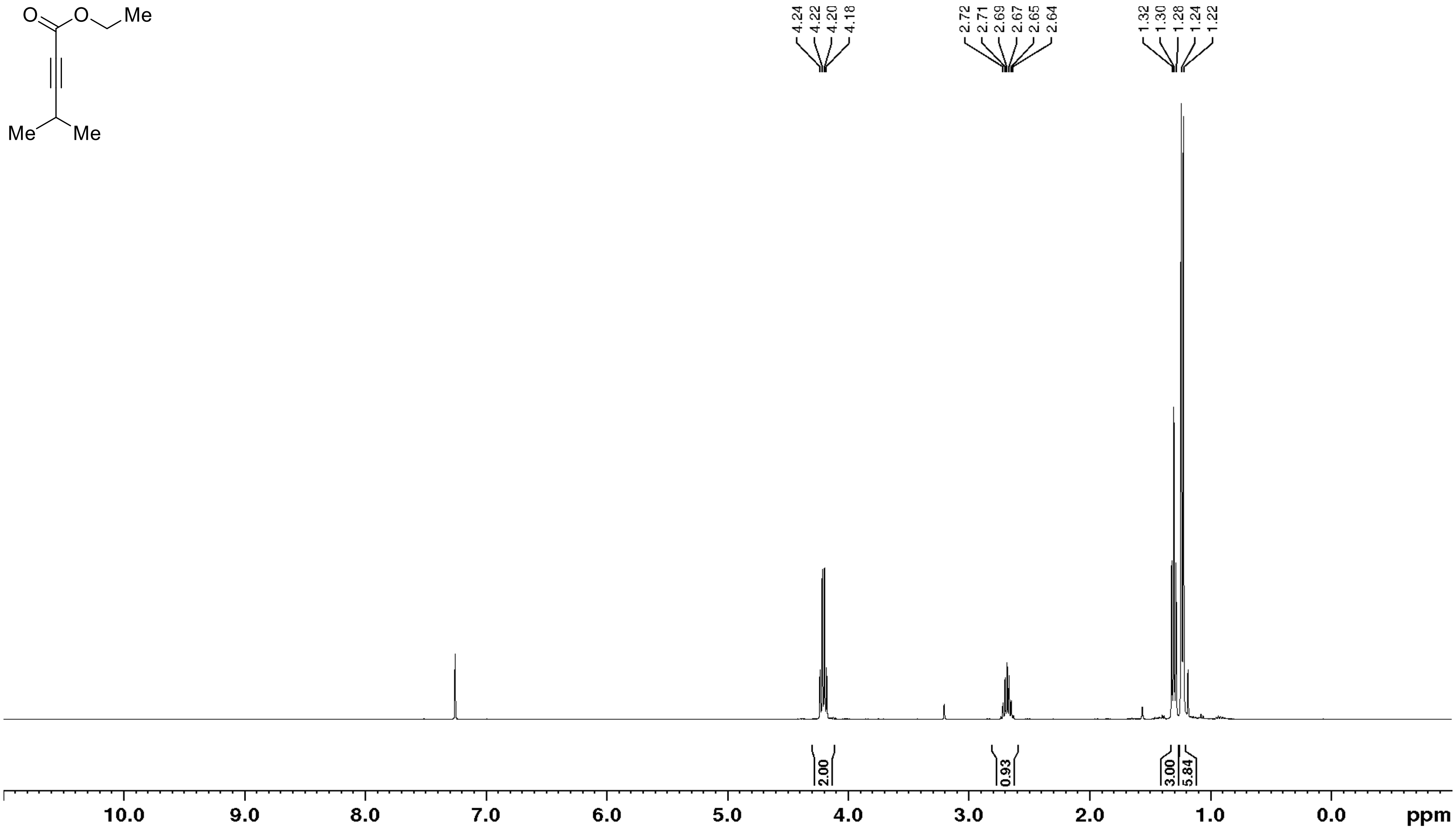

**Figure S92.** $^{13}C\{^1H\}$ NMR spectrum (100 MHz, $CDCl_3$, 298 K) of **2j**.

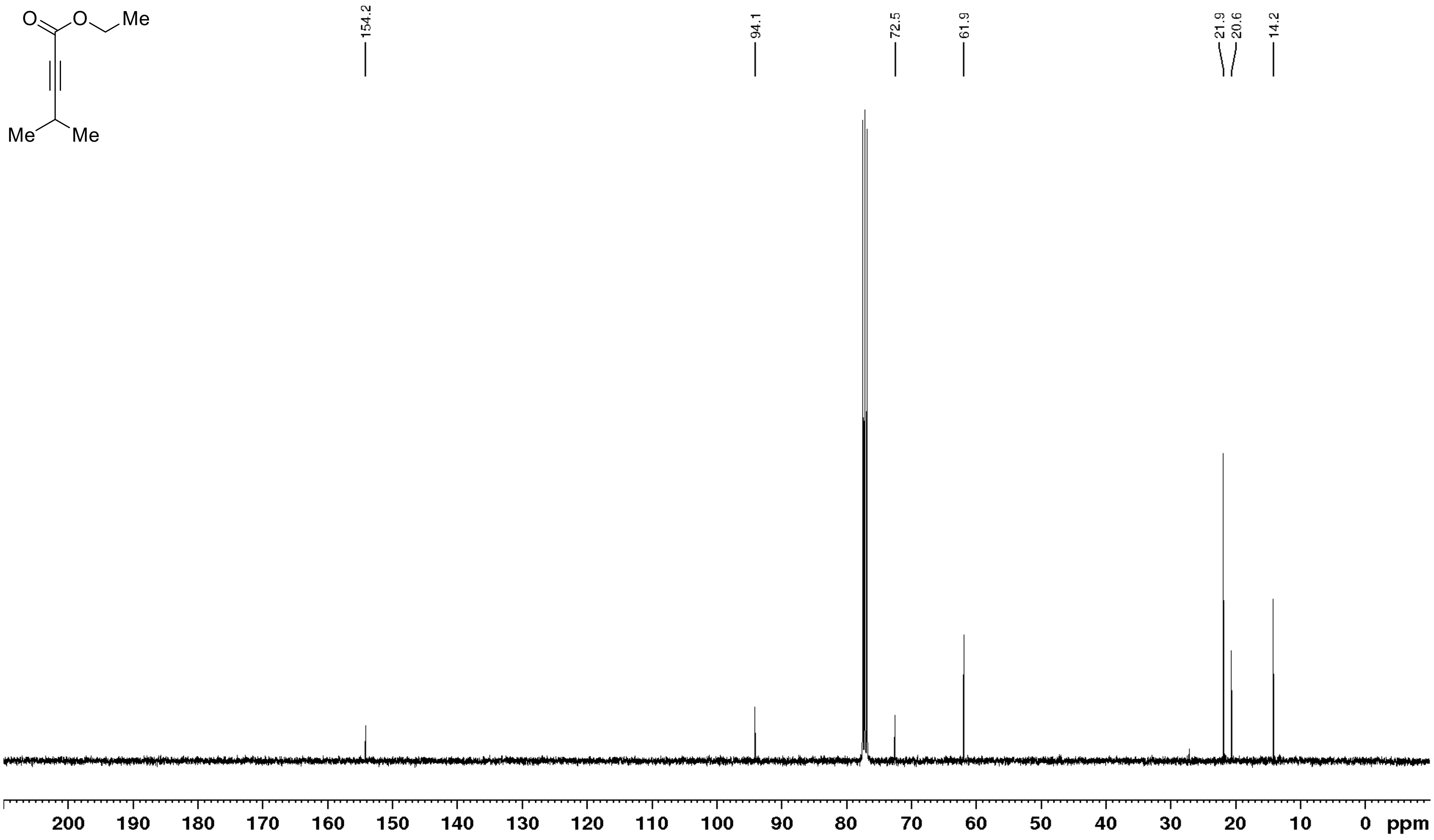

**Figure S93.** $^{1}$H NMR spectrum (400 MHz, $CDCl_3$, 298 K) of **2k**.

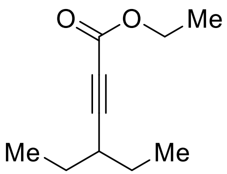


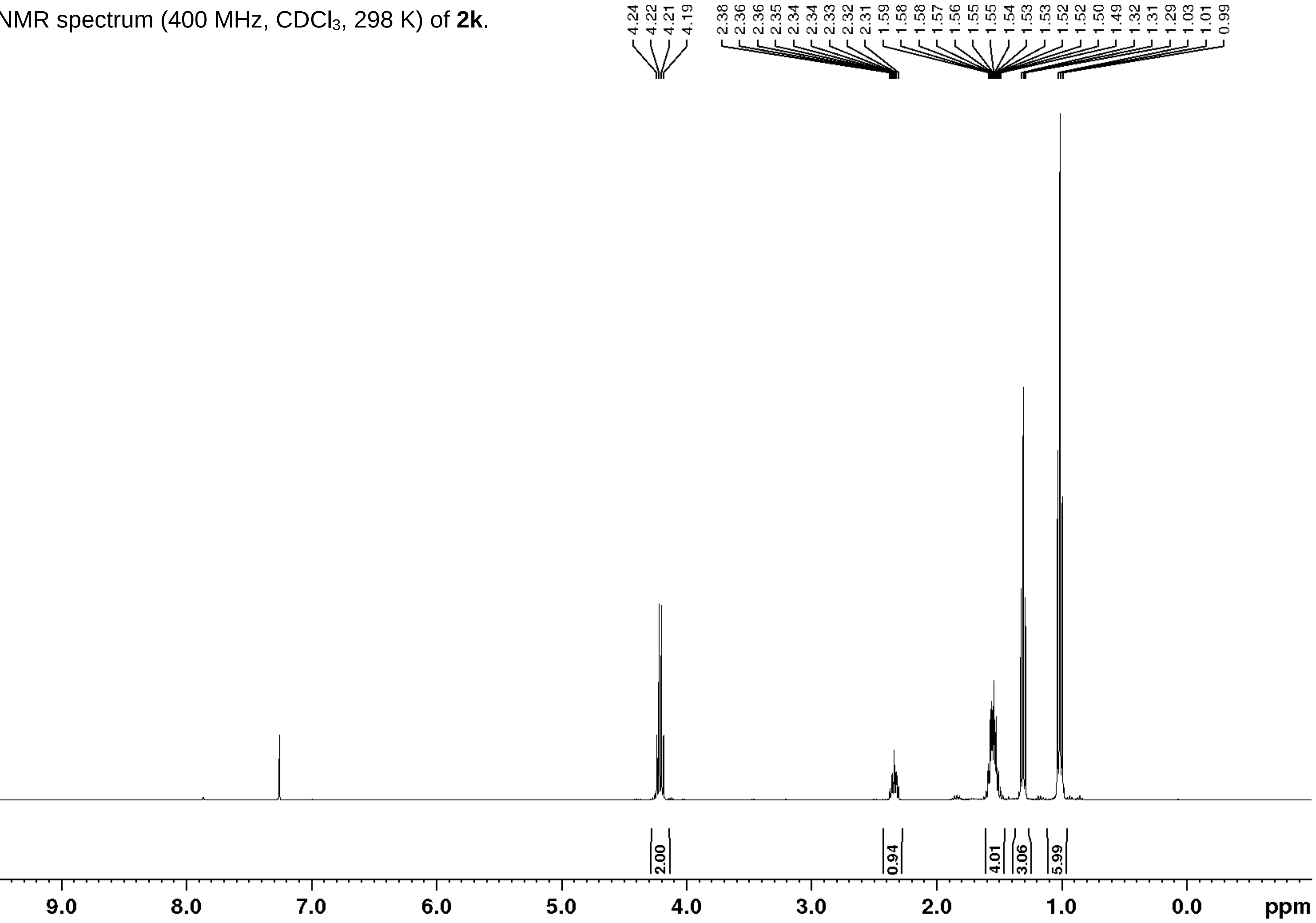

**Figure S94.** $^{13}C\{1H\}$ NMR spectrum (100 MHz, $CDCl_3$, 298 K) of **2k**.

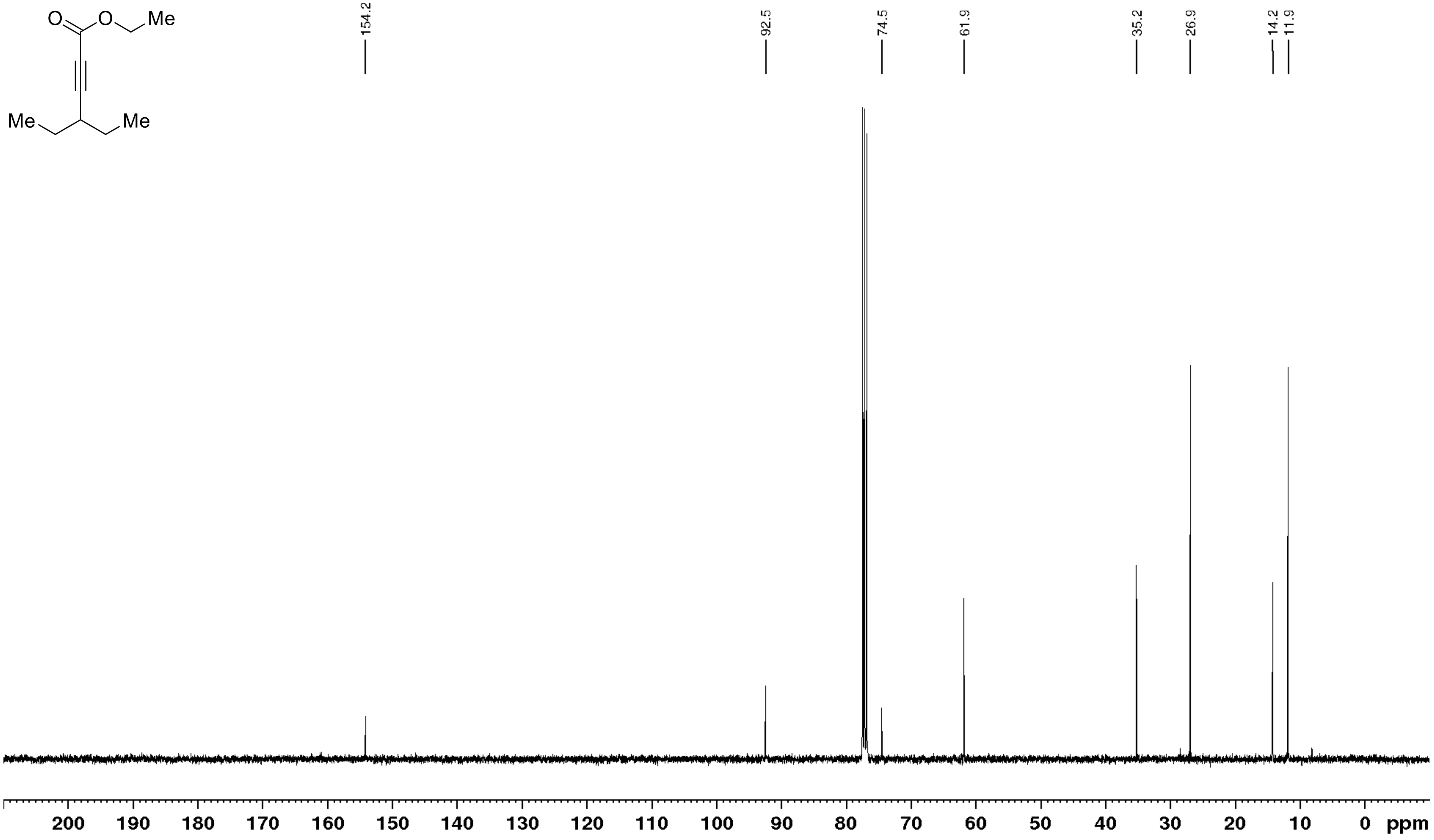

**Figure S95.** $^{1}$H NMR spectrum (400 MHz, $CDCl_3$, 298 K) of.

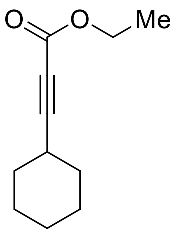


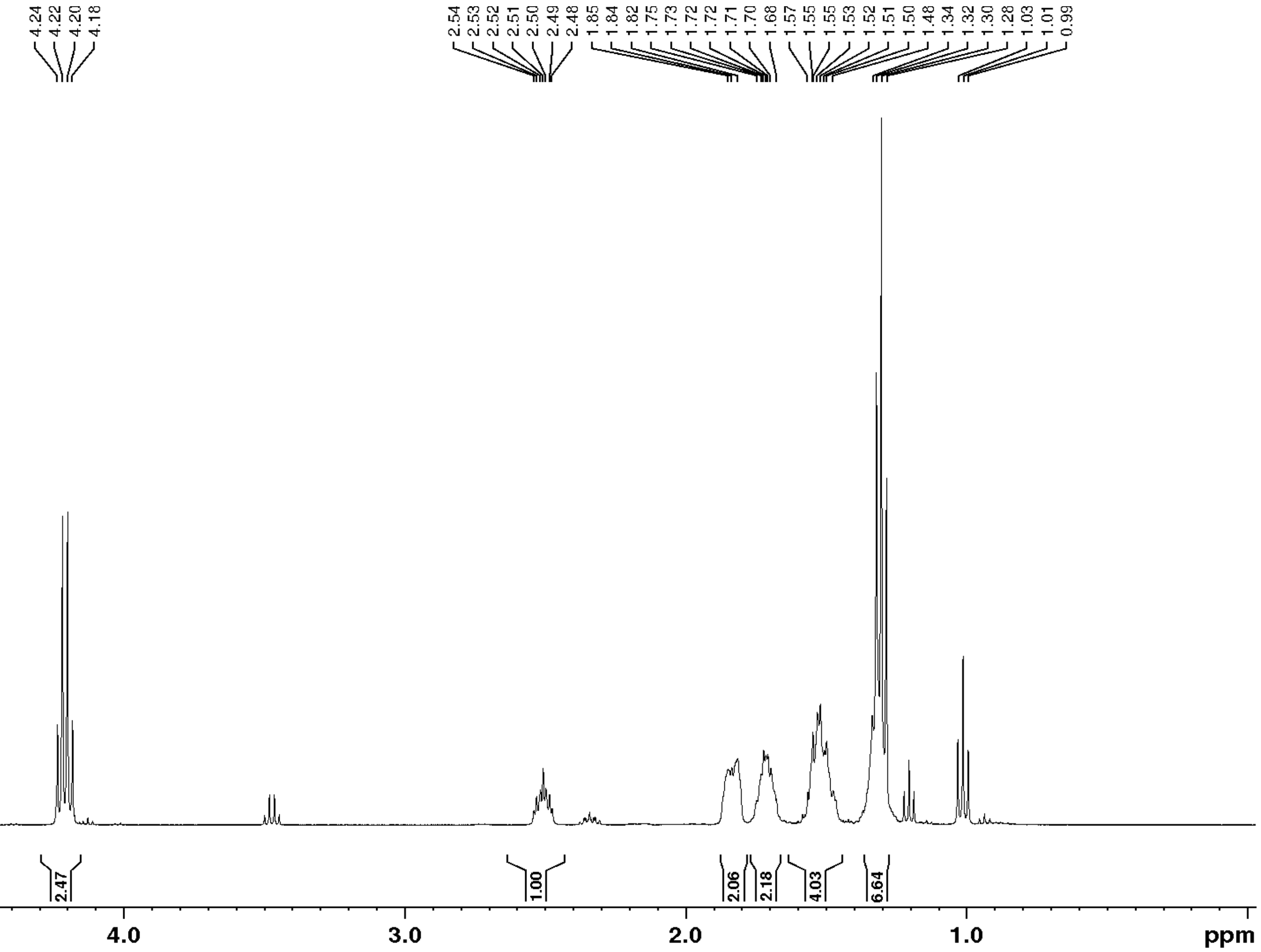

**Figure S96.** $^{13}C\{1H\}$ NMR spectrum (100 MHz, $CDCl_3$, 298 K) of **2l**.

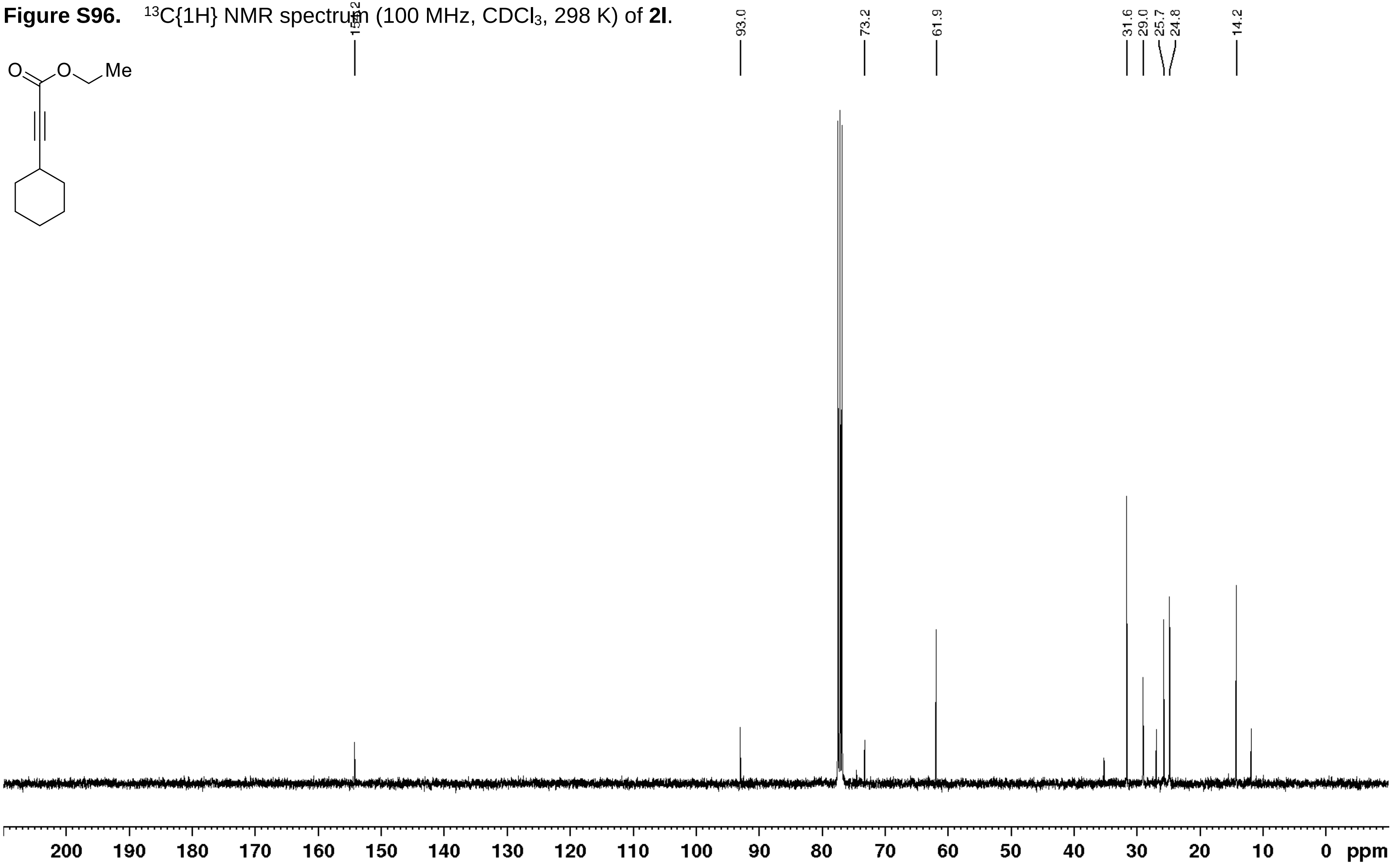

**Figure S97.** $^{1}$H NMR spectrum (400 MHz, $CDCl_3$, 298 K) of **2m**.

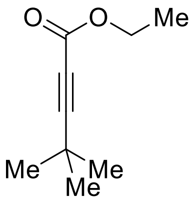


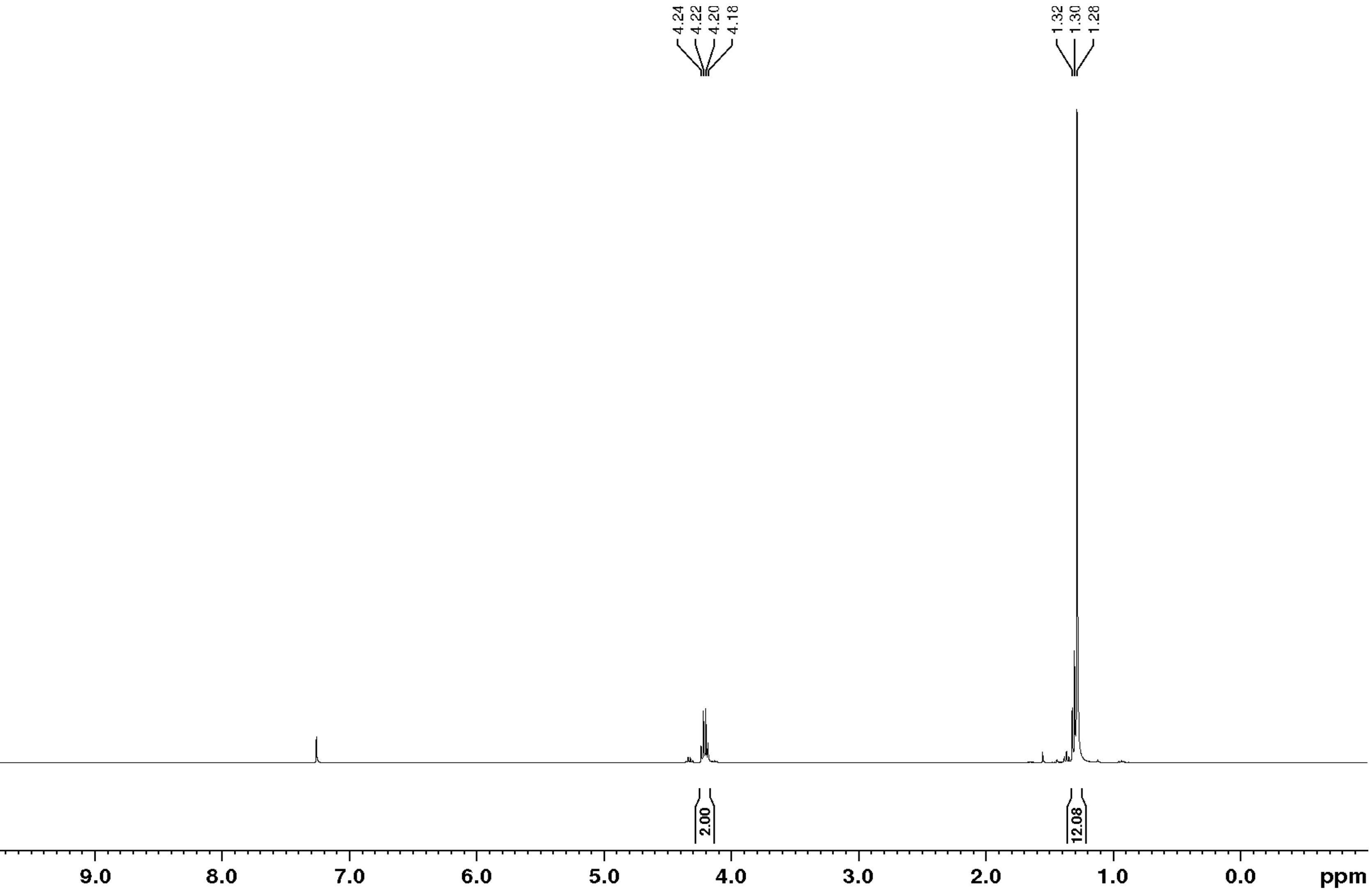

**Figure S98.** $^{13}C\{1H\}$ NMR spectrum (100 MHz, $CDCl_3$, 298 K) of **2m**.

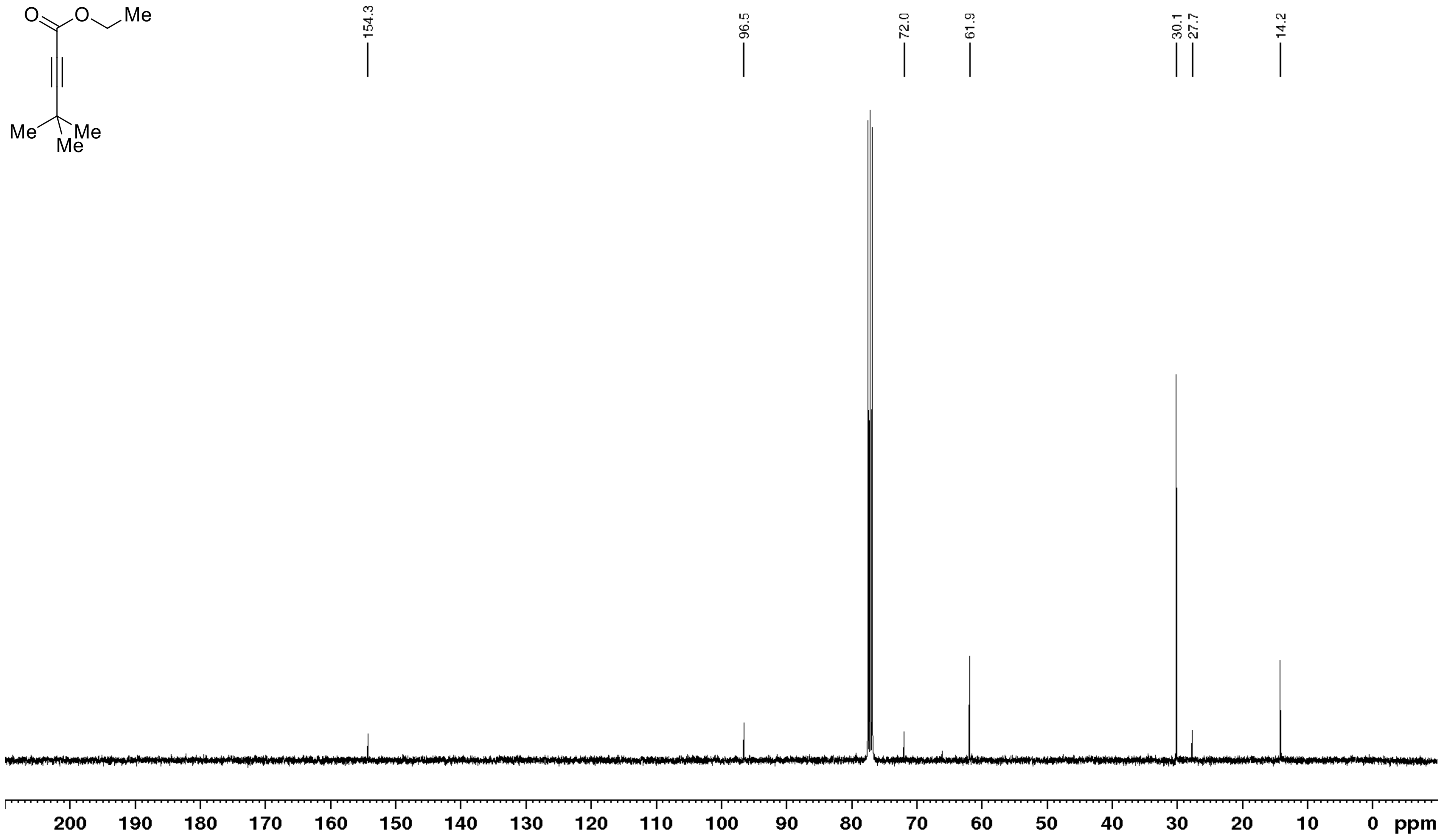

**Figure S99.** $^{1}$H NMR spectrum (400 MHz, $CDCl_3$, 298 K) of **3aa**.

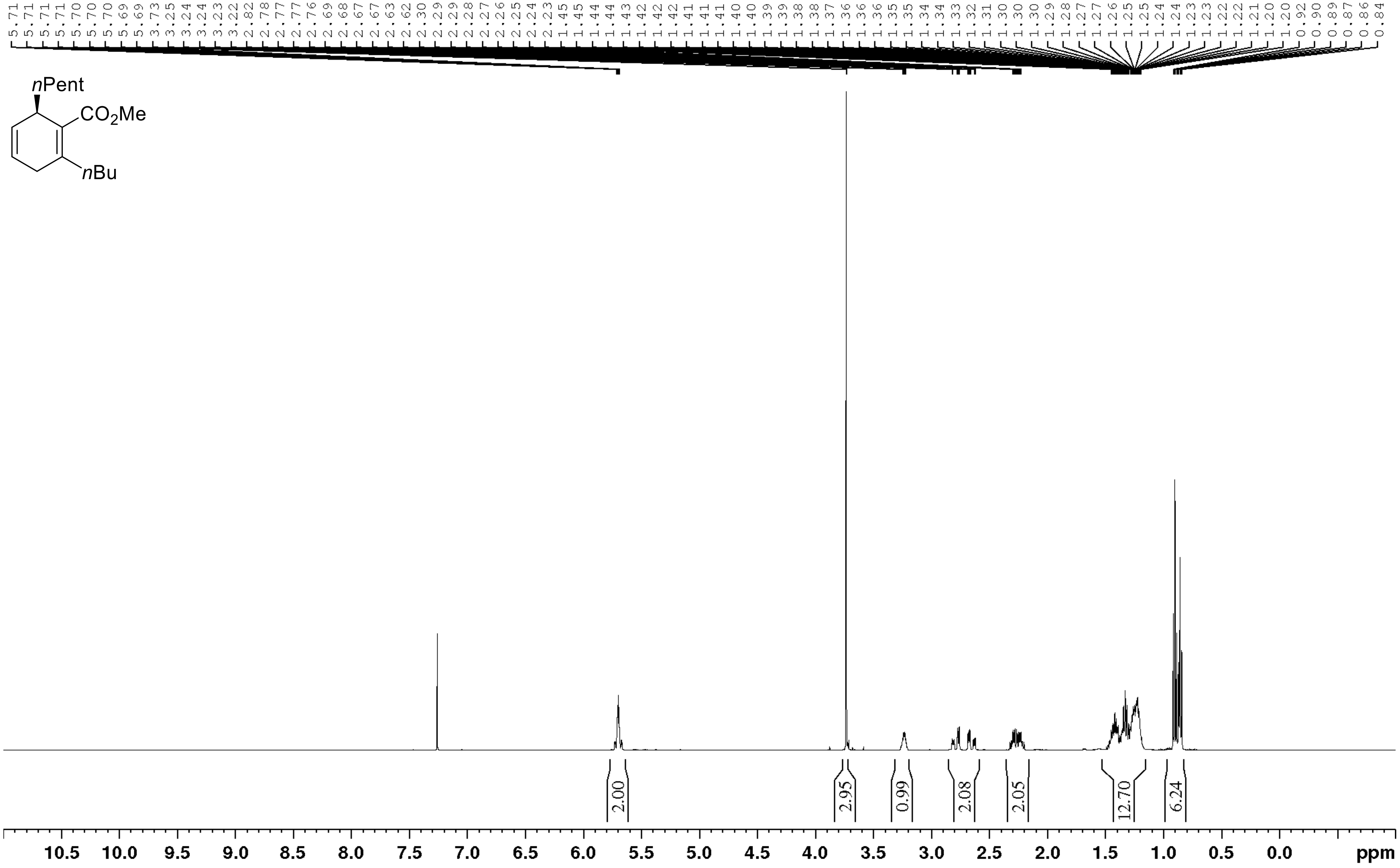

**Figure S100.** $^{13}C\{^1H\}$ NMR spectrum (100 MHz, $CDCl_3$, 298 K) of **3aa**.

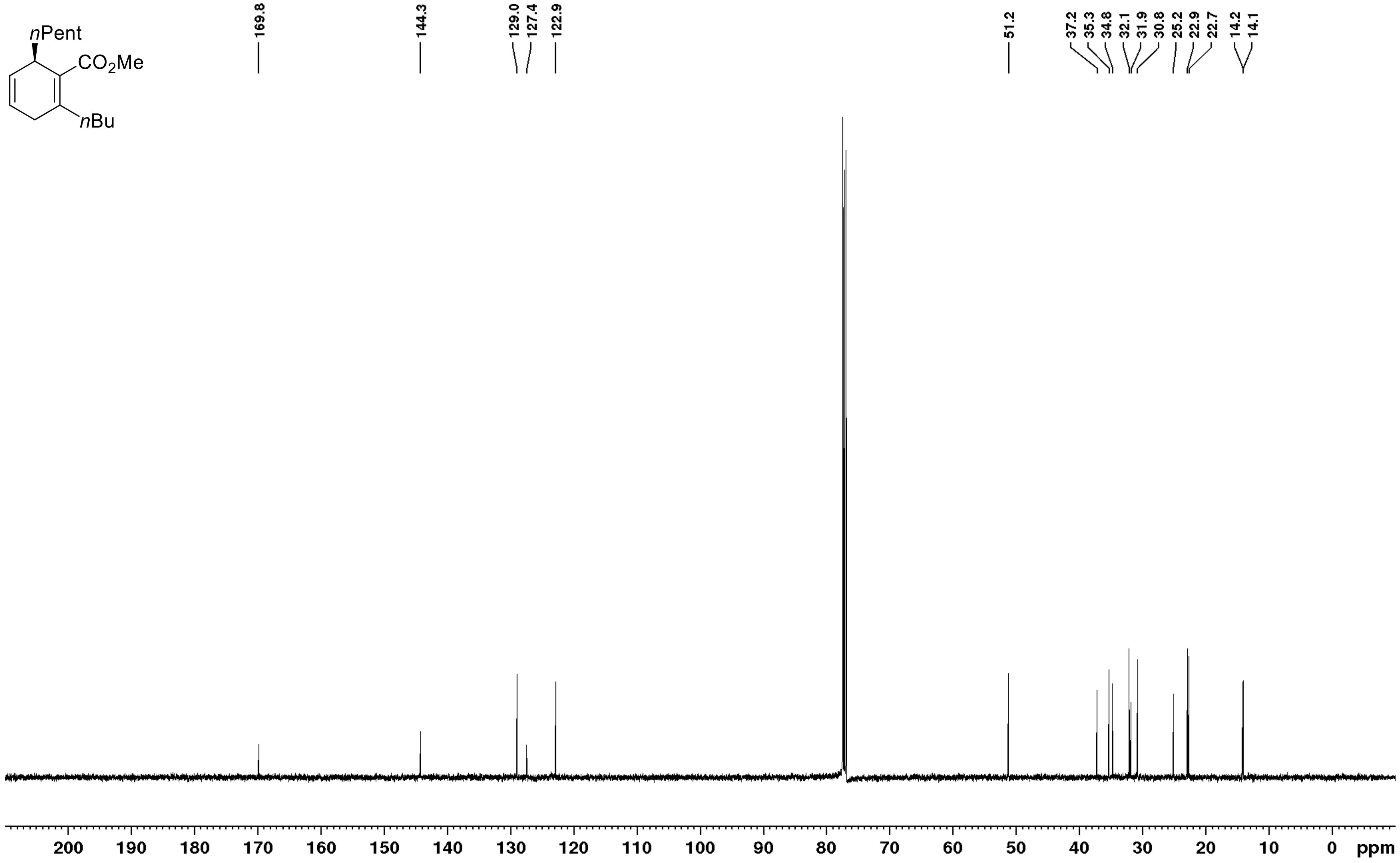

**Figure S101.** $^1$H NMR spectrum (400 MHz, $CDCl_3$, 298 K) of **3ab**.

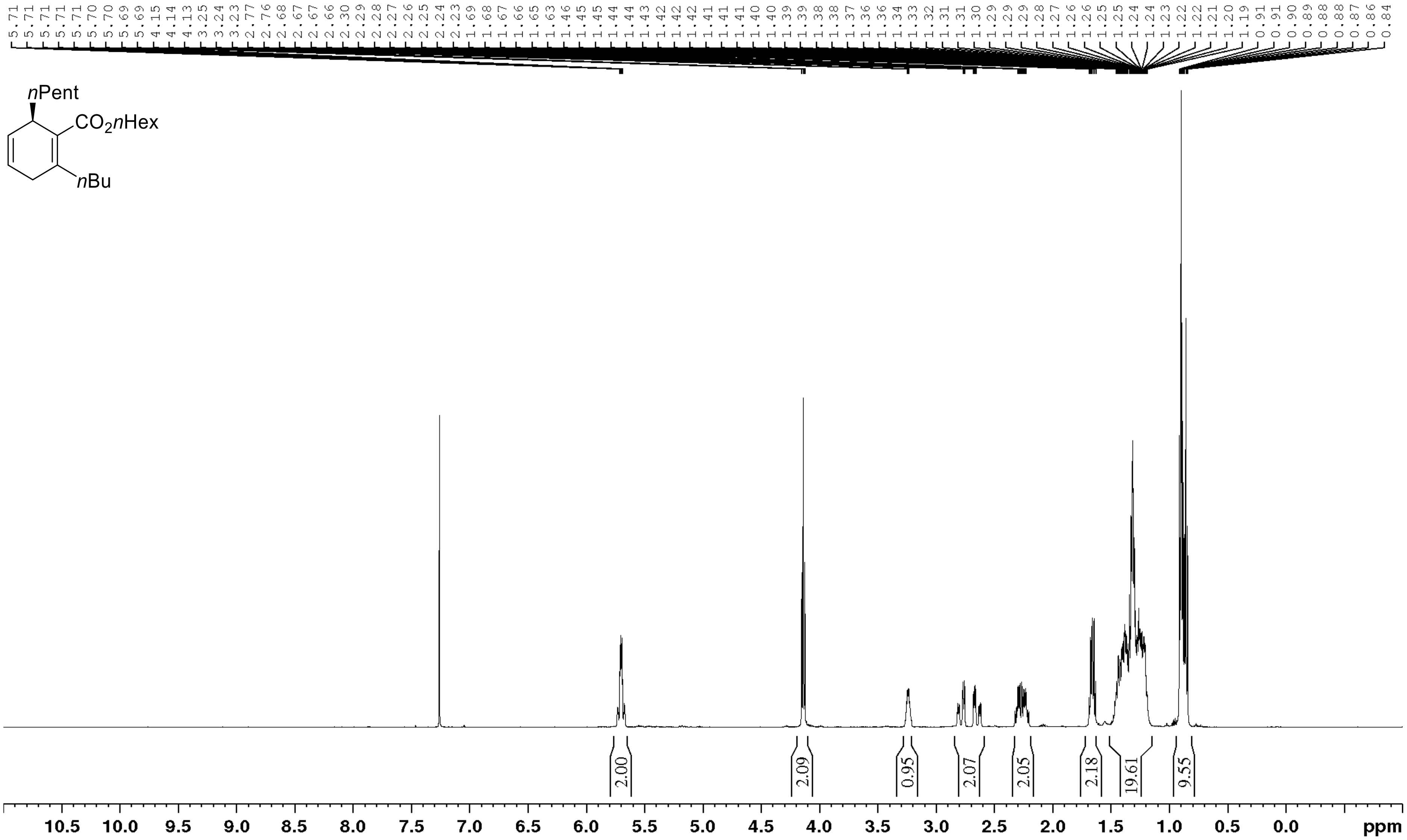

**Figure S102.** $^{13}C\{^1H\}$ NMR spectrum (100 MHz, $CDCl_3$, 298 K) of **3ab**.

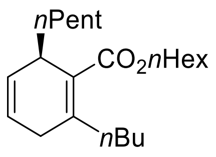


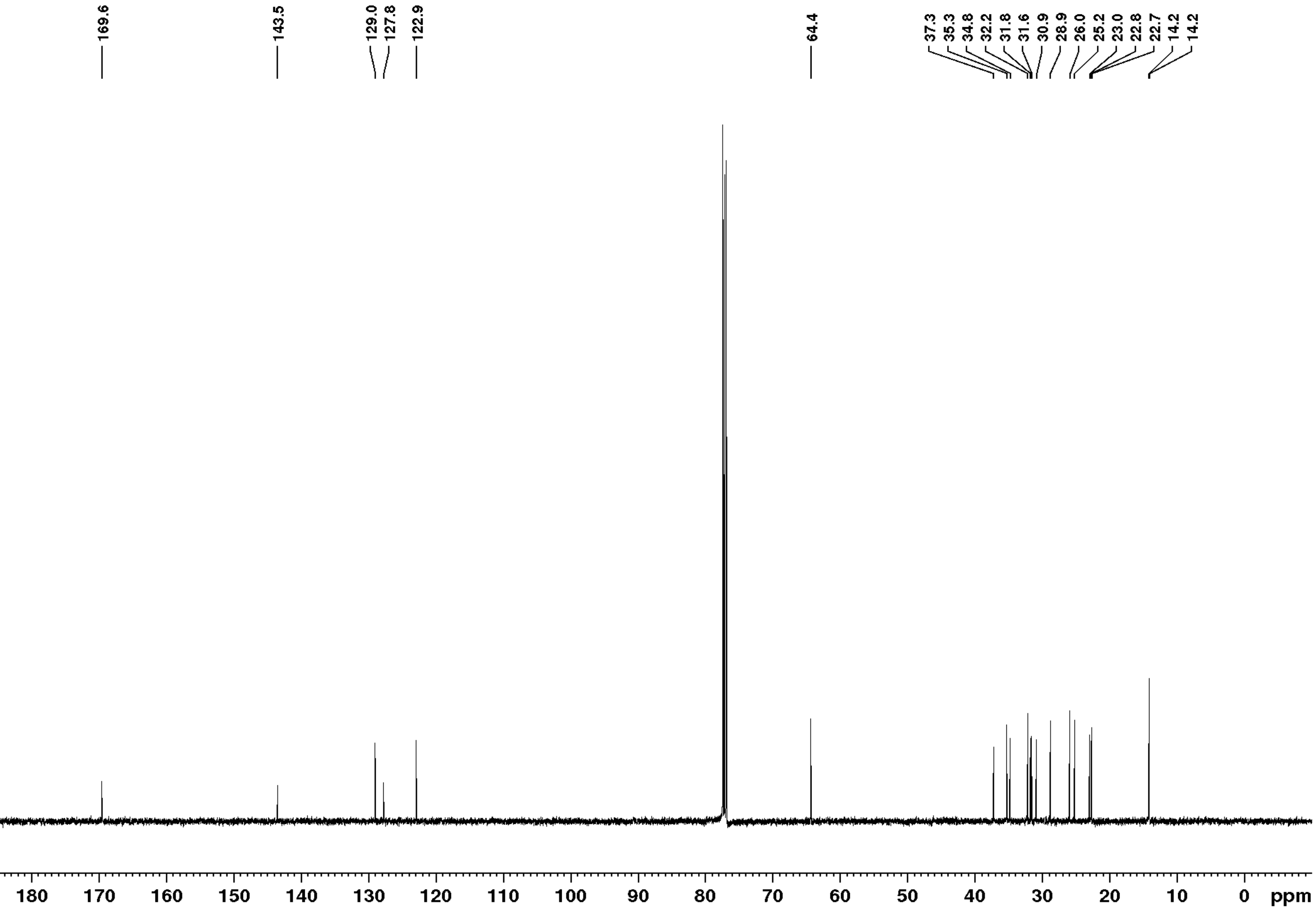

**Figure S103.** $^1H$ NMR spectrum (400 MHz, $CDCl_3$, 298 K) of **3ac**.

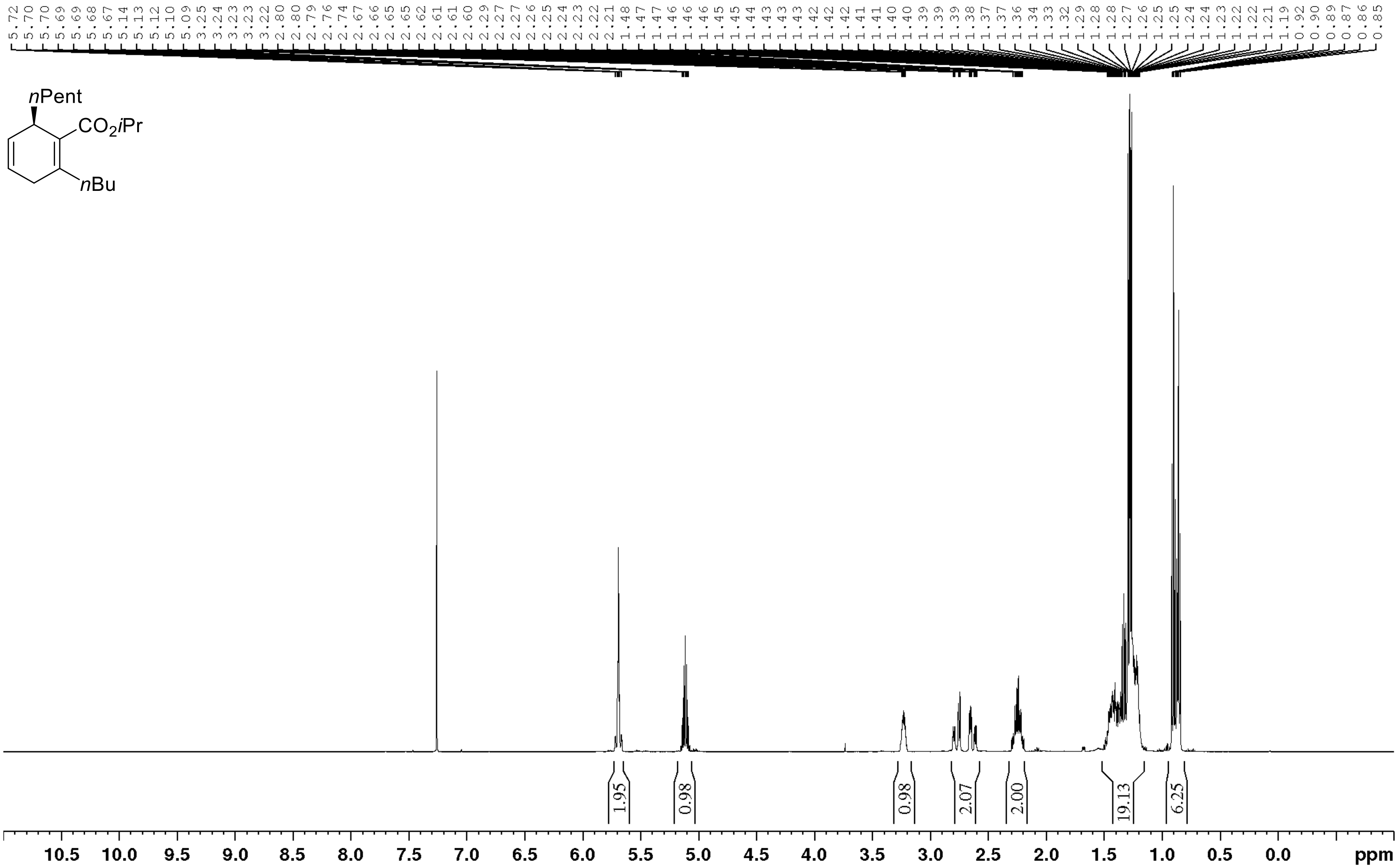

**Figure S104.** $^{13}C\{1H\}$ NMR spectrum (100 MHz, $CDCl_3$, 298 K) of **3ac**.

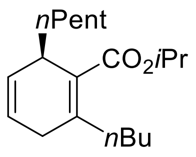


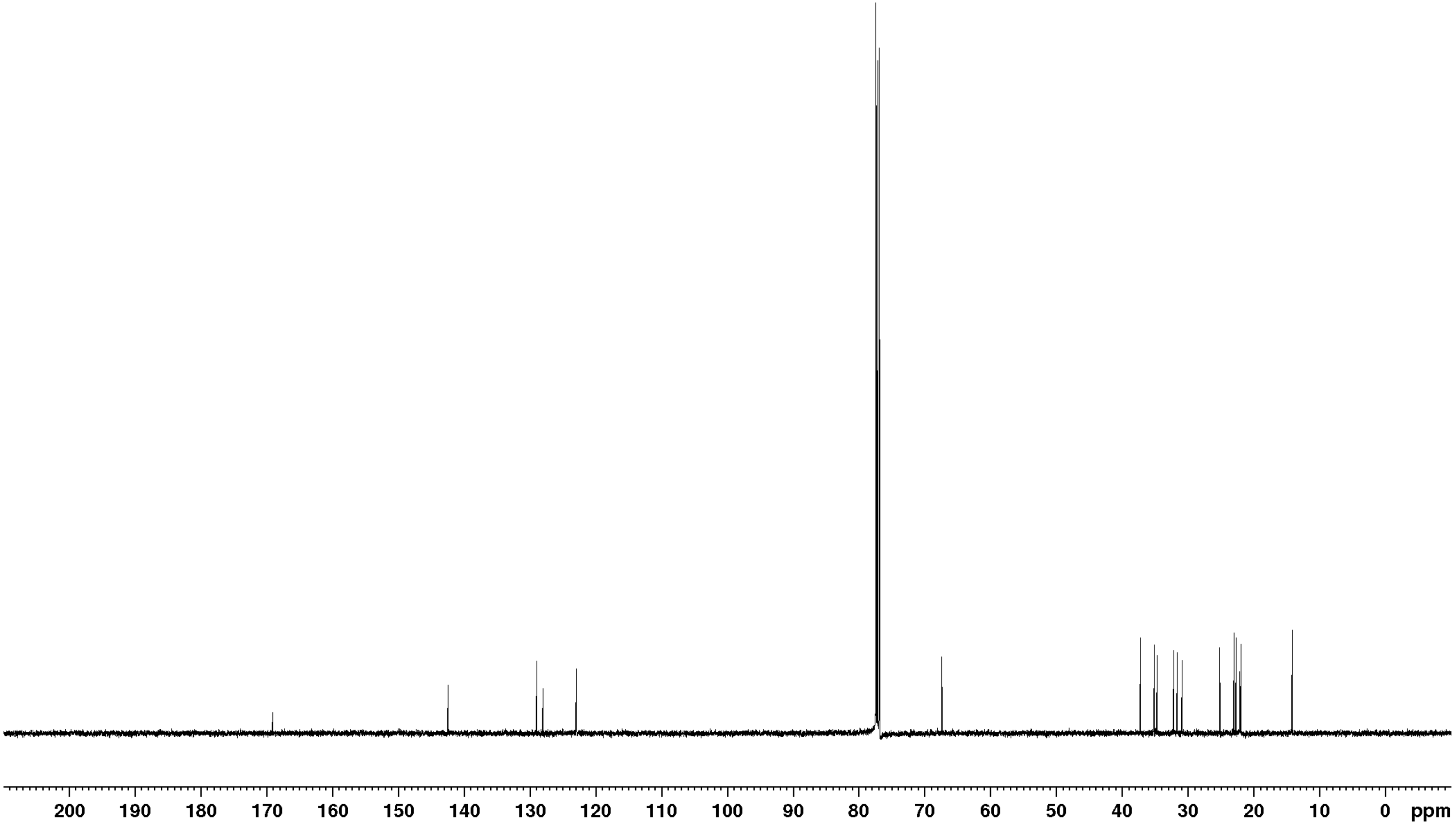

**Figure S105.** $^1H$ NMR spectrum (400 MHz, $CDCl_3$, 298 K) of **3ad**.

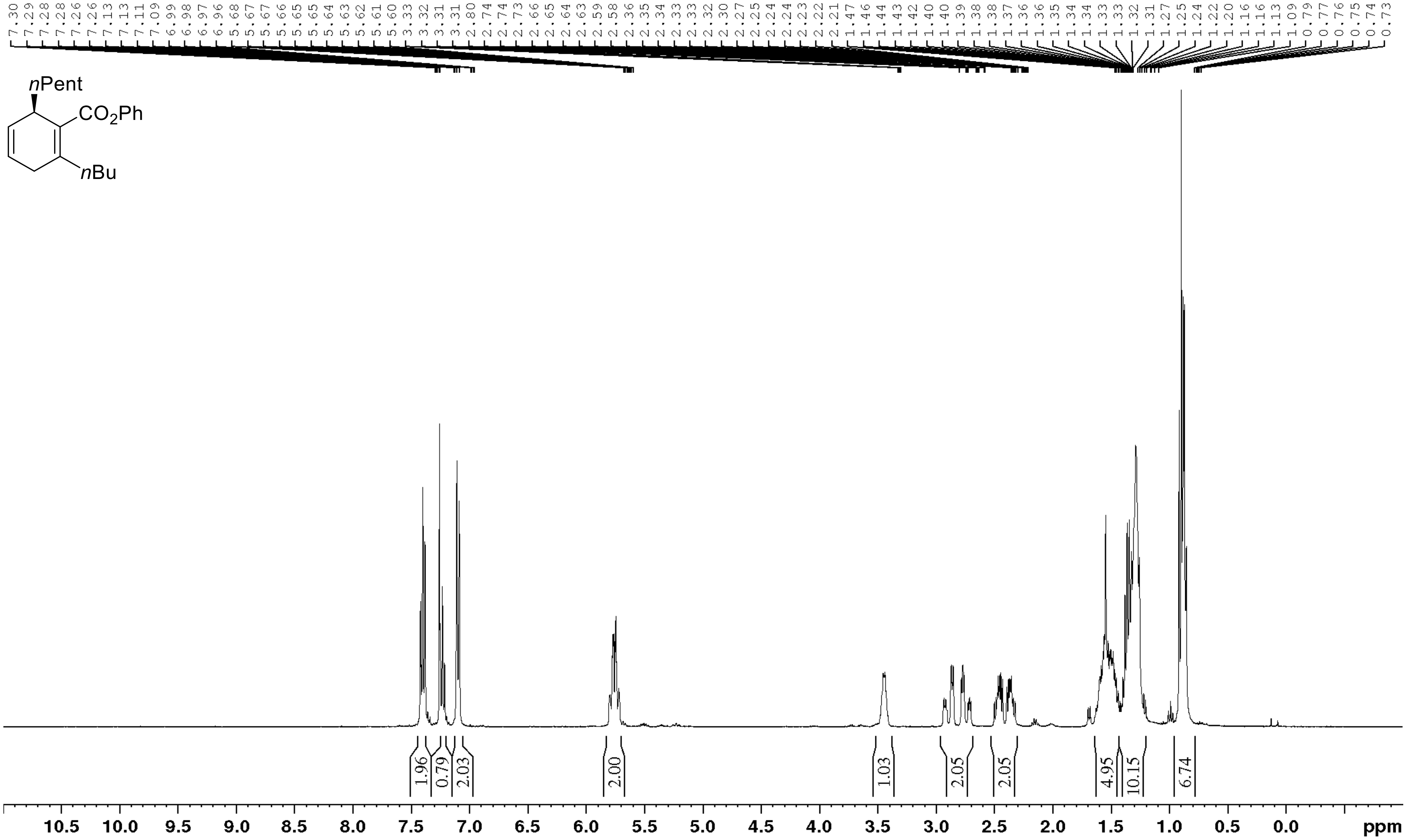

**Figure S106.** $^{13}C\{1H\}$ NMR spectrum (100 MHz, $CDCl_3$, 298 K) of **3ad**.

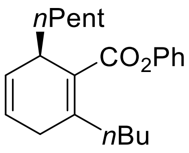


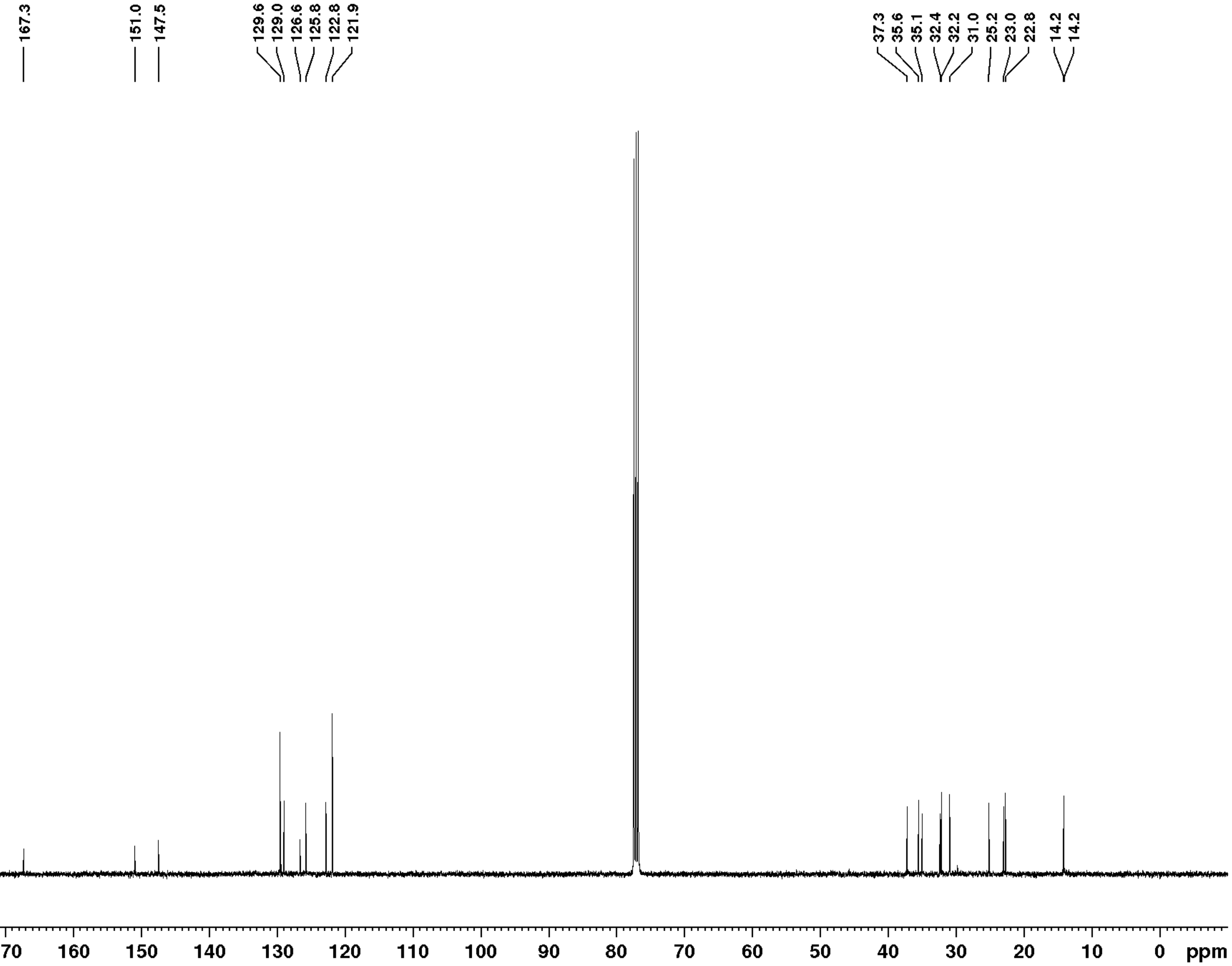

**Figure S107.** $^{1}$H NMR spectrum (400 MHz, $CDCl_3$, 298 K) of **3ae**.

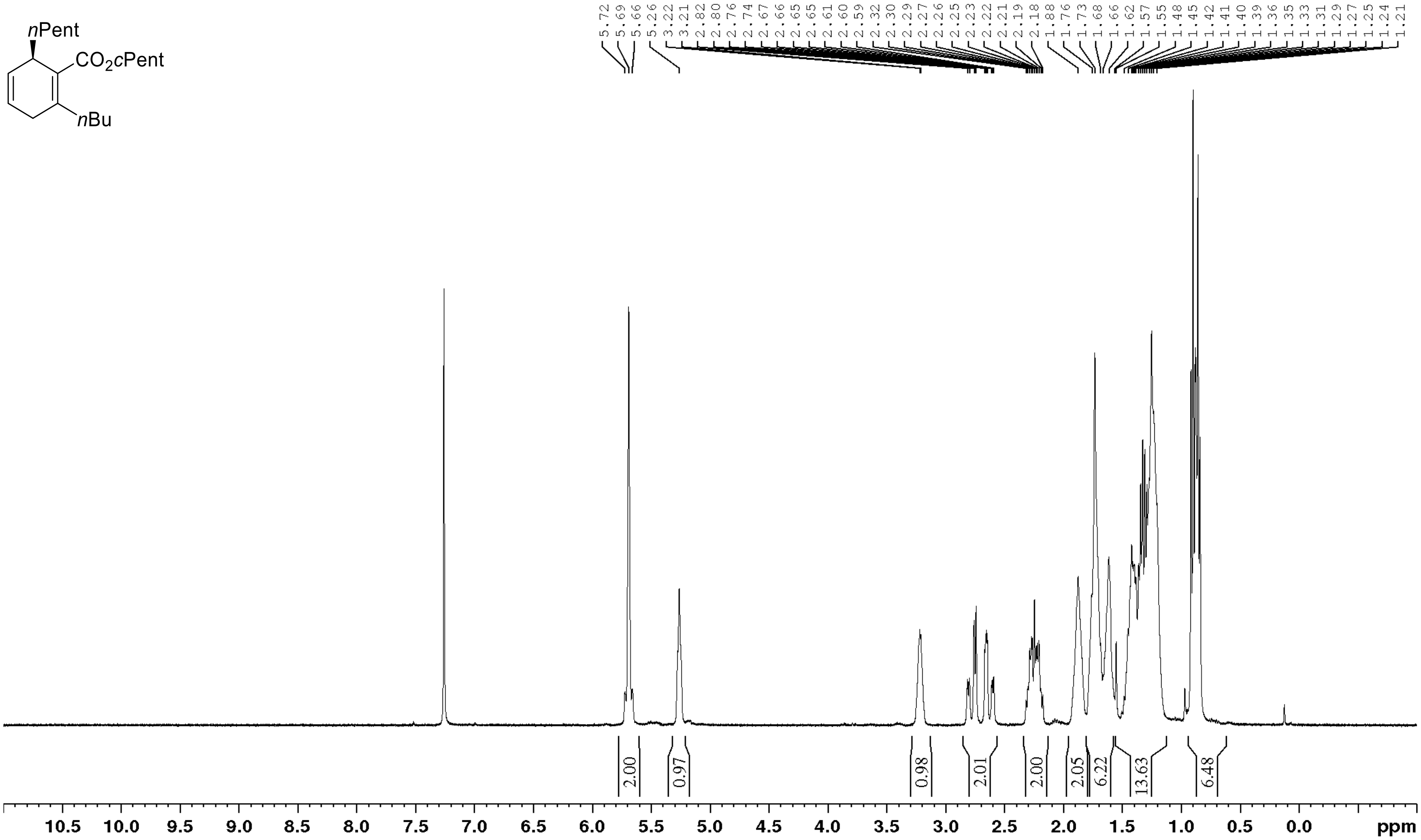

**Figure S108.** $^{13}C\{1H\}$ NMR spectrum (100 MHz, $CDCl_3$, 298 K) of **3ae**.

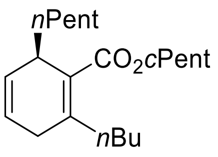


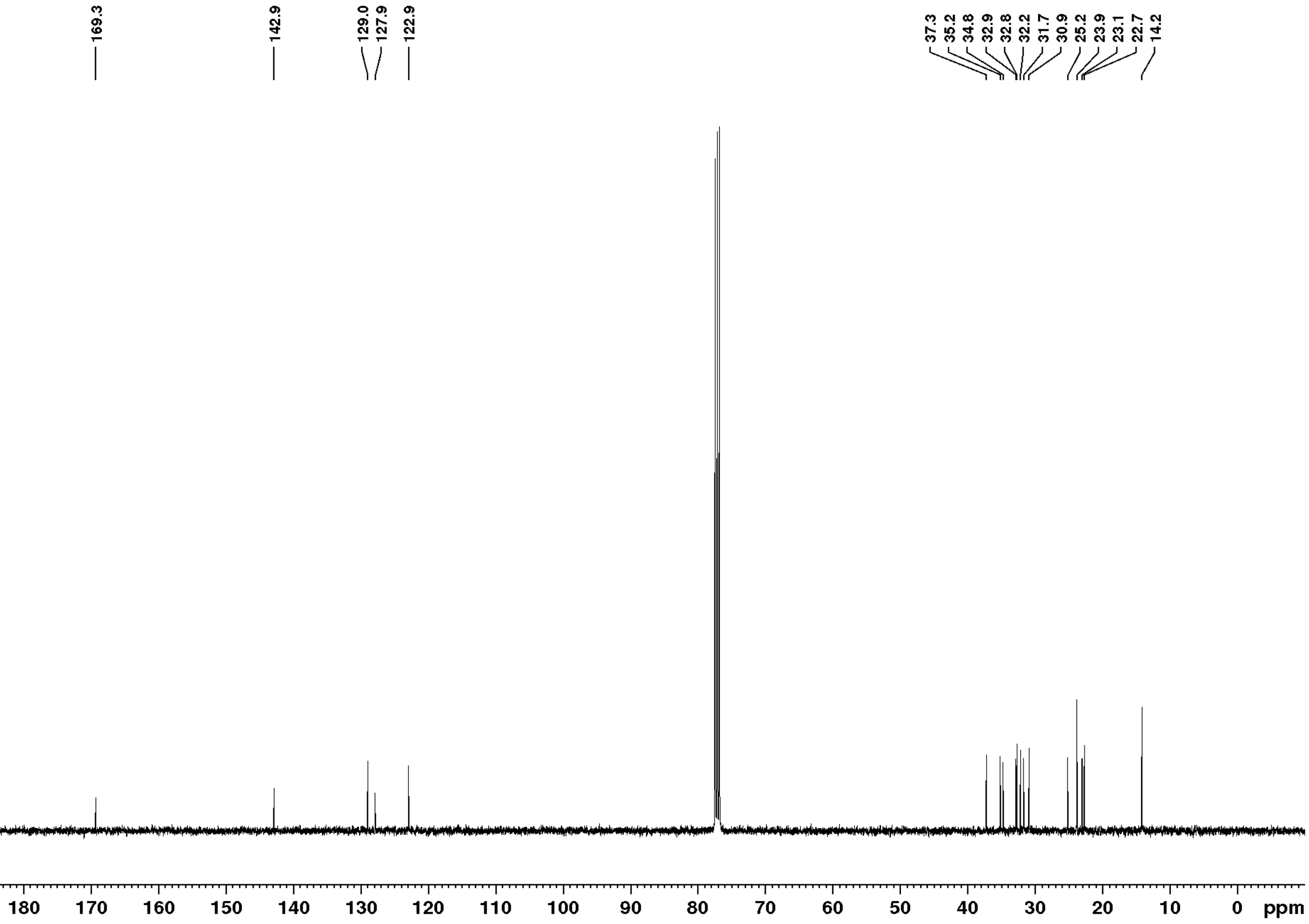

**Figure S109.** $^{1}$H NMR spectrum (400 MHz, $CDCl_3$, 298 K) of **3af**.

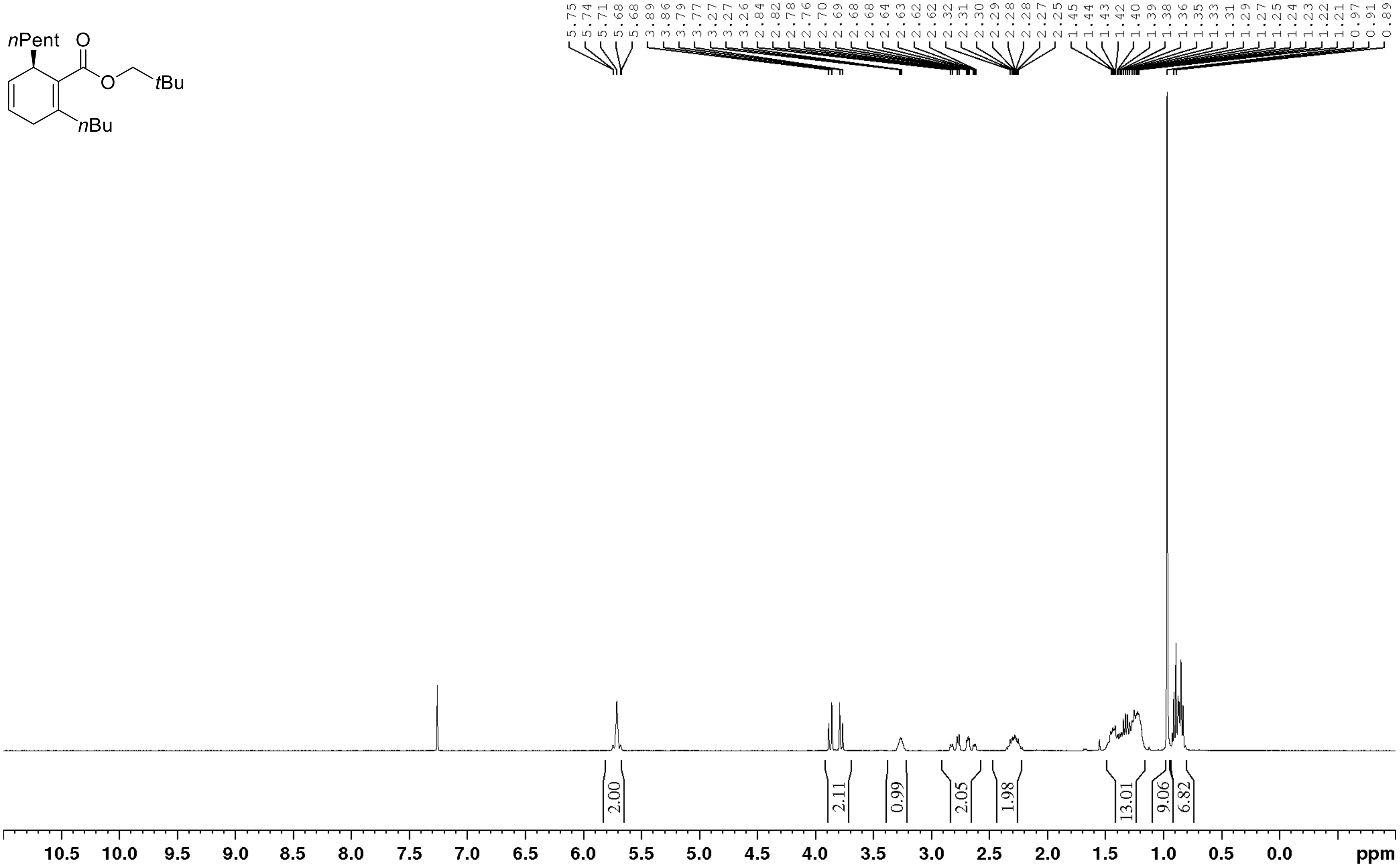

**Figure S110.** $^{13}C\{^1H\}$ NMR spectrum (100 MHz, $CDCl_3$, 298 K) of **3af**.

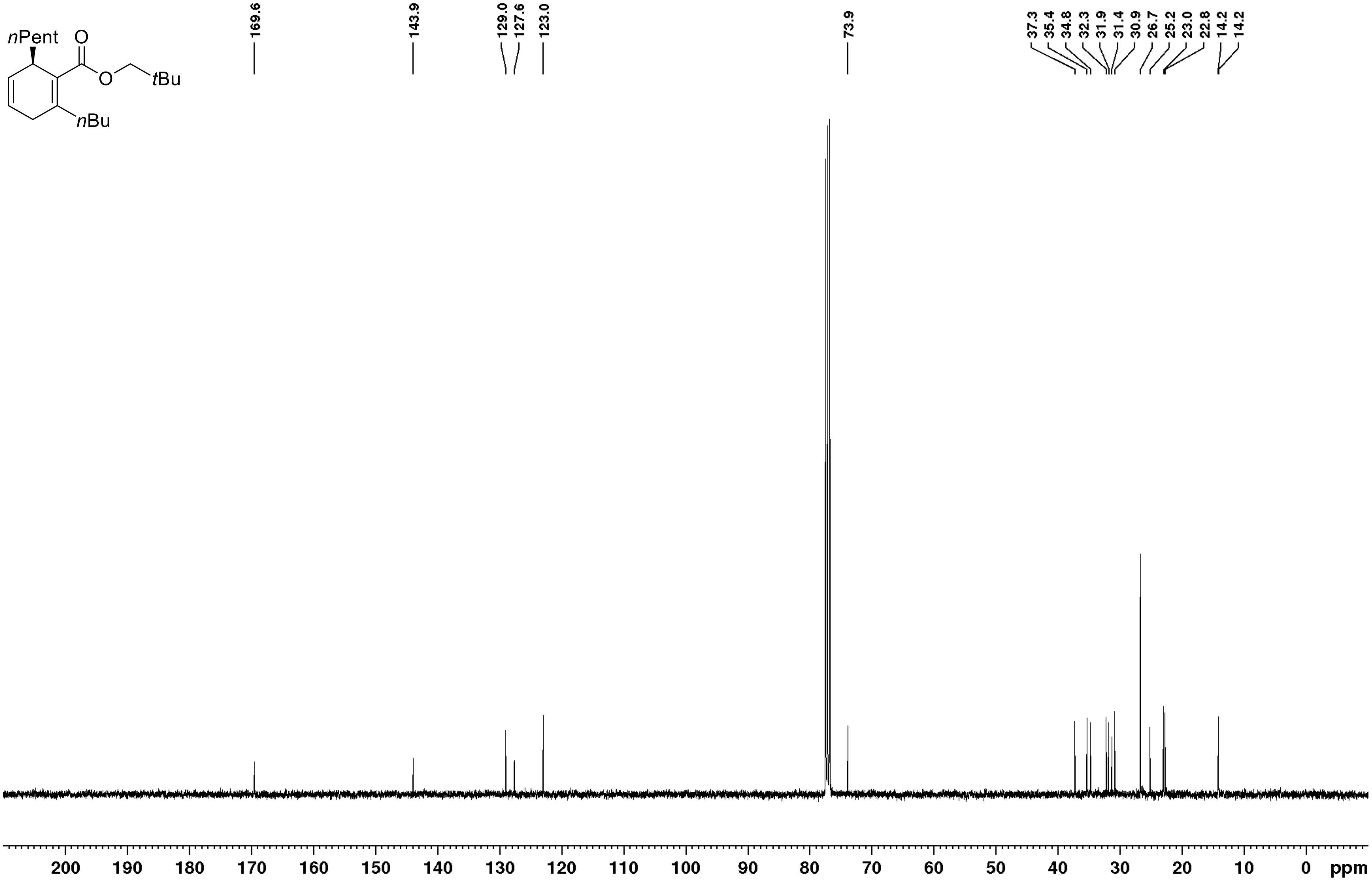

**Figure S111.** $^1$H NMR spectrum (400 MHz, $CDCl_3$, 298 K) of **3ag**.

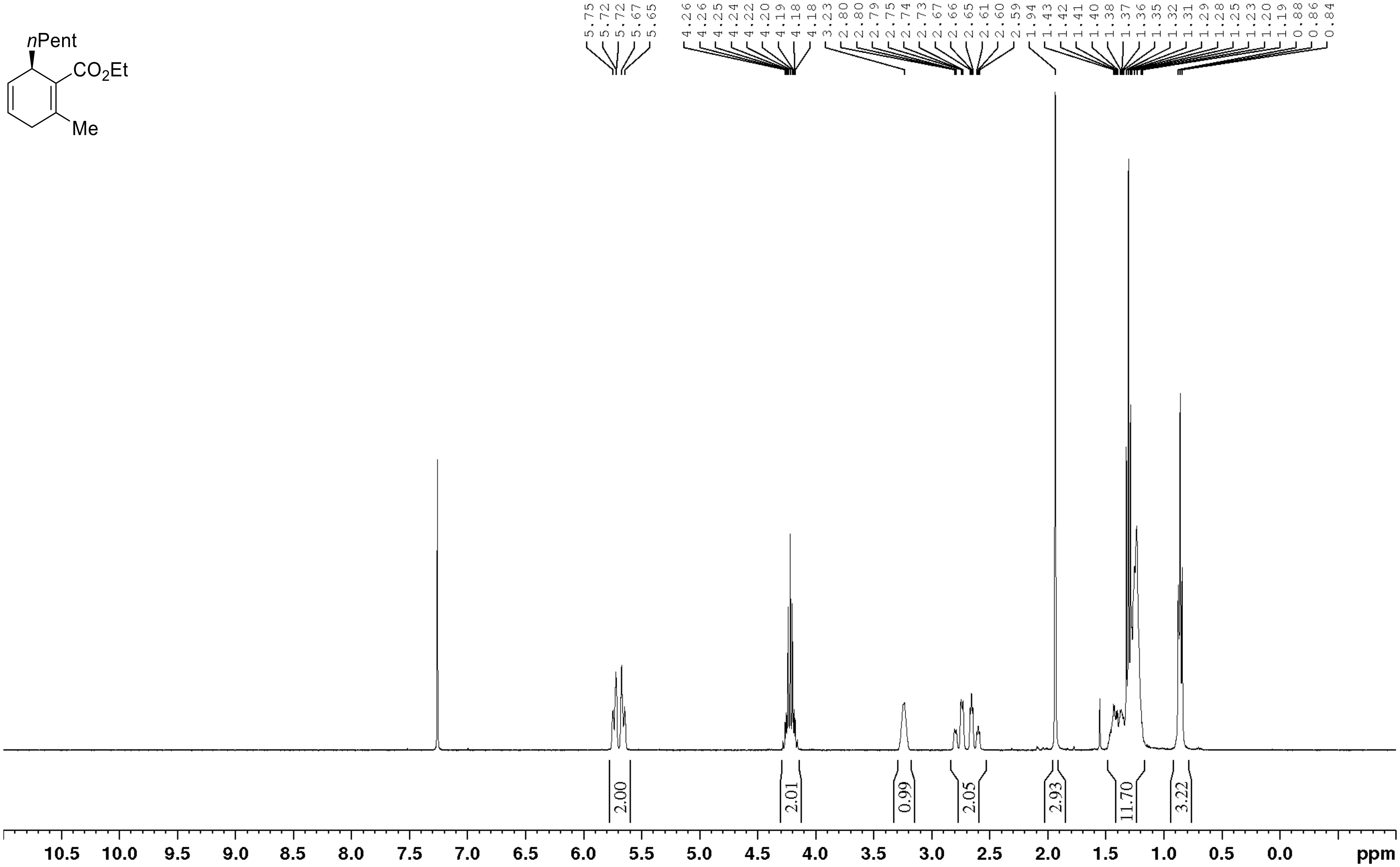

**Figure S112.** $^{13}C\{1H\}$ NMR spectrum (100 MHz, $CDCl_3$, 298 K) of **3ag**.

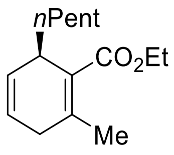


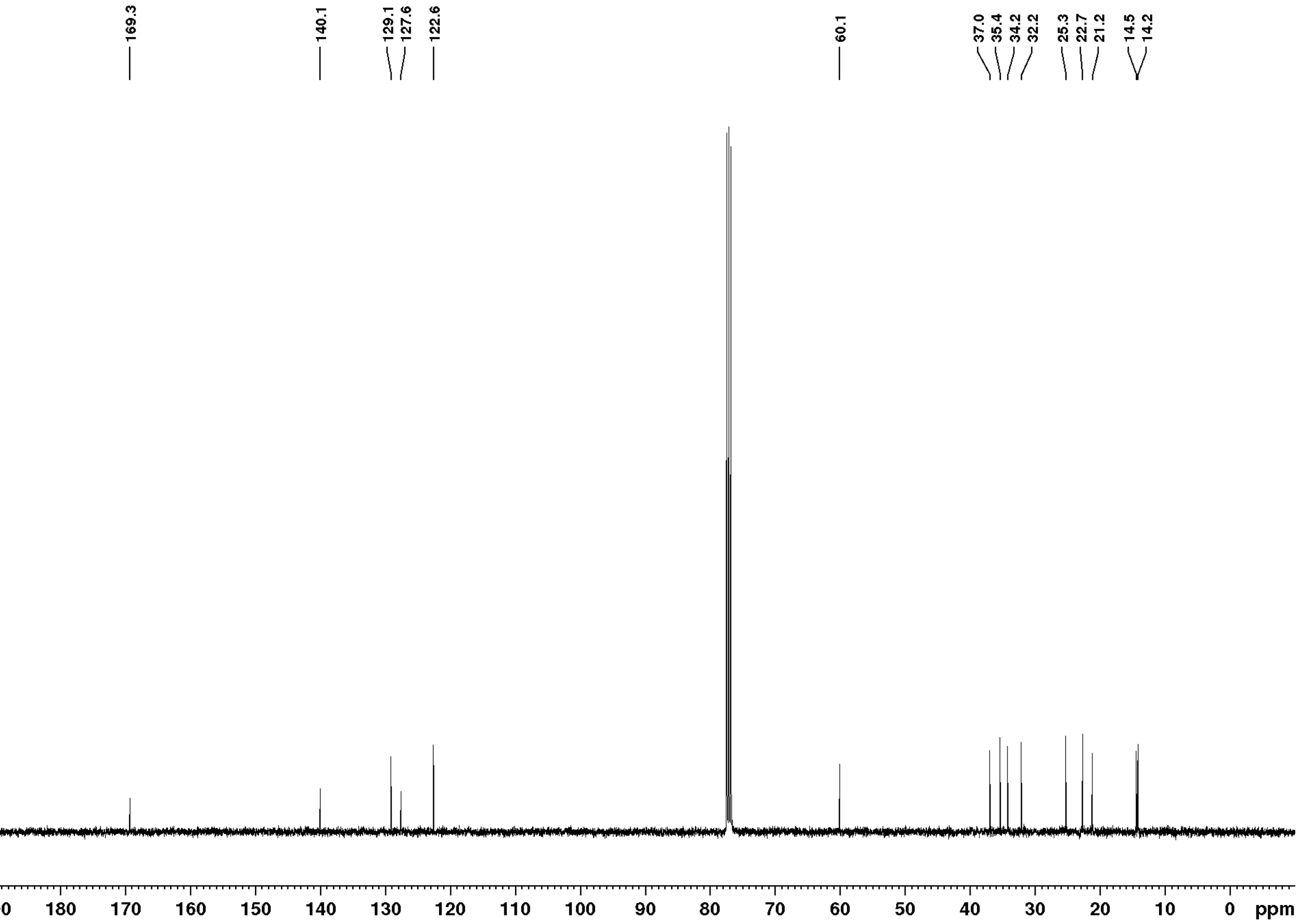

**Figure S113.** $^{1}H$ NMR spectrum (400 MHz, $CDCl_3$, 298 K) of **3ah**.

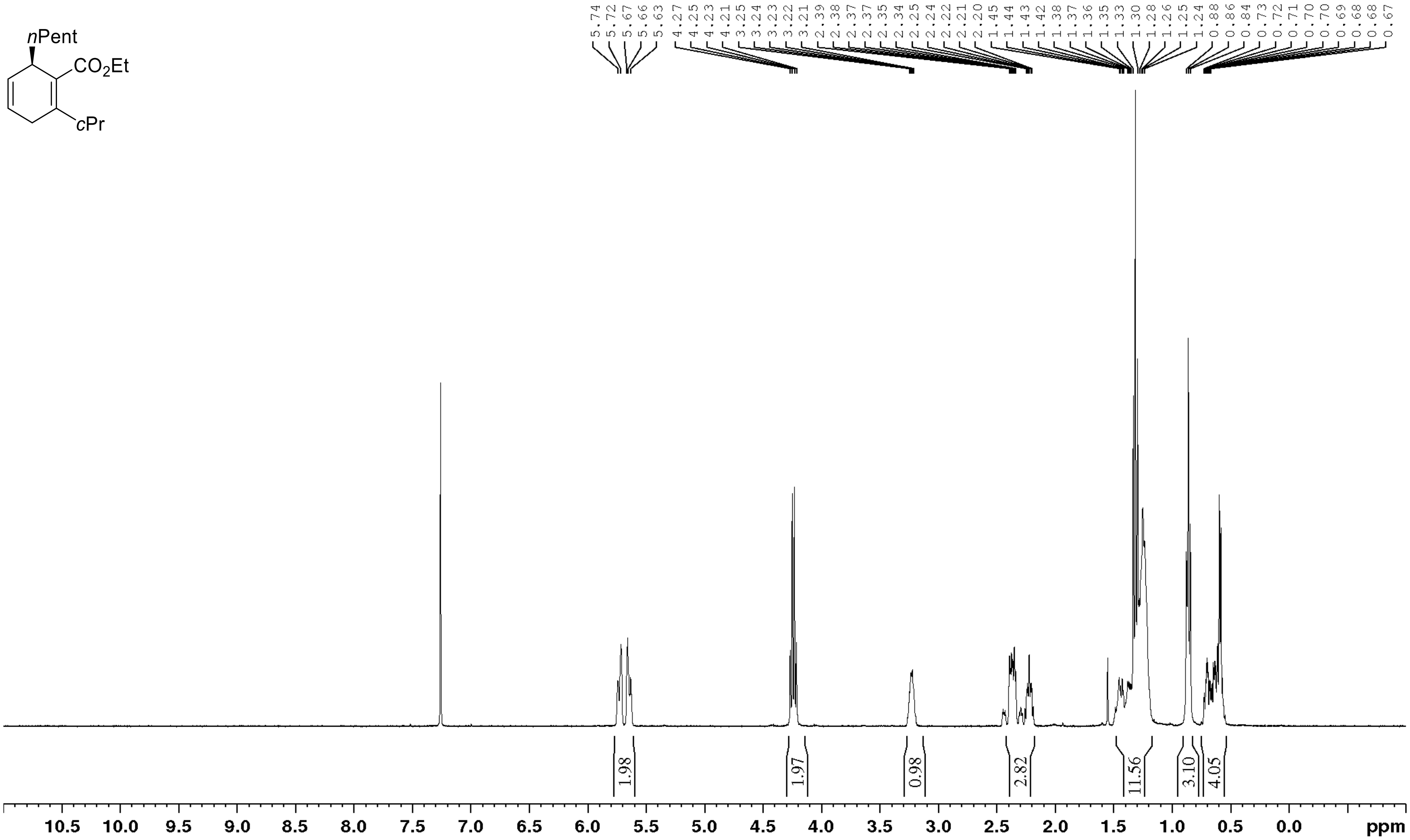

**Figure S114.** $^{13}C\{1H\}$ NMR spectrum (100 MHz, $CDCl_3$, 298 K) of **3ah**.

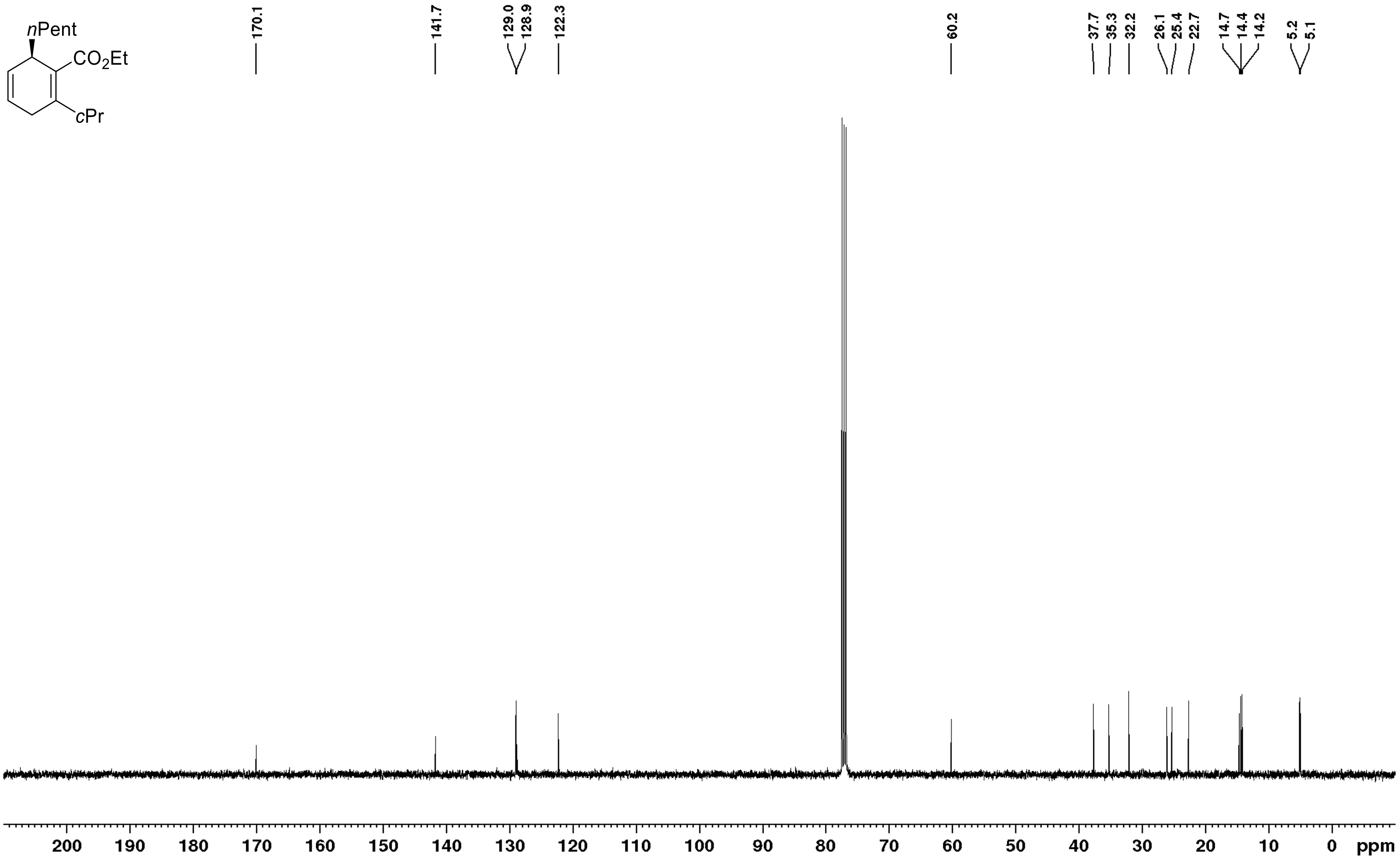

**Figure S115.** $^1$H NMR spectrum (400 MHz, $CDCl_3$, 298 K) of **3ai**.

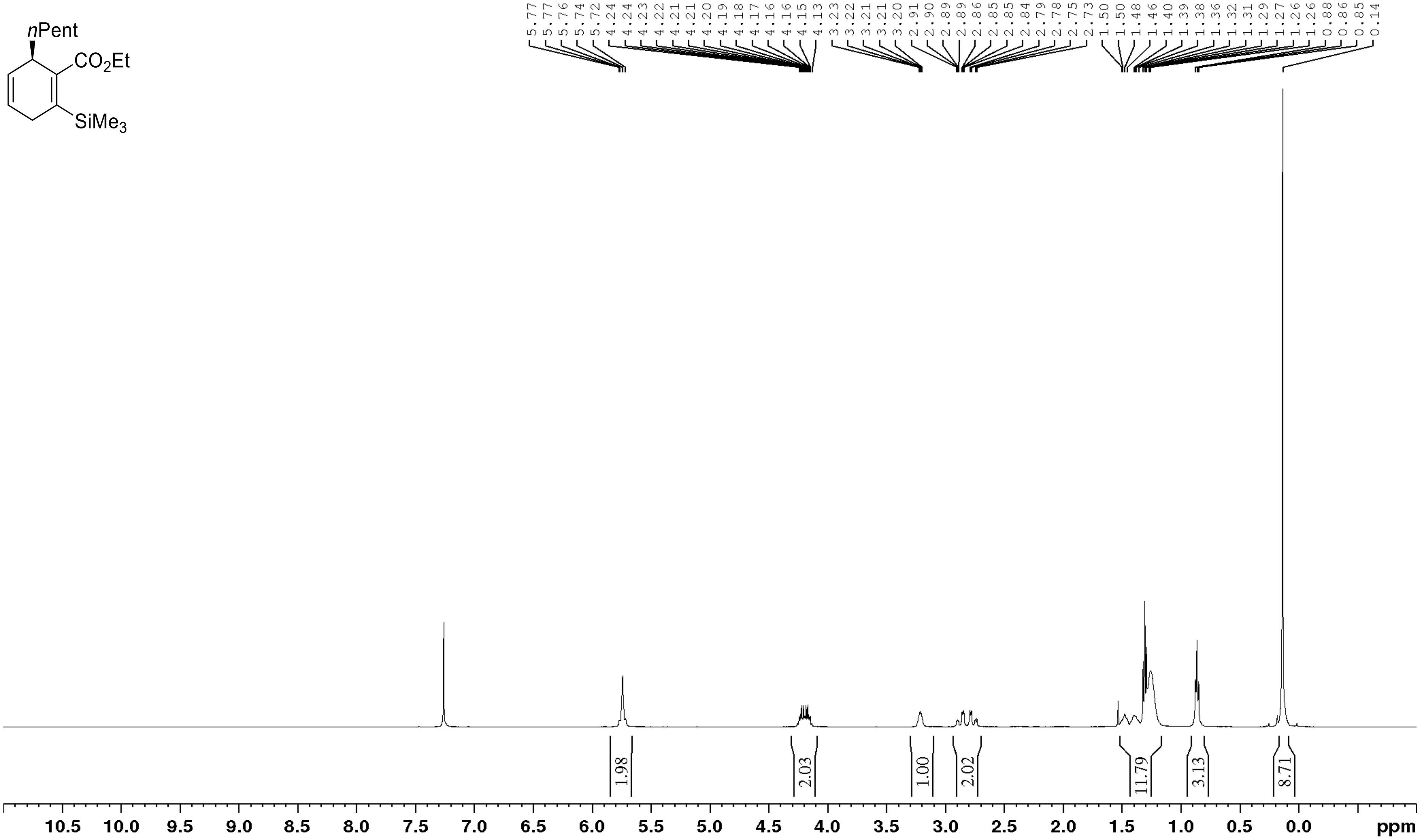

**Figure S116.** $^{13}C\{1H\}$ NMR spectrum (100 MHz, $CDCl_3$, 298 K) of **3ai**.

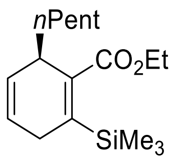


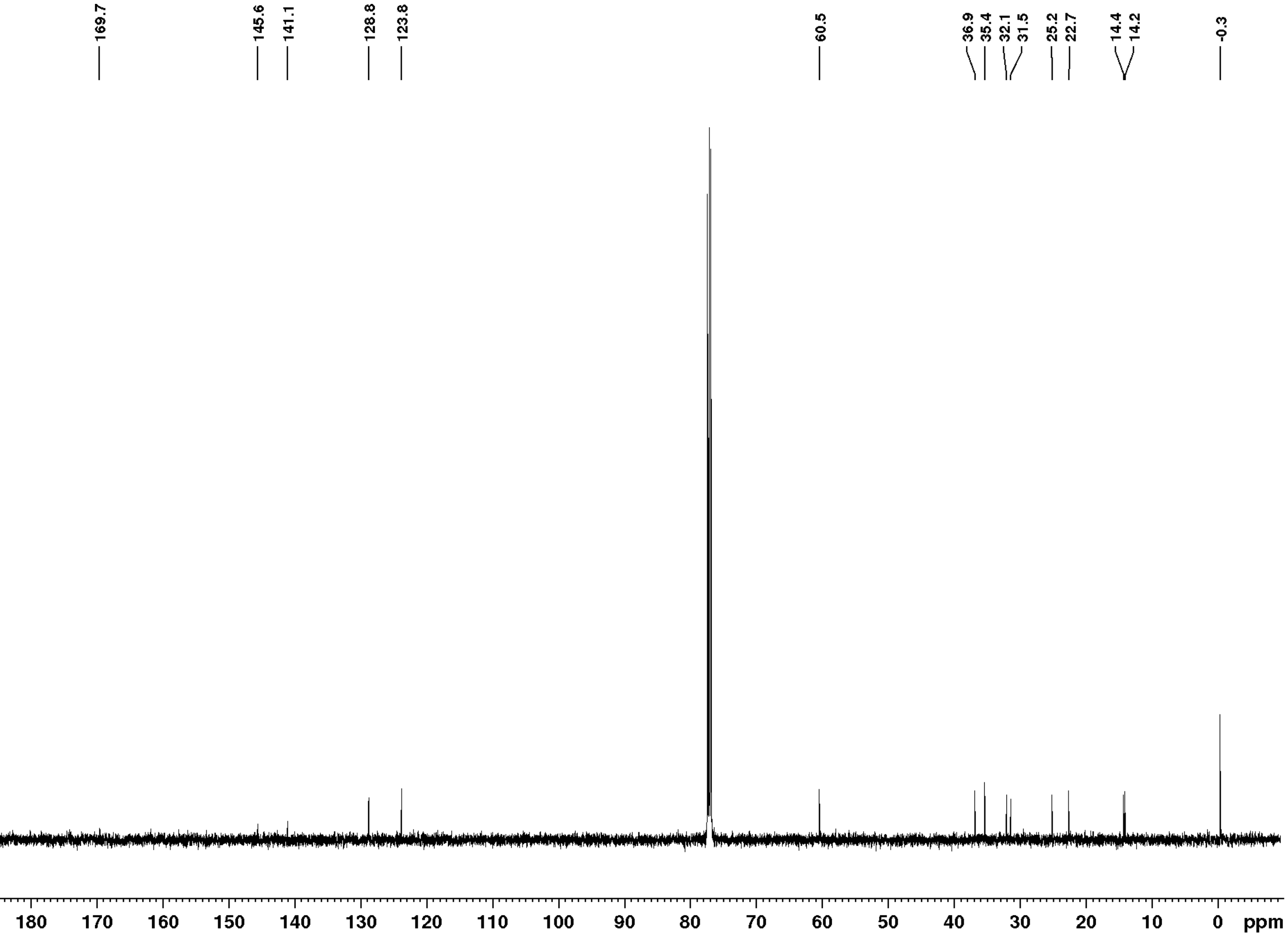

**Figure S117.** $^{1}H$ NMR spectrum (400 MHz, $CDCl_3$, 298 K) of **3aj**.

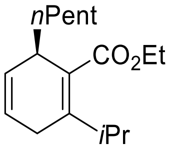


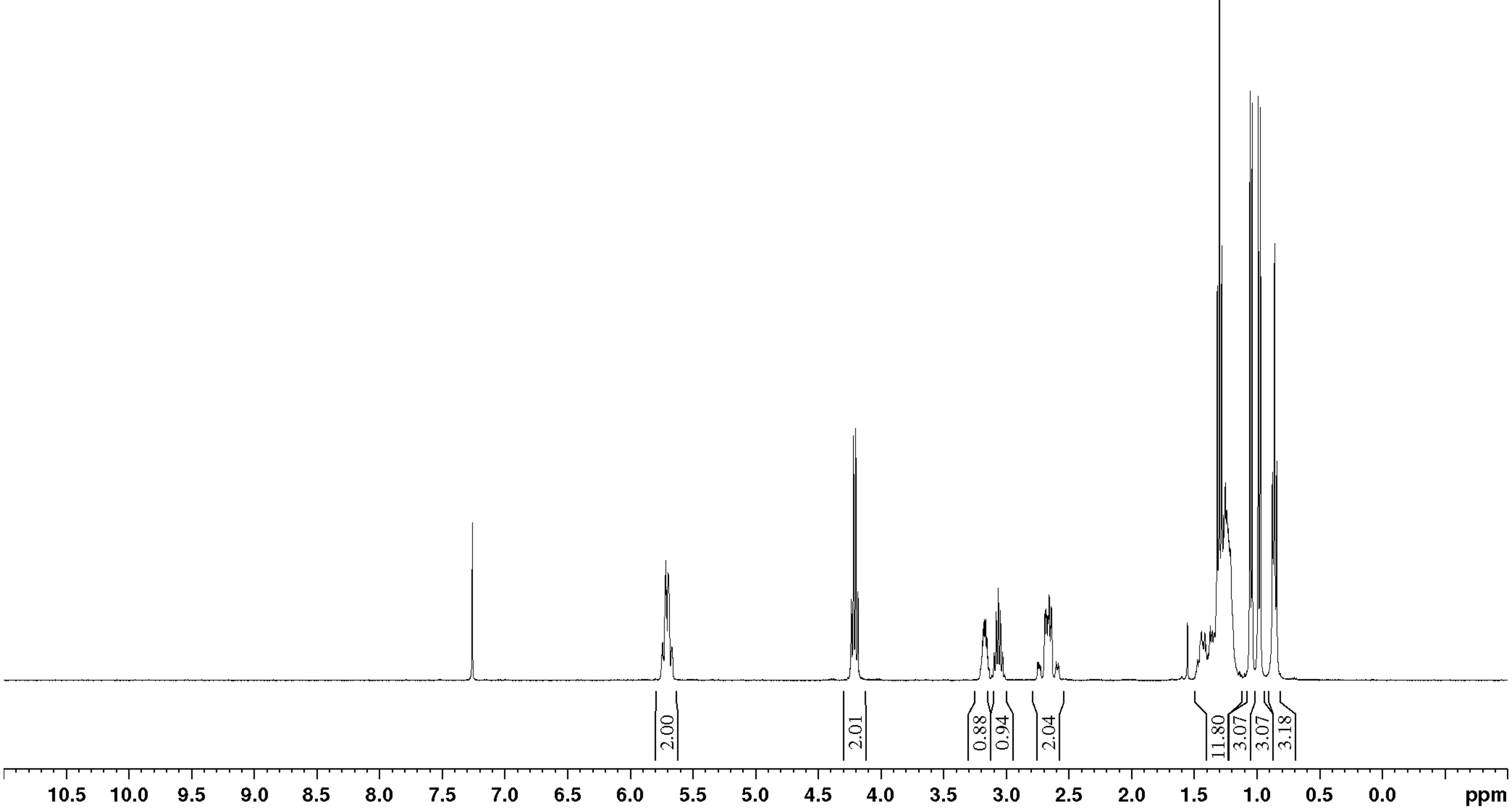

**Figure S118.** $^{13}C\{1H\}$ NMR spectrum (100 MHz, $CDCl_3$, 298 K) of **3aj**.

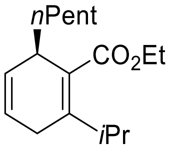


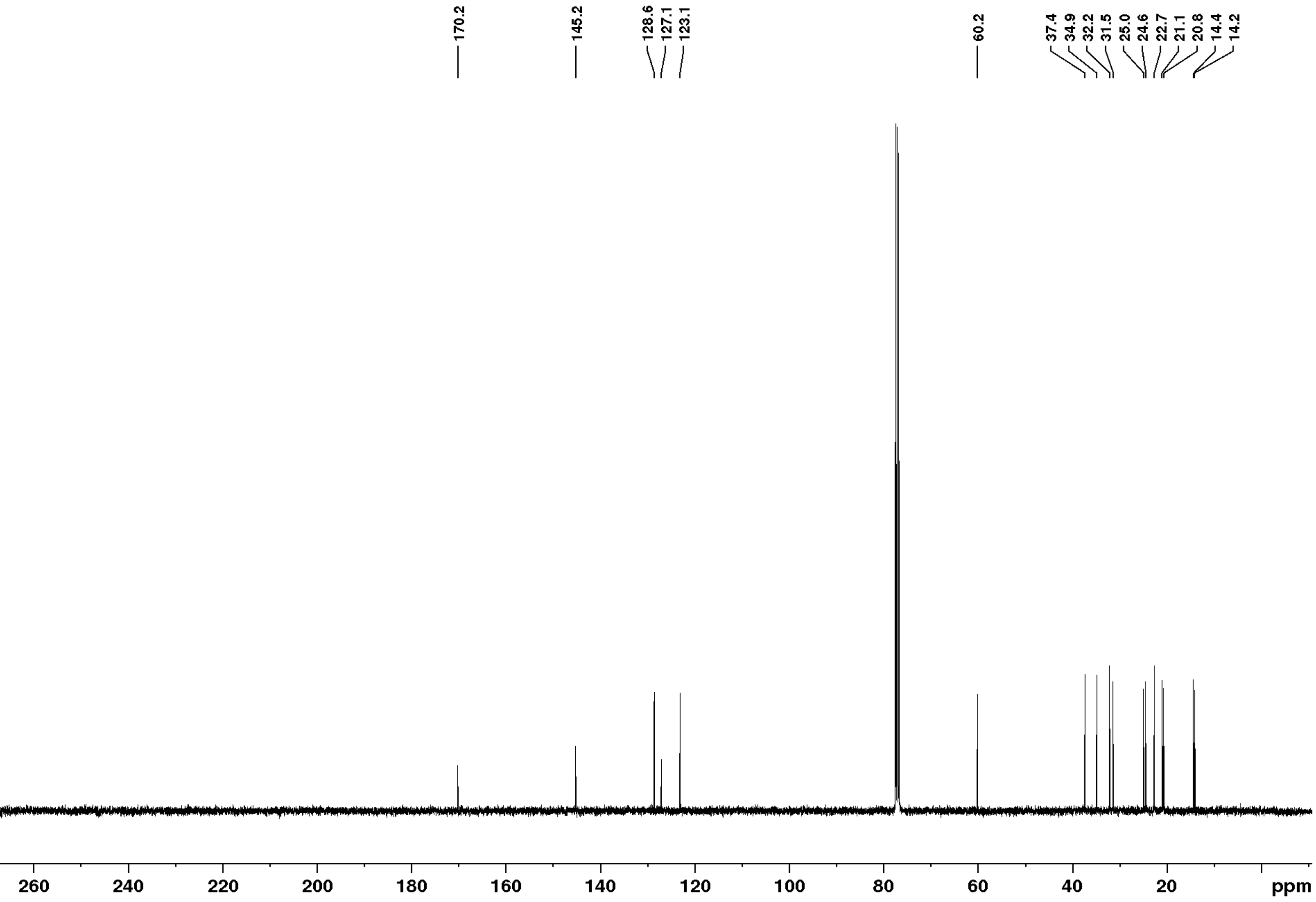

**Figure S119.** $^1$H NMR spectrum (400 MHz, $CDCl_3$, 298 K) of **3ak**.

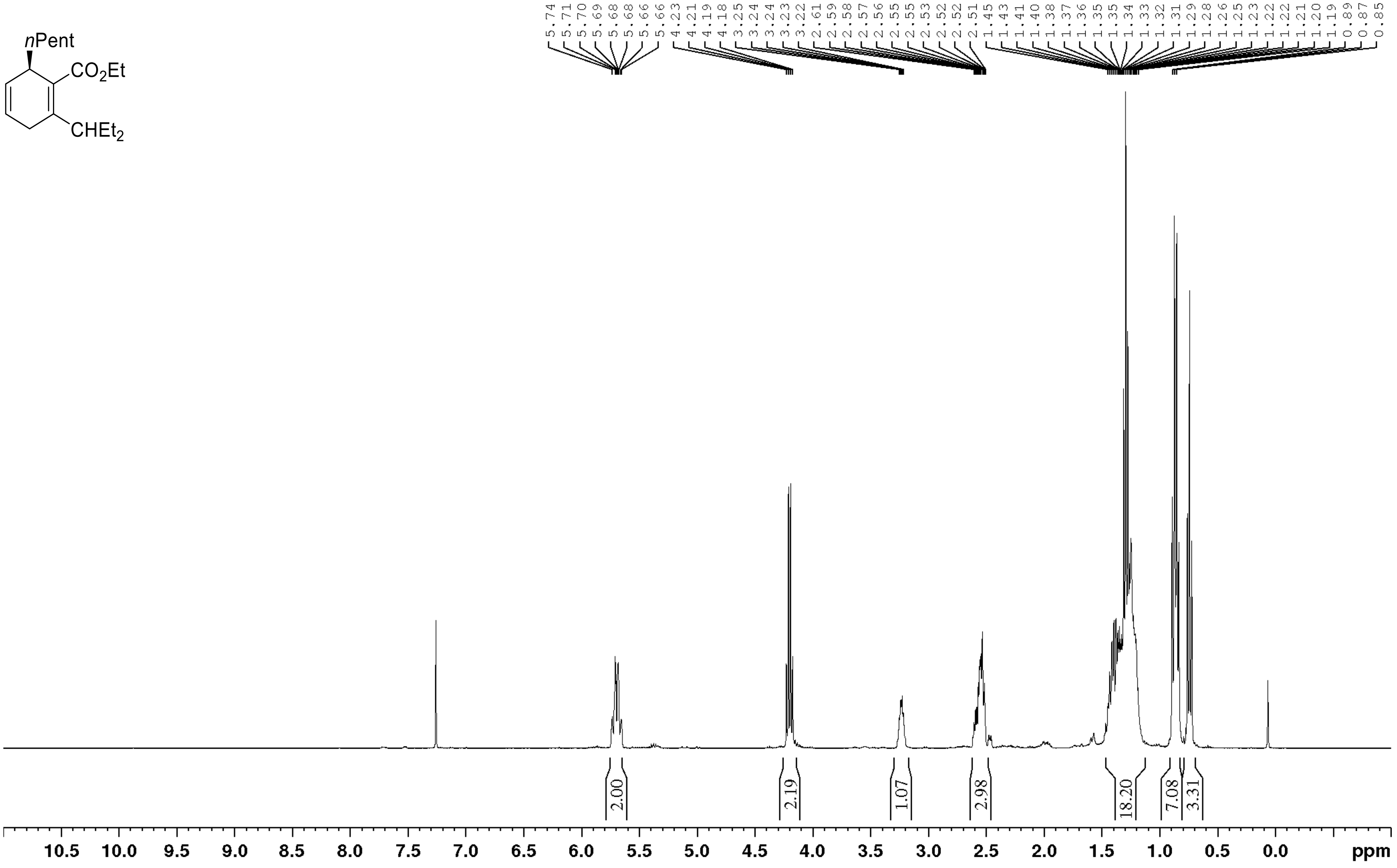

**Figure S120.** $^{13}C\{1H\}$ NMR spectrum (100 MHz, $CDCl_3$, 298 K) of **3ak**.

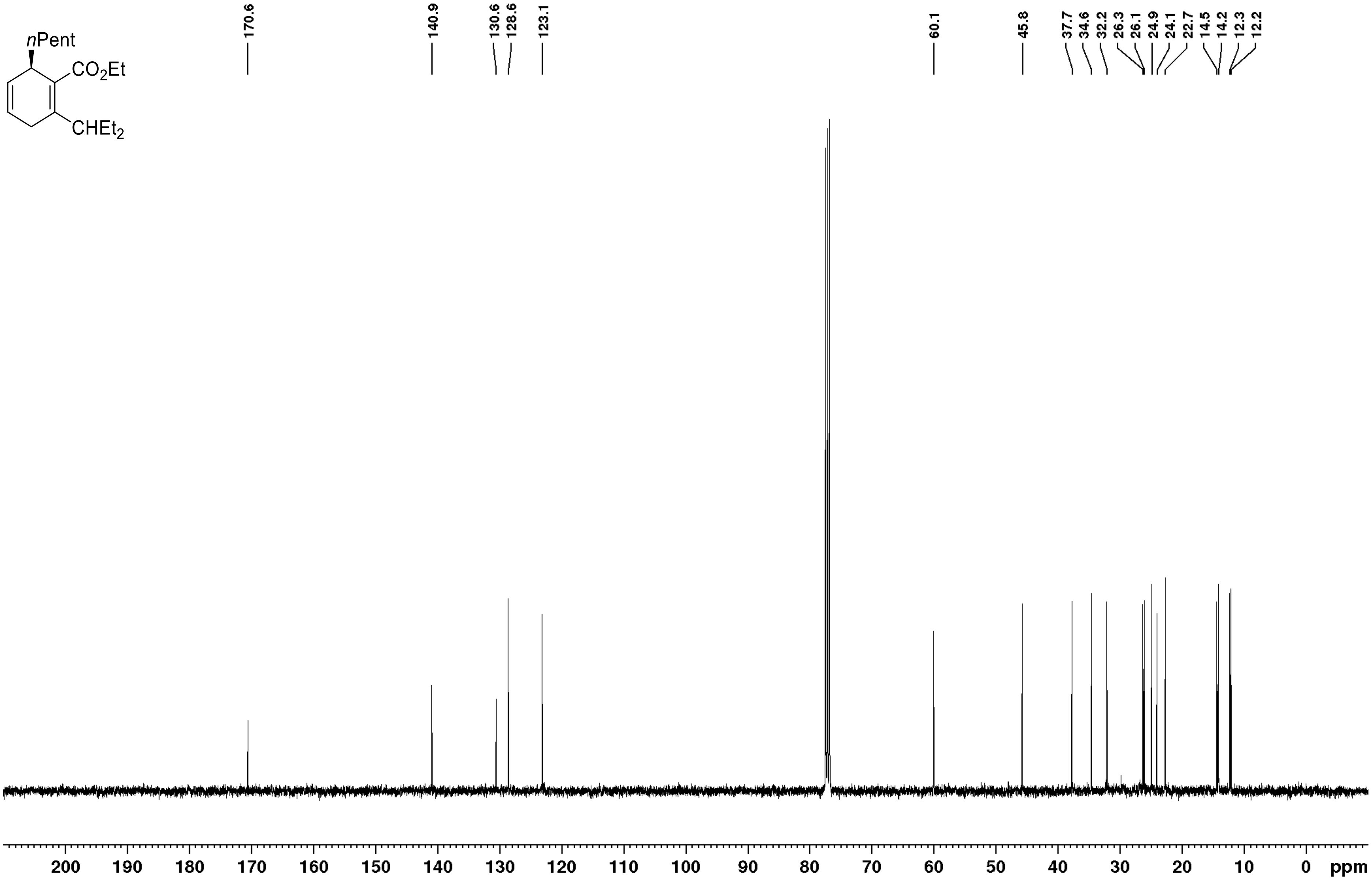

**Figure S121.** $^1$H NMR spectrum (400 MHz, $CDCl_3$, 298 K) of **3al**.

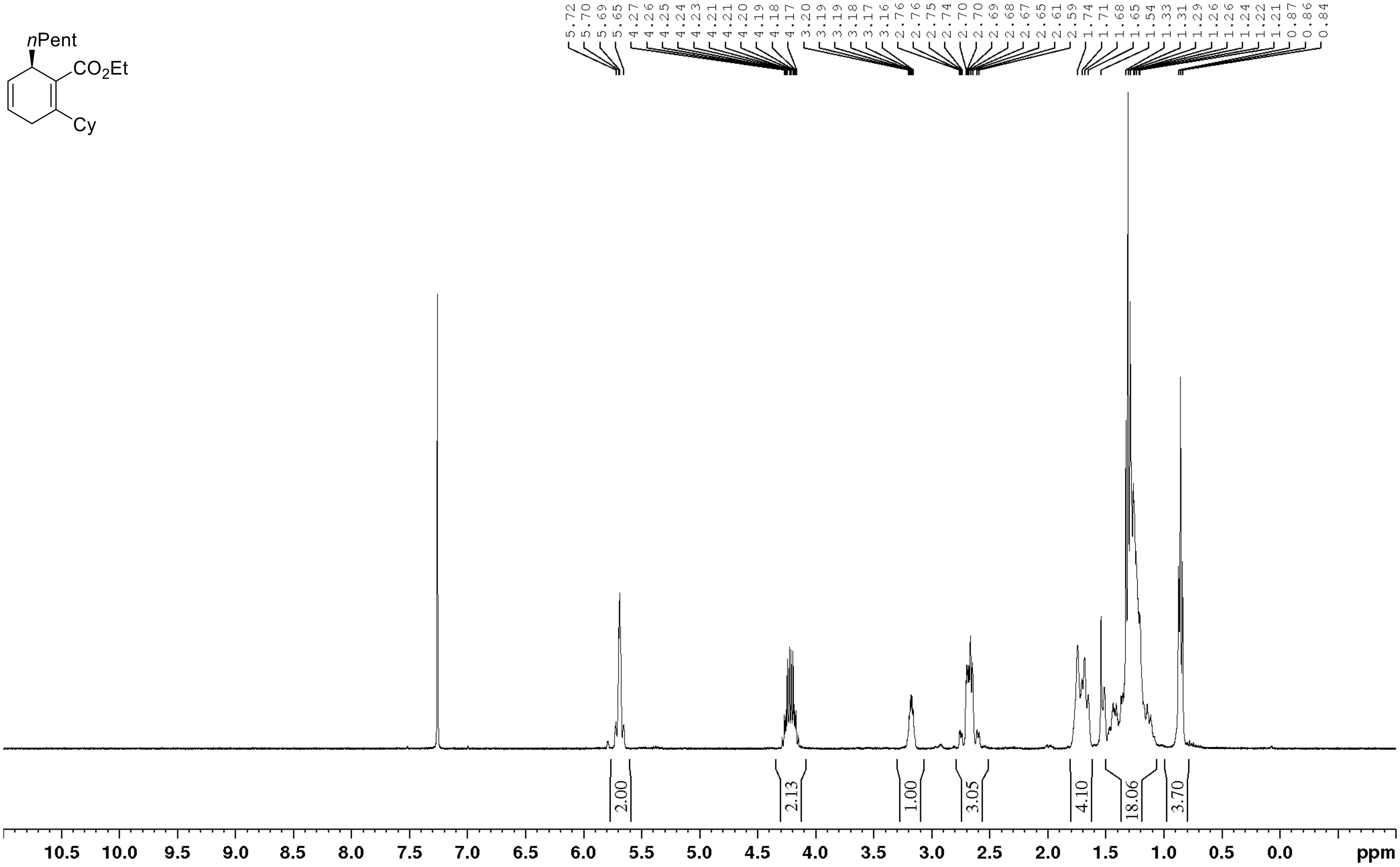

**Figure S122.** $^{13}C\{1H\}$ NMR spectrum (100 MHz, $CDCl_3$, 298 K) of **3al**.

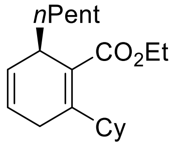

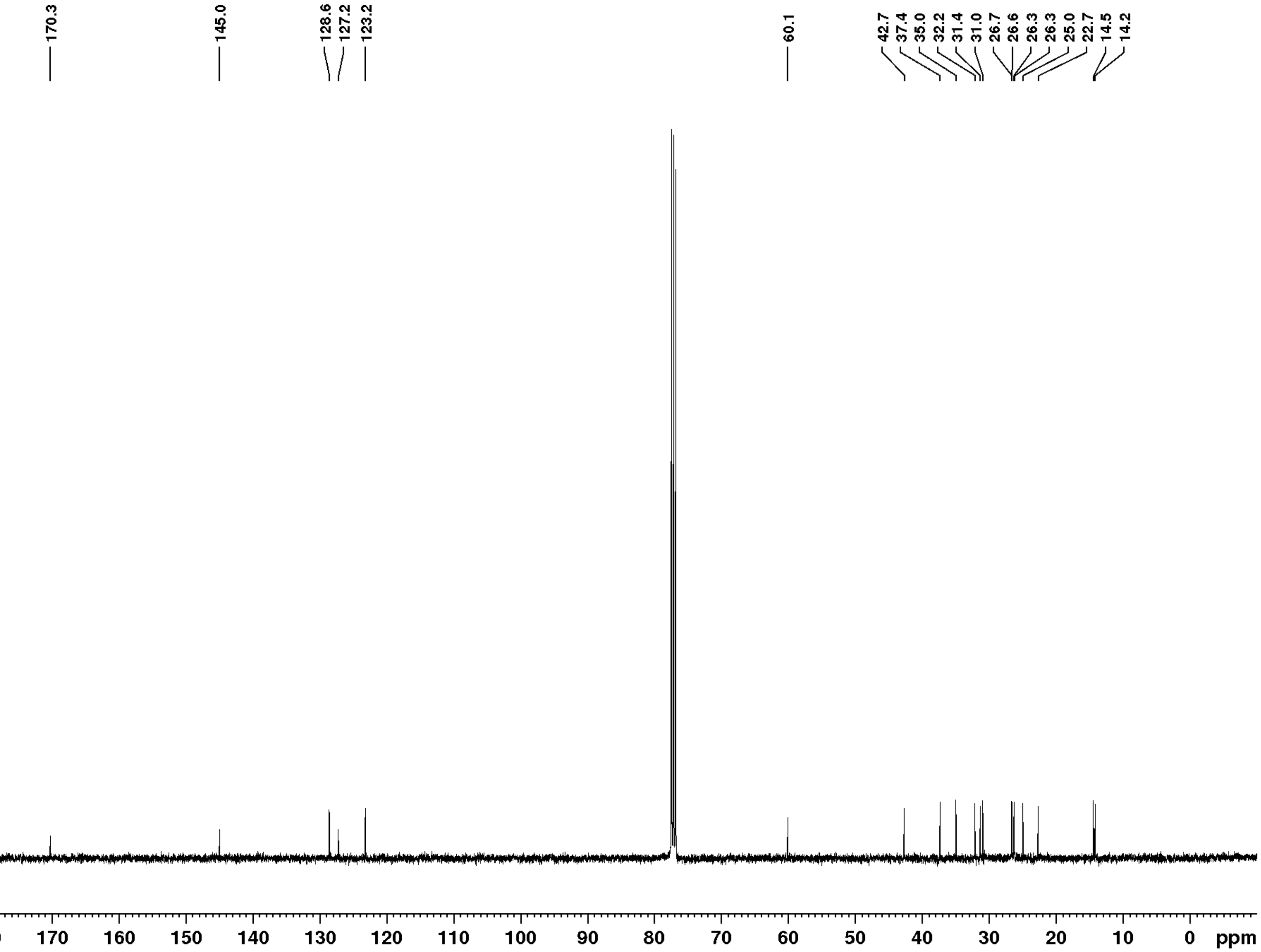

**Figure S123.** $^{1}$H NMR spectrum (400 MHz, $CDCl_3$, 298 K) of **3am**.

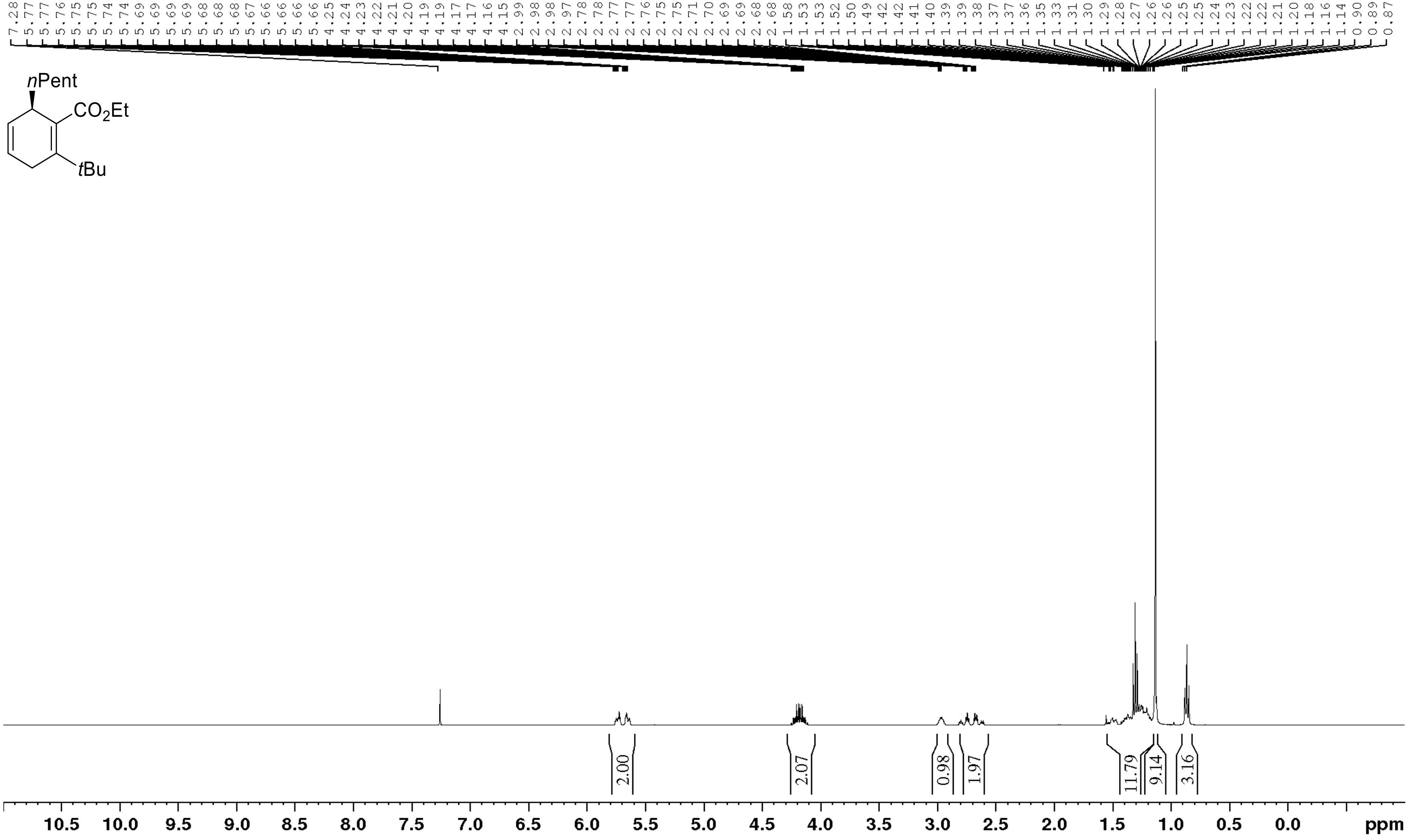

**Figure S124.** $^{13}C\{1H\}$ NMR spectrum (100 MHz, $CDCl_3$, 298 K) of **3am**.

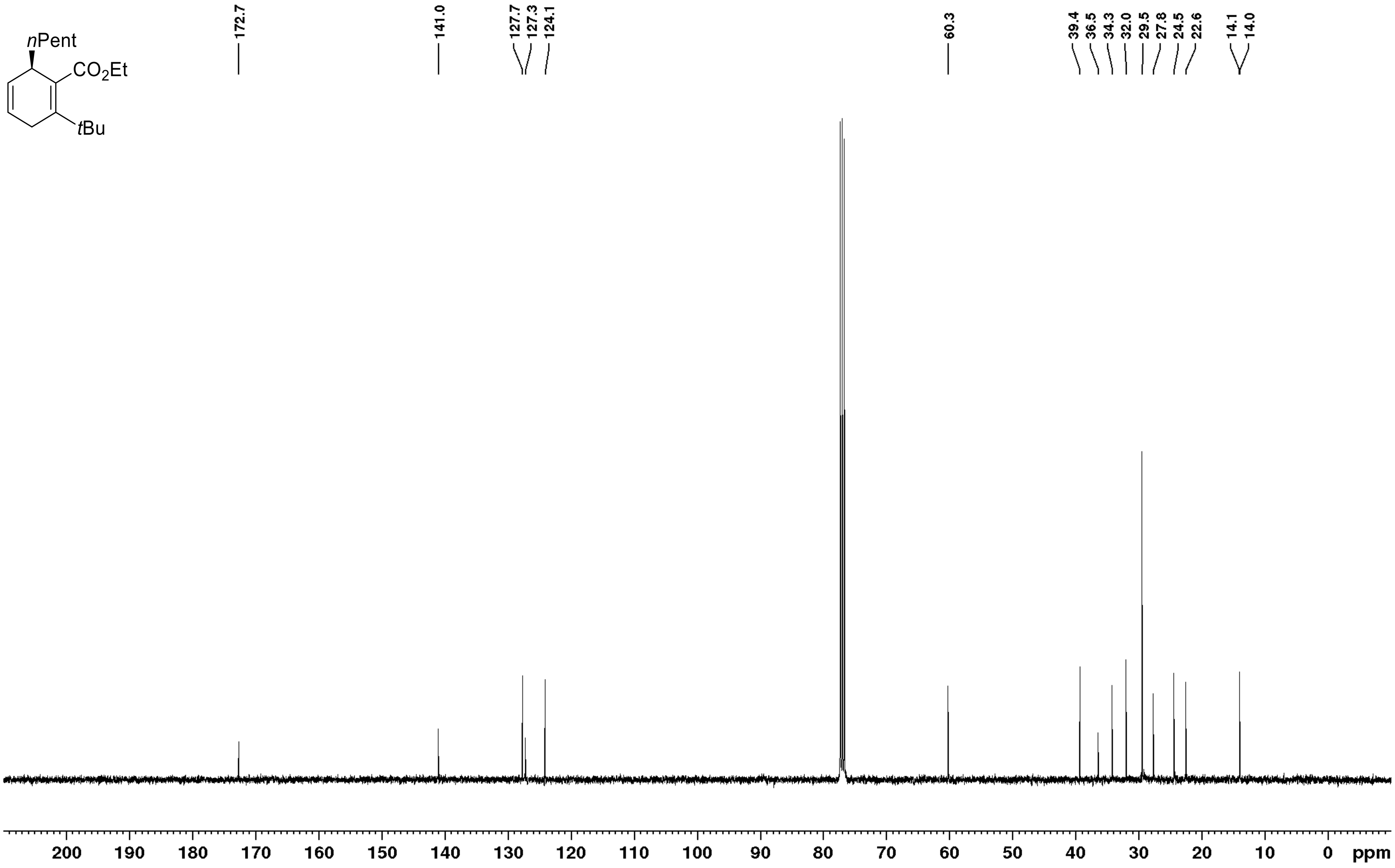

**Figure S125.** $^1H$ NMR spectrum (400 MHz, $CDCl_3$, 298 K) of **3bb**.

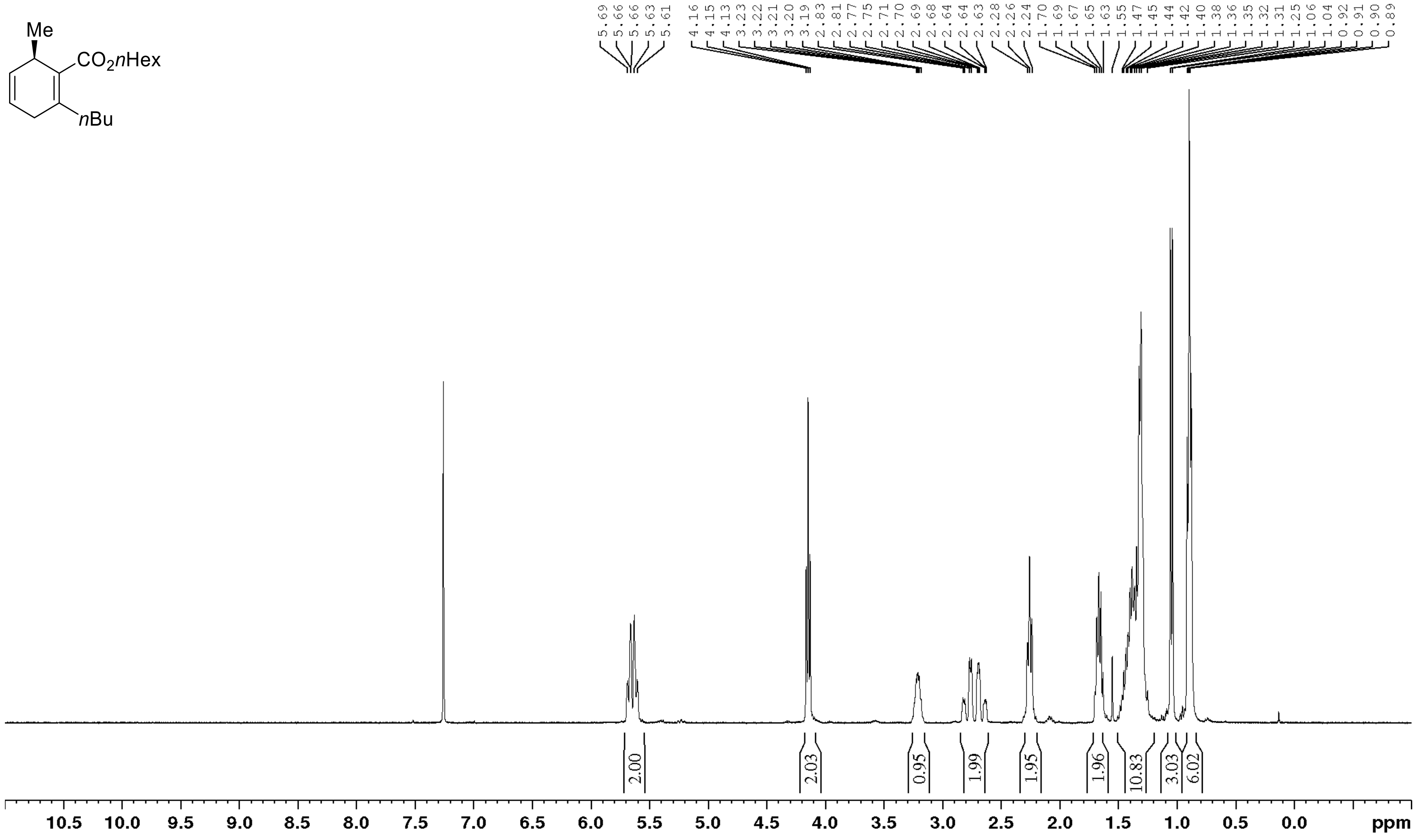

**Figure S126.** $^{13}C\{^1H\}$ NMR spectrum (100 MHz, $CDCl_3$, 298 K) of **3bb**.

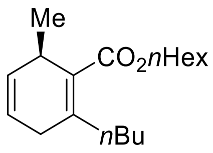


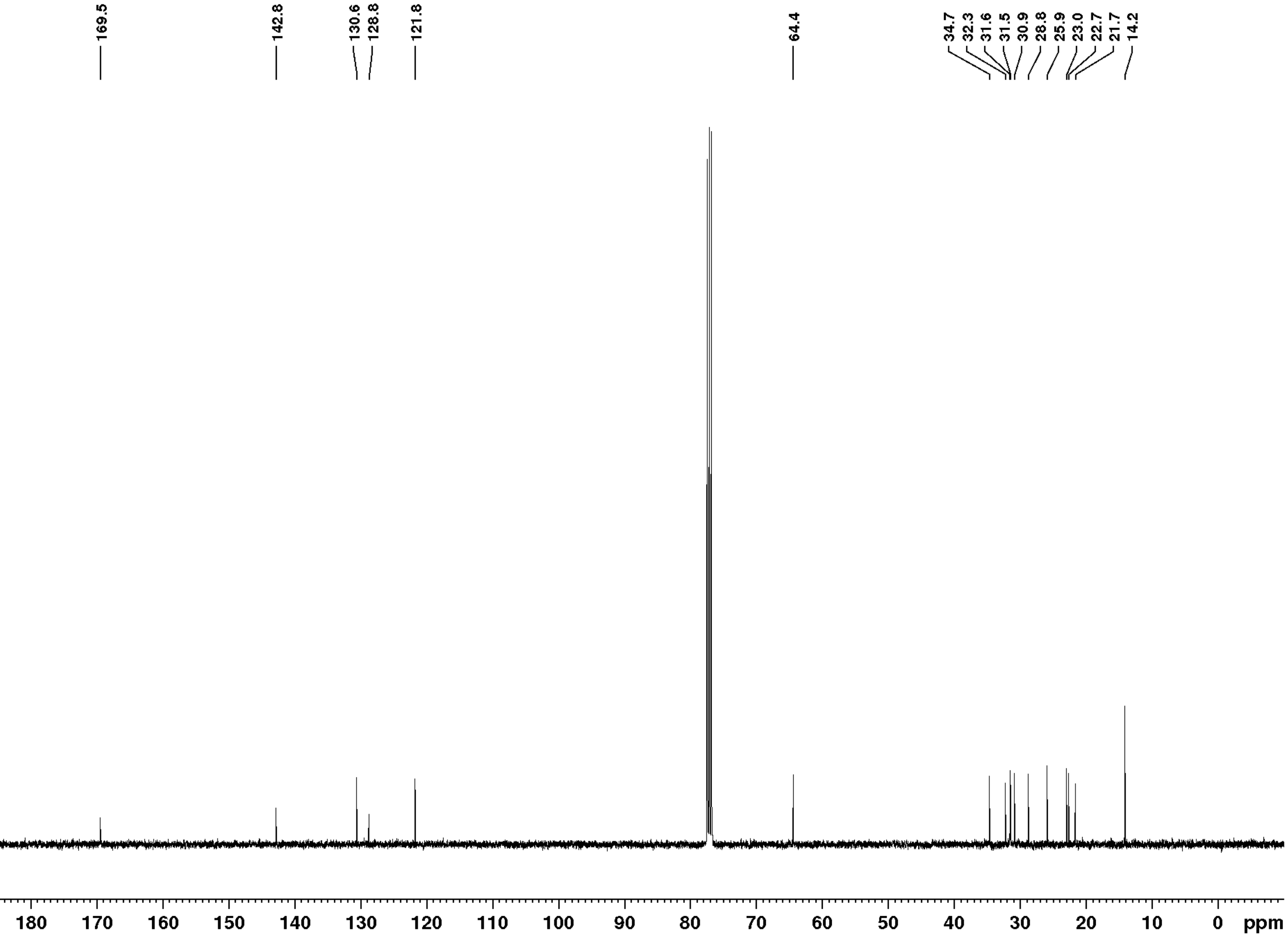

**Figure S127.** $^{1}$H NMR spectrum (400 MHz, $CDCl_3$, 298 K) of **3cb**.

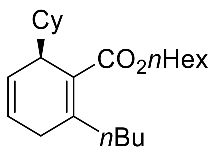


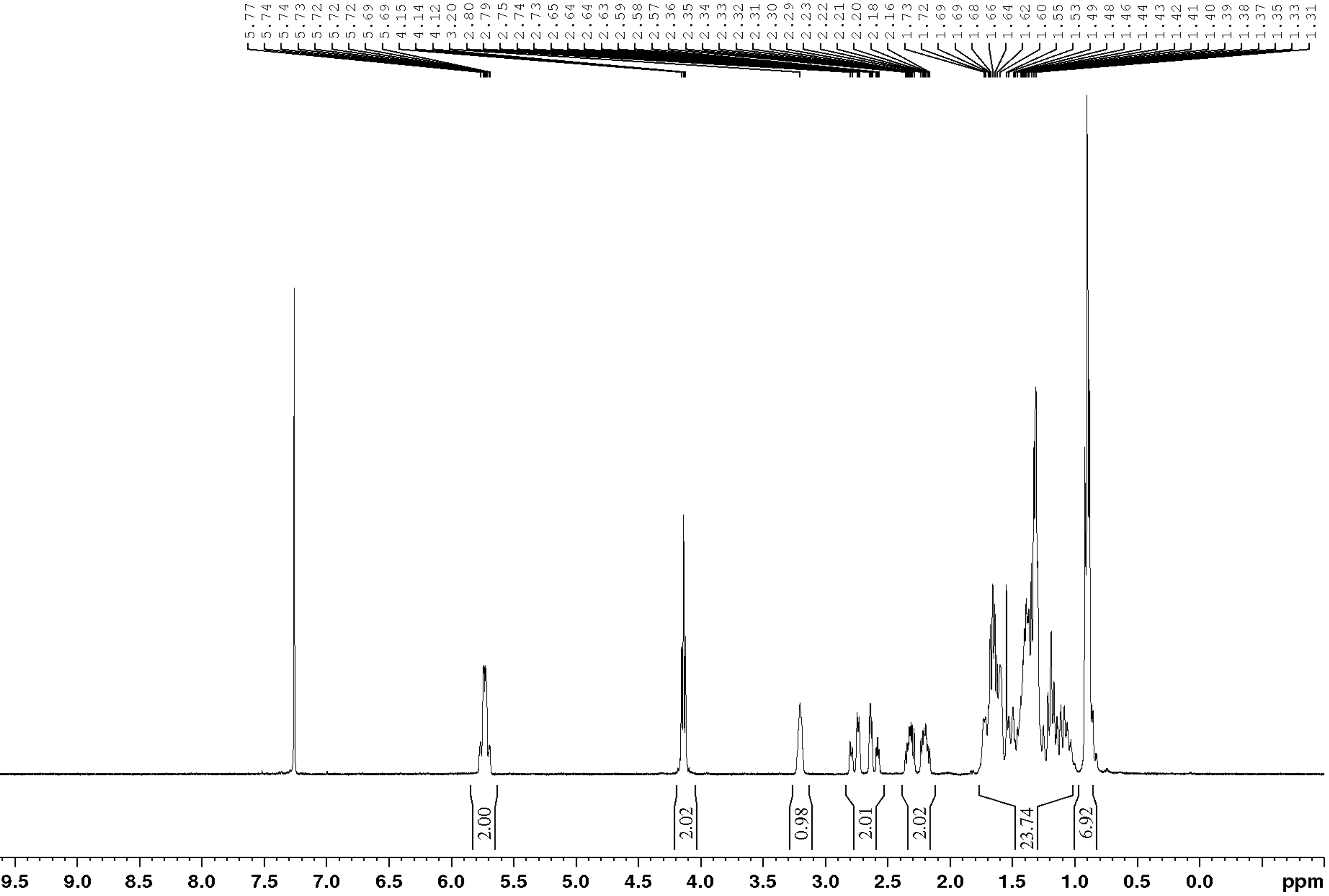

**Figure S128.** $^{13}C\{^1H\}$ NMR spectrum (100 MHz, $CDCl_3$, 298 K) of **3cb**.

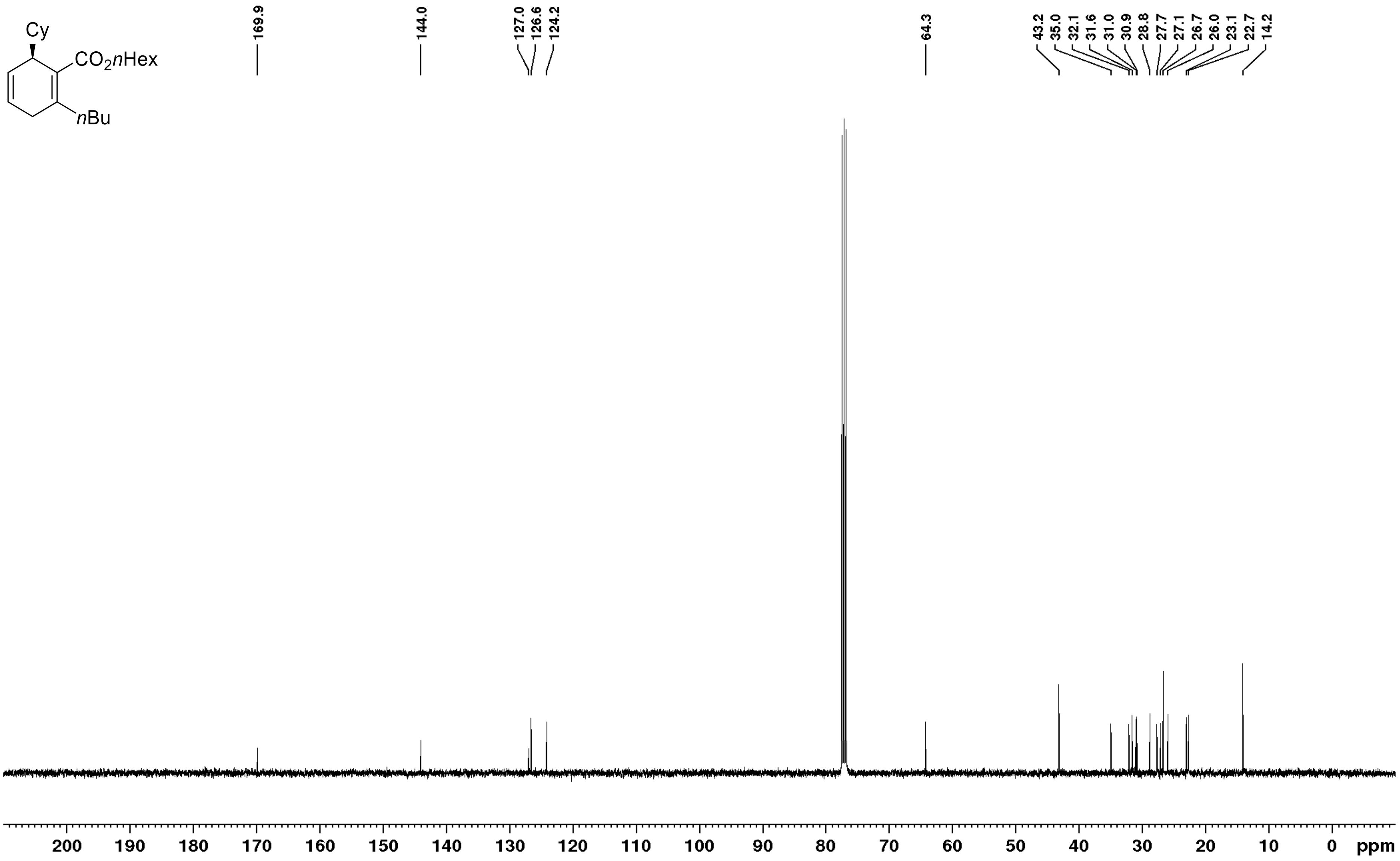

**Figure S129.** $^{1}H$ NMR spectrum (400 MHz, $CDCl_3$, 298 K) of **5a**.

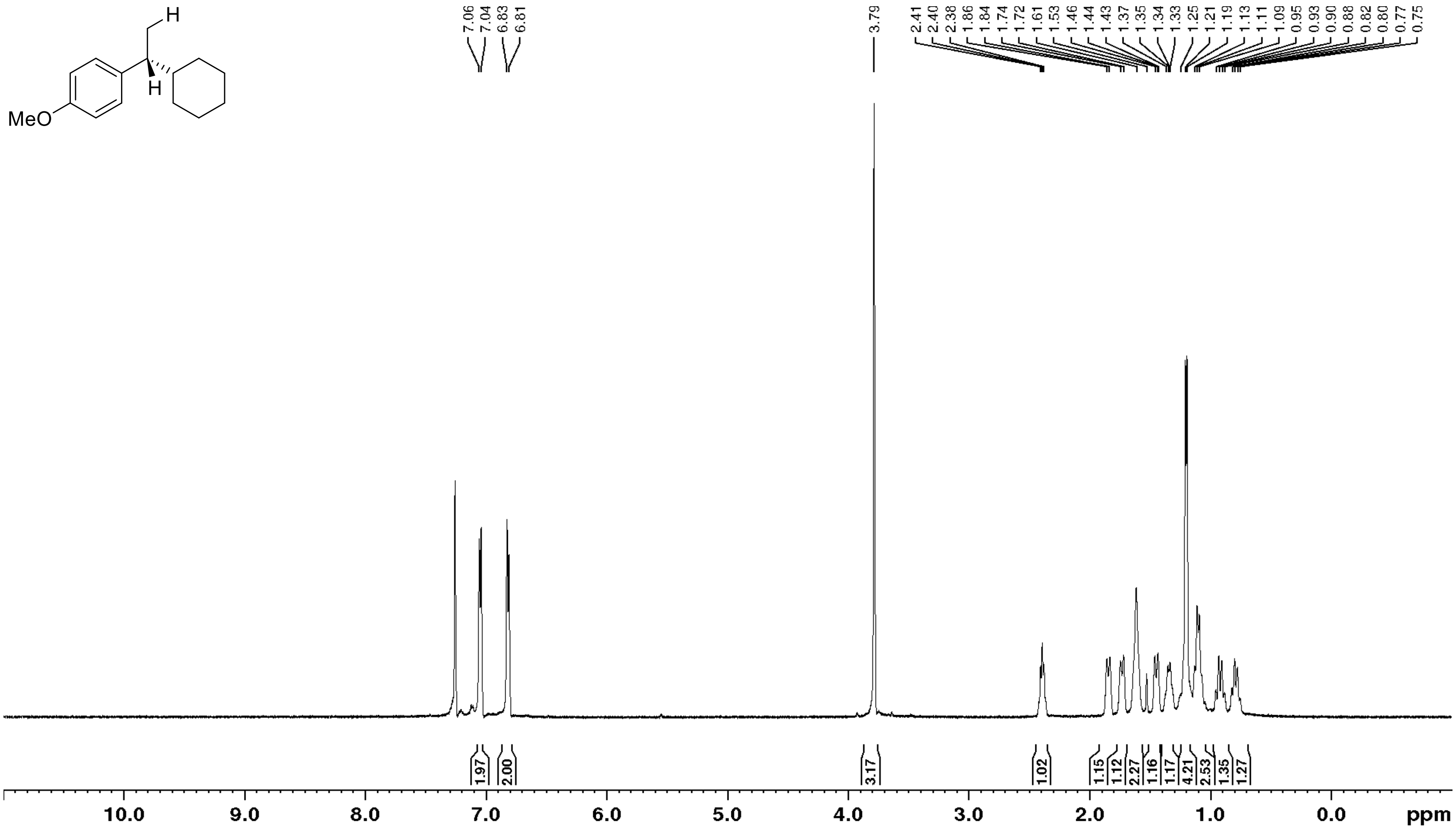

**Figure S130.** $^{13}C\{1H\}$ NMR spectrum (100 MHz, $CDCl_3$, 298 K) of **5a**.

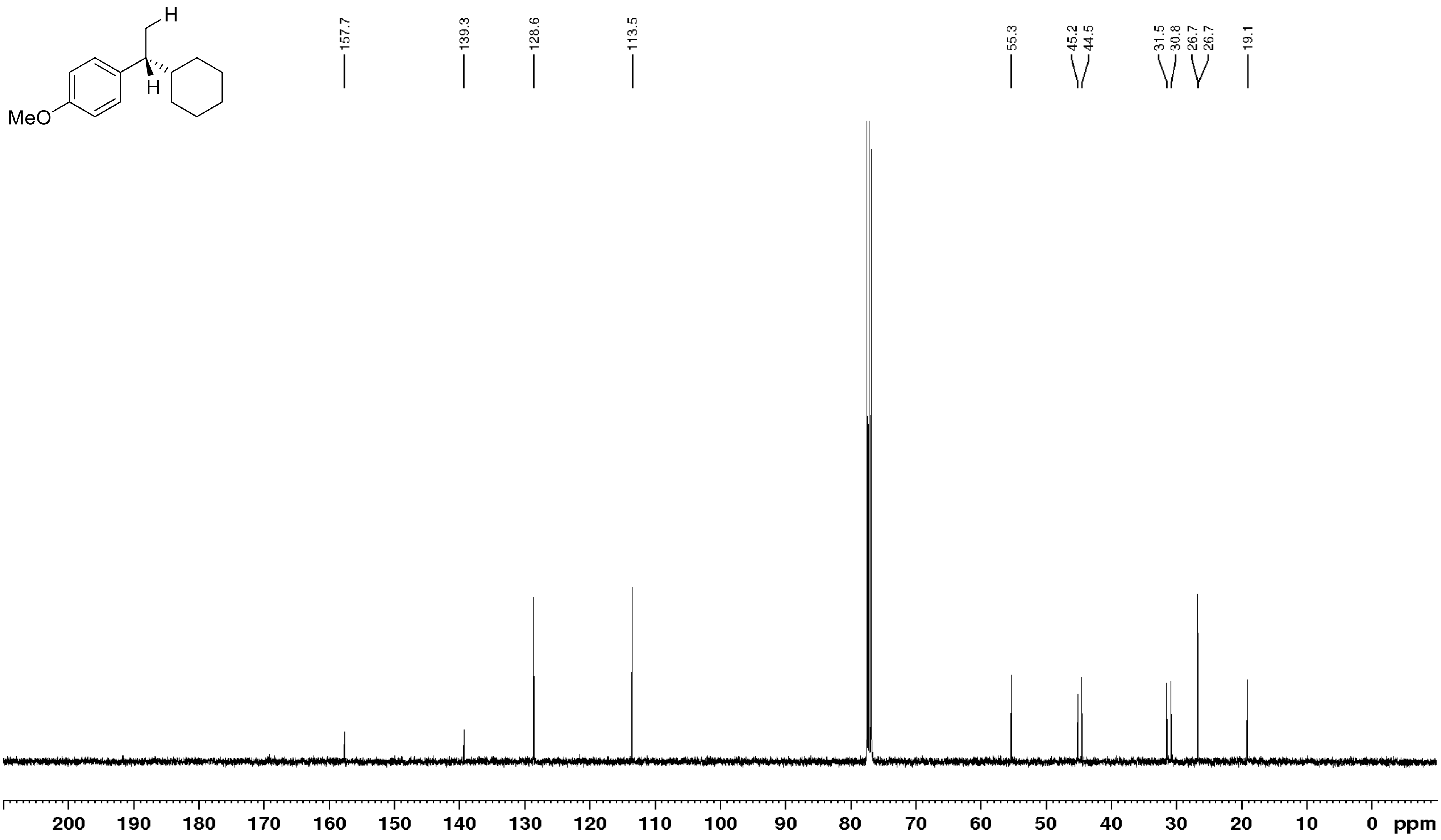

**Figure S131.** $^{1}$H NMR spectrum (400 MHz, $CDCl_3$, 298 K) of **5b**.

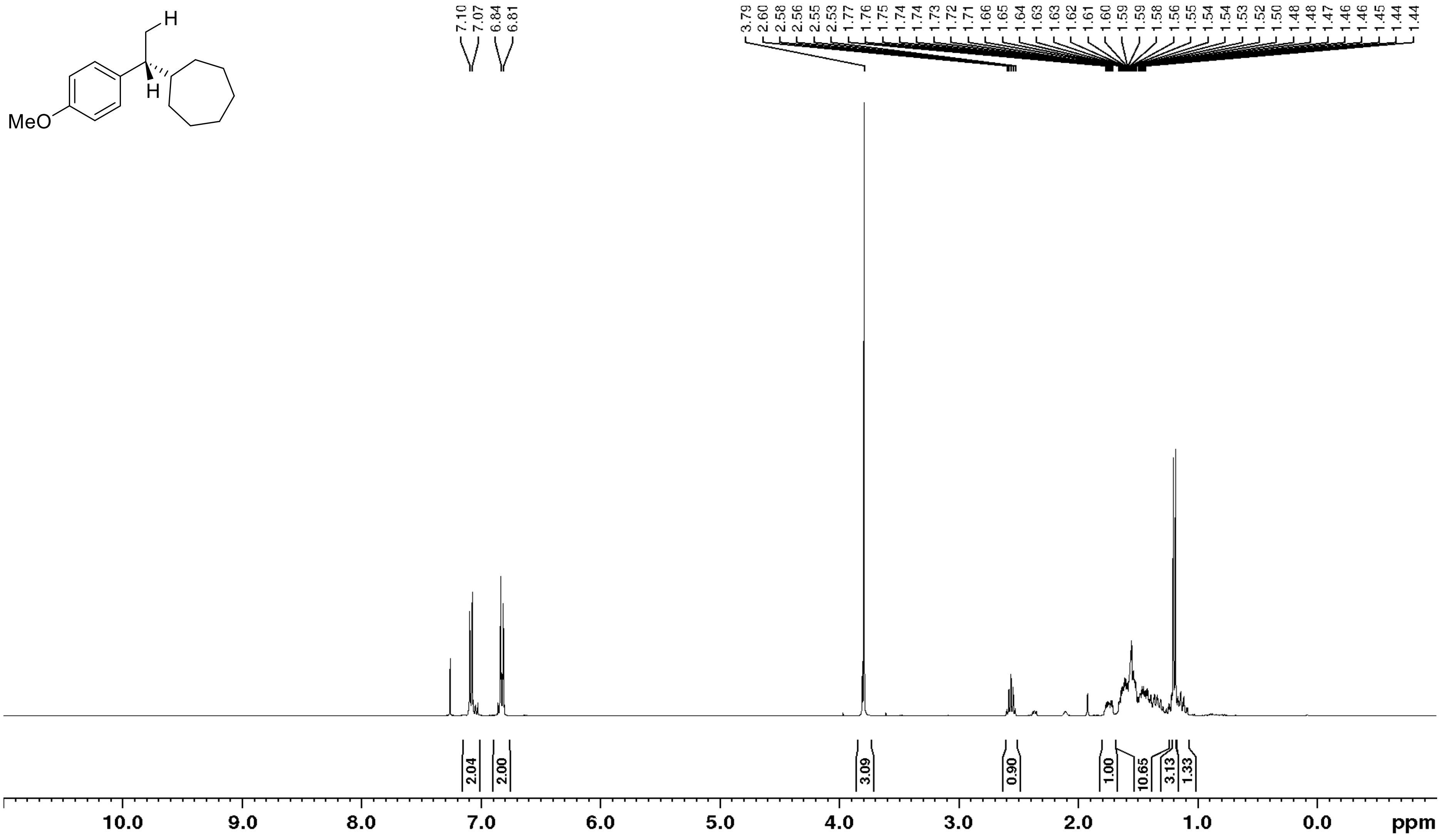

**Figure S132.** $^{13}C\{1H\}$ NMR spectrum (100 MHz, $CDCl_3$, 298 K) of **5b**.

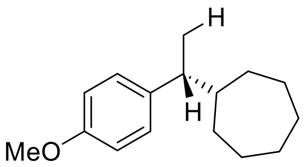


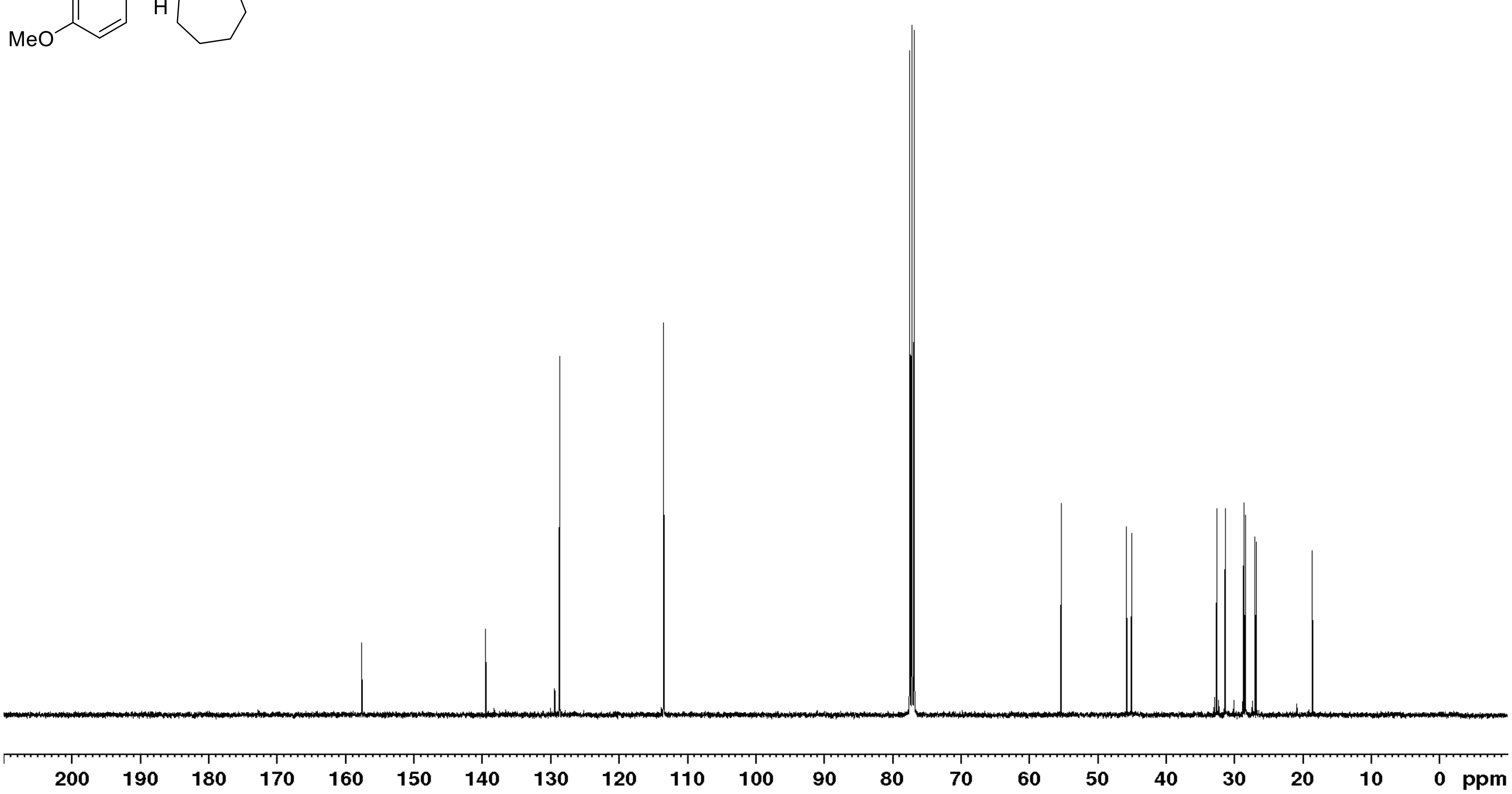

**Figure S133.** $^{1}$H NMR spectrum (400 MHz, $CDCl_3$, 298 K) of **5c**.

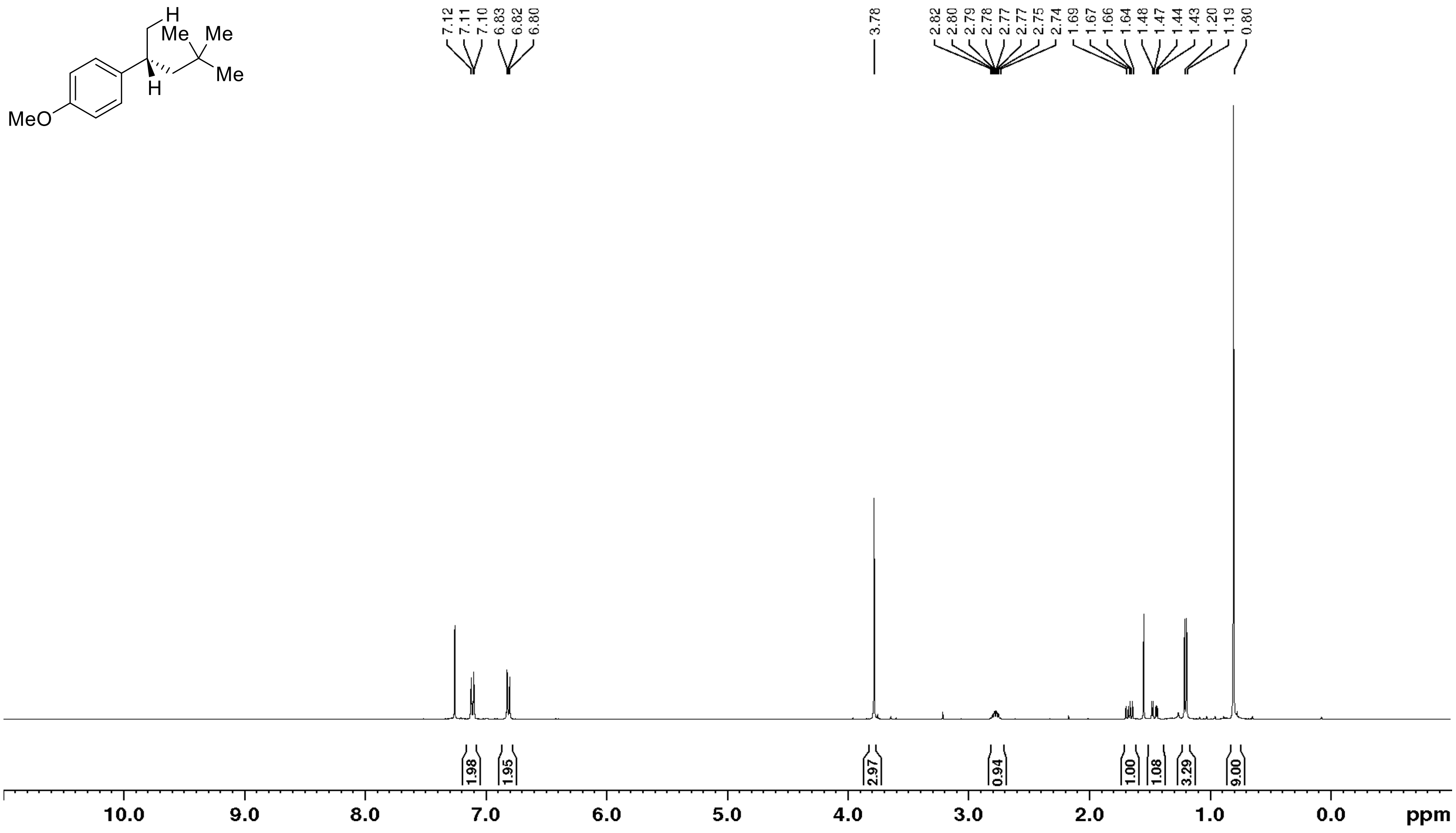

**Figure S134.** $^{13}C\{1H\}$ NMR spectrum (100 MHz, $CDCl_3$, 298 K) of **5c**.

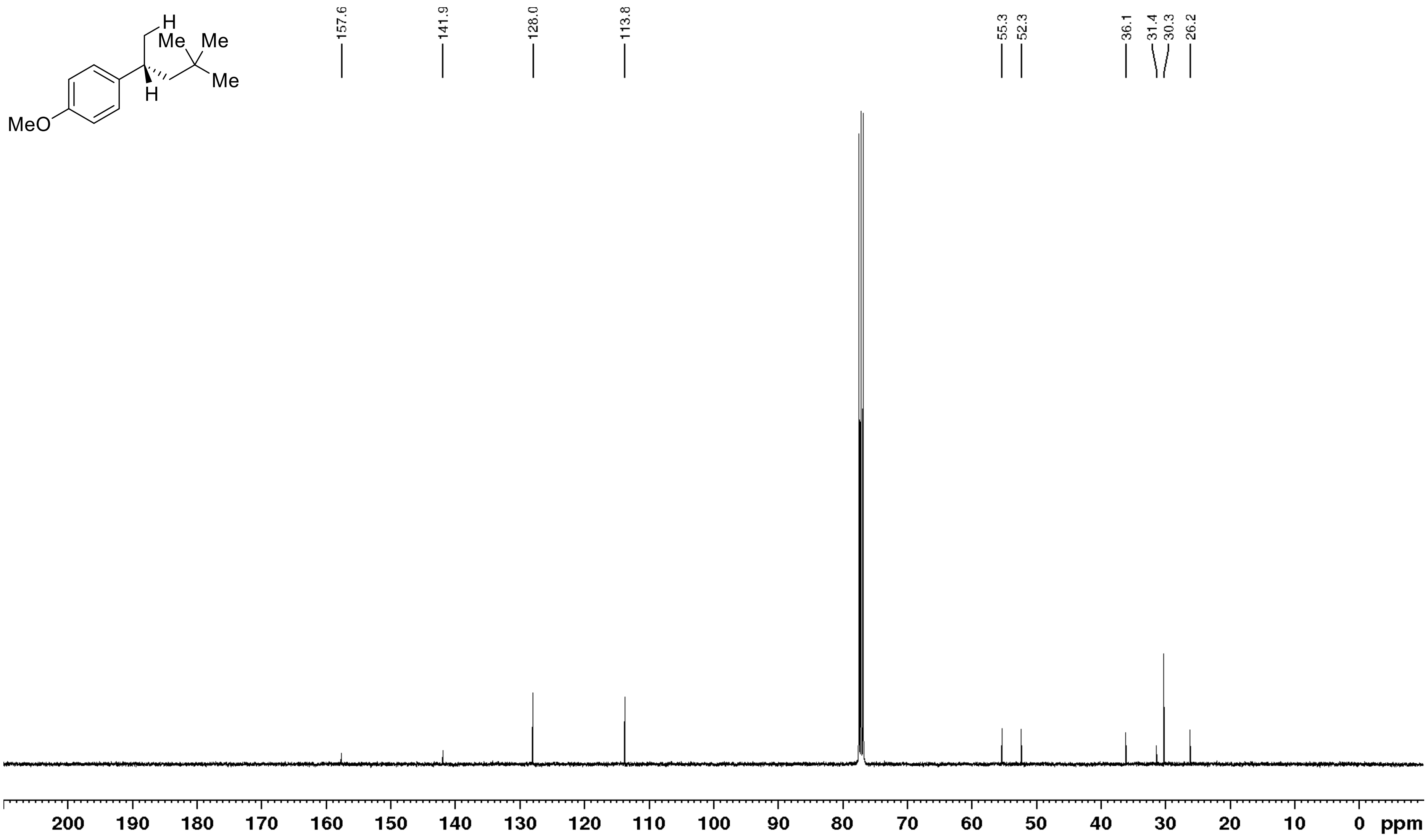

**Figure S135.** $^{1}$H NMR spectrum (400 MHz, $CDCl_3$, 298 K) of **5d**.

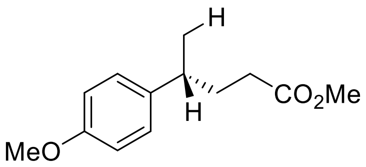

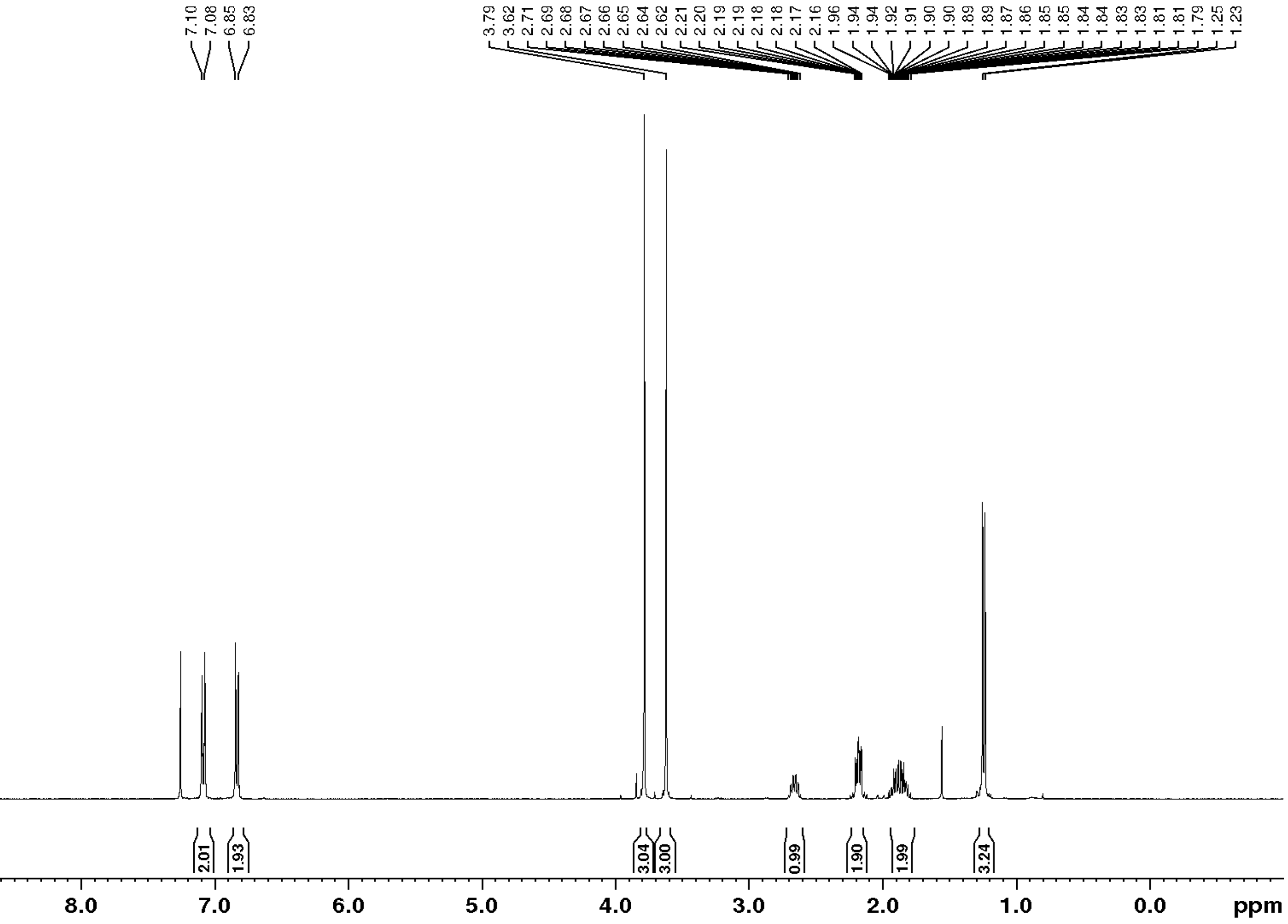

**Figure S136.** $^{13}C\{^1H\}$ NMR spectrum (100 MHz, $CDCl_3$, 298 K) of **5d**.

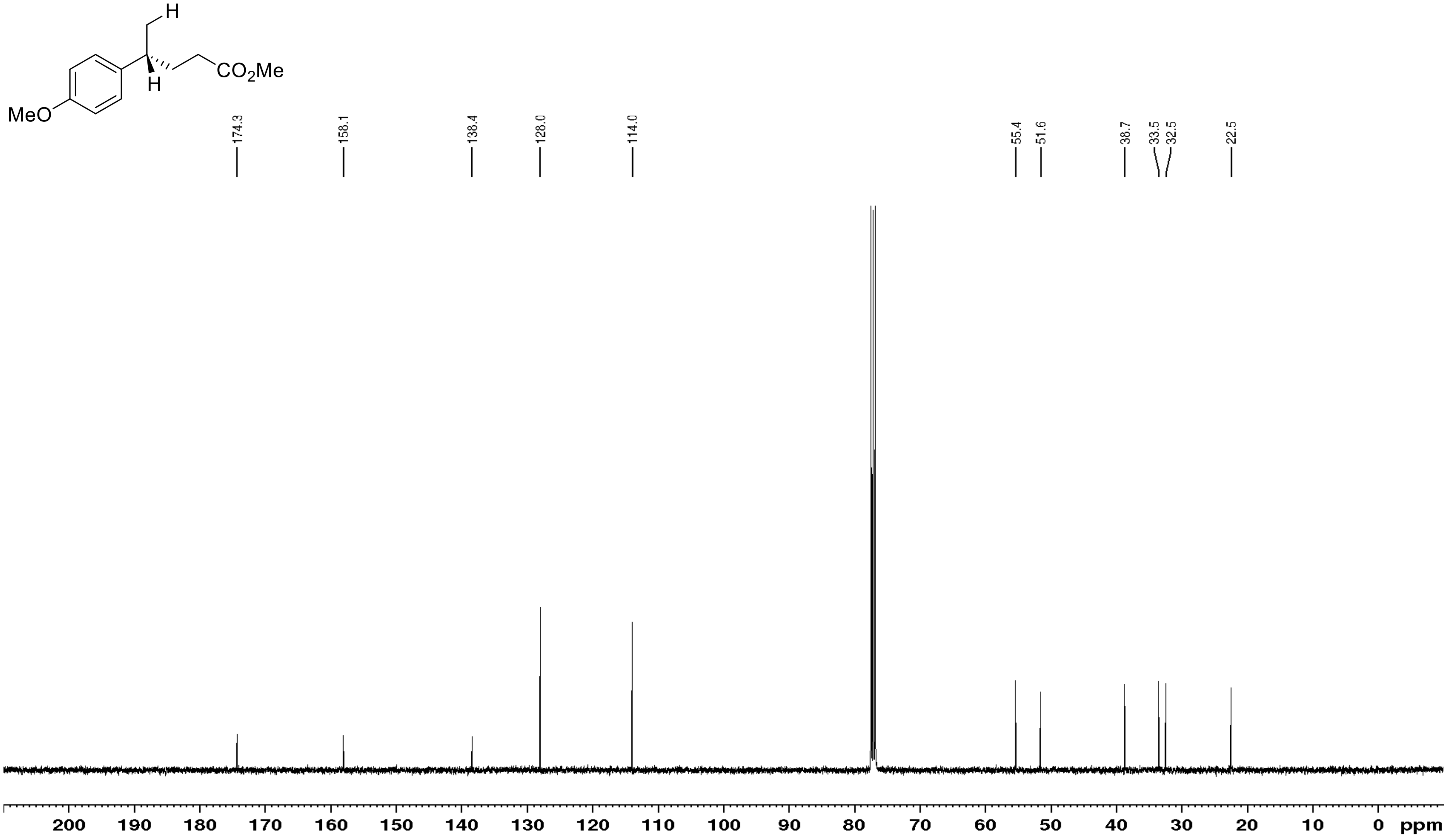

**Figure S137.** $^{1}$H NMR spectrum (400 MHz, $CDCl_3$, 298 K) of **5e**.

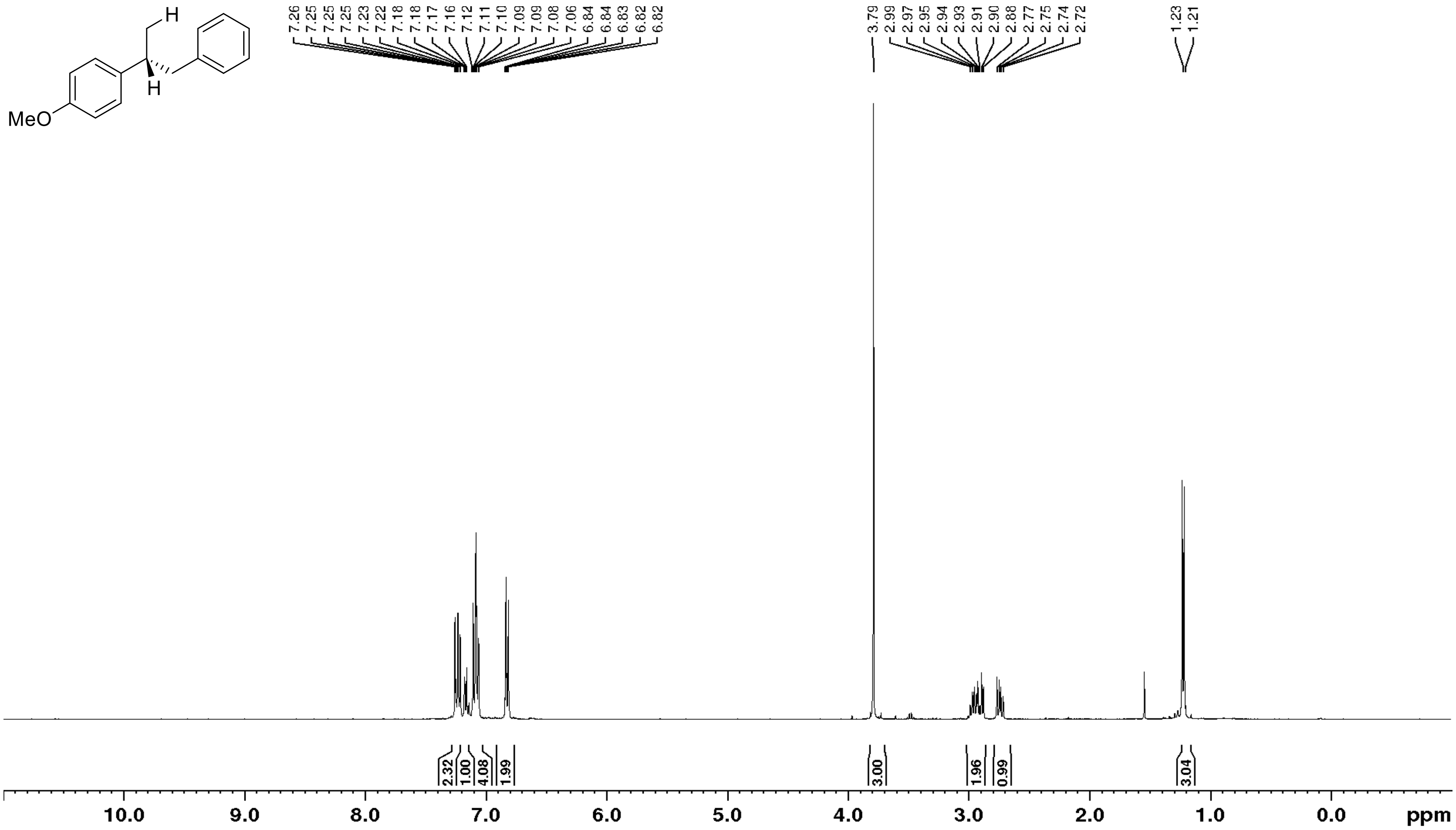

**Figure S138.** $^{13}$C{1H} NMR spectrum (100 MHz, $CDCl_3$, 298 K) of **5e**.

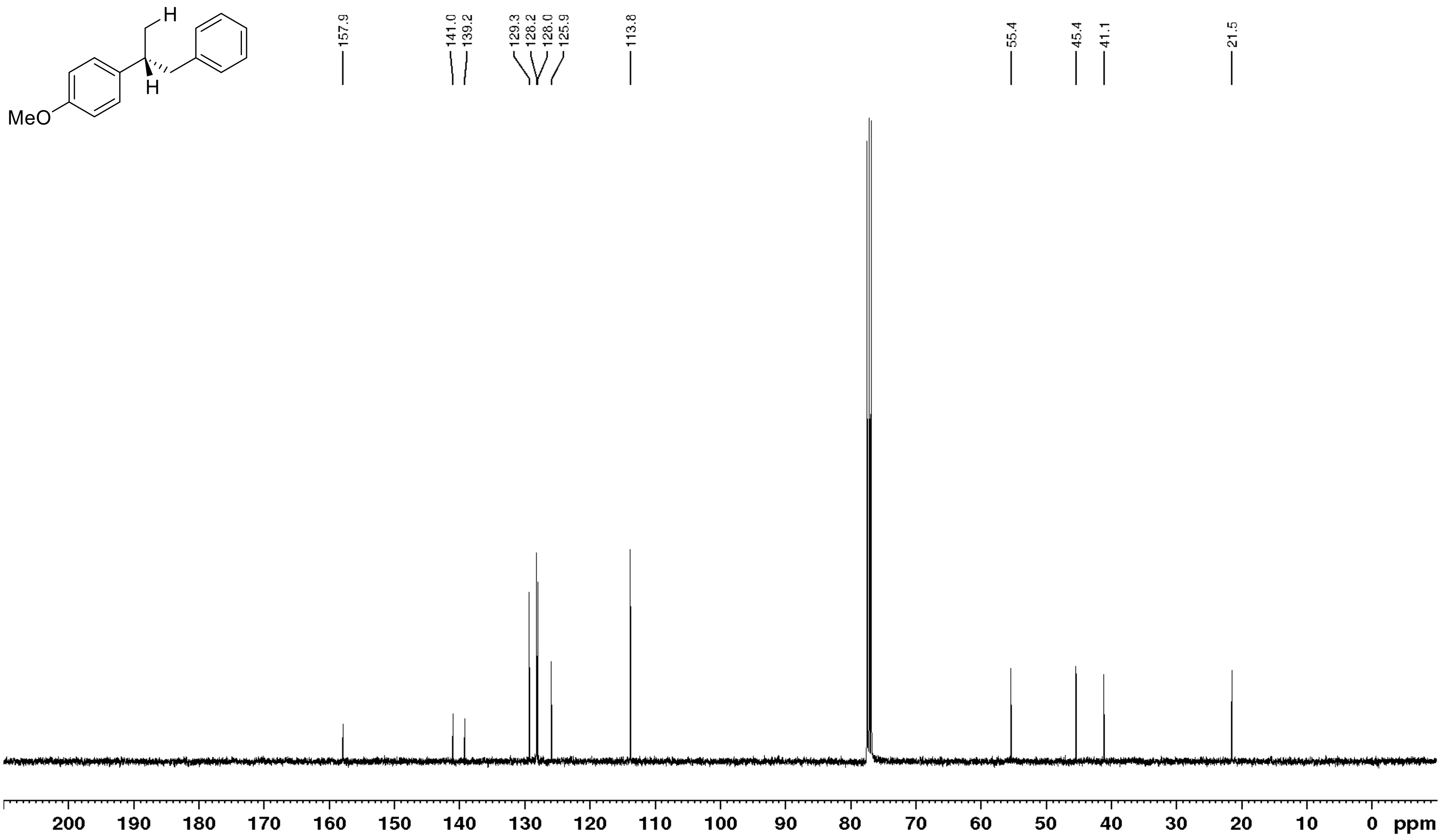

**Figure S139.** $^{1}$H NMR spectrum (400 MHz, $CDCl_3$, 298 K) of **5f**.

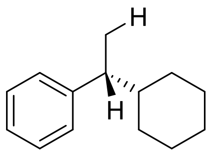

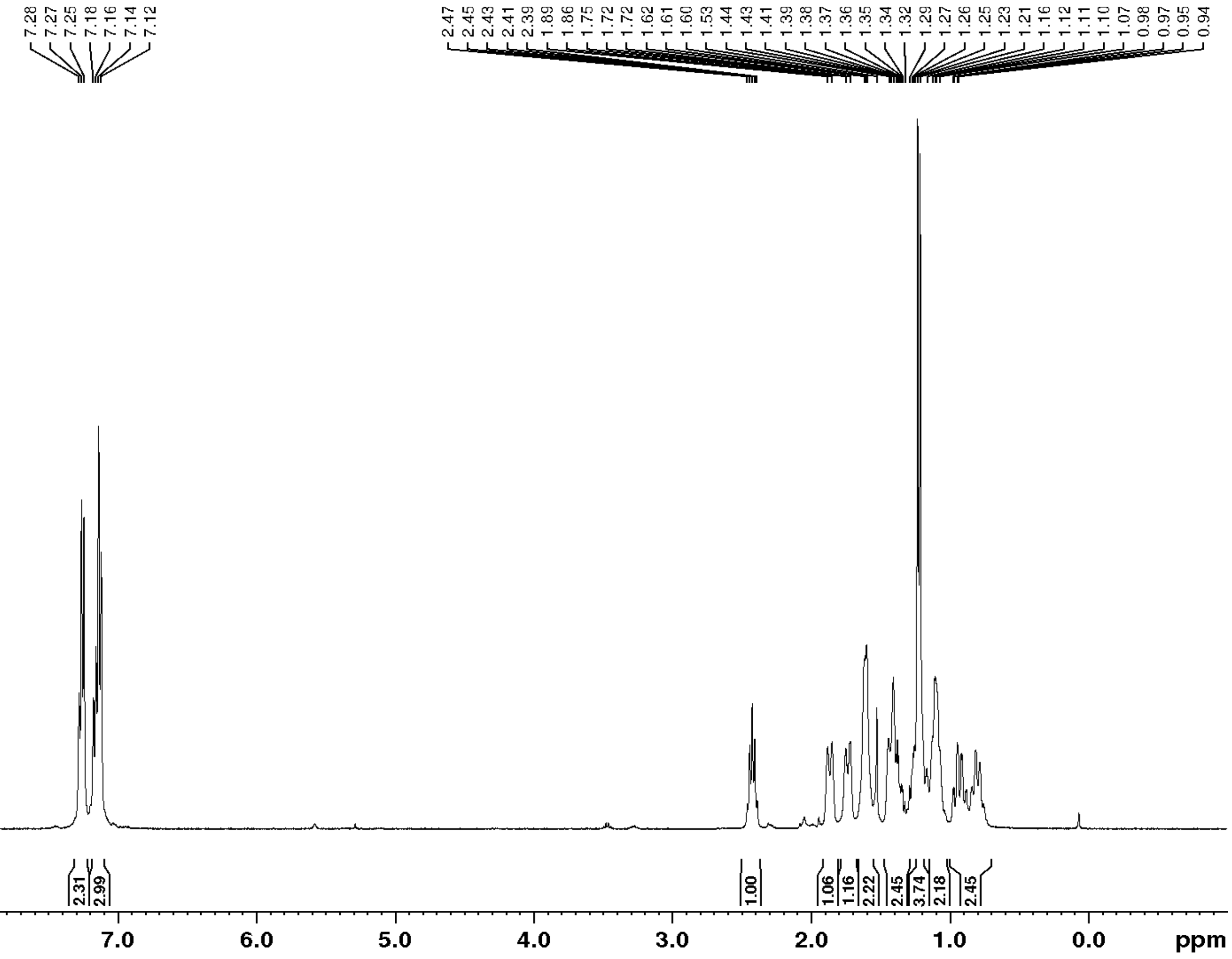

**Figure S140.** $^{13}C\{^1H\}$ NMR spectrum (100 MHz, $CDCl_3$, 298 K) of **5f**.

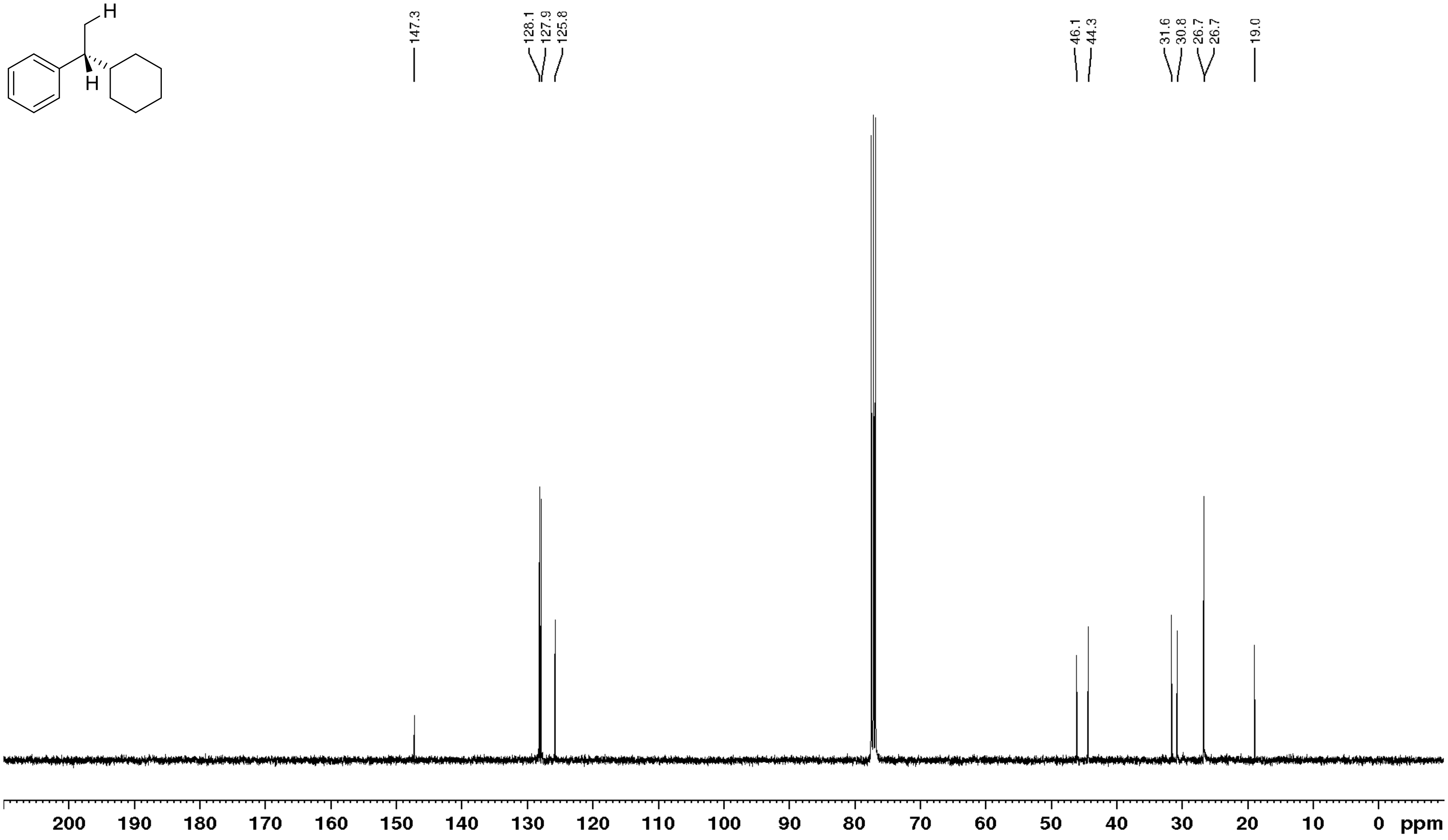

## 10 References

[1] Dipshi, S.; RajanBabu, T. V. Chemodivergent, Regio- and Enantioselective Cycloaddition Reactions between 1,3-Dienes and Alkynes. *Angew. Chem., Int. Ed.* **2023**, *62*, e202216000.

[2] Wolff, B.; Qu, Z.-W.; Grimme, S.; Oestreich, M. Discrimination of the Enantiotopic Faces of Structurally Unbiased Carbenium Ions Employing a Cyclohexadiene-Based Chiral Hydride Source. *Angew. Chem., Int. Ed.* **2023**, *62*, e202305295.

[3] Braconi, E.; Götzinger, A. C.; Cramer, N. Enantioselective Iron-Catalyzed Cross-[4+4]-Cycloaddition of 1,3-Dienes Provides Chiral Cyclooctadienes. *J. Am. Chem. Soc.* **2020**, *142*, 19819–19824.

[4] Mutra, M. R.; Wang J.-J. Photoinduced ynamide structural reshuffling and functionalization. *Nat. Commun*. **2022**, *13*, 2345.

[5] Vo, D.-V.; Karmakar, R.; Lee, D. Reactivity of Enyne-Allenes Generated via an Alder-Ene Reaction. *Org. Lett.*, **2024**, *26*, 1299–1303.

[6] Foster, R. W.; Tame, C. J.; Hailes, H. C.; Sheppard, T. D. Highly Regioselective Synthesis of Substituted Isoindolinones via Ruthenium-Catalyzed Alkyne Cyclotrimerizations. *Adv. Synth. Catal*. **2013**, *355*, 2353–2360.

[7] Vercruysse, S.; Cornelissen, L.; Nahra, F.; Collard, L.; Riant, O. $Cu^I/Pd^0$ Cooperative Dual Catalysis: Tunable Stereoselective Construction of Tetra-Substituted Alkenes. *Chem. Eur. J.* **2014**, *20*, 1834–1838.

[8] Arndt, M.; Risto, E.; Krause, T.; Gooßen, L. J. C–H Carboxylation of Terminal Alkynes Catalyzed by Low Loadings of Silver(I)/DMSO at Ambient $CO_2$ Pressure. *ChemCatChem* **2012**, *4*, 484–487.

[9] Yasuhara, Y.; Nishimura, T.; Hayashi, T. Rhodium-catalyzed aryl- and alkylation–oligomerization of alkynoates with organoboron reagents giving salicylates. *Chem. Commun.* **2010**, *46*, 2130–2132.

[10] Calderone, J. A.; Santos, W. L. Copper(II)-Catalyzed Silylation of Activated Alkynes in Water: Diastereodivergent Access to *E*- or *Z*-β-Silyl-α,β-Unsaturated Carbonyl and Carboxyl Compounds. *Angew. Chem., Int. Ed.* **2014**, *53*, 4154–4158.

[11] Fischer, D. F.; Xin, Z.-Q.; Peters, R. Asymmetric Formation of Allylic Amines with N-Substituted Quaternary Stereocenters by $Pd^{II}$-Catalyzed Aza-Claisen Rearrangements. *Angew. Chem., Int. Ed.* **2007**, *46*, 7704–7707.

[12] Nelson, A. K.; Peck, C. L.; Rafferty, S. M.; Santos, W. L. Chemo-, Regio-, and Stereoselective Copper(II)-Catalyzed Boron Addition to Acetylenic Esters and Amides in Aqueous Media. *J. Org. Chem.* **2016**, *81*, 4269–4279.

[13] Arde, P.; Reddy, V.; Anand, R. V. NHC catalysed trimethylsilylation of terminal alkynes and indoles with Ruppert's reagent under solvent free conditions. *RSC Adv*. **2014**, *4*, 49775–49779.

[14] Sahani, R. L.; Liu, R.-S. Development of Gold-catalyzed [4+1] and [2+2+1]/[4+2] Annulations between Propiolate Derivatives and Isoxazoles. *Angew. Chem., Int. Ed.* **2017**, *56*, 1026–1030.

[15] Singh, D.; RajanBabu, T. V. Chemodivergent, Regio- and Enantioselective Cycloaddition Reactions between 1,3-Dienes and Alkynes. *Angew. Chem., Int. Ed*. **2022**, *62*, e202216000.
